# HCV eradication with IFN-based therapy does not completely restore gene expression in PBMCs from HIV/HCV-coinfected patients

**DOI:** 10.1186/s12929-021-00718-6

**Published:** 2021-03-30

**Authors:** Óscar Brochado, Isidoro Martínez, Juan Berenguer, Luz Medrano, Juan González-García, María Ángeles Jiménez-Sousa, Ana Carrero, Víctor Hontañón, Jordi Navarro, Josep M. Guardiola, Amanda Fernández-Rodríguez, Salvador Resino, the GESIDA Study Group, A. Carrero, P. Miralles, J. C. López, F. Parras, B. Padilla, T. Aldamiz-Echevarría, F. Tejerina, C. Díez, L. Pérez-Latorre, C. Fanciulli, I. Gutiérrez, M. Ramírez, S. Carretero, J. M. Bellón, J. Bermejo, J. Berenguer, V. Hontañón, J. R. Arribas, M. L. Montes, I. Bernardino, J. F. Pascual, F. Zamora, J. M. Peña, F. Arnalich, M. Díaz, J. González-García, P. Domingo, J. M. Guardiola, E. Van den Eynde, M. Pérez, E. Ribera, M. Crespo, J. L. Casado, F. Dronda, A. Moreno, M. J. Pérez-Elías, M. A. Sanfrutos, S. Moreno, C. Quereda, A. Arranz, E. Casas, J. de Miguel, S. Schroeder, J. Sanz, J. Sanz, I. Santos, M. J. Bustinduy, J. A. Iribarren, F. Rodríguez-Arrondo, M. A. Von-Wichmann, J. Vergas, M. J. Téllez, D. Vinuesa, L. Muñoz, J. Hernández-Quero, A. Ferrer, M. J. Galindo, L. Ortiz, E. Ortega, M. Montero, M. Blanes, S. Cuellar, J. Lacruz, M. Salavert, J. López-Aldeguer, G. Pérez, G. Gaspar, M. Yllescas, P. Crespo, E. Aznar, H. Esteban

**Affiliations:** 1 Unidad de Infección Viral E Inmunidad, Centro Nacional de Microbiología, Instituto de Salud Carlos III (Campus Majadahonda), Carretera Majadahonda-Pozuelo, Km 2.2, 28220 MajadahondaMadrid, Spain; 2 Unidad de Enfermedades Infecciosas/VIH, Hospital General Universitario “Gregorio Marañón”, Madrid, Spain; 3 Instituto de Investigación Sanitaria del Gregorio Marañón, Madrid, Spain; 4 Unidad de VIH, Servicio de Medicina Interna, Hospital Universitario “La Paz”, Madrid, Spain; 5 Instituto de Investigacion Sanitaria La Paz (IdiPAZ), Madrid, Spain; 6 Servicio de Enfermedades Infecciosas, Hospital Universitari Vall D’Hebron, Barcelona, Spain; 7 Institut de Recerca Vall D’Hebron, Barcelona, Spain; 8 Hospital Santa Creu I Sant Pau, Barcelona, Spain

**Keywords:** HIV/HCV coinfection, Gene expression, Immune system, HCV clearance, Interferon therapy, PBMCs

## Abstract

**Objective:**

To evaluate the impact of hepatitis C virus (HCV) elimination via interferon (IFN)-based therapy on gene expression profiles related to the immune system in HIV/HCV-coinfected patients.

**Methods:**

We conducted a prospective study in 28 HIV/HCV-coinfected patients receiving IFN-based therapy at baseline (HIV/HCV-b) and week 24 after sustained virological response (HIV/HCV-f). Twenty-seven HIV-monoinfected patients (HIV-mono) were included as a control. RNA-seq analysis was performed on peripheral blood mononuclear cells (PBMCs). Genes with a fold-change (FC) ≥ 1.5 (in either direction) and false discovery rate (FDR) ≤ 0.05 were identified as significantly differentially expressed (SDE).

**Results:**

HIV/HCV-b showed six SDE genes compared to HIV-mono group, but no significantly enriched pathways were observed. For HIV/HCV-f vs. HIV/HCV-b, we found 58 SDE genes, 34 upregulated and 24 downregulated in the HIV/HCV-f group. Of these, the most overexpressed were *CXCL2*, *PDCD6IP, ATP5B, IGSF9, RAB26,* and *CSRNP1*, and the most downregulated were *IFI44* and *IFI44L*. These 58 SDE genes revealed two significantly enriched pathways (FDR < 0.05), one linked to Epstein-Barr virus infection and another related to p53 signaling. For HIV/HCV-f vs. HIV-mono group, we found 44 SDE genes that revealed 31 enriched pathways (FDR < 0.05) related to inflammation, cancer/cell cycle alteration, viral and bacterial infection, and comorbidities associated with HIV/HCV-coinfection. Five genes were overrepresented in most pathways (*JUN, NFKBIA, PIK3R2, CDC42,* and *STAT3*).

**Conclusion:**

HIV/HCV-coinfected patients who eradicated hepatitis C with IFN-based therapy showed profound gene expression changes after achieving sustained virological response. The altered pathways were related to inflammation and liver-related complications, such as non-alcoholic fatty liver disease and hepatocellular carcinoma, underscoring the need for active surveillance for these patients.

**Supplementary Information:**

The online version contains supplementary material available at 10.1186/s12929-021-00718-6.

## Introduction

Hepatitis C virus (HCV) infection is a leading cause of chronic liver disease worldwide [1]. HCV and human immunodeficiency virus (HIV) share transmission routes and many HIV-infected individuals are coinfected with HCV [2]. HIV/HCV-coinfected patients develop chronic hepatitis C over decades of infection, but the cirrhosis progression is faster than in HCV-monoinfected patients [3], leading to higher rates of liver-related events (LREs) such as liver decompensation, end-stage liver disease, hepatocellular carcinoma (HCC), and liver-related death [4–7]. Chronic HCV infection promotes inflammation, immune activation, and dysregulation of immune function [8, 9], which boosts the development of LRE and other comorbidities in infected people [7–9]. HIV infection also aggravates the dysregulation of the immune system [7, 8], since many disturbances remain in patients infected with HIV after suppressive antiretroviral therapy (ART), such as deficits in T-cell functions [10–15], immune activation [13], inflammation [16], and dysbiosis [17–19]. All these immunological alterations increase the risk of AIDS, non-AIDS-related events, and death [20, 21].

For over a decade, interferon (IFN)-based therapy was the mainstay of antiviral treatment against HCV. However, the appearance of direct-acting antivirals (DAAs) has revolutionized HCV therapy because it has allowed almost all treated patients to achieve sustained virological responses (SVR) [22–25]. HCV clearance after peg-IFN-α/ribavirin treatment decreases the risk of clinical events and death in HIV/HCV-coinfected patients [26, 27]. However, liver fibrosis regression after SVR with peg-IFN-α/ribavirin is slow since some of the cured patients maintain a low rate of liver fibrosis progression [28, 29] and develop LREs [30–32], making monitoring of the cirrhotic patient necessary after the eradication of HCV. Moreover, peg-IFN-α/ribavirin treatment is associated with numerous side effects, some of which may require long-term follow-up [33]. This concern is even greater for HIV/HCV-coinfected patients since, in this population, peg-IFN-α/ribavirin therapy shows higher rates of adverse side effects.

Transcriptome analysis has emerged as a key tool for profiling the immune response against pathogens. Peripheral blood transcriptome analysis may provide relevant information on the host response in chronic hepatitis C [34] and after overcoming HCV infection via spontaneous HCV clearance [35], peg-IFN-α/ribavirin treatment [36], or DAA therapy [37, 38]. There is little information about the long-term impact of successful peg-IFN-α/ribavirin treatment on the peripheral blood transcriptome. The only article found on this topic shows that inflammatory gene expression persists in peripheral blood mononuclear cells (PBMCs) after achieving SVR with peg-IFN treatment [39].

### Objective

We aimed to evaluate the impact of HCV elimination via IFN-based therapy on gene expression profiles related to the immune system in HIV/HCV-coinfected patients.

### Patients and methods

#### Study subjects

We carried out a prospective study (before and after design) on 28 HIV/HCV-coinfected patient samples collected between 2012 and February 2016. In this study, patients were a subgroup from the GESIDA 3603b study (see Additional file 7: Appendix S1) previously described [40]. The study was carried out strictly following the Declaration of Helsinki and was approved by the Research Ethics Committee of the Instituto de Salud Carlos III (CEI PI 23_2011). Before enrolment, all the participants signed written consent.

The selection criteria of HIV/HCV-coinfected patients were: 1) chronic HCV and HIV infection; 2) starting IFN-based therapy (peg-IFN-α/ribavirin or peg-IFN-α/ribavirin/DAAs) and achieving SVR; 3) CD4+ T cell ≥200 cells/µL; 4) stable ART ≥6 months; 4) frozen PBMCs samples available. The exclusion criteria were: 1) hepatitis B virus coinfection; 2) acute hepatitis C; 3) hepatic decompensation; 4) HCC.

Additionally, 27 HIV-monoinfected patients (HIV-mono) were used as a control group, matched by gender and age with the HIV/HCV-coinfected patient group. Individuals in the HIV-mono group were negative for HCV and HBV infections and had undetectable HIV viral load and CD4^+^ >500 cells/µl (normal standard for HIV-infected patients). We included this HIV-mono group as a reference point for HIV-monoinfected patients with stable HIV disease.

#### Clinical data and Samples

Clinical data were collected prospectively using an online form. Subsequently, the database was monitored to verify the data collected. Peripheral venous blood samples were collected in EDTA tubes, and PBMCs were isolated by Ficoll-Paque gradient. The viable PBMCs were stored in liquid nitrogen at the Spanish HIV HGM BioBank until use. The clinical data and samples used in this study were from the beginning of HCV treatment (HIV/HCV-b) and 24 weeks after SVR (HIV/HCV-f).

#### RNA extraction, library preparation, and sequencing

The RNeasy Minikit (Qiagen™) was used for total RNA extraction from PBMCs following the manufacturer's instructions. Then, the RNA quantity and quality were evaluated by Nanodrop 2000 and 2100 Bioanalyzer RNA NANO assay (Agilent). Only those samples with an RNA integrity number higher than 7.5 were selected for sequencing. DNAse treatment was performed with the RNase-free DNase Set (Qiagen). Poly-A RNA libraries and sequencing were performed at the Centre for Genomic Regulation in Barcelona (Spain). Briefly, Illumina's TruSeq Stranded mRNA Sample Prep Kit v2 was used on 500 nanograms of total RNA for library synthesis. In this way, coding and multiple forms of non-coding RNAs are captured because of polyadenylation. Ten libraries were multiplexed and pooled on each line of the Illumina HiSeq2500 sequencer to obtain an average of 25 million reads per sample, with a single read, 50nts (1x50), approach.

We followed a specific bioinformatics pipeline that has been set out in detail in Additional file 1:Table S1. We used FastQC (v. 0.11.8) for the quality control test. The adapter trimming step was carried out with Trimmomatic (v. 0.33). TopHat (v. 2.0.14) with GRCH38 was used as a reference genome to perform the alignment and HTSeq for count extraction (v. 0.6.1).

#### Genes selected for analysis

**Fig. 1:**
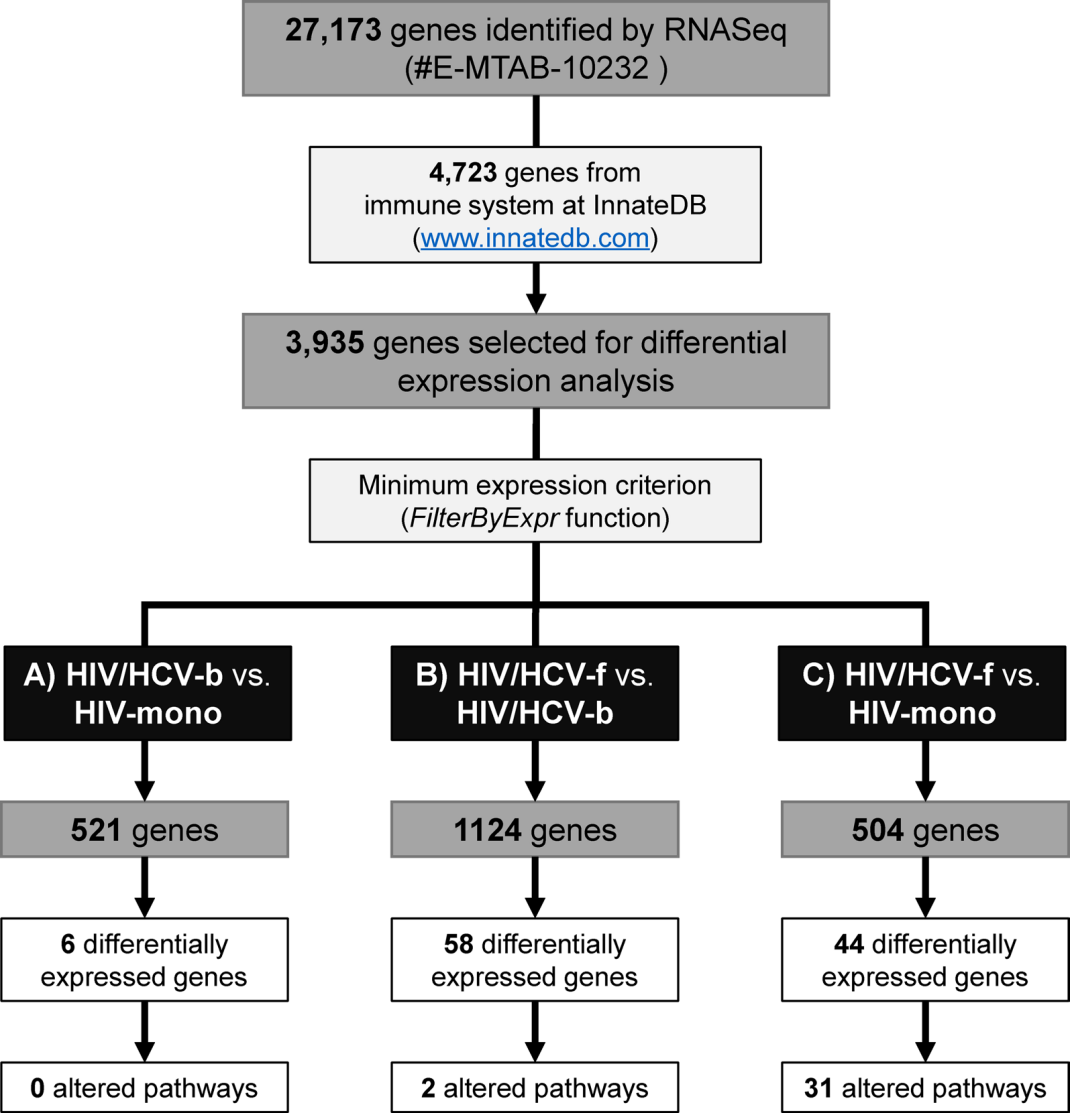
Flowchart of the study design. A total of 27,173 genes were identified by RNA sequencing. Of these, 4723 genes from the immune system were selected from InnateDB, and 3935 genes were selected for differential expression analysis. The FilterByExpr function was applied for each comparison: **a** HIV/HCV-b vs. HIV-mono, 521 genes were selected, six were SDE genes not involved in any biological pathway; **b** HIV/HCV-f vs. HIV/HCV-b, 1124 genes were selected, 58 were SDE genes involved in two biological pathways; **c** HIV/HCV-f vs. HIV-mono, 504 genes were selected, 44 were SDE genes involved in 31 biological pathways. HIV, human immunodeficiency virus; HCV, hepatitis C virus; HIV/HCV-b, HIV/HCV-coinfected patients at baseline; HIV/HCV-f, HIV/HCV-coinfected patients 24 weeks after SVR; HIV-mono, HIV-monoinfected patients; SDE, significantly differentially expressed

Figure 1 shows the flowchart of the gene selection process. The bioinformatics analysis identified a gene universe composed of 27,173 different genes. Samples showed an average of about 24 million reads, from which 99.07% of them mapped to the genome. Raw sequence data are publicly available at the ArrayExpress repository (EMBL-EBI; https://www.ebi.ac.uk/arrayexpress/) under the accession number E-MTAB-10232.

From this initial gene universe, we proposed to analyze 4723 genes related to the immune system according to InnateDB (Additional file 2:Table S2), a public database of human immune genes (www.innatedb.com). However, we only found 3935 (83%) genes in our initial gene universe out of the total listed in the InnateDB (Fig. 1).

#### Calculation of the sample size

The sample size for each group was calculated according to the RNASeqPower [41], which established a minimum of 25.6 samples per group to reject the null hypothesis (the two groups are equal). Calculates were performed using the following parameters: 25 million reads per sample, an estimated dispersion of 0.4, an alpha of 0.05, and a minimum fold change of 1.5. Thus, the longitudinal study (28 vs. 28) and the independent comparisons (28 vs. 27) showed a statistical power of 0.92.

### Statistical analysis

We used the Statistical Package for the Social Sciences (SPSS) v22.0 software (IBM Corp., Chicago, USA) to perform descriptive statistical analyzes of the study populations. Differences were calculated by Mann-Whitney tests and Pearson’s chi-squared test.

We used the R statistical package version v3.4.1 (R Foundation for Statistical Computing, Vienna, Austria) for gene expression analysis. The Trimmed Mean of M (TMM) was used to normalize the data (R-package *edgeR* v3.20.9) after selecting genes by using the list of immune system-related genes (see the previous section). We carried out the TMM normalization for each of the comparisons (HIV/HCV-b vs. HIV-mono, HIV/HCV-f vs. HIV/HCV-b, and HIV/HCV-f vs. HIV-mono). Next, we used the *FilterByExpr* function with default parameters to apply a minimum expression criterion and select just those genes well represented among all samples. Finally, we explored the gene expression between groups by a generalized linear mixed model (GLMM) for paired samples and a generalized linear model (GLM) for non-paired comparison (R-package *lme4* V. 1.1-23), both with a negative binomial distribution. Finally, we used NetworkAnalyst v3.0 to find enriched pathways and networks.

We calculated the fold-change (FC) and log2-FC for each gene for measuring the changes in different groups. *P-values* were two-tailed and corrected for multiple testing using the false discovery rate (FDR) with the Benjamini and Hochberg method (*q*-values) to reduce the risk of spurious results. Significantly differentially expressed (SDE) genes were those with FDR ≤0.05 and log2-FC ≤ − 0.584 (1.5-fold downregulated) or log2-FC ≥0.584 (1.5-fold upregulated).

## Results

### Patient characteristics at baseline

Table 1 shows the baseline characteristics of the patients included in this study. Overall, HIV/HCV-coinfected patients showed higher alcohol intake values, HIV acquisition by injection drug use, and FIB4. Also, HIV/HCV-coinfected individuals had lower values of CD4+ T-cells than HIV-monoinfected patients.

**Table 1:** Clinical, epidemiological, and virological characteristics of HIV and HIV/HCV-coinfected patients

	HIV	HIV/HCV	*p*-value
No	27	28	
Age (years)	51 (46; 53)	48.5 (45.5; 53)	0.272
Gender (male)	16 (59.3%)	19 (67.9%)	0.508
BMI (kg/m^2^)	25 (23.4; 26.7)	24 (20.8; 26)	0.125
BMI ≥ 25 (kg/m^2^)	14 (51.9%)	9 (32.1%)	0.139
Diabetes	4 (14.8%)	2 (7.1%)	0.362
High alcohol intake	1 (3.7%)	13 (46.4%)	< 0.001
HIV acquired by IVDU	0 (0%)	22 (78.6%)	< 0.001
Prior AIDS	10 (37%)	8 (28.6%)	0.504
Years since HIV infection		22.5 (17.5; 26.5)	–
Years since HCV diagnosis		13 (12; 23)	–
Previous HCV therapy (IFNα + rib)		13 (46.4%)	–
Antiretroviral therapy	27 (100%)	28 (100%)	0.999
PI-based	6 (22.2%)	6(21.43%)	0.019
2NRTI + II-based	3 (11.1%)	11 (39.3%)	
2NRTI + PI-based	0 (0%)	3 (10.7%)	
2NRTI + NNRTI-based	16 (59.3%)	7 (25%)	
Others	2 (7.4%)	1 (3.6%)	
HIV markers			
Nadir CD4 + T-cells	261 (99; 402)	185 (72; 269)	0.155
Nadir CD4 + T-cells < 200 cells/mm^3^	11 (44%)	15 (53.6%)	0.487
CD4 + T-cells	804 (685; 1036)	678.5 (446.5; 906)	0.026
CD4 + T-cells < 500 cells/mm^3^	0 (0%)	8 (28.6%)	0.003
HIV-RNA > 50 cp/mL	0 (0%)	0 (0%)	–
HCV markers			
HCV genotype			
1		18 (64.3%)	–
2		1 (3.6%)	
3		8 (28.6%)	
4		1 (3.6%)	
Log_10_ HCV-RNA (IU/mL)		6.3 (5.8; 6.6)	–
HCV-RNA > 850.000 IU/mL		19 (67.9%)	–
Non-invasive fibrosis indexes			
FIB-4	1 (0.9; 1.2)	2.6 (1.9; 3.2)	< 0.001
FIB-4 ≥ 3.25	0 (0%)	7 (25%)	0.006
LSM (Kpa)		11.9 (9.7; 19.2)	–
F0–F1 (< 7.1 kPa)		2 (7.1%)	–
F2 (7.1–9.4 kPa)		5 (17.9%)	
F3 (9.5–12.4 kPa)		8 (28.6%)	
F4 (≥ 12.5 kPa)		13 (46.4%)	

Statistics: The values are expressed as the absolute number (percentage) and median (interquartile range). *P-values* were calculated by Mann–Whitney and chi-squared tests

HCV, hepatitis C virus; HCV-RNA, HCV plasma viral load; HIV, human immunodeficiency virus; LSM, liver stiffness measure; HIV-RNA, HIV plasma viral load; IVDU, intravenous drug user; AIDS, acquired immune deficiency syndrome; IFNα + rib, interferon-alpha plus ribavirin; NNRTI, non-nucleoside analogue HIV reverse transcriptase inhibitor; NRTI, nucleoside analogue HIV reverse; BMI, body mass index

### Differences in gene expression at baseline (HIV/HCV-b vs. HIV-mono)

**Fig. 2:**
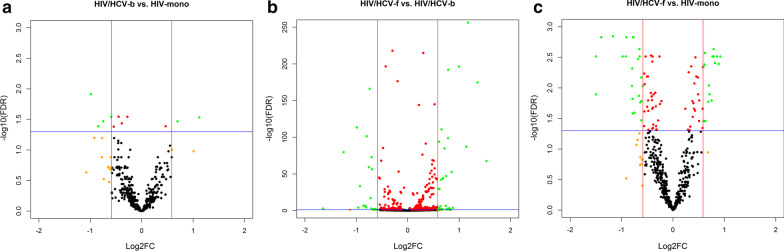
Volcano plots for differentially expressed genes between groups: **a** HIV/HCV-b versus HIV-mono, **b** HIV/HCV-f versus HIV/HCV-b, **c** HIV/HCV-f versus HIV-mono. Volcano-plot discriminates by FDR and log2(FC). The red vertical lines represent the cut-off of FC 1.5 and the blue horizontal line indicates the cut-off of FDR = 0.05. Green dots represent genes significantly altered with FDR ≤ 0.05 and absolute FC ≥ 1.5. FDR, false discovery rate for multiple comparisons using Benjamini and Hochberg procedure; FC, fold-change; HIV, human immunodeficiency virus; HCV, hepatitis C virus; HIV/HCV-b, HIV/HCV-coinfected patients at baseline; HIV/HCV-f, HIV/HCV-coinfected patients 24 weeks after SVR; HIV-mono, HIV-monoinfected patients

A total of 521 genes fitted the expression criteria for the comparison between HIV/HCV-b vs. HIV-mono group (Fig. 1), but only six SDE genes were found (Fig. 2a, full description in Additional file 3:Table S3A): four upregulated in the HIV/HCV-b group (*IL23A*, FKBP15, *CALR*, and *DDIT3*) and two downregulated (*TLR5* and *STXBP1*). The analysis of the enriched pathways did not show statistically significant results.

### Changes in gene expression during follow-up (HIV/HCV-f vs. HIV/HCV-b)

A total of 1124 genes fulfilled the expression criteria (Fig. 1), and 58 genes were SDE (Fig. 2b, full description in Additional file 3:Table S3B). Thirty-four genes were upregulated in the HIV/HCV-f group, but only six genes were higher than 2-fold (*CXCL2*, *PDCD6IP, ATP5B, IGSF9*, *RAB26*, and *CSRNP1)*. Twenty-four genes were downregulated in the HIV/HCV-f group, but only two genes were less than 0.5-fold (*IFI44* and *IFI44L*).

We searched for significantly enriched pathways in the SDE genes list, and two significant pathways were identified (Additional file 4:Table S4; *q*-value <0.05). One was the Epstein-Barr virus infection pathway (*q*-value=0.025), represented by four upregulated genes (*CDKN1A, NFKB2, RELB,* and *PDIA3*) and two downregulated genes (*BID* and *HLA-A*). The other was the p53 signaling pathway (*q*-value= 0.025), represented by two SDE upregulated genes (*CDKN1A* and *PMAIP1*) and two downregulated genes (*BID* and *TP73*).

### Differences in gene expression at the end of the follow-up to the control group (HIV/HCV-f vs. HIV-mono)

Finally, 504 genes fulfilled the expression criteria when comparing HIV/HCV-f vs. HIV-mono (Fig. 1), and 44 genes were SDE (Fig. 2c, full description in Additional file 3: Table S3C). Twenty-six SDE genes were upregulated in the HIV/HCV-f group, but only five were higher than 2-fold upregulated (*KLF6, HSPA5, JUN, PRRC2C,* and *PPP1R15A*). Eighteen SDE genes were downregulated, but none were less than 0.5-fold.

**Fig. 3:**
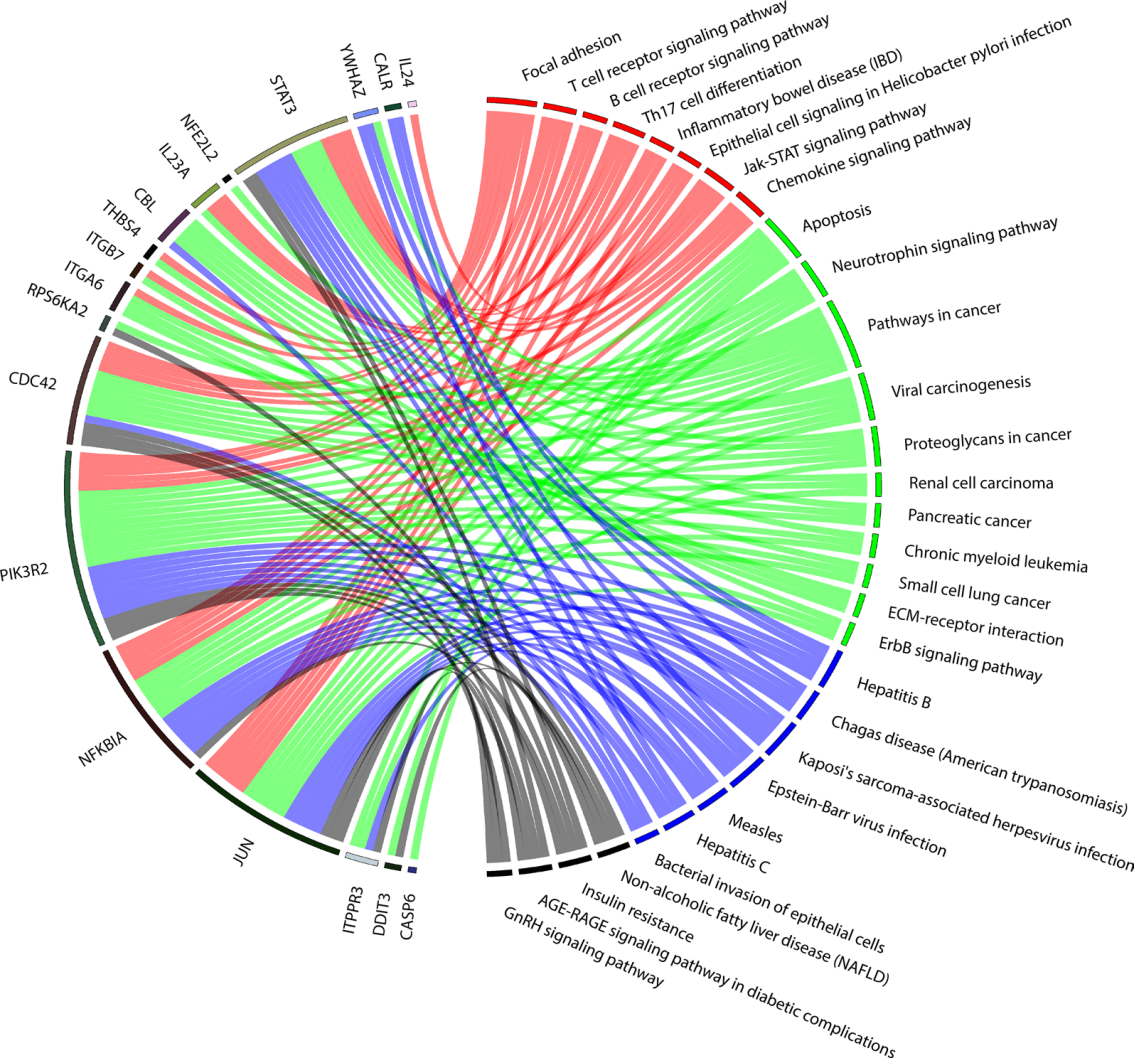
The chord-diagram represents connections between SDE genes (left-side) and pathways (right-side) in comparing HIV/HCV-f versus HIV-mono. Red corresponds to inflammation, green corresponds to cell cycle alteration and cancer, blue corresponds to viral and bacterial infection, and black corresponds to complications related to HIV/HCV-coinfection. HIV, human immunodeficiency virus; HCV, hepatitis C virus; HIV/HCV-b, HIV/HCV-coinfected patients at baseline; HIV/HCV-f, HIV/HCV-coinfected patients 24 weeks after SVR; HIV-mono, HIV-monoinfected patients; SDE, significantly differentially expressed

The pathway analysis showed 31 enriched pathways related to four main topics (Fig. 3, full description in Additional file 5:Table S5): (i) inflammation, (ii) cancer/cell cycle alteration, (iii) viral and bacterial infection, and (iv) comorbidities related to HIV/HCV-coinfection. Overall, five genes were overrepresented among the majority of the altered pathways (*JUN, NFKBIA, PIK3R2, CDC42,* and *STAT3)*.

**Fig. 4:**
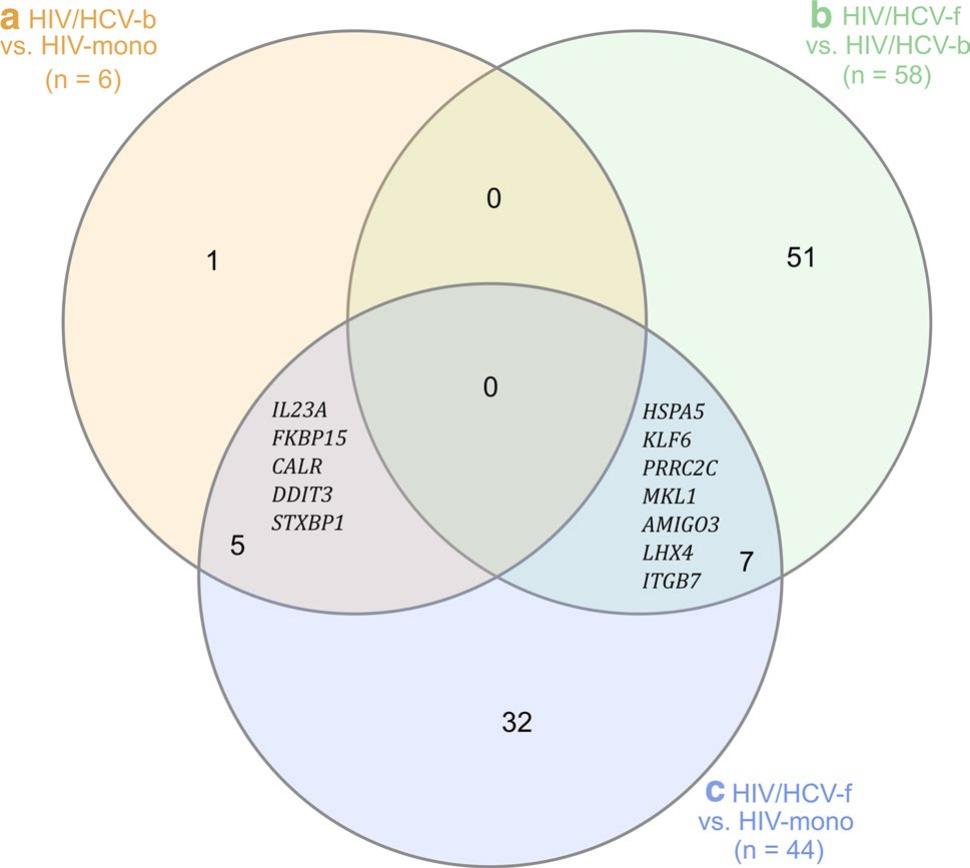
Venn diagram of the SDE genes in the three comparisons analyzed in the study: **a** HIV/HCV-b versus HIV-mono, **b** HIV/HCV-f versus HIV/HCV-b, **c** HIV/HCV-f versus HIV-mono. The number in each circle corresponds to the number of SDE genes in the different comparisons. The overlapping numbers are the number of SDE genes shared between the different comparisons. The non-overlapping numbers are the unique SDE genes in each comparison. HIV, human immunodeficiency virus; HCV, hepatitis C virus; HIV/HCV-b, HIV/HCV-coinfected patients at baseline; HIV/HCV-f, HIV/HCV-coinfected patients 24 weeks after SVR; HIV-mono, HIV-monoinfected patients

Finally, the HIV/HCV-b vs. HIV-mono and the HIV/HCV-f vs. HIV-mono comparisons had four upregulated genes in common (*IL23A*, *FKBP15*, *CALR*, and *DDIT3*) and one downregulated gene (*STXBP1*) (Fig. 4, Additional file 3: Table S3). Therefore, these five SDE genes remained deregulated at 24-week after SVR. Additionally, three SDE genes were upregulated (*HSPA5, KLF6*, and *PRRC2C*), and four downregulated (*MKL1, AMIGO3, LHX4*, and *ITGB7*) both in the HIV/HCV-f vs. HIV/HCV-b and the HIV/HCV-f vs. HIV-mono comparisons (Fig. 4, Additional file 3:Table S3).

### Changes in metabolic, HIV-related disease, and liver disease profiles (HIV/HCV-f vs. HIV/HCV-b)

At the end of follow-up, we found significant increases in levels of cholesterol (p=0.022), CD4^+^ T-cells/mm^3^ (p=0.026), and albumin (p=0.045); while values of LSM (p=0.002), FIB4 (p<0.001), AST (p<0.001), and ALT (p<0.001) significantly decreased (Table 2).

**Table 2:** Summary of metabolic, HIV disease, and liver disease characteristics in HIV/HCV-coinfected patients at baseline and the end of follow-up

Variable–median (IQR)	Baseline	Final	*p*-value
Metabolic profile			
Glucose (mg/dL)	97.5 (83.8; 103)	93 (86.5; 96.5)	0.967
Triglycerides (mg/dL)	108 (78.5; 169.5)	145 (99.5; 189.3)	0.063
Cholesterol (mg/dL)	169 (139.5; 194.5)	183.5 (169.8; 208.5)	**0.022**
LDL (mg/dL)	104 (76.3; 118.8)	110 (97; 135)	0.071
HDL (mg/dL)	48 (36; 56.5)	36 (33; 50)	0.119
HIV disease profile			
CD4^+^ T-cells/mm^3^	678 (446; 911)	827 (588; 1131)	**0.026**
CD8^+^ T-cells/mm^3^	946 (555; 1356)	1093 (840; 1308)	0.531
HIV-viral load > 50 copies/mL (%)	0/28 (0%)	4/26 (15.4%)	0.982
Liver disease profile			
LSM (kPa)	11.9 (9.6; 19.9)	7.6 (5.9; 13.9.)	**0.002**
FIB4	2.6 (1.9; 3.2)	1.7 (1.2; 2.1)	**0.000**
AST (UI/L)	61 (42.5; 79.3)	19 (13.8; 25)	**0.000**
ALT (UI/L)	71.5 (56; 100.5)	33.5 (26.5; 42)	**0.000**
GGT (UI/L)	41 (35.5; 44)	33.5 (26.5; 42)	0.951
Alkaline phosphatase (UI/L)	94.5 (78; 117)	95 (85; 120)	0.381
INR	1 (1; 1.1)	1 (0.95; 1.1)	0.353
Bilirubin (mg/dL)	0.6 (0.5; 1.15)	0.6 (0.4; 0.8)	0.177
Albumin (g/dL)	4.3 (3.9; 4.6)	4.5 (4.2; 4.7)	**0.045**

Statistics: The values are expressed as the median (IQR, interquartile range). *P-values* were calculated by generalized linear mixed models (GLMM) for paired samples

HCV, hepatitis C virus; HIV, human immunodeficiency virus; LSM, liver stiffness measure; LDL, low-density lipoprotein; HDL, high-density lipoprotein; LSM; liver stiffness measure; FIB4; fibrosis-4 index; ALT, alanine aminotransferase; AST, aspartate transaminase; GGT, gamma-glutamyl transferase; INR, international normalized ratio

## Discussion

This study shows that the gene expression profile in PBMCs of HIV/HCV-coinfected patients 24 weeks after achieving SVR (HIV/HCV-f) was significantly different from either HIV/HCV-coinfected patients before initiation of IFN-based therapy (HIV/HCV-b) or the control HIV group (HIV-mono). The highest differences were identified after HCV eradication compared to baseline (HIV/HCV-f vs. HIV/HCV-b). Unexpectedly some genes also remained overexpressed or suppressed after HCV clearance with respect to the HIV-monoinfected patients (HIV/HCV-f vs. HIV-mono). However, HIV/HCV-b showed only discrete expression differences from the HIV-mono, and no significantly enriched pathways were found.

### HIV/HCV-b vs. HIV-mono

Unexpectedly, when HIV/HCV-b and HIV-mono were compared, we found only six SDE genes and no biological pathways overrepresented, indicating that HCV infection had a moderate impact on PBMCs gene expression in HIV/HCV-coinfected patients. Kottilil et al. showed that although HCV mono-infection induces significant PBMC gene expression changes related to immunoregulatory and proinflammatory pathways, the differences between HIV/HCV-coinfected and HIV-monoinfected patients were small [42]. Our results also agree with previous findings showing that HCV infection leads to hyporesponsiveness of PBMCs [43, 44]. Furthermore, continuous stimulation of TLR2, TLR4, and TLR5 has been shown to lead to monocyte tolerance [45].

All the upregulated genes in the HIV/HCV-b group (*IL23A*, *FKBP15*, *CALR*, and *DDIT3*) are associated with HCV infection. IL23A is a crucial cytokine in the immune and inflammatory response that plays an essential role in HCV infection. Its increase may enhance the antiviral activity of IFN-based therapy by modulating the molecules expressed by Th17 cells in patients infected with HCV [46]. HCV promotes significant changes in the endoplasmic reticulum (ER) [47]. CALR is a chaperone that interacts with HCV to correctly fold their glycoproteins, avoiding the appearance of chronic stress in the ER when the folding capacity of cellular proteins is exceeded [48]. FKBP15 is a binding protein directly involved in regulating endosome trafficking, which HCV hijacks to produce viral particles [49]. Therefore, a higher FKBP15 expression may be related to the higher activation of the endosome-based secretory pathway for a higher virus production through exosome pathways. Besides, FKBP15 has been identified as mutated in HCV-infected cirrhotic liver and is associated with HCC [50]. DDIT3 is another gene related to the ER that is activated by the HCV core protein after promoting ER stress [51].

All the downregulated regulated genes (*TLR5* and *STXBP1*) have also been linked to HCV infection. STXBP1 is a protein crucial in vesicle docking and fusion. Its downregulation seems to be related to a lower HCV release [52] and a lower cytolytic T lymphocyte activity [53]. TLR5 recognizes explicitly bacterial flagellin. However, stimulation of TLR2 by bacterial products induces *TLR5* downregulation in human monocytes [45]. In this sense, chronic hepatitis C increases bacterial translocation to the blood, and downregulation of *TLR5* may be beneficial for the host in the context of persistent immune stimulation by reducing the immune response and the associated immunopathology.

Finally, *TLR5* was down-regulated by more than twofold in the HIV/HCV-b group, but its expression was normalized at the end of the study when HCV eradication reduces microbial translocation in HIV/HCV-coinfected patients [54]. The remaining five SDE genes (*IL23A*, *FKBP15*, *CALR*, *DDIT3,* and *STXBP1*) stayed deregulated at the end of follow-up, indicating that the ER-related and exosome pathways may remain altered after HCV elimination with IFN-based therapy. Therefore, of the six SDE genes, when HCV infection disappears, only the expression of TLR5 was normalized. However, the biological significance of this result is difficult to ascertain since the genes did not group in any biological pathway. Besides, the expression differences with respect to the HIV-mono group were small (FC<2).

### HIV/HCV-f vs. HIV/HCV-b groups

Gene expression in PBMCs from HIV/HCV-coinfected patients showed the deregulation of 58 genes after achieving SVR. These SDE genes represent two biological pathways, Epstein-Barr virus (EBV) infection and p53 signaling.

EBV establishes a latent infection in over 90% of human adults, alternating between episodes of latency and reactivation inside B-lymphocytes, which may lead to the malignant transformation of B cells. Thus, EBV has been linked to a broad spectrum of human malignancies [55]. Additionally, EBV coinfection may alter HIV-infected patients' immunological response, increasing inflammation and immune activation [55]. However, these SDE genes deregulated in the EBV pathway are also involved in many other immune pathways that may have been affected after HCV treatment.

On the one hand, *BID* and *HLA-A* were downregulated after HCV eradication. *BID* encodes a proapoptotic member of the Bcl-2 protein family, and its downregulation may indicate a decrease of apoptosis after HCV clearance with HCV therapy. Liver BID suppression improves inflammation and liver fibrosis in experimental NASH [56] and can protect from the development of HCC [57, 58]. *HLA-A* is a member of the major histocompatibility complex (MHC) from class Ia involved in antigen presentation to CD8^+^ T cells during an immune response [59]. A decrease of HLA-A expression may be related to a reduction of CD8^+^ T cell response after HCV clearance with HCV therapy. However, increased HLA-A expression levels are related to poor control of HIV through HLA-E induction and the inhibition of NK cells [60]. Therefore, the downregulation of *BID* and *HLA-A* genes could improve liver disease and immune response.

On the other hand, *CDKN1A*, *NFKB2*, *RELB,* and *PDIA3* genes were upregulated after HCV eradication. *CDKN1A* encodes p21, a known inhibitor of the cell cycle, linked to senescence arrest and regulated by p53. In the liver, p21 overexpression is related to the arrest of liver regeneration and the development of NAFLD, liver fibrosis, and cirrhosis [61]. However, p21 may have two contradictory functions related to HCC (tumor suppressor or promoter), depending on its subcellular localization [62]. Thus, its nuclear location is associated with a tumor suppression function. Besides, IFN may keep p21 in the nucleus, where it develops its anti-hepatocarcinogenesis role [62]. Therefore, p21 upregulation after IFN-treatment may be associated with a reduced risk of HCC compared to the baseline. P21 also has an antiviral function, and its upregulation blocks HIV replication in HIV-infected patients with elite control phenotype [63, 64]. *NFKB2* encodes the nuclear factor kappa-B (NF-κB) p100, a subunit of the NF-κB complex, which may function as a repressor or transcriptional activator, depending on its dimerization partner. As a transcriptional repressor, NF-κB2 may support the establishment of HIV latency, while as a transcriptional activator, it may facilitate HIV transcription [65]. NF-κB2 has an essential role in HCC pathogenesis [66] and NAFLD [67]. *RELB* also encodes a transcription factor that interacts with NF-κB2, and it may also facilitate HIV transcription [65, 68]. RelB expression is increased in chronic hepatitis C and promotes liver fibrosis [69, 70]. *PDIA3* encodes an isomerase enzyme that participates in the synthesis of glycoproteins such as HLA-I. It is a redox sensor by activating mTORC1, and it regulates cell growth, death, and signal transduction via STAT3 [71]. *PDIA3* deregulation is associated with diverse pathological conditions [71]. *PDIA3* overexpression can promote liver fibrosis [72] and HCC [73, 74]. Overall, the upregulation of *CDKN1A*, *NFKB2*, *RELB,* and *PDIA3* indicates a worse prognosis of liver disease and an impaired immune response against HIV.

We have also identified SDE genes related to the p53 signaling pathway. P53 is a tumor suppressor protein that restricts tumorigenesis by regulating cell cycle arrest, senescence, and apoptosis. The p53 signaling pathway has been previously associated with HCV infection as p53 function is disrupted by direct interaction with viral particles [75] or by the host immune response [76]. Thus, p53 function disruption contributes to hepatocellular carcinogenesis. Two genes of this pathway were downregulated (*BID* and *TP73*) and two upregulated (*CDKN1A* and *PMAIP1*). *BID* and *CDKN1A* are both involved in the pathway of EBV infection that has been previously discussed. *TP73* encodes p73, which is considered a p53-related transcription factor involved in cell cycle regulation and apoptosis. *TP73* is up-regulated in tumors of HCC patients [77], and lower p73 expression is related to higher survival in HCC patients [78]. *PMAIP1* encodes a pro-apoptotic protein of the Bcl-2 family that is regulated by p53. *PMAIP1* overexpression is linked to NASH and fibrosis histological criteria [79], which could indicate a worse prognosis of liver disease. However, in vitro studies have shown PMAIP1 plays a crucial role in hepatocarcinogenesis by limiting cancer cell survival [80] and an anti-tumoral function [81]. Therefore, *PMAIP1* upregulation after HCV eradication would be related to a reduced risk of HCC in these patients.

Finally, we found several 2-fold deregulated genes. HIV/HCV-f showed two highly downregulated genes (*IFI44* and *IFI44L*) and six highly upregulated genes (*CXCL2, PDCD6IP, ATP5B, IGSF9, RAB26,* and *CSRNP1*) compared to HIV/HCV-b. For the sake of brevity, we have discussed these genes in Additional file 6:Table S6.

### HIV/HCV-f vs. HIV-mono

The comparison of HIV/HCV-f vs. HIV-mono also revealed the upregulation of inflammatory and carcinogenic genes after HCV clearance. Specifically, we found 44 SDE genes and 31 significantly enriched pathways related to inflammation, cancer, complications associated with HIV/HCV coinfection, and viral and bacterial infections.

Overall, five upregulated SDE genes were overrepresented in most pathways (*JUN*, *NFKBIA*, *PIK3R2*, *CDC42*, and *STAT3*). *JUN* encodes a subunit of the AP-1 transcription factor that promotes inflammation and insulin resistance. *JUN* overexpression promotes liver fibrosis and correlates with progression from steatosis to NASH [82]. *NFKBIA* encodes an inhibitor of NF-κB. *NFKBIA* overexpression is related to steatosis development by induction of hepatocyte apoptosis and secretion of TNF-α and IL-1β by Kupffer cells [83]. Together with bacterial translocation, these mediators can activate hepatic stellate cells and promote liver fibrosis and establish the conditions for the development of HCC [84]. Moreover, *NFKBIA* overexpression may attenuate NF-κB signaling and enhance HIV-1 latency [85, 86]. *PIK3R2* encodes a negative regulatory protein of the PI3K pathway. Increased *PIK3R2* expression has been found in cancer, and it has been proposed as an oncogene [87]. *CDC42* encodes a Rho GTPase that regulates the cell cycle, and its overexpression is related to the development of fibrosis [88], NAFLD [89], and HCC [90]. *STAT3* encodes a transcription factor that regulates proliferation, cell survival, and the immune response. STAT3 signaling is related to liver inflammation, injury, steatosis, fibrosis, and HCC [91], and it is overexpressed in patients with NASH [92] and HCC [93].

The HIV/HCV-f group showed five SDE genes that were 2-fold upregulated with respect to the HIV-mono (*KLF6, HSPA5, JUN, PRRC2C,* and *PPP1R15A*). A brief discussion of these genes is provided in Additional file 6:Table S6.

### Consequences of HCV elimination by IFN-based therapy

Overall, our results revealed that HCV elimination after IFN-based treatment positively and negatively affects HIV/HCV-coinfected patients.

On the one hand, IFN-based therapy eliminates HCV infection, improving some aspects of inflammation, immune activation, and liver disease progression. Thus, we found an improvement at the end of follow-up in non-invasive markers of liver disease (liver stiffness, FIB-4, AST, ALT, and albumin) and a significant increase in CD4^+^ T-cells/mm^3^, improving the patient's health status. Besides, we have recently described in this cohort (GESIDA 3603b study) a decrease in some plasma and T-cell biomarkers related to inflammation and immune activation in HIV/HCV-coinfected patients after achieving SVR with IFN-based treatment. Still, most biomarkers in plasma and T-cell did not improve and were far from normalization compared to HIV-monoinfected patients [40]. Moreover, we explored the specific signatures of lymphocytes T CD4^+^ and CD8^+^, based on previous transcriptome profiling on these cells [94, 95], but none of these genes were identified as differentially expressed after HCV eradication.

On the other hand, HCV elimination with IFN-based therapy does not lead to the restoration of normal physiological conditions in some cases. As previously discussed, some SDE genes and enriched pathways are related to increased inflammation and the development of NASH, NAFLD, or HCC. This may be due to multiple factors, some of which are the following:

Firstly, IFN treatment was usually dispensed to patients in advanced stages of liver disease (F ≥2), where severe liver damage already exists and may not completely reverse after reaching SVR. Persistent histological inflammation, which may contribute to hepatic fibrosis and carcinogenesis, was detected several years after successful IFN-based therapy [96]. Besides, HCV elimination after DAAs therapy does not restore the epigenetic changes related to HCC development induced by HCV infection [97, 98], which indicates that the failure to normalize the liver injury after HCV elimination is not unique to IFN-based treatment. In our study, the follow-up time of HIV/HCV-coinfected patients was long enough (24 weeks after SVR) to observe an increase in plasma cholesterol levels at the end of follow-up. This finding is consistent with data recently published by Carrero *et al.* [99], which reports an increase in plasma cholesterol and cardiovascular risk in HIV/HCV-coinfected patients who achieved SVR with IFN-based therapy. However, we have not observed the development of other adverse outcomes, such as the development of NAFLD or HCC.

Secondly, it may be due to the immunomodulatory effects of IFN-based treatment. In this regard, our findings are in line with previous investigations. For example, IFN treatment induces a robust cytokine response mediated by STAT1, NF-κB and JNK signaling pathways in PBMCs of HCV-infected patients with SVR compared to non-responder patients [100]. An inflammatory response was also observed in PMBCs after successful IFN treatment of HCV infection [39]. Moreover, as previously commented, we have reported in this cohort (GESIDA 3603b study) that a large number of biomarkers did not improve after achieving SVR with IFN-based therapy, staying away from the values of the HIV control group [40].

Thirdly, another possible explanation resides in the elimination of HCV itself. Chronic hepatitis C profoundly affects the host immune response and other physiological processes [101]. Therefore, HCV elimination may induce undesired effects in the already severely affected immune system and liver physiology, particularly in the context of HIV coinfection.

### Limitations of the study

Our study has some limitations. Therefore, our conclusions should be interpreted with caution: (i) The number of patients per group was limited, which could prevent the detection of small differences between groups and increase the risk of false positives results. However, the GLMM approach properly accounts for the random effect in our model, limiting the false-positive rate and increasing the statistical power. (ii) Our study had a prospective observational design and may introduce biases. (iii) Another limitation of this study was the lack of other control groups that could give our findings more robustness. So, we lack an HCV monoinfected group of patients with similar liver disease characteristics to the HIV/HCV group. This control group would help to elucidate gene expression profiles related to the immune system after the HCV clearance with IFN treatment without HIV interaction. Nor were available a group of HCV/HIV-coinfected patients who failed the IFN treatment or HCV/HIV-coinfected patients receiving DAA treatment (IFN-free treatment) as controls. These control group options would have helped us to elucidate whether the differences observed in this study were due to the effect of HCV clearance or IFN treatment per se. iv) iv) This work is an association study and lacks mechanistic exploration or functional validation. Functional validation by quantitative PCR is not usually necessary when using a large number of biological replicates. Given the complex virus-host interactions, it is difficult to know which genetic changes are truly clinically important and contribute to clinical outcomes.

## Conclusions

HIV/HCV-coinfected patients who eradicated chronic hepatitis C with peg-IFN-α/ribavirin therapy showed profound gene expression changes in PBMCs, which seem to be related to inflammation and liver-related complications, such as NASH and HCC. Our findings may be influenced by the long-term impact of IFN therapy, although a rebound effect due to HCV elimination itself could not be ruled out. Prospective studies should be carried out to monitor the long-term normalization of gene expression profiles and clinical evolution in these patients who achieved successful SVR after IFN-based regimens.

## Supplementary Information

**Additional file 1: Table S1.** Bioinformatics pipeline to analyze raw sequences from RNA-seq of HIV/HCV-infected and HIV-monoinfected patients.

**Additional file 2: Table S2.** List of 4723 genes related to the immune system according to InnateDB, a public database of human immune genes (www.innatedb.com).

**Additional file 3: Table S3.** Summary of significant differentially expressed genes (FC ≥1.5; FDR ≤0.05) in peripheral blood mononuclear cells: A) HIV/HCV-b versus HIV-mono. B) HIV/HCV-coinfected at week 24 (HIV/HCV-f) after SVR versus at baseline (HIV/HCV-b). C) HIV/HCV-f versus HIV-mono.

**Additional file 4: Table S4**. Summary of significant KEGG pathways (FDR ≤0.05) in the HIV/HCV-f versus HIV/HCV-b comparison.

**Additional file 5: Table S5**. Summary of significant KEGG pathways (FDR ≤0.05) in the HIV/HCV-f versus HIV-mono comparison.

**Additional file 6: Table S6**. Summary of the most deregulated SDE genes (absolute FC ≥2 and FDR ≤0.05).

**Additional file 7: Appendix S1.** The GESIDA 3603b Cohort Study Group.

## Data Availability

The datasets used and analyzed during the current study are available from the corresponding authors upon reasoned request. HIV/HCV-coinfected patients' raw sequences data are publicly available at the ArrayExpress repository (EMBL-EBI; https://www.ebi.ac.uk/) under the accession number E-MTAB-8275. The raw RNA sequences of HIV-infected patients can also be accessed under the accession number E-MTAB-8249.

## References

[1] The Polaris Observatory HCV Collaborators (2017). Global prevalence and genotype distribution of hepatitis C virus infection in 2015: a modelling study. Lancet Gastroenterol Hepatol.

[2] Platt L, Easterbrook P, Gower E, McDonald B, Sabin K, McGowan C, Yanny I, Razavi H, Vickerman P (2016). Prevalence and burden of HCV co-infection in people living with HIV: a global systematic review and meta-analysis. Lancet Infect Dis.

[3] Vallet-Pichard A, Pol S (2006). Natural history and predictors of severity of chronic hepatitis C virus (HCV) and human immunodeficiency virus (HIV) co-infection. J Hepatol.

[4] Lo-Re V, Kallan MJ, Tate JP, Localio AR, Lim JK, Goetz MB, Klein MB, Rimland D, Rodriguez-Barradas MC, Butt AA (2014). Hepatic decompensation in antiretroviral-treated patients co-infected with HIV and hepatitis C virus compared with hepatitis C virus-monoinfected patients: a cohort study. Ann Intern Med.

[5] López-Diéguez M, Montes ML, Pascual-Pareja JF, Quereda C, Von Wichmann MA, Berenguer J, Tural C, Hernando A, González-García J, Serrano L (2011). The natural history of liver cirrhosis in HIV-hepatitis C virus-coinfected patients. AIDS.

[6] Macias J, Berenguer J, Japon MA, Giron JA, Rivero A, Lopez-Cortes LF, Moreno A, Gonzalez-Serrano M, Iribarren JA, Ortega E (2009). Fast fibrosis progression between repeated liver biopsies in patients coinfected with human immunodeficiency virus/hepatitis C virus. Hepatology.

[7] Ingiliz P, Rockstroh JK (2015). Natural history of liver disease and effect of hepatitis C virus on HIV disease progression. Curr Opin HIV AIDS.

[8] Naggie S (2017). Hepatitis C virus, inflammation, and cellular aging: turning back time. Top Antivir Med.

[9] Shin EC, Sung PS, Park SH (2016). Immune responses and immunopathology in acute and chronic viral hepatitis. Nat Rev Immunol.

[10] Pandiyan P, Younes SA, Ribeiro SP, Talla A, McDonald D, Bhaskaran N, Levine AD, Weinberg A, Sekaly RP (2016). Mucosal Regulatory T Cells and T Helper 17 Cells in HIV-Associated Immune Activation. Front Immunol.

[11] Miles B, Miller SM, Connick E (2016). CD4 t follicular helper and regulatory cell dynamics and function in HIV infection. Front Immunol.

[12] De Biasi S, Bianchini E, Nasi M, Digaetano M, Gibellini L, Carnevale G, Borghi V, Guaraldi G, Pinti M, Mussini C (2016). Th1 and Th17 proinflammatory profile characterizes invariant natural killer T cells in virologically suppressed HIV+ patients with low CD4+/CD8+ ratio. AIDS.

[13] Fernandes JR, Berthoud TK, Kumar A, Angel JB (2017). IL-23 signaling in Th17 cells is inhibited by HIV infection and is not restored by HAART: Implications for persistent immune activation. PLoS ONE.

[14] DaFonseca S, Niessl J, Pouvreau S, Wacleche VS, Gosselin A, Cleret-Buhot A, Bernard N, Tremblay C, Jenabian MA, Routy JP (2015). Impaired Th17 polarization of phenotypically naive CD4(+) T-cells during chronic HIV-1 infection and potential restoration with early ART. Retrovirology.

[15] Mahnke YD, Fletez-Brant K, Sereti I, Roederer M (2016). Reconstitution of Peripheral T Cells by Tissue-Derived CCR4+ central memory cells following HIV-1 antiretroviral therapy. Pathog Immun.

[16] de Paula HHS, Ferreira ACG, Caetano DG, Delatorre E, Teixeira SLM, Coelho LE, Joao EG, de Andrade MM, Cardoso SW, Grinsztejn B (2018). Reduction of inflammation and T cell activation after 6 months of cART initiation during acute, but not in early chronic HIV-1 infection. Retrovirology.

[17] Merlini E, Tincati C, Biasin M, Saulle I, Cazzaniga FA, d'Arminio Monforte A, Cappione AJ, Snyder-Cappione J, Clerici M, Marchetti GC (2016). Stimulation of PBMC and monocyte-derived macrophages via toll-like receptor activates innate immune pathways in HIV-infected patients on virally suppressive combination antiretroviral therapy. Front Immunol.

[18] Mudd JC, Brenchley JM (2016). Gut mucosal barrier dysfunction, microbial dysbiosis, and their role in HIV-1 disease progression. J Infect Dis.

[19] Tincati C, Merlini E, Braidotti P, Ancona G, Savi F, Tosi D, Borghi E, Callegari ML, Mangiavillano B, Barassi A (2016). Impaired gut junctional complexes feature late-treated individuals with suboptimal CD4+ T-cell recovery upon virologically suppressive combination antiretroviral therapy. AIDS.

[20] Hunt PW, Lee SA, Siedner MJ (2016). Immunologic Biomarkers, Morbidity, and Mortality in Treated HIV Infection. J Infect Dis.

[21] Hart BB, Nordell AD, Okulicz JF, Palfreeman A, Horban A, Kedem E, Neuhaus J, Jacobs DR, Duprez DA, Neaton JD (2018). Inflammation-related morbidity and mortality among hiv-positive adults: how extensive is it?. J Acquir Immune Defic Syndr.

[22] Liang TJ, Ghany MG (2014). Therapy of hepatitis C–back to the future. N Engl J Med.

[23] Panel A-IHG: Hepatitis C Guidance 2018 Update: AASLD-IDSA recommendations for testing, managing, and treating hepatitis C Virus Infection. *Clin Infect Dis* 2018, 67(10):1477–1492. 10.1093/cid/ciy585 PMC7190892 30215672

[24] European Association for the Study of the Liver (2018). Electronic address eee, European Association for the Study of the L: EASL Recommendations on Treatment of Hepatitis C 2018. J Hepatol.

[25] Berenguer J, Gil-Martin A, Jarrin I, Moreno A, Dominguez L, Montes M, Aldamiz-Echevarria T, Tellez MJ, Santos I, Benitez L (2018). All-oral direct-acting antiviral therapy against hepatitis C virus (HCV) in human immunodeficiency virus/HCV-coinfected subjects in real-world practice: Madrid coinfection registry findings. Hepatology.

[26] Berenguer J, Rodriguez E, Miralles P, Von Wichmann MA, Lopez-Aldeguer J, Mallolas J, Galindo MJ, Van Den Eynde E, Tellez MJ, Quereda C (2012). Sustained virological response to interferon plus ribavirin reduces non-liver-related mortality in patients coinfected with HIV and Hepatitis C virus. Clin Infect Dis.

[27] Berenguer J, Rodriguez-Castellano E, Carrero A, Von Wichmann MA, Montero M, Galindo MJ, Mallolas J, Crespo M, Tellez MJ, Quereda C (2017). Eradication of hepatitis C virus and non-liver-related non-acquired immune deficiency syndrome-related events in human immunodeficiency virus/hepatitis C virus coinfection. Hepatology.

[28] Liu Z, Wei X, Chen T, Huang C, Liu H, Wang Y (2017). Characterization of fibrosis changes in chronic hepatitis C patients after virological cure: a systematic review with meta-analysis. J Gastroenterol Hepatol.

[29] Labarga P, Fernandez-Montero JV, de Mendoza C, Barreiro P, Pinilla J, Soriano V (2015). Liver fibrosis progression despite HCV cure with antiviral therapy in HIV-HCV-coinfected patients. Antivir Ther.

[30] Mira JA, Neukam K, Lopez-Cortes LF, Rivero-Juarez A, Tellez F, Giron-Gonzalez JA, de los Santos-Gil I, Ojeda-Burgos G, Merino D, Rios-Villegas MJ *et al*: Efficacy of and risk of bleeding during pegylated interferon plus ribavirin treatment in HIV/HCV-coinfected patients with pretreatment thrombocytopenia. *Eur J Clin Microbiol Infect Dis* 2015; 34(9):1879–1884. 10.1007/s10096-015-2426-6 26115631

[31] Salmon-Ceron D, Nahon P, Layese R, Bourcier V, Sogni P, Bani-Sadr F, Audureau E, Merchadou L, Dabis F, Wittkop L *et al*: Human Immunodeficiency Virus/Hepatitis C Virus (HCV) Co-infected Patients With Cirrhosis Are No Longer at Higher Risk for Hepatocellular Carcinoma or End-Stage Liver Disease as Compared to HCV Mono-infected Patients. *Hepatology* 2018. 10.1002/hep.30400 30569448

[32] Allaire M, Nahon P, Layese R, Bourcier V, Cagnot C, Marcellin P, Guyader D, Pol S, Larrey D, De Ledinghen V (2018). Extrahepatic cancers are the leading cause of death in patients achieving hepatitis B virus control or hepatitis C virus eradication. Hepatology.

[33] European Association for Study of L (2014). EASL Clinical Practice Guidelines: management of hepatitis C virus infection. J Hepatol.

[34] Rosenberg BR, Freije CA, Imanaka N, Chen ST, Eitson JL, Caron R, Uhl SA, Zeremski M, Talal A, Jacobson IM (2018). Genetic Variation at IFNL4 influences extrahepatic interferon-stimulated gene expression in chronic HCV Patients. J Infect Dis.

[35] Rosenberg BR, Depla M, Freije CA, Gaucher D, Mazouz S, Boisvert M, Bedard N, Bruneau J, Rice CM, Shoukry NH (2018). Longitudinal transcriptomic characterization of the immune response to acute hepatitis C virus infection in patients with spontaneous viral clearance. PLoS Pathog.

[36] Lu MY, Huang CI, Hsieh MY, Hsieh TJ, Hsi E, Tsai PC, Tsai YS, Lin CC, Hsieh MH, Liang PC (2016). Dynamics of PBMC gene expression in hepatitis C virus genotype 1-infected patients during combined peginterferon/ribavirin therapy. Oncotarget.

[37] Carlton-Smith C, Holmes JA, Naggie S, Lidofsky A, Lauer GM, Kim AY, Chung RT (2018). of the AAsg: IFN-free therapy is associated with restoration of type I IFN response in HIV-1 patients with acute HCV infection who achieve SVR. J Viral Hepat.

[38] Alao H, Cam M, Keembiyehetty C, Zhang F, Serti E, Suarez D, Park H, Fourie NH, Wright EC, Henderson WA (2018). Baseline intrahepatic and peripheral innate immunity are associated with hepatitis C virus clearance during direct-acting antiviral therapy. Hepatology.

[39] Waldron PR, Holodniy M (2015). Peripheral blood mononuclear cell gene expression remains broadly altered years after successful interferon-based hepatitis C virus treatment. J Immunol Res.

[40] Garcia-Broncano P, Medrano LM, Berenguer J, Brochado-Kith O, Gonzalez-Garcia J, Jimenez-Sousa MA, Quereda C, Sanz J, Tellez MJ, Diaz L (2020). Mild profile improvement of immune biomarkers in HIV/HCV-coinfected patients who removed hepatitis C after HCV treatment: a prospective study. J Infect.

[41] Hart SN, Therneau TM, Zhang Y, Poland GA, Kocher JP (2013). Calculating sample size estimates for RNA sequencing data. J Comput Biol.

[42] Kottilil S, Yan MY, Reitano KN, Zhang X, Lempicki R, Roby G, Daucher M, Yang J, Cortez KJ, Ghany M (2009). Human immunodeficiency virus and hepatitis C infections induce distinct immunologic imprints in peripheral mononuclear cells. Hepatology.

[43] Roque-Cuellar MC, Sanchez B, Garcia-Lozano JR, Praena-Fernandez JM, Marquez-Galan JL, Nunez-Roldan A, Aguilar-Reina J (2014). Hepatitis C virus-specific cellular immune responses in sustained virological responders with viral persistence in peripheral blood mononuclear cells. Liver Int.

[44] Chung H, Watanabe T, Kudo M, Chiba T (2011). Correlation between hyporesponsiveness to Toll-like receptor ligands and liver dysfunction in patients with chronic hepatitis C virus infection. J Viral Hepat.

[45] Cabral ES, Gelderblom H, Hornung RL, Munson PJ, Martin R, Marques AR (2006). Borrelia burgdorferi lipoprotein-mediated TLR2 stimulation causes the down-regulation of TLR5 in human monocytes. J Infect Dis.

[46] Meng P, Zhao S, Niu X, Fu N, Su S, Wang R, Zhang Y, Qiao L, Nan Y (2016). Involvement of the Interleukin-23/Interleukin-17 axis in chronic hepatitis C virus infection and its treatment responses. Int J Mol Sci.

[47] Ravindran MS, Bagchi P, Cunningham CN, Tsai B (2016). Opportunistic intruders: how viruses orchestrate ER functions to infect cells. Nat Rev Microbiol.

[48] Choukhi A, Ung S, Wychowski C, Dubuisson J (1998). Involvement of endoplasmic reticulum chaperones in the folding of hepatitis C virus glycoproteins. J Virol.

[49] Lai CK, Jeng KS, Machida K, Lai MM (2010). Hepatitis C virus egress and release depend on endosomal trafficking of core protein. J Virol.

[50] Ikeda A, Shimizu T, Matsumoto Y, Fujii Y, Eso Y, Inuzuka T, Mizuguchi A, Shimizu K, Hatano E, Uemoto S (2014). Leptin receptor somatic mutations are frequent in HCV-infected cirrhotic liver and associated with hepatocellular carcinoma. Gastroenterology.

[51] Wang J, Kang R, Huang H, Xi X, Wang B, Wang J, Zhao Z (2014). Hepatitis C virus core protein activates autophagy through EIF2AK3 and ATF6 UPR pathway-mediated MAP1LC3B and ATG12 expression. Autophagy.

[52] Ren H, Elgner F, Himmelsbach K, Akhras S, Jiang B, Medvedev R, Ploen D, Hildt E (2017). Identification of syntaxin 4 as an essential factor for the hepatitis C virus life cycle. Eur J Cell Biol.

[53] Phatarpekar PV, Billadeau DD (2020). Molecular regulation of the plasma membrane-proximal cellular steps involved in NK cell cytolytic function. J Cell Sci.

[54] Lopez-Cortes LF, Trujillo-Rodriguez M, Baez-Palomo A, Benmarzouk-Hidalgo OJ, Dominguez-Molina B, Milanes-Guisado Y, Espinosa N, Viciana P, Gutierrez-Valencia A (2018). Eradication of Hepatitis C Virus (HCV) Reduces Immune Activation, Microbial Translocation, and the HIV DNA Level in HIV/HCV-Coinfected Patients. J Infect Dis.

[55] Shindiapina P, Ahmed EH, Mozhenkova A, Abebe T, Baiocchi RA (2020). Immunology of EBV-Related Lymphoproliferative Disease in HIV-Positive Individuals. Front Oncol.

[56] Eguchi A (2016). De Mollerat Du Jeu X, Johnson CD, Nektaria A, Feldstein AE: Liver Bid suppression for treatment of fibrosis associated with non-alcoholic steatohepatitis. J Hepatol.

[57] Bao L, Zhang M, Han S, Zhan Y, Guo W, Teng F, Liu F, Guo M, Zhang L, Ding G (2018). MicroRNA-500a promotes the progression of hepatocellular carcinoma by post-transcriptionally targeting BID. Cell Physiol Biochem.

[58] Wree A, Johnson CD, Font-Burgada J, Eguchi A, Povero D, Karin M, Feldstein AE (2015). Hepatocyte-specific Bid depletion reduces tumor development by suppressing inflammation-related compensatory proliferation. Cell Death Differ.

[59] Crux NB, Elahi S (2017). Human Leukocyte Antigen (HLA) and immune regulation: how do classical and non-classical hla alleles modulate immune response to human immunodeficiency virus and hepatitis C virus infections?. Front Immunol.

[60] Ramsuran V, Naranbhai V, Horowitz A, Qi Y, Martin MP, Yuki Y, Gao X, Walker-Sperling V, Del Prete GQ, Schneider DK (2018). Elevated HLA-A expression impairs HIV control through inhibition of NKG2A-expressing cells. Science.

[61] Huda N, Liu G, Hong H, Yan S, Khambu B, Yin XM (2019). Hepatic senescence, the good and the bad. World J Gastroenterol.

[62] Ohkoshi S, Yano M, Matsuda Y (2015). Oncogenic role of p21 in hepatocarcinogenesis suggests a new treatment strategy. World J Gastroenterol.

[63] Diez-Fuertes F, De La Torre-Tarazona HE, Calonge E, Pernas M, Alonso-Socas MDM, Capa L, Garcia-Perez J, Sakuntabhai A, Alcami J (2019). Transcriptome sequencing of peripheral blood mononuclear cells from elite controller-long term non progressors. Sci Rep.

[64] Leng J, Ho HP, Buzon MJ, Pereyra F, Walker BD, Yu XG, Chang EJ, Lichterfeld M (2014). A cell-intrinsic inhibitor of HIV-1 reverse transcription in CD4(+) T cells from elite controllers. Cell Host Microbe.

[65] Jiang G, Dandekar S (2015). Targeting NF-kappaB signaling with protein kinase C agonists as an emerging strategy for combating HIV latency. AIDS Res Hum Retroviruses.

[66] O'Neil BH, Buzkova P, Farrah H, Kashatus D, Sanoff H, Goldberg RM, Baldwin AS, Funkhouser WK (2007). Expression of nuclear factor-kappaB family proteins in hepatocellular carcinomas. Oncology.

[67] Sookoian S, Pirola CJ (2013). Systems biology elucidates common pathogenic mechanisms between nonalcoholic and alcoholic-fatty liver disease. PLoS ONE.

[68] Wang M, Yang W, Chen Y, Wang J, Tan J, Qiao W (2018). Cellular RelB interacts with the transactivator Tat and enhance HIV-1 expression. Retrovirology.

[69] Zhou D, Huang W, Wei J, Zhang J, Liu Z, Ji R, Ge S, Xiao M, Fan Y, Lu C (2020). RelB promotes liver fibrosis via inducing the release of injury-associated inflammatory cytokines. J Cell Mol Med.

[70] Elssner C, Goeppert B, Longerich T, Scherr AL, Stindt J, Nanduri LK, Rupp C, Kather JN, Schmitt N, Kautz N (2019). Nuclear translocation of RELB is increased in diseased human liver and promotes ductular reaction and biliary fibrosis in mice. Gastroenterology.

[71] Hettinghouse A, Liu R, Liu CJ (2018). Multifunctional molecule ERp57: From cancer to neurodegenerative diseases. Pharmacol Ther.

[72] Zhao Q, Feng Y, Jia X, Yin L, Zheng Y, Ouyang D, Zhou H, Zhang L (2014). Proteome analysis of hepatic non-parenchymal cells of immune liver fibrosis rats. Sci China Life Sci.

[73] Kondo R, Ishino K, Wada R, Takata H, Peng WX, Kudo M, Kure S, Kaneya Y, Taniai N, Yoshida H (2019). Downregulation of protein disulfideisomerase A3 expression inhibits cell proliferation and induces apoptosis through STAT3 signaling in hepatocellular carcinoma. Int J Oncol.

[74] Ko E, Kim JS, Ju S, Seo HW, Chang Y, Kang JA, Park SG, Jung G (2018). Oxidatively modified protein-disulfide isomerase-associated 3 promotes dyskerin pseudouridine synthase 1-mediated malignancy and survival of hepatocellular carcinoma cells. Hepatology.

[75] Cheng D, Zhao L, Zhang L, Jiang Y, Tian Y, Xiao X, Gong G (2013). p53 controls hepatitis C virus non-structural protein 5A-mediated downregulation of GADD45α expression via the NF-κB and PI3K-Akt pathways. J Gen Virol.

[76] Mitchell JK, Midkiff BR, Israelow B, Evans MJ, Lanford RE, Walker CM, Lemon SM, McGivern DR (2017). Hepatitis C Virus Indirectly Disrupts DNA Damage-Induced p53 Responses by Activating Protein Kinase R. mBio.

[77] Jin Y, Lee WY, Toh ST, Tennakoon C, Toh HC, Chow PK, Chung AY, Chong SS, Ooi LL, Sung WK (2019). Comprehensive analysis of transcriptome profiles in hepatocellular carcinoma. J Transl Med.

[78] Tannapfel A, Wasner M, Krause K, Geissler F, Katalinic A, Hauss J, Mossner J, Engeland K, Wittekind C (1999). Expression of p73 and its relation to histopathology and prognosis in hepatocellular carcinoma. J Natl Cancer Inst.

[79] Bechmann LP, Gieseler RK, Sowa JP, Kahraman A, Erhard J, Wedemeyer I, Emons B, Jochum C, Feldkamp T, Gerken G (2010). Apoptosis is associated with CD36/fatty acid translocase upregulation in non-alcoholic steatohepatitis. Liver Int.

[80] Chang SC, Choo WQ, Toh HC, Ding JL (2015). SAG-UPS attenuates proapoptotic SARM and Noxa to confer survival advantage to early hepatocellular carcinoma. Cell Death Discov.

[81] Zhu H, Yang W, He LJ, Ding WJ, Zheng L, Liao SD, Huang P, Lu W, He QJ, Yang B (2012). Upregulating Noxa by ER stress, celastrol exerts synergistic anti-cancer activity in combination with ABT-737 in human hepatocellular carcinoma cells. PLoS ONE.

[82] Schulien I, Hockenjos B, Schmitt-Graeff A, Perdekamp MG, Follo M, Thimme R, Hasselblatt P (2019). The transcription factor c-Jun/AP-1 promotes liver fibrosis during non-alcoholic steatohepatitis by regulating Osteopontin expression. Cell Death Differ.

[83] DeAngelis RA, Markiewski MM, Taub R, Lambris JD (2005). A high-fat diet impairs liver regeneration in C57BL/6 mice through overexpression of the NF-kappaB inhibitor IkappaBalpha. Hepatology.

[84] Luedde T, Schwabe RF (2011). NF-kappaB in the liver–linking injury, fibrosis and hepatocellular carcinoma. Nat Rev Gastroenterol Hepatol.

[85] Taura M, Kudo E, Kariya R, Goto H, Matsuda K, Hattori S, Vaeteewoottacharn K, McDonald F, Suico MA, Shuto T (2015). COMMD1/Murr1 reinforces HIV-1 latent infection through IkappaB-alpha stabilization. J Virol.

[86] Kogan M, Deshmane S, Sawaya BE, Gracely EJ, Khalili K, Rappaport J (2013). Inhibition of NF-kappaB activity by HIV-1 Vpr is dependent on Vpr binding protein. J Cell Physiol.

[87] Vallejo-Diaz J, Chagoyen M, Olazabal-Moran M, Gonzalez-Garcia A, Carrera AC (2019). The opposing roles of PIK3R1/p85alpha and PIK3R2/p85beta in cancer. Trends Cancer.

[88] Muhanna N, Doron S, Wald O, Horani A, Eid A, Pappo O, Friedman SL, Safadi R (2008). Activation of hepatic stellate cells after phagocytosis of lymphocytes: a novel pathway of fibrogenesis. Hepatology.

[89] Wang R, Wang X, Zhuang L (2016). Gene expression profiling reveals key genes and pathways related to the development of non-alcoholic fatty liver disease. Ann Hepatol.

[90] Grise F, Bidaud A, Moreau V (2009). Rho GTPases in hepatocellular carcinoma. Biochim Biophys Acta.

[91] Wang H, Lafdil F, Kong X, Gao B (2011). Signal transducer and activator of transcription 3 in liver diseases: a novel therapeutic target. Int J Biol Sci.

[92] Handa P, Vemulakonda AL, Maliken BD, Morgan-Stevenson V, Nelson JE, Dhillon BK, Hennessey KA, Gupta R, Yeh MM, Kowdley KV (2017). Differences in hepatic expression of iron, inflammation and stress-related genes in patients with nonalcoholic steatohepatitis. Ann Hepatol.

[93] Lee C, Cheung ST (2019). STAT3: An Emerging Therapeutic Target for Hepatocellular Carcinoma. Cancers (Basel).

[94] Lee MS, Hanspers K, Barker CS, Korn AP, McCune JM (2004). Gene expression profiles during human CD4+ T cell differentiation. Int Immunol.

[95] Helgeland H, Gabrielsen I, Akselsen H, Sundaram AYM, Flam ST, Lie BA (2020). Transcriptome profiling of human thymic CD4+ and CD8+ T cells compared to primary peripheral T cells. BMC Genomics.

[96] Nirei K, Kanda T, Nakamura H, Matsuoka S, Takayama T, Sugitani M, Moriyama M (2018). Persistent hepatic inflammation plays a role in hepatocellular carcinoma after sustained virological response in patients with HCV Infection. Int J Med Sci.

[97] Perez S, Kaspi A, Domovitz T, Davidovich A, Lavi-Itzkovitz A, Meirson T, Alison Holmes J, Dai CY, Huang CF, Chung RT (2019). Hepatitis C virus leaves an epigenetic signature post cure of infection by direct-acting antivirals. PLoS Genet.

[98] Hamdane N, Juhling F, Crouchet E, El Saghire H, Thumann C, Oudot MA, Bandiera S, Saviano A, Ponsolles C, Roca Suarez AA (2019). HCV-induced epigenetic changes associated with liver cancer risk persist after sustained virologic response. Gastroenterology.

[99] Carrero A, Berenguer J, Hontanon V, Navarro J, Hernandez-Quero J, Galindo MJ, Quereda C, Santos I, Tellez MJ, Ortega E (2020). Effects of Eradication of HCV on cardiovascular risk and preclinical atherosclerosis in HIV/HCV-coinfected patients. J Acquir Immune Defic Syndr.

[100] Wandrer F, Falk CS, John K, Skawran B, Manns MP, Schulze-Osthoff K, Bantel H (2016). Interferon-mediated cytokine induction determines sustained virus control in chronic hepatitis C virus infection. J Infect Dis.

[101] Irshad M, Gupta P, Irshad K (2019). Immunopathogenesis of liver injury during hepatitis C virus infection. Viral Immunol.

