# Supplementary material for: HCV eradication with IFN-based therapy does not completely restore gene expression in PBMCs from HIV/HCV-coinfected patients
Source: J Biomed Sci. 2021 Mar 30;28:23. doi: 10.1186/s12929-021-00718-6 (PMC8010945; doi:10.1186/s12929-021-00718-6)
Supplement: Supplementary file 1 — Additional file 1: Table S1. Bioinformatics pipeline to analyze raw sequences from RNA-seq of HIV/HCV-infected and HIV-monoinfected patients. [file 12929_2021_718_MOESM1_ESM.docx]

**Supplementary Table 1.** Bioinformatics pipeline to analyze raw sequences from RNA-seq of HIV/HCV-infected and HIV-monoinfected patients.

| *1. Filtering step*  Settings employed to eliminate adapters and low quality reads.  - Software:  Trimmomatic  - Version:  0.33  - Code:  java -jar PATH-TO-TRIMMOMATIC/trimmomatic-0.33.jar SE -threads 10 -phred33 PATH-TO-SAMPLES/”sample”.fastq.gz “sample”/”sample”_filtered.fastq ILLUMINACLIP:all_PE.fa:2:30:10 SLIDINGWINDOW:4:15 MINLEN:50 |
| --- |
| *2. Mapping Step*  Settings employed for mapping the filtered counts.  - Software:  Tophat2  - Version:  2.0.14  - Human genome:  GRCh38  - Code:  mkdir -p “sample”; qsub -V -b y -j y -cwd -N TOPHATALIGNMENT -q all.q -pe openmp 10 tophat2 -p 10 -o 026C -G ../../REFERENCES/GRCh38_refseq.gtf --transcriptome-index ../../REFERENCES/ ../../REFERENCES/hg38.fullAnalysisSet.fa ../02-preprocessing/”sample”/”sample”_filtered.fastq.gz |
| *3. Count step*  Settings employed to obtain the number of counts per gene and sample  - Software:  HTSeq  - Version:  0.6.1  - Code:  #!/bin/bash  #$ -V  #$ -b y  #$ -j y  #$ -cwd  #$ -N HTSEQCOUNT  #$ -q all.q  #$ -t 1-100  set -e  set -x  infile=../samples_id.txt  in=$(awk "NR==$SGE_TASK_ID" $infile)  mkdir -p $in  htseq-count -f bam ../04-tophat/$in/"$in"_accepted_hits.bam ../../REFERENCES/GRCh38_refseq.gtf > $in/"$in"_htseqCount.txt |
