# Supplementary material for: HCV eradication with IFN-based therapy does not completely restore gene expression in PBMCs from HIV/HCV-coinfected patients
Source: J Biomed Sci. 2021 Mar 30;28:23. doi: 10.1186/s12929-021-00718-6 (PMC8010945; doi:10.1186/s12929-021-00718-6)
Supplement: Supplementary file 2 — Additional file 2: Table S2. List of 4723 genes related to the immune system according to InnateDB, a public database of human immune genes (www.innatedb.com). [file 12929_2021_718_MOESM2_ESM.docx]

**Supplementary Table 2.** List of 4,723 genes related to the immune system according to InnateDB, a public database of human immune genes ([www.innatedb.com](http://www.innatedb.com/)).

| **ensembl** | **name** | **fullname** | **chromName** | **band** |
| --- | --- | --- | --- | --- |
| ENSG00000099715 | PCDH11Y | protocadherin 11 Y-linked | Y | p11.2 |
| ENSG00000092377 | TBL1Y | transducin (beta)-like 1, Y-linked | Y | p11.2 |
| ENSG00000114374 | USP9Y | ubiquitin specific peptidase 9, Y-linked | Y | q11.221 |
| ENSG00000165246 | NLGN4Y | neuroligin 4, Y-linked | Y | q11.221 |
| ENSG00000101557 | USP14 | ubiquitin specific peptidase 14 (tRNA-guanine transglycosylase) | 18 | p11.32 |
| ENSG00000079134 | THOC1 | THO complex 1 | 18 | p11.32 |
| ENSG00000158270 | COLEC12 | collectin sub-family member 12 | 18 | p11.32 |
| ENSG00000141433 | ADCYAP1 | adenylate cyclase activating polypeptide 1 (pituitary) | 18 | p11.32 |
| ENSG00000132205 | EMILIN2 | elastin microfibril interfacer 2 | 18 | p11.32 |
| ENSG00000101680 | LAMA1 | laminin, alpha 1 | 18 | p11.31 |
| ENSG00000173482 | PTPRM | protein tyrosine phosphatase, receptor type, M | 18 | p11.23 |
| ENSG00000017797 | RALBP1 | ralA binding protein 1 | 18 | p11.22 |
| ENSG00000154639 | CXADR | coxsackie virus and adenovirus receptor | 21 | q21.1 |
| ENSG00000172967 | XKR3 | XK, Kell blood group complex subunit-related family, member 3 | 22 | q11.1 |
| ENSG00000177663 | IL17RA | interleukin 17 receptor A | 22 | q11.1 |
| ENSG00000093072 | CECR1 | cat eye syndrome chromosome region, candidate 1 | 22 | q11.1 |
| ENSG00000099954 | CECR2 | cat eye syndrome chromosome region, candidate 2 | 22 | q11.1 |
| ENSG00000099968 | BCL2L13 | BCL2-like 13 (apoptosis facilitator) | 22 | q11.21 |
| ENSG00000154654 | NCAM2 | neural cell adhesion molecule 2 | 21 | q21.1 |
| ENSG00000015475 | BID | BH3 interacting domain death agonist | 22 | q11.21 |
| ENSG00000154721 | JAM2 | junctional adhesion molecule 2 | 21 | q21.3 |
| ENSG00000176194 | CIDEA | cell death-inducing DFFA-like effector a | 18 | p11.21 |
| ENSG00000142192 | APP | amyloid beta (A4) precursor protein | 21 | q21.3 |
| ENSG00000128789 | PSMG2 | proteasome (prosome, macropain) assembly chaperone 2 | 18 | p11.21 |
| ENSG00000183628 | DGCR6 | DiGeorge syndrome critical region gene 6 | 22 | q11.21 |
| ENSG00000154734 | ADAMTS1 | ADAM metallopeptidase with thrombospondin type 1 motif, 1 | 21 | q21.3 |
| ENSG00000100033 | PRODH | proline dehydrogenase (oxidase) 1 | 22 | q11.21 |
| ENSG00000154736 | ADAMTS5 | ADAM metallopeptidase with thrombospondin type 1 motif, 5 | 21 | q21.3 |
| ENSG00000070413 | DGCR2 | DiGeorge syndrome critical region gene 2 | 22 | q11.21 |
| ENSG00000198862 | LTN1 | listerin E3 ubiquitin protein ligase 1 | 21 | q21.3 |
| ENSG00000100056 | DGCR14 | DiGeorge syndrome critical region gene 14 | 22 | q11.21 |
| ENSG00000070371 | CLTCL1 | clathrin, heavy chain-like 1 | 22 | q11.21 |
| ENSG00000067900 | ROCK1 | Rho-associated, coiled-coil containing protein kinase 1 | 18 | q11.1 |
| ENSG00000184113 | CLDN5 | claudin 5 | 22 | q11.21 |
| ENSG00000184702 | SEPT5 | septin 5 | 22 | q11.21 |
| ENSG00000156282 | CLDN17 | claudin 17 | 21 | q21.3 |
| ENSG00000184058 | TBX1 | T-box 1 | 22 | q11.21 |
| ENSG00000156284 | CLDN8 | claudin 8 | 21 | q22.11 |
| ENSG00000101752 | MIB1 | mindbomb E3 ubiquitin protein ligase 1 | 18 | q11.2 |
| ENSG00000141448 | GATA6 | GATA binding protein 6 | 18 | q11.2 |
| ENSG00000093010 | COMT | catechol-O-methyltransferase | 22 | q11.21 |
| ENSG00000099889 | ARVCF | armadillo repeat gene deleted in velocardiofacial syndrome | 22 | q11.21 |
| ENSG00000128191 | DGCR8 | DiGeorge syndrome critical region gene 8 | 22 | q11.21 |
| ENSG00000156299 | TIAM1 | T-cell lymphoma invasion and metastasis 1 | 21 | q22.11 |
| ENSG00000141458 | NPC1 | Niemann-Pick disease, type C1 | 18 | q11.2 |
| ENSG00000142168 | SOD1 | superoxide dismutase 1, soluble | 21 | q22.11 |
| ENSG00000053747 | LAMA3 | laminin, alpha 3 | 18 | q11.2 |
| ENSG00000170262 | MRAP | melanocortin 2 receptor accessory protein | 21 | q22.11 |
| ENSG00000128185 | DGCR6L | DiGeorge syndrome critical region gene 6-like | 22 | q11.21 |
| ENSG00000134489 | HRH4 | histamine receptor H4 | 18 | q11.2 |
| ENSG00000159082 | SYNJ1 | synaptojanin 1 | 21 | q22.11 |
| ENSG00000141380 | SS18 | synovial sarcoma translocation, chromosome 18 | 18 | q11.2 |
| ENSG00000154611 | PSMA8 | proteasome (prosome, macropain) subunit, alpha type, 8 | 18 | q11.2 |
| ENSG00000099937 | SERPIND1 | serpin peptidase inhibitor, clade D (heparin cofactor), member 1 | 22 | q11.21 |
| ENSG00000099940 | SNAP29 | synaptosomal-associated protein, 29kDa | 22 | q11.21 |
| ENSG00000099942 | CRKL | v-crk sarcoma virus CT10 oncogene homolog (avian)-like | 22 | q11.21 |
| ENSG00000171885 | AQP4 | aquaporin 4 | 18 | q11.2 |
| ENSG00000183773 | AIFM3 | apoptosis-inducing factor, mitochondrion-associated, 3 | 22 | q11.21 |
| ENSG00000170558 | CDH2 | cadherin 2, type 1, N-cadherin (neuronal) | 18 | q12.1 |
| ENSG00000134762 | DSC3 | desmocollin 3 | 18 | q12.1 |
| ENSG00000134755 | DSC2 | desmocollin 2 | 18 | q12.1 |
| ENSG00000159110 | IFNAR2 | interferon (alpha, beta and omega) receptor 2 | 21 | q22.11 |
| ENSG00000134765 | DSC1 | desmocollin 1 | 18 | q12.1 |
| ENSG00000134760 | DSG1 | desmoglein 1 | 18 | q12.1 |
| ENSG00000175065 | DSG4 | desmoglein 4 | 18 | q12.1 |
| ENSG00000134757 | DSG3 | desmoglein 3 | 18 | q12.1 |
| ENSG00000046604 | DSG2 | desmoglein 2 | 18 | q12.1 |
| ENSG00000128228 | SDF2L1 | stromal cell-derived factor 2-like 1 | 22 | q11.21 |
| ENSG00000142166 | IFNAR1 | interferon (alpha, beta and omega) receptor 1 | 21 | q22.11 |
| ENSG00000100023 | PPIL2 | peptidylprolyl isomerase (cyclophilin)-like 2 | 22 | q11.21 |
| ENSG00000159128 | IFNGR2 | interferon gamma receptor 2 (interferon gamma transducer 1) | 21 | q22.11 |
| ENSG00000100030 | MAPK1 | mitogen-activated protein kinase 1 | 22 | q11.22 |
| ENSG00000100814 | CCNB1IP1 | cyclin B1 interacting protein 1, E3 ubiquitin protein ligase | 14 | q11.2 |
| ENSG00000100034 | PPM1F | protein phosphatase, Mg2+/Mn2+ dependent, 1F | 22 | q11.22 |
| ENSG00000169575 | VPREB1 | pre-B lymphocyte 1 | 22 | q11.22 |
| ENSG00000185686 | PRAME | preferentially expressed antigen in melanoma | 22 | q11.22 |
| ENSG00000198805 | PNP | purine nucleoside phosphorylase | 14 | q11.2 |
| ENSG00000159140 | SON | SON DNA binding protein | 21 | q22.11 |
| ENSG00000169385 | RNASE2 | ribonuclease, RNase A family, 2 (liver, eosinophil-derived neurotoxin) | 14 | q11.2 |
| ENSG00000128322 | IGLL1 | immunoglobulin lambda-like polypeptide 1 | 22 | q11.23 |
| ENSG00000205726 | ITSN1 | intersectin 1 (SH3 domain protein) | 21 | q22.11 |
| ENSG00000128218 | VPREB3 | pre-B lymphocyte 3 | 22 | q11.23 |
| ENSG00000165804 | ZNF219 | zinc finger protein 219 | 14 | q11.2 |
| ENSG00000100888 | CHD8 | chromodomain helicase DNA binding protein 8 | 14 | q11.2 |
| ENSG00000129562 | DAD1 | defender against cell death 1 | 14 | q11.2 |
| ENSG00000141469 | SLC14A1 | solute carrier family 14 (urea transporter), member 1 (Kidd blood group) | 18 | q12.3 |
| ENSG00000159216 | RUNX1 | runt-related transcription factor 1 | 21 | q22.12 |
| ENSG00000128271 | ADORA2A | adenosine A2a receptor | 22 | q11.23 |
| ENSG00000197324 | LRP10 | low density lipoprotein receptor-related protein 10 | 14 | q11.2 |
| ENSG00000152234 | ATP5A1 | ATP synthase, H+ transporting, mitochondrial F1 complex, alpha subunit 1, cardiac muscle | 18 | q21.1 |
| ENSG00000159228 | CBR1 | carbonyl reductase 1 | 21 | q22.12 |
| ENSG00000184571 | PIWIL3 | piwi-like 3 (Drosophila) | 22 | q11.23 |
| ENSG00000129474 | AJUBA | ajuba LIM protein | 14 | q11.2 |
| ENSG00000100804 | PSMB5 | proteasome (prosome, macropain) subunit, beta type, 5 | 14 | q11.2 |
| ENSG00000159261 | CLDN14 | claudin 14 | 21 | q22.13 |
| ENSG00000206066 | IGLL3P | immunoglobulin lambda-like polypeptide 3, pseudogene | 22 | q11.23 |
| ENSG00000100068 | LRP5L | low density lipoprotein receptor-related protein 5-like | 22 | q11.23 |
| ENSG00000139880 | CDH24 | cadherin 24, type 2 | 14 | q11.2 |
| ENSG00000100813 | ACIN1 | apoptotic chromatin condensation inducer 1 | 14 | q11.2 |
| ENSG00000092067 | CEBPE | CCAAT/enhancer binding protein (C/EBP), epsilon | 14 | q11.2 |
| ENSG00000175387 | SMAD2 | SMAD family member 2 | 18 | q21.1 |
| ENSG00000100099 | HPS4 | Hermansky-Pudlak syndrome 4 | 22 | q12.1 |
| ENSG00000129473 | BCL2L2 | BCL2-like 2 | 14 | q11.2 |
| ENSG00000101665 | SMAD7 | SMAD family member 7 | 18 | q21.1 |
| ENSG00000100842 | EFS | embryonal Fyn-associated substrate | 14 | q11.2 |
| ENSG00000166090 | IL25 | interleukin 25 | 14 | q11.2 |
| ENSG00000166091 | CMTM5 | CKLF-like MARVEL transmembrane domain containing 5 | 14 | q11.2 |
| ENSG00000183765 | CHEK2 | checkpoint kinase 2 | 22 | q12.1 |
| ENSG00000183527 | PSMG1 | proteasome (prosome, macropain) assembly chaperone 1 | 21 | q22.2 |
| ENSG00000100867 | DHRS2 | dehydrogenase/reductase (SDR family) member 2 | 14 | q11.2 |
| ENSG00000141646 | SMAD4 | SMAD family member 4 | 18 | q21.2 |
| ENSG00000187323 | DCC | deleted in colorectal carcinoma | 18 | q21.2 |
| ENSG00000092010 | PSME1 | proteasome (prosome, macropain) activator subunit 1 (PA28 alpha) | 14 | q12 |
| ENSG00000100911 | PSME2 | proteasome (prosome, macropain) activator subunit 2 (PA28 beta) | 14 | q12 |
| ENSG00000100280 | AP1B1 | adaptor-related protein complex 1, beta 1 subunit | 22 | q12.2 |
| ENSG00000092098 | RNF31 | ring finger protein 31 | 14 | q12 |
| ENSG00000041353 | RAB27B | RAB27B, member RAS oncogene family | 18 | q21.2 |
| ENSG00000100296 | THOC5 | THO complex 5 | 22 | q12.2 |
| ENSG00000186575 | NF2 | neurofibromin 2 (merlin) | 22 | q12.2 |
| ENSG00000188403 | VSIG7 | V-set and immunoglobulin domain containing 7 | 15 | q11.1 |
| ENSG00000119547 | ONECUT2 | one cut homeobox 2 | 18 | q21.31 |
| ENSG00000183067 | IGSF5 | immunoglobulin superfamily, member 5 | 21 | q22.2 |
| ENSG00000171587 | DSCAM | Down syndrome cell adhesion molecule | 21 | q22.2 |
| ENSG00000049759 | NEDD4L | neural precursor cell expressed, developmentally down-regulated 4-like, E3 ubiquitin protein ligase | 18 | q21.31 |
| ENSG00000136305 | CIDEB | cell death-inducing DFFA-like effector b | 14 | q12 |
| ENSG00000183844 | FAM3B | family with sequence similarity 3, member B | 21 | q22.3 |
| ENSG00000129465 | RIPK3 | receptor-interacting serine-threonine kinase 3 | 14 | q12 |
| ENSG00000100968 | NFATC4 | nuclear factor of activated T-cells, cytoplasmic, calcineurin-dependent 4 | 14 | q12 |
| ENSG00000172175 | MALT1 | mucosa associated lymphoid tissue lymphoma translocation gene 1 | 18 | q21.32 |
| ENSG00000157601 | MX1 | myxovirus (influenza virus) resistance 1, interferon-inducible protein p78 (mouse) | 21 | q22.3 |
| ENSG00000128342 | LIF | leukemia inhibitory factor | 22 | q12.2 |
| ENSG00000092009 | CMA1 | chymase 1, mast cell | 14 | q12 |
| ENSG00000099985 | OSM | oncostatin M | 22 | q12.2 |
| ENSG00000100448 | CTSG | cathepsin G | 14 | q12 |
| ENSG00000100450 | GZMH | granzyme H (cathepsin G-like 2, protein h-CCPX) | 14 | q12 |
| ENSG00000100453 | GZMB | granzyme B (granzyme 2, cytotoxic T-lymphocyte-associated serine esterase 1) | 14 | q12 |
| ENSG00000166569 | CPLX4 | complexin 4 | 18 | q21.32 |
| ENSG00000157617 | C2CD2 | C2 calcium-dependent domain containing 2 | 21 | q22.3 |
| ENSG00000183287 | CCBE1 | collagen and calcium binding EGF domains 1 | 18 | q21.32 |
| ENSG00000141682 | PMAIP1 | phorbol-12-myristate-13-acetate-induced protein 1 | 18 | q21.32 |
| ENSG00000182636 | NDN | necdin homolog (mouse) | 15 | q11.2 |
| ENSG00000101542 | CDH20 | cadherin 20, type 2 | 18 | q21.33 |
| ENSG00000184304 | PRKD1 | protein kinase D1 | 14 | q12 |
| ENSG00000160179 | ABCG1 | ATP-binding cassette, sub-family G (WHITE), member 1 | 21 | q22.3 |
| ENSG00000092140 | G2E3 | G2/M-phase specific E3 ubiquitin protein ligase | 14 | q12 |
| ENSG00000092108 | SCFD1 | sec1 family domain containing 1 | 14 | q12 |
| ENSG00000114062 | UBE3A | ubiquitin protein ligase E3A | 15 | q11.2 |
| ENSG00000141655 | TNFRSF11A | tumor necrosis factor receptor superfamily, member 11a, NFKB activator | 18 | q21.33 |
| ENSG00000081913 | PHLPP1 | PH domain and leucine rich repeat protein phosphatase 1 | 18 | q21.33 |
| ENSG00000171791 | BCL2 | B-cell CLL/lymphoma 2 | 18 | q21.33 |
| ENSG00000160185 | UBASH3A | ubiquitin associated and SH3 domain containing A | 21 | q22.3 |
| ENSG00000160188 | RSPH1 | radial spoke head 1 homolog (Chlamydomonas) | 21 | q22.3 |
| ENSG00000100852 | ARHGAP5 | Rho GTPase activating protein 5 | 14 | q12 |
| ENSG00000129521 | EGLN3 | egl nine homolog 3 (C. elegans) | 14 | q13.1 |
| ENSG00000197632 | SERPINB2 | serpin peptidase inhibitor, clade B (ovalbumin), member 2 | 18 | q21.33 |
| ENSG00000129515 | SNX6 | sorting nexin 6 | 14 | q13.1 |
| ENSG00000100105 | PATZ1 | POZ (BTB) and AT hook containing zinc finger 1 | 22 | q12.2 |
| ENSG00000160199 | PKNOX1 | PBX/knotted 1 homeobox 1 | 21 | q22.3 |
| ENSG00000081138 | CDH7 | cadherin 7, type 2 | 18 | q22.1 |
| ENSG00000071991 | CDH19 | cadherin 19, type 2 | 18 | q22.1 |
| ENSG00000160202 | CRYAA | crystallin, alpha A | 21 | q22.3 |
| ENSG00000142178 | SIK1 | salt-inducible kinase 1 | 21 | q22.3 |
| ENSG00000100902 | PSMA6 | proteasome (prosome, macropain) subunit, alpha type, 6 | 14 | q13.2 |
| ENSG00000100906 | NFKBIA | nuclear factor of kappa light polypeptide gene enhancer in B-cells inhibitor, alpha | 14 | q13.2 |
| ENSG00000150637 | CD226 | CD226 molecule | 18 | q22.2 |
| ENSG00000170677 | SOCS6 | suppressor of cytokine signaling 6 | 18 | q22.2 |
| ENSG00000160213 | CSTB | cystatin B (stefin B) | 21 | q22.3 |
| ENSG00000198690 | FAN1 | FANCD2/FANCI-associated nuclease 1 | 15 | q13.3 |
| ENSG00000136352 | NKX2-1 | NK2 homeobox 1 | 14 | q13.3 |
| ENSG00000100191 | SLC5A4 | solute carrier family 5 (low affinity glucose cotransporter), member 4 | 22 | q12.3 |
| ENSG00000175344 | CHRNA7 | cholinergic receptor, nicotinic, alpha 7 (neuronal) | 15 | q13.3 |
| ENSG00000160223 | ICOSLG | inducible T-cell co-stimulator ligand | 21 | q22.3 |
| ENSG00000100220 | C22orf28 | chromosome 22 open reading frame 28 | 22 | q12.3 |
| ENSG00000184459 | BPIFC | BPI fold containing family C | 22 | q12.3 |
| ENSG00000160224 | AIRE | autoimmune regulator | 21 | q22.3 |
| ENSG00000166923 | GREM1 | gremlin 1 | 15 | q13.3 |
| ENSG00000100941 | PNN | pinin, desmosome associated protein | 14 | q21.1 |
| ENSG00000169857 | AVEN | apoptosis, caspase activation inhibitor | 15 | q14 |
| ENSG00000100284 | TOM1 | target of myb1 (chicken) | 22 | q12.3 |
| ENSG00000175894 | TSPEAR | thrombospondin-type laminin G domain and EAR repeats | 21 | q22.3 |
| ENSG00000100292 | HMOX1 | heme oxygenase (decycling) 1 | 22 | q12.3 |
| ENSG00000128463 | EMC4 | ER membrane protein complex subunit 4 | 15 | q14 |
| ENSG00000182372 | CLN8 | ceroid-lipofuscinosis, neuronal 8 (epilepsy, progressive with mental retardation) | 8 | p23.3 |
| ENSG00000131196 | NFATC1 | nuclear factor of activated T-cells, cytoplasmic, calcineurin-dependent 1 | 18 | q23 |
| ENSG00000198125 | MB | myoglobin | 22 | q12.3 |
| ENSG00000100442 | FKBP3 | FK506 binding protein 3, 25kDa | 14 | q21.2 |
| ENSG00000187790 | FANCM | Fanconi anemia, complementation group M | 14 | q21.2 |
| ENSG00000160255 | ITGB2 | integrin, beta 2 (complement component 3 receptor 3 and 4 subunit) | 21 | q22.3 |
| ENSG00000159251 | ACTC1 | actin, alpha, cardiac muscle 1 | 15 | q14 |
| ENSG00000165501 | LRR1 | leucine rich repeat protein 1 | 14 | q21.3 |
| ENSG00000197381 | ADARB1 | adenosine deaminase, RNA-specific, B1 | 21 | q22.3 |
| ENSG00000128335 | APOL2 | apolipoprotein L, 2 | 22 | q12.3 |
| ENSG00000100345 | MYH9 | myosin, heavy chain 9, non-muscle | 22 | q12.3 |
| ENSG00000182871 | COL18A1 | collagen, type XVIII, alpha 1 | 21 | q22.3 |
| ENSG00000165527 | ARF6 | ADP-ribosylation factor 6 | 14 | q21.3 |
| ENSG00000091879 | ANGPT2 | angiopoietin 2 | 8 | p23.1 |
| ENSG00000100485 | SOS2 | son of sevenless homolog 2 (Drosophila) | 14 | q21.3 |
| ENSG00000137801 | THBS1 | thrombospondin 1 | 15 | q14 |
| ENSG00000100348 | TXN2 | thioredoxin 2 | 22 | q12.3 |
| ENSG00000164825 | DEFB1 | defensin, beta 1 | 8 | p23.1 |
| ENSG00000164822 | DEFA6 | defensin, alpha 6, Paneth cell-specific | 8 | p23.1 |
| ENSG00000012983 | MAP4K5 | mitogen-activated protein kinase kinase kinase kinase 5 | 14 | q22.1 |
| ENSG00000142156 | COL6A1 | collagen, type VI, alpha 1 | 21 | q22.3 |
| ENSG00000100360 | IFT27 | intraflagellar transport 27 homolog (Chlamydomonas) | 22 | q12.3 |
| ENSG00000164821 | DEFA4 | defensin, alpha 4, corticostatin | 8 | p23.1 |
| ENSG00000198513 | ATL1 | atlastin GTPase 1 | 14 | q22.1 |
| ENSG00000104081 | BMF | Bcl2 modifying factor | 15 | q15.1 |
| ENSG00000197461 | PDGFA | platelet-derived growth factor alpha polypeptide | 7 | p22.3 |
| ENSG00000206047 | DEFA1 | defensin, alpha 1 | 8 | p23.1 |
| ENSG00000142173 | COL6A2 | collagen, type VI, alpha 2 | 21 | q22.3 |
| ENSG00000156970 | BUB1B | budding uninhibited by benzimidazoles 1 homolog beta (yeast) | 15 | q15.1 |
| ENSG00000168993 | CPLX1 | complexin 1 | 4 | p16.3 |
| ENSG00000188191 | PRKAR1B | protein kinase, cAMP-dependent, regulatory, type I, beta | 7 | p22.3 |
| ENSG00000100368 | CSF2RB | colony stimulating factor 2 receptor, beta, low-affinity (granulocyte-macrophage) | 22 | q12.3 |
| ENSG00000206042 | DEFA7P | defensin, alpha 7 pseudogene | 8 | p23.1 |
| ENSG00000164816 | DEFA5 | defensin, alpha 5, Paneth cell-specific | 8 | p23.1 |
| ENSG00000145214 | DGKQ | diacylglycerol kinase, theta 110kDa | 4 | p16.3 |
| ENSG00000206034 | DEFB109P1B | defensin, beta 109, pseudogene 1B | 8 | p23.1 |
| ENSG00000177257 | DEFB4B | defensin, beta 4B | 8 | p23.1 |
| ENSG00000177243 | DEFB103B | defensin, beta 103B | 8 | p23.1 |
| ENSG00000177023 | DEFB104B | defensin, beta 104B | 8 | p23.1 |
| ENSG00000187082 | DEFB106B | defensin, beta 106B | 8 | p23.1 |
| ENSG00000100385 | IL2RB | interleukin 2 receptor, beta | 22 | q12.3 |
| ENSG00000186599 | DEFB105B | defensin, beta 105B | 8 | p23.1 |
| ENSG00000133466 | C1QTNF6 | C1q and tumor necrosis factor related protein 6 | 22 | q12.3 |
| ENSG00000198129 | DEFB107B | defensin, beta 107B | 8 | p23.1 |
| ENSG00000159674 | SPON2 | spondin 2, extracellular matrix protein | 4 | p16.3 |
| ENSG00000164877 | MICALL2 | MICAL-like 2 | 7 | p22.3 |
| ENSG00000128340 | RAC2 | ras-related C3 botulinum toxin substrate 2 (rho family, small GTP binding protein Rac2) | 22 | q13.1 |
| ENSG00000159692 | CTBP1 | C-terminal binding protein 1 | 4 | p16.3 |
| ENSG00000139921 | TMX1 | thioredoxin-related transmembrane protein 1 | 14 | q22.1 |
| ENSG00000137824 | FAM82A2 | family with sequence similarity 82, member A2 | 15 | q15.1 |
| ENSG00000186572 | DEFB107A | defensin, beta 107A | 8 | p23.1 |
| ENSG00000186562 | DEFB105A | defensin, beta 105A | 8 | p23.1 |
| ENSG00000186579 | DEFB106A | defensin, beta 106A | 8 | p23.1 |
| ENSG00000090316 | MAEA | macrophage erythroblast attacher | 4 | p16.3 |
| ENSG00000100065 | CARD10 | caspase recruitment domain family, member 10 | 22 | q13.1 |
| ENSG00000176782 | DEFB104A | defensin, beta 104A | 8 | p23.1 |
| ENSG00000087303 | NID2 | nidogen 2 (osteonidogen) | 14 | q22.1 |
| ENSG00000157778 | PSMG3 | proteasome (prosome, macropain) assembly chaperone 3 | 7 | p22.3 |
| ENSG00000176797 | DEFB103A | defensin, beta 103A | 8 | p23.1 |
| ENSG00000168229 | PTGDR | prostaglandin D2 receptor (DP) | 14 | q22.1 |
| ENSG00000171711 | DEFB4A | defensin, beta 4A | 8 | p23.1 |
| ENSG00000125384 | PTGER2 | prostaglandin E receptor 2 (subtype EP2), 53kDa | 14 | q22.1 |
| ENSG00000205989 | DEFB109P1 | defensin, beta 109, pseudogene 1 | 8 | p23.1 |
| ENSG00000197930 | ERO1L | ERO1-like (S. cerevisiae) | 14 | q22.1 |
| ENSG00000100519 | PSMC6 | proteasome (prosome, macropain) 26S subunit, ATPase, 6 | 14 | q22.1 |
| ENSG00000128965 | CHAC1 | ChaC, cation transport regulator homolog 1 (E. coli) | 15 | q15.1 |
| ENSG00000073712 | FERMT2 | fermitin family member 2 | 14 | q22.1 |
| ENSG00000100097 | LGALS1 | lectin, galactoside-binding, soluble, 1 | 22 | q13.1 |
| ENSG00000125378 | BMP4 | bone morphogenetic protein 4 | 14 | q22.2 |
| ENSG00000187446 | CHP1 | calcineurin-like EF hand protein 1 | 15 | q15.1 |
| ENSG00000137804 | NUSAP1 | nucleolar and spindle associated protein 1 | 15 | q15.1 |
| ENSG00000100124 | ANKRD54 | ankyrin repeat domain 54 | 22 | q13.1 |
| ENSG00000171056 | SOX7 | SRY (sex determining region Y)-box 7 | 8 | p23.1 |
| ENSG00000206172 | HBA1 | hemoglobin, alpha 1 | 16 | p13.3 |
| ENSG00000146535 | GNA12 | guanine nucleotide binding protein (G protein) alpha 12 | 7 | p22.2 |
| ENSG00000062524 | LTK | leukocyte receptor tyrosine kinase | 15 | q15.1 |
| ENSG00000198286 | CARD11 | caspase recruitment domain family, member 11 | 7 | p22.2 |
| ENSG00000131979 | GCH1 | GTP cyclohydrolase 1 | 14 | q22.2 |
| ENSG00000171044 | XKR6 | XK, Kell blood group complex subunit-related family, member 6 | 8 | p23.1 |
| ENSG00000146555 | SDK1 | sidekick cell adhesion molecule 1 | 7 | p22.2 |
| ENSG00000092445 | TYRO3 | TYRO3 protein tyrosine kinase | 15 | q15.1 |
| ENSG00000180008 | SOCS4 | suppressor of cytokine signaling 4 | 14 | q22.3 |
| ENSG00000167930 | ITFG3 | integrin alpha FG-GAP repeat containing 3 | 16 | p13.3 |
| ENSG00000100151 | PICK1 | protein interacting with PRKCA 1 | 22 | q13.1 |
| ENSG00000157927 | RADIL | Ras association and DIL domains | 7 | p22.1 |
| ENSG00000136573 | BLK | B lymphoid tyrosine kinase | 8 | p23.1 |
| ENSG00000136297 | MMD2 | monocyte to macrophage differentiation-associated 2 | 7 | p22.1 |
| ENSG00000185615 | PDIA2 | protein disulfide isomerase family A, member 2 | 16 | p13.3 |
| ENSG00000103126 | AXIN1 | axin 1 | 16 | p13.3 |
| ENSG00000103966 | EHD4 | EH-domain containing 4 | 15 | q15.1 |
| ENSG00000129925 | TMEM8A | transmembrane protein 8A | 16 | p13.3 |
| ENSG00000154328 | NEIL2 | nei endonuclease VIII-like 2 (E. coli) | 8 | p23.1 |
| ENSG00000070367 | EXOC5 | exocyst complex component 5 | 14 | q22.3 |
| ENSG00000168884 | TNIP2 | TNFAIP3 interacting protein 2 | 4 | p16.3 |
| ENSG00000090565 | RAB11FIP3 | RAB11 family interacting protein 3 (class II) | 16 | p13.3 |
| ENSG00000100211 | CBY1 | chibby homolog 1 (Drosophila) | 22 | q13.1 |
| ENSG00000164733 | CTSB | cathepsin B | 8 | p23.1 |
| ENSG00000075618 | FSCN1 | fascin homolog 1, actin-bundling protein (Strongylocentrotus purpuratus) | 7 | p22.1 |
| ENSG00000011275 | RNF216 | ring finger protein 216 | 7 | p22.1 |
| ENSG00000109736 | MFSD10 | major facilitator superfamily domain containing 10 | 4 | p16.3 |
| ENSG00000100567 | PSMA3 | proteasome (prosome, macropain) subunit, alpha type, 3 | 14 | q23.1 |
| ENSG00000205884 | DEFB136 | defensin, beta 136 | 8 | p23.1 |
| ENSG00000205883 | DEFB135 | defensin, beta 135 | 8 | p23.1 |
| ENSG00000205882 | DEFB134 | defensin, beta 134 | 8 | p23.1 |
| ENSG00000127578 | WFIKKN1 | WAP, follistatin/kazal, immunoglobulin, kunitz and netrin domain containing 1 | 16 | p13.3 |
| ENSG00000197386 | HTT | huntingtin | 4 | p16.3 |
| ENSG00000155026 | RSPH10B | radial spoke head 10 homolog B (Chlamydomonas) | 7 | p22.1 |
| ENSG00000122512 | PMS2 | PMS2 postmeiotic segregation increased 2 (S. cerevisiae) | 7 | p22.1 |
| ENSG00000092531 | SNAP23 | synaptosomal-associated protein, 23kDa | 15 | q15.1 |
| ENSG00000164741 | DLC1 | deleted in liver cancer 1 | 8 | p22 |
| ENSG00000140983 | RHOT2 | ras homolog family member T2 | 16 | p13.3 |
| ENSG00000106305 | AIMP2 | aminoacyl tRNA synthetase complex-interacting multifunctional protein 2 | 7 | p22.1 |
| ENSG00000008256 | CYTH3 | cytohesin 3 | 7 | p22.1 |
| ENSG00000100311 | PDGFB | platelet-derived growth factor beta polypeptide | 22 | q13.1 |
| ENSG00000136238 | RAC1 | ras-related C3 botulinum toxin substrate 1 (rho family, small GTP binding protein Rac1) | 7 | p22.1 |
| ENSG00000159459 | UBR1 | ubiquitin protein ligase E3 component n-recognin 1 | 15 | q15.2 |
| ENSG00000102854 | MSLN | mesothelin | 16 | p13.3 |
| ENSG00000100324 | TAB1 | TGF-beta activated kinase 1/MAP3K7 binding protein 1 | 22 | q13.1 |
| ENSG00000038945 | MSR1 | macrophage scavenger receptor 1 | 8 | p22 |
| ENSG00000126778 | SIX1 | SIX homeobox 1 | 14 | q23.1 |
| ENSG00000162006 | MSLNL | mesothelin-like | 16 | p13.3 |
| ENSG00000100625 | SIX4 | SIX homeobox 4 | 14 | q23.1 |
| ENSG00000020426 | MNAT1 | menage a trois homolog 1, cyclin H assembly factor (Xenopus laevis) | 14 | q23.1 |
| ENSG00000187953 | PMS2CL | PMS2 C-terminal like pseudogene | 7 | p22.1 |
| ENSG00000168803 | ADAL | adenosine deaminase-like | 15 | q15.3 |
| ENSG00000169402 | RSPH10B2 | radial spoke head 10 homolog B2 (Chlamydomonas) | 7 | p22.1 |
| ENSG00000145220 | LYAR | Ly1 antibody reactive homolog (mouse) | 4 | p16.3 |
| ENSG00000005513 | SOX8 | SRY (sex determining region Y)-box 8 | 16 | p13.3 |
| ENSG00000027075 | PRKCH | protein kinase C, eta | 14 | q23.1 |
| ENSG00000100644 | HIF1A | hypoxia inducible factor 1, alpha subunit (basic helix-loop-helix transcription factor) | 14 | q23.2 |
| ENSG00000184471 | C1QTNF8 | C1q and tumor necrosis factor related protein 8 | 16 | p13.3 |
| ENSG00000003147 | ICA1 | islet cell autoantigen 1, 69kDa | 7 | p21.3 |
| ENSG00000163132 | MSX1 | msh homeobox 1 | 4 | p16.2 |
| ENSG00000170891 | CYTL1 | cytokine-like 1 | 4 | p16.2 |
| ENSG00000006747 | SCIN | scinderin | 7 | p21.3 |
| ENSG00000104213 | PDGFRL | platelet-derived growth factor receptor-like | 8 | p22 |
| ENSG00000122644 | ARL4A | ADP-ribosylation factor-like 4A | 7 | p21.3 |
| ENSG00000126821 | SGPP1 | sphingosine-1-phosphate phosphatase 1 | 14 | q23.2 |
| ENSG00000196588 | MKL1 | megakaryoblastic leukemia (translocation) 1 | 22 | q13.2 |
| ENSG00000136267 | DGKB | diacylglycerol kinase, beta 90kDa | 7 | p21.2 |
| ENSG00000152969 | JAKMIP1 | janus kinase and microtubule interacting protein 1 | 4 | p16.1 |
| ENSG00000109501 | WFS1 | Wolfram syndrome 1 (wolframin) | 4 | p16.1 |
| ENSG00000100393 | EP300 | E1A binding protein p300 | 22 | q13.2 |
| ENSG00000106546 | AHR | aryl hydrocarbon receptor | 7 | p21.1 |
| ENSG00000140009 | ESR2 | estrogen receptor 2 (ER beta) | 14 | q23.3 |
| ENSG00000048052 | HDAC9 | histone deacetylase 9 | 7 | p21.1 |
| ENSG00000187535 | IFT140 | intraflagellar transport 140 homolog (Chlamydomonas) | 16 | p13.3 |
| ENSG00000122691 | TWIST1 | twist homolog 1 (Drosophila) | 7 | p21.1 |
| ENSG00000167004 | PDIA3 | protein disulfide isomerase family A, member 3 | 15 | q15.3 |
| ENSG00000105855 | ITGB8 | integrin, beta 8 | 7 | p21.1 |
| ENSG00000103024 | NME3 | NME/NM23 nucleoside diphosphate kinase 3 | 16 | p13.3 |
| ENSG00000196419 | XRCC6 | X-ray repair complementing defective repair in Chinese hamster cells 6 | 22 | q13.2 |
| ENSG00000099769 | IGFALS | insulin-like growth factor binding protein, acid labile subunit | 16 | p13.3 |
| ENSG00000136244 | IL6 | interleukin 6 (interferon, beta 2) | 7 | p15.3 |
| ENSG00000159958 | TNFRSF13C | tumor necrosis factor receptor superfamily, member 13C | 22 | q13.2 |
| ENSG00000125952 | MAX | MYC associated factor X | 14 | q23.3 |
| ENSG00000100167 | SEPT3 | septin 3 | 22 | q13.2 |
| ENSG00000196408 | NOXO1 | NADPH oxidase organizer 1 | 16 | p13.3 |
| ENSG00000033170 | FUT8 | fucosyltransferase 8 (alpha (1,6) fucosyltransferase) | 14 | q23.3 |
| ENSG00000136235 | GPNMB | glycoprotein (transmembrane) nmb | 7 | p15.3 |
| ENSG00000175445 | LPL | lipoprotein lipase | 8 | p21.3 |
| ENSG00000136231 | IGF2BP3 | insulin-like growth factor 2 mRNA binding protein 3 | 7 | p15.3 |
| ENSG00000103197 | TSC2 | tuberous sclerosis 2 | 16 | p13.3 |
| ENSG00000186146 | DEFB131 | defensin, beta 131 | 4 | p16.1 |
| ENSG00000169676 | DRD5 | dopamine receptor D5 | 4 | p16.1 |
| ENSG00000166710 | B2M | beta-2-microglobulin | 15 | q21.1 |
| ENSG00000008710 | PKD1 | polycystic kidney disease 1 (autosomal dominant) | 16 | p13.3 |
| ENSG00000185880 | TRIM69 | tripartite motif containing 69 | 15 | q21.1 |
| ENSG00000100568 | VTI1B | vesicle transport through interaction with t-SNAREs homolog 1B (yeast) | 14 | q24.1 |
| ENSG00000172115 | CYCS | cytochrome c, somatic | 7 | p15.3 |
| ENSG00000109684 | CLNK | cytokine-dependent hematopoietic cell linker | 4 | p16.1 |
| ENSG00000167964 | RAB26 | RAB26, member RAS oncogene family | 16 | p13.3 |
| ENSG00000140279 | DUOX2 | dual oxidase 2 | 15 | q21.1 |
| ENSG00000131653 | TRAF7 | TNF receptor-associated factor 7, E3 ubiquitin protein ligase | 16 | p13.3 |
| ENSG00000050344 | NFE2L3 | nuclear factor (erythroid-derived 2)-like 3 | 7 | p15.2 |
| ENSG00000137857 | DUOX1 | dual oxidase 1 | 15 | q21.1 |
| ENSG00000109705 | NKX3-2 | NK3 homeobox 2 | 4 | p15.33 |
| ENSG00000072110 | ACTN1 | actinin, alpha 1 | 14 | q24.1 |
| ENSG00000005020 | SKAP2 | src kinase associated phosphoprotein 2 | 7 | p15.2 |
| ENSG00000138606 | SHF | Src homology 2 domain containing F | 15 | q21.1 |
| ENSG00000180104 | EXOC3 | exocyst complex component 3 | 5 | p15.33 |
| ENSG00000100266 | PACSIN2 | protein kinase C and casein kinase substrate in neurons 2 | 22 | q13.2 |
| ENSG00000066230 | SLC9A3 | solute carrier family 9, subfamily A (NHE3, cation proton antiporter 3), member 3 | 5 | p15.33 |
| ENSG00000168481 | LGI3 | leucine-rich repeat LGI family, member 3 | 8 | p21.3 |
| ENSG00000105997 | HOXA3 | homeobox A3 | 7 | p15.2 |
| ENSG00000100626 | GALNTL1 | UDP-N-acetyl-alpha-D-galactosamine:polypeptide N-acetylgalactosaminyltransferase-like 1 | 14 | q24.1 |
| ENSG00000100290 | BIK | BCL2-interacting killer (apoptosis-inducing) | 22 | q13.2 |
| ENSG00000168487 | BMP1 | bone morphogenetic protein 1 | 8 | p21.3 |
| ENSG00000163145 | C1QTNF7 | C1q and tumor necrosis factor related protein 7 | 4 | p15.32 |
| ENSG00000106004 | HOXA5 | homeobox A5 | 7 | p15.2 |
| ENSG00000122592 | HOXA7 | homeobox A7 | 7 | p15.2 |
| ENSG00000100300 | TSPO | translocator protein (18kDa) | 22 | q13.2 |
| ENSG00000104164 | PLDN | biogenesis of lysosomal organelles complex-1, subunit 6, pallidin | 15 | q21.1 |
| ENSG00000198732 | SMOC1 | SPARC related modular calcium binding 1 | 14 | q24.2 |
| ENSG00000109743 | BST1 | bone marrow stromal cell antigen 1 | 4 | p15.32 |
| ENSG00000106031 | HOXA13 | homeobox A13 | 7 | p15.2 |
| ENSG00000004468 | CD38 | CD38 molecule | 4 | p15.32 |
| ENSG00000106052 | TAX1BP1 | Tax1 (human T-cell leukemia virus type I) binding protein 1 | 7 | p15.2 |
| ENSG00000168495 | POLR3D | polymerase (RNA) III (DNA directed) polypeptide D, 44kDa | 8 | p21.3 |
| ENSG00000197181 | PIWIL2 | piwi-like 2 (Drosophila) | 8 | p21.3 |
| ENSG00000145506 | NKD2 | naked cuticle homolog 2 (Drosophila) | 5 | p15.33 |
| ENSG00000188677 | PARVB | parvin, beta | 22 | q13.31 |
| ENSG00000136193 | SCRN1 | secernin 1 | 7 | p14.3 |
| ENSG00000138964 | PARVG | parvin, gamma | 22 | q13.31 |
| ENSG00000106080 | FKBP14 | FK506 binding protein 14, 22 kDa | 7 | p14.3 |
| ENSG00000120896 | SORBS3 | sorbin and SH3 domain containing 3 | 8 | p21.3 |
| ENSG00000164362 | TERT | telomerase reverse transcriptase | 5 | p15.33 |
| ENSG00000106100 | NOD1 | nucleotide-binding oligomerization domain containing 1 | 7 | p14.3 |
| ENSG00000049656 | CLPTM1L | CLPTM1-like | 5 | p15.33 |
| ENSG00000158941 | KIAA1967 | KIAA1967 | 8 | p21.3 |
| ENSG00000119707 | RBM25 | RNA binding motif protein 25 | 14 | q24.2 |
| ENSG00000147439 | BIN3 | bridging integrator 3 | 8 | p21.3 |
| ENSG00000080815 | PSEN1 | presenilin 1 | 14 | q24.2 |
| ENSG00000145147 | SLIT2 | slit homolog 2 (Drosophila) | 4 | p15.31 |
| ENSG00000184697 | CLDN6 | claudin 6 | 16 | p13.3 |
| ENSG00000006327 | TNFRSF12A | tumor necrosis factor receptor superfamily, member 12A | 16 | p13.3 |
| ENSG00000103145 | HCFC1R1 | host cell factor C1 regulator 1 (XPO1 dependent) | 16 | p13.3 |
| ENSG00000120889 | TNFRSF10B | tumor necrosis factor receptor superfamily, member 10b | 8 | p21.3 |
| ENSG00000171840 | NINJ2 | ninjurin 2 | 12 | p13.33 |
| ENSG00000140285 | FGF7 | fibroblast growth factor 7 | 15 | q21.2 |
| ENSG00000008517 | IL32 | interleukin 32 | 16 | p13.3 |
| ENSG00000060237 | WNK1 | WNK lysine deficient protein kinase 1 | 12 | p13.33 |
| ENSG00000106341 | PPP1R17 | protein phosphatase 1, regulatory subunit 17 | 7 | p14.3 |
| ENSG00000173535 | TNFRSF10C | tumor necrosis factor receptor superfamily, member 10c, decoy without an intracellular domain | 8 | p21.3 |
| ENSG00000140287 | HDC | histidine decarboxylase | 15 | q21.2 |
| ENSG00000173530 | TNFRSF10D | tumor necrosis factor receptor superfamily, member 10d, decoy with truncated death domain | 8 | p21.3 |
| ENSG00000140043 | PTGR2 | prostaglandin reductase 2 | 14 | q24.3 |
| ENSG00000082805 | ERC1 | ELKS/RAB6-interacting/CAST family member 1 | 12 | p13.33 |
| ENSG00000104689 | TNFRSF10A | tumor necrosis factor receptor superfamily, member 10a | 8 | p21.3 |
| ENSG00000122642 | FKBP9 | FK506 binding protein 9, 63 kDa | 7 | p14.3 |
| ENSG00000109819 | PPARGC1A | peroxisome proliferator-activated receptor gamma, coactivator 1 alpha | 4 | p15.2 |
| ENSG00000186951 | PPARA | peroxisome proliferator-activated receptor alpha | 22 | q13.31 |
| ENSG00000092439 | TRPM7 | transient receptor potential cation channel, subfamily M, member 7 | 15 | q21.2 |
| ENSG00000134013 | LOXL2 | lysyl oxidase-like 2 | 8 | p21.3 |
| ENSG00000122507 | BBS9 | Bardet-Biedl syndrome 9 | 7 | p14.3 |
| ENSG00000119711 | ALDH6A1 | aldehyde dehydrogenase 6 family, member A1 | 14 | q24.3 |
| ENSG00000111186 | WNT5B | wingless-type MMTV integration site family, member 5B | 12 | p13.33 |
| ENSG00000119688 | ABCD4 | ATP-binding cassette, sub-family D (ALD), member 4 | 14 | q24.3 |
| ENSG00000124279 | FASTKD3 | FAST kinase domains 3 | 5 | p15.31 |
| ENSG00000183578 | TNFAIP8L3 | tumor necrosis factor, alpha-induced protein 8-like 3 | 15 | q21.2 |
| ENSG00000197085 | NPSR1-AS1 | NPSR1 antisense RNA 1 | 7 | p14.3 |
| ENSG00000167984 | NLRC3 | NLR family, CARD domain containing 3 | 16 | p13.3 |
| ENSG00000075275 | CELSR1 | cadherin, EGF LAG seven-pass G-type receptor 1 (flamingo homolog, Drosophila) | 22 | q13.31 |
| ENSG00000183186 | C2CD4C | C2 calcium-dependent domain containing 4C | 19 | p13.3 |
| ENSG00000112902 | SEMA5A | sema domain, seven thrombospondin repeats (type 1 and type 1-like), transmembrane domain (TM) and short cytoplasmic domain, (semaphorin) 5A | 5 | p15.31 |
| ENSG00000075240 | GRAMD4 | GRAM domain containing 4 | 22 | q13.31 |
| ENSG00000119681 | LTBP2 | latent transforming growth factor beta binding protein 2 | 14 | q24.3 |
| ENSG00000180053 | NKX2-6 | NK2 homeobox 6 | 8 | p21.2 |
| ENSG00000099866 | MADCAM1 | mucosal vascular addressin cell adhesion molecule 1 | 19 | p13.3 |
| ENSG00000100422 | CERK | ceramide kinase | 22 | q13.31 |
| ENSG00000126602 | TRAP1 | TNF receptor-associated protein 1 | 16 | p13.3 |
| ENSG00000004478 | FKBP4 | FK506 binding protein 4, 59kDa | 12 | p13.33 |
| ENSG00000119616 | FCF1 | FCF1 small subunit (SSU) processome component homolog (S. cerevisiae) | 14 | q24.3 |
| ENSG00000172270 | BSG | basigin (Ok blood group) | 19 | p13.3 |
| ENSG00000134028 | ADAMDEC1 | ADAM-like, decysin 1 | 8 | p21.2 |
| ENSG00000111203 | ITFG2 | integrin alpha FG-GAP repeat containing 2 | 12 | p13.33 |
| ENSG00000137875 | BCL2L10 | BCL2-like 10 (apoptosis facilitator) | 15 | q21.2 |
| ENSG00000122545 | SEPT7 | septin 7 | 7 | p14.2 |
| ENSG00000197540 | GZMM | granzyme M (lymphocyte met-ase 1) | 19 | p13.3 |
| ENSG00000112977 | DAP | death-associated protein | 5 | p15.2 |
| ENSG00000168214 | RBPJ | recombination signal binding protein for immunoglobulin kappa J region | 4 | p15.2 |
| ENSG00000169862 | CTNND2 | catenin (cadherin-associated protein), delta 2 | 5 | p15.2 |
| ENSG00000011426 | ANLN | anillin, actin binding protein | 7 | p14.2 |
| ENSG00000090447 | TFAP4 | transcription factor AP-4 (activating enhancer binding protein 4) | 16 | p13.3 |
| ENSG00000155849 | ELMO1 | engulfment and cell motility 1 | 7 | p14.1 |
| ENSG00000147459 | DOCK5 | dedicator of cytokinesis 5 | 8 | p21.2 |
| ENSG00000198355 | PIM3 | pim-3 oncogene | 22 | q13.33 |
| ENSG00000163394 | CCKAR | cholecystokinin A receptor | 4 | p15.2 |
| ENSG00000188263 | IL17REL | interleukin 17 receptor E-like | 22 | q13.33 |
| ENSG00000070404 | FSTL3 | follistatin-like 3 (secreted glycoprotein) | 19 | p13.3 |
| ENSG00000103423 | DNAJA3 | DnaJ (Hsp40) homolog, subfamily A, member 3 | 16 | p13.3 |
| ENSG00000169856 | ONECUT1 | one cut homeobox 1 | 15 | q21.3 |
| ENSG00000073146 | MOV10L1 | Mov10l1, Moloney leukemia virus 10-like 1, homolog (mouse) | 22 | q13.33 |
| ENSG00000106483 | SFRP4 | secreted frizzled-related protein 4 | 7 | p14.1 |
| ENSG00000147437 | GNRH1 | gonadotropin-releasing hormone 1 (luteinizing-releasing hormone) | 8 | p21.2 |
| ENSG00000038382 | TRIO | trio Rho guanine nucleotide exchange factor | 5 | p15.2 |
| ENSG00000137766 | UNC13C | unc-13 homolog C (C. elegans) | 15 | q21.3 |
| ENSG00000089486 | CDIP1 | cell death-inducing p53 target 1 | 16 | p13.3 |
| ENSG00000078053 | AMPH | amphiphysin | 7 | p14.1 |
| ENSG00000170345 | FOS | FBJ murine osteosarcoma viral oncogene homolog | 14 | q24.3 |
| ENSG00000069974 | RAB27A | RAB27A, member RAS oncogene family | 15 | q21.3 |
| ENSG00000169851 | PCDH7 | protocadherin 7 | 4 | p15.1 |
| ENSG00000172232 | AZU1 | azurocidin 1 | 19 | p13.3 |
| ENSG00000119686 | FLVCR2 | feline leukemia virus subgroup C cellular receptor family, member 2 | 14 | q24.3 |
| ENSG00000104765 | BNIP3L | BCL2/adenovirus E1B 19kDa interacting protein 3-like | 8 | p21.2 |
| ENSG00000197561 | ELANE | elastase, neutrophil expressed | 19 | p13.3 |
| ENSG00000185386 | MAPK11 | mitogen-activated protein kinase 11 | 22 | q13.33 |
| ENSG00000197766 | CFD | complement factor D (adipsin) | 19 | p13.3 |
| ENSG00000119699 | TGFB3 | transforming growth factor, beta 3 | 14 | q24.3 |
| ENSG00000006451 | RALA | v-ral simian leukemia viral oncogene homolog A (ras related) | 7 | p14.1 |
| ENSG00000121742 | GJB6 | gap junction protein, beta 6, 30kDa | 13 | q12.11 |
| ENSG00000134121 | CHL1 | cell adhesion molecule with homology to L1CAM (close homolog of L1) | 3 | p26.3 |
| ENSG00000120907 | ADRA1A | adrenoceptor alpha 1A | 8 | p21.2 |
| ENSG00000134115 | CNTN6 | contactin 6 | 3 | p26.3 |
| ENSG00000185652 | NTF3 | neurotrophin 3 | 12 | p13.31 |
| ENSG00000032742 | IFT88 | intraflagellar transport 88 homolog (Chlamydomonas) | 13 | q12.11 |
| ENSG00000122641 | INHBA | inhibin, beta A | 7 | p14.1 |
| ENSG00000144619 | CNTN4 | contactin 4 | 3 | p26.3 |
| ENSG00000033011 | ALG1 | asparagine-linked glycosylation 1, beta-1,4-mannosyltransferase homolog (S. cerevisiae) | 16 | p13.3 |
| ENSG00000110799 | VWF | von Willebrand factor | 12 | p13.31 |
| ENSG00000106571 | GLI3 | GLI family zinc finger 3 | 7 | p14.1 |
| ENSG00000174123 | TLR10 | toll-like receptor 10 | 4 | p14 |
| ENSG00000091181 | IL5RA | interleukin 5 receptor, alpha | 3 | p26.2 |
| ENSG00000106588 | PSMA2 | proteasome (prosome, macropain) subunit, alpha type, 2 | 7 | p14.1 |
| ENSG00000010278 | CD9 | CD9 molecule | 12 | p13.31 |
| ENSG00000174125 | TLR1 | toll-like receptor 1 | 4 | p14 |
| ENSG00000145526 | CDH18 | cadherin 18, type 2 | 5 | p14.3 |
| ENSG00000064687 | ABCA7 | ATP-binding cassette, sub-family A (ABC1), member 7 | 19 | p13.3 |
| ENSG00000174130 | TLR6 | toll-like receptor 6 | 4 | p14 |
| ENSG00000164543 | STK17A | serine/threonine kinase 17a | 7 | p13 |
| ENSG00000180448 | HMHA1 | histocompatibility (minor) HA-1 | 19 | p13.3 |
| ENSG00000154162 | CDH12 | cadherin 12, type 2 (N-cadherin 2) | 5 | p14.3 |
| ENSG00000172458 | IL17D | interleukin 17D | 13 | q12.11 |
| ENSG00000104228 | TRIM35 | tripartite motif containing 35 | 8 | p21.2 |
| ENSG00000067182 | TNFRSF1A | tumor necrosis factor receptor superfamily, member 1A | 12 | p13.31 |
| ENSG00000025708 | TYMP | thymidine phosphorylase | 22 | q13.33 |
| ENSG00000181031 | RPH3AL | rabphilin 3A-like (without C2 domains) | 17 | p13.3 |
| ENSG00000064932 | SBNO2 | strawberry notch homolog 2 (Drosophila) | 19 | p13.3 |
| ENSG00000120899 | PTK2B | PTK2B protein tyrosine kinase 2 beta | 8 | p21.2 |
| ENSG00000040731 | CDH10 | cadherin 10, type 2 (T2-cadherin) | 5 | p14.1 |
| ENSG00000111321 | LTBR | lymphotoxin beta receptor (TNFR superfamily, member 3) | 12 | p13.31 |
| ENSG00000128918 | ALDH1A2 | aldehyde dehydrogenase 1 family, member A2 | 15 | q21.3 |
| ENSG00000150457 | LATS2 | LATS, large tumor suppressor, homolog 2 (Drosophila) | 13 | q12.11 |
| ENSG00000139193 | CD27 | CD27 molecule | 12 | p13.31 |
| ENSG00000113100 | CDH9 | cadherin 9, type 2 (T1-cadherin) | 5 | p14.1 |
| ENSG00000136279 | DBNL | drebrin-like | 7 | p13 |
| ENSG00000139192 | TAPBPL | TAP binding protein-like | 12 | p13.31 |
| ENSG00000008735 | MAPK8IP2 | mitogen-activated protein kinase 8 interacting protein 2 | 22 | q13.33 |
| ENSG00000183454 | GRIN2A | glutamate receptor, ionotropic, N-methyl D-aspartate 2A | 16 | p13.2 |
| ENSG00000113361 | CDH6 | cadherin 6, type 2, K-cadherin (fetal kidney) | 5 | p13.3 |
| ENSG00000137845 | ADAM10 | ADAM metallopeptidase domain 10 | 15 | q21.3 |
| ENSG00000150995 | ITPR1 | inositol 1,4,5-trisphosphate receptor, type 1 | 3 | p26.1 |
| ENSG00000106624 | AEBP1 | AE binding protein 1 | 7 | p13 |
| ENSG00000021645 | NRXN3 | neurexin 3 | 14 | q24.3 |
| ENSG00000171861 | RNMTL1 | RNA methyltransferase like 1 | 17 | p13.3 |
| ENSG00000137776 | SLTM | SAFB-like, transcription modulator | 15 | q22.1 |
| ENSG00000120885 | CLU | clusterin | 8 | p21.1 |
| ENSG00000211448 | DIO2 | deiodinase, iodothyronine, type II | 14 | q31.1 |
| ENSG00000121897 | LIAS | lipoic acid synthetase | 4 | p14 |
| ENSG00000179583 | CIITA | class II, major histocompatibility complex, transactivator | 16 | p13.13 |
| ENSG00000133401 | PDZD2 | PDZ domain containing 2 | 5 | p13.3 |
| ENSG00000159842 | ABR | active BCR-related | 17 | p13.3 |
| ENSG00000157456 | CCNB2 | cyclin B2 | 15 | q22.2 |
| ENSG00000106636 | YKT6 | YKT6 v-SNARE homolog (S. cerevisiae) | 7 | p13 |
| ENSG00000157483 | MYO1E | myosin IE | 15 | q22.2 |
| ENSG00000185338 | SOCS1 | suppressor of cytokine signaling 1 | 16 | p13.13 |
| ENSG00000108953 | YWHAE | tyrosine 3-monooxygenase/tryptophan 5-monooxygenase activation protein, epsilon polypeptide | 17 | p13.3 |
| ENSG00000140299 | BNIP2 | BCL2/adenovirus E1B 19kDa interacting protein 2 | 15 | q22.2 |
| ENSG00000140022 | STON2 | stonin 2 | 14 | q31.1 |
| ENSG00000168079 | SCARA5 | scavenger receptor class A, member 5 (putative) | 8 | p21.1 |
| ENSG00000111653 | ING4 | inhibitor of growth family, member 4 | 12 | p13.31 |
| ENSG00000189067 | LITAF | lipopolysaccharide-induced TNF factor | 16 | p13.13 |
| ENSG00000185070 | FLRT2 | fibronectin leucine rich transmembrane protein 2 | 14 | q31.3 |
| ENSG00000140030 | GPR65 | G protein-coupled receptor 65 | 14 | q31.3 |
| ENSG00000168421 | RHOH | ras homolog family member H | 4 | p14 |
| ENSG00000071564 | TCF3 | transcription factor 3 (E2A immunoglobulin enhancer binding factors E12/E47) | 19 | p13.3 |
| ENSG00000180914 | OXTR | oxytocin receptor | 3 | p25.3 |
| ENSG00000069667 | RORA | RAR-related orphan receptor A | 15 | q22.2 |
| ENSG00000089693 | MLF2 | myeloid leukemia factor 2 | 12 | p13.31 |
| ENSG00000151835 | SACS | spastic ataxia of Charlevoix-Saguenay (sacsin) | 13 | q12.12 |
| ENSG00000089692 | LAG3 | lymphocyte-activation gene 3 | 12 | p13.31 |
| ENSG00000103342 | GSPT1 | G1 to S phase transition 1 | 16 | p13.13 |
| ENSG00000082196 | C1QTNF3 | C1q and tumor necrosis factor related protein 3 | 5 | p13.2 |
| ENSG00000010610 | CD4 | CD4 molecule | 12 | p13.31 |
| ENSG00000048462 | TNFRSF17 | tumor necrosis factor receptor superfamily, member 17 | 16 | p13.13 |
| ENSG00000127863 | TNFRSF19 | tumor necrosis factor receptor superfamily, member 19 | 13 | q12.12 |
| ENSG00000074660 | SCARF1 | scavenger receptor class F, member 1 | 17 | p13.3 |
| ENSG00000175595 | ERCC4 | excision repair cross-complementing rodent repair deficiency, complementation group 4 | 16 | p13.12 |
| ENSG00000198535 | C2CD4A | C2 calcium-dependent domain containing 4A | 15 | q22.2 |
| ENSG00000205502 | C2CD4B | C2 calcium-dependent domain containing 4B | 15 | q22.2 |
| ENSG00000171914 | TLN2 | talin 2 | 15 | q22.2 |
| ENSG00000165533 | TTC8 | tetratricopeptide repeat domain 8 | 14 | q31.3 |
| ENSG00000099875 | MKNK2 | MAP kinase interacting serine/threonine kinase 2 | 19 | p13.3 |
| ENSG00000140416 | TPM1 | tropomyosin 1 (alpha) | 15 | q22.2 |
| ENSG00000103429 | BFAR | bifunctional apoptosis regulator | 16 | p13.12 |
| ENSG00000146676 | PURB | purine-rich element binding protein B | 7 | p13 |
| ENSG00000205863 | C1QTNF9B | C1q and tumor necrosis factor related protein 9B | 13 | q12.12 |
| ENSG00000069764 | PLA2G10 | phospholipase A2, group X | 16 | p13.12 |
| ENSG00000197892 | KIF13B | kinesin family member 13B | 8 | p12 |
| ENSG00000185561 | TLCD2 | TLC domain containing 2 | 17 | p13.3 |
| ENSG00000136280 | CCM2 | cerebral cavernous malformation 2 | 7 | p13 |
| ENSG00000167711 | SERPINF2 | serpin peptidase inhibitor, clade F (alpha-2 antiplasmin, pigment epithelium derived factor), member 2 | 17 | p13.3 |
| ENSG00000136270 | TBRG4 | transforming growth factor beta regulator 4 | 7 | p13 |
| ENSG00000122679 | RAMP3 | receptor (G protein-coupled) activity modifying protein 3 | 7 | p13 |
| ENSG00000104660 | LEPROTL1 | leptin receptor overlapping transcript-like 1 | 8 | p12 |
| ENSG00000185088 | RPS27L | ribosomal protein S27-like | 15 | q22.2 |
| ENSG00000111679 | PTPN6 | protein tyrosine phosphatase, non-receptor type 6 | 12 | p13.31 |
| ENSG00000138613 | APH1B | anterior pharynx defective 1 homolog B (C. elegans) | 15 | q22.2 |
| ENSG00000100764 | PSMC1 | proteasome (prosome, macropain) 26S subunit, ATPase, 1 | 14 | q32.11 |
| ENSG00000185627 | PSMD13 | proteasome (prosome, macropain) 26S subunit, non-ATPase, 13 | 11 | p15.5 |
| ENSG00000113494 | PRLR | prolactin receptor | 5 | p13.2 |
| ENSG00000146674 | IGFBP3 | insulin-like growth factor binding protein 3 | 7 | p12.3 |
| ENSG00000104899 | AMH | anti-Mullerian hormone | 19 | p13.3 |
| ENSG00000182326 | C1S | complement component 1, s subcomponent | 12 | p13.31 |
| ENSG00000159403 | C1R | complement component 1, r subcomponent | 12 | p13.31 |
| ENSG00000139178 | C1RL | complement component 1, r subcomponent-like | 12 | p13.31 |
| ENSG00000158683 | PKD1L1 | polycystic kidney disease 1 like 1 | 7 | p12.3 |
| ENSG00000104695 | PPP2CB | protein phosphatase 2, catalytic subunit, beta isozyme | 8 | p12 |
| ENSG00000139182 | CLSTN3 | calsyntenin 3 | 12 | p13.31 |
| ENSG00000100784 | RPS6KA5 | ribosomal protein S6 kinase, 90kDa, polypeptide 5 | 14 | q32.11 |
| ENSG00000165392 | WRN | Werner syndrome, RecQ helicase-like | 8 | p12 |
| ENSG00000035664 | DAPK2 | death-associated protein kinase 2 | 15 | q22.31 |
| ENSG00000142102 | ATHL1 | ATH1, acid trehalase-like 1 (yeast) | 11 | p15.5 |
| ENSG00000167720 | SRR | serine racemase | 17 | p13.3 |
| ENSG00000168685 | IL7R | interleukin 7 receptor | 5 | p13.2 |
| ENSG00000099860 | GADD45B | growth arrest and DNA-damage-inducible, beta | 19 | p13.3 |
| ENSG00000206013 | IFITM5 | interferon induced transmembrane protein 5 | 11 | p15.5 |
| ENSG00000157168 | NRG1 | neuregulin 1 | 8 | p12 |
| ENSG00000185201 | IFITM2 | interferon induced transmembrane protein 2 | 11 | p15.5 |
| ENSG00000028528 | SNX1 | sorting nexin 1 | 15 | q22.31 |
| ENSG00000185885 | IFITM1 | interferon induced transmembrane protein 1 | 11 | p15.5 |
| ENSG00000177675 | CD163L1 | CD163 molecule-like 1 | 12 | p13.31 |
| ENSG00000142089 | IFITM3 | interferon induced transmembrane protein 3 | 11 | p15.5 |
| ENSG00000070444 | MNT | MNT, MAX dimerization protein | 17 | p13.3 |
| ENSG00000177575 | CD163 | CD163 molecule | 12 | p13.31 |
| ENSG00000172009 | THOP1 | thimet oligopeptidase 1 | 19 | p13.3 |
| ENSG00000184363 | PKP3 | plakophilin 3 | 11 | p15.5 |
| ENSG00000185811 | IKZF1 | IKAROS family zinc finger 1 (Ikaros) | 7 | p12.2 |
| ENSG00000007168 | PAFAH1B1 | platelet-activating factor acetylhydrolase 1b, regulatory subunit 1 (45kDa) | 17 | p13.3 |
| ENSG00000184344 | GDF3 | growth differentiation factor 3 | 12 | p13.31 |
| ENSG00000185187 | SIGIRR | single immunoglobulin and toll-interleukin 1 receptor (TIR) domain | 11 | p15.5 |
| ENSG00000140092 | FBLN5 | fibulin 5 | 14 | q32.12 |
| ENSG00000079215 | SLC1A3 | solute carrier family 1 (glial high affinity glutamate transporter), member 3 | 5 | p13.2 |
| ENSG00000184166 | OR1D2 | olfactory receptor, family 1, subfamily D, member 2 | 17 | p13.3 |
| ENSG00000187288 | CIDEC | cell death-inducing DFFA-like effector c | 3 | p25.3 |
| ENSG00000156687 | UNC5D | unc-5 homolog D (C. elegans) | 8 | p12 |
| ENSG00000104964 | AES | amino-terminal enhancer of split | 19 | p13.3 |
| ENSG00000164190 | NIPBL | Nipped-B homolog (Drosophila) | 5 | p13.2 |
| ENSG00000171860 | C3AR1 | complement component 3a receptor 1 | 12 | p13.31 |
| ENSG00000163701 | IL17RE | interleukin 17 receptor E | 3 | p25.3 |
| ENSG00000089818 | NECAP1 | NECAP endocytosis associated 1 | 12 | p13.31 |
| ENSG00000111729 | CLEC4A | C-type lectin domain family 4, member A | 12 | p13.31 |
| ENSG00000174775 | HRAS | v-Ha-ras Harvey rat sarcoma viral oncogene homolog | 11 | p15.5 |
| ENSG00000090487 | SPG21 | spastic paraplegia 21 (autosomal recessive, Mast syndrome) | 15 | q22.31 |
| ENSG00000146648 | EGFR | epidermal growth factor receptor | 7 | p11.2 |
| ENSG00000163702 | IL17RC | interleukin 17 receptor C | 3 | p25.3 |
| ENSG00000090470 | PDCD7 | programmed cell death 7 | 15 | q22.31 |
| ENSG00000100599 | RIN3 | Ras and Rab interactor 3 | 14 | q32.12 |
| ENSG00000103489 | XYLT1 | xylosyltransferase I | 16 | p12.3 |
| ENSG00000168621 | GDNF | glial cell derived neurotrophic factor | 5 | p13.2 |
| ENSG00000176826 | FKBP9L | FK506 binding protein 9-like | 7 | p11.2 |
| ENSG00000185507 | IRF7 | interferon regulatory factor 7 | 11 | p15.5 |
| ENSG00000074966 | TXK | TXK tyrosine kinase | 4 | p12 |
| ENSG00000188778 | ADRB3 | adrenoceptor beta 3 | 8 | p11.23 |
| ENSG00000174498 | IGDCC3 | immunoglobulin superfamily, DCC subclass, member 3 | 15 | q22.31 |
| ENSG00000111732 | AICDA | activation-induced cytidine deaminase | 12 | p13.31 |
| ENSG00000103742 | IGDCC4 | immunoglobulin superfamily, DCC subclass, member 4 | 15 | q22.31 |
| ENSG00000135605 | TEC | tec protein tyrosine kinase | 4 | p11 |
| ENSG00000099834 | CDHR5 | cadherin-related family member 5 | 11 | p15.5 |
| ENSG00000165943 | MOAP1 | modulator of apoptosis 1 | 14 | q32.12 |
| ENSG00000147465 | STAR | steroidogenic acute regulatory protein | 8 | p11.23 |
| ENSG00000156735 | BAG4 | BCL2-associated athanogene 4 | 8 | p11.23 |
| ENSG00000113594 | LIFR | leukemia inhibitory factor receptor alpha | 5 | p13.1 |
| ENSG00000003056 | M6PR | mannose-6-phosphate receptor (cation dependent) | 12 | p13.31 |
| ENSG00000069696 | DRD4 | dopamine receptor D4 | 11 | p15.5 |
| ENSG00000167186 | COQ7 | coenzyme Q7 homolog, ubiquinone (yeast) | 16 | p12.3 |
| ENSG00000144554 | FANCD2 | Fanconi anemia, complementation group D2 | 3 | p25.3 |
| ENSG00000139187 | KLRG1 | killer cell lectin-like receptor subfamily G, member 1 | 12 | p13.31 |
| ENSG00000145623 | OSMR | oncostatin M receptor | 5 | p13.1 |
| ENSG00000175899 | A2M | alpha-2-macroglobulin | 12 | p13.31 |
| ENSG00000103769 | RAB11A | RAB11A, member RAS oncogene family | 15 | q22.31 |
| ENSG00000082074 | FYB | FYN binding protein | 5 | p13.1 |
| ENSG00000083457 | ITGAE | integrin, alpha E (antigen CD103, human mucosal lymphocyte antigen 1; alpha polypeptide) | 17 | p13.2 |
| ENSG00000077782 | FGFR1 | fibroblast growth factor receptor 1 | 8 | p11.23 |
| ENSG00000134086 | VHL | von Hippel-Lindau tumor suppressor, E3 ubiquitin protein ligase | 3 | p25.3 |
| ENSG00000103540 | CCP110 | centriolar coiled coil protein 110kDa | 16 | p12.3 |
| ENSG00000177602 | GSG2 | germ cell associated 2 (haspin) | 17 | p13.2 |
| ENSG00000113600 | C9 | complement component 9 | 5 | p13.1 |
| ENSG00000134070 | IRAK2 | interleukin-1 receptor-associated kinase 2 | 3 | p25.3 |
| ENSG00000165948 | IFI27L1 | interferon, alpha-inducible protein 27-like 1 | 14 | q32.12 |
| ENSG00000165949 | IFI27 | interferon, alpha-inducible protein 27 | 14 | q32.12 |
| ENSG00000157017 | GHRL | ghrelin/obestatin prepropeptide | 3 | p25.3 |
| ENSG00000119632 | IFI27L2 | interferon, alpha-inducible protein 27-like 2 | 14 | q32.12 |
| ENSG00000169032 | MAP2K1 | mitogen-activated protein kinase kinase 1 | 15 | q22.31 |
| ENSG00000077009 | ITGB1BP3 | nicotinamide riboside kinase 2 | 19 | p13.3 |
| ENSG00000110848 | CD69 | CD69 molecule | 12 | p13.31 |
| ENSG00000177595 | PIDD | p53-induced death domain protein | 11 | p15.5 |
| ENSG00000167657 | DAPK3 | death-associated protein kinase 3 | 19 | p13.3 |
| ENSG00000171522 | PTGER4 | prostaglandin E receptor 4 (subtype EP4) | 5 | p13.1 |
| ENSG00000150045 | KLRF1 | killer cell lectin-like receptor subfamily F, member 1 | 12 | p13.31 |
| ENSG00000197249 | SERPINA1 | serpin peptidase inhibitor, clade A (alpha-1 antiproteinase, antitrypsin), member 1 | 14 | q32.13 |
| ENSG00000108405 | P2RX1 | purinergic receptor P2X, ligand-gated ion channel, 1 | 17 | p13.2 |
| ENSG00000174442 | ZWILCH | Zwilch, kinetochore associated, homolog (Drosophila) | 15 | q22.31 |
| ENSG00000132356 | PRKAA1 | protein kinase, AMP-activated, alpha 1 catalytic subunit | 5 | p13.1 |
| ENSG00000169344 | UMOD | uromodulin | 16 | p12.3 |
| ENSG00000137834 | SMAD6 | SMAD family member 6 | 15 | q22.31 |
| ENSG00000166949 | SMAD3 | SMAD family member 3 | 15 | q22.33 |
| ENSG00000168615 | ADAM9 | ADAM metallopeptidase domain 9 | 8 | p11.22 |
| ENSG00000184178 | SCFD2 | sec1 family domain containing 2 | 4 | q12 |
| ENSG00000177697 | CD151 | CD151 molecule (Raph blood group) | 11 | p15.5 |
| ENSG00000196136 | SERPINA3 | serpin peptidase inhibitor, clade A (alpha-1 antiproteinase, antitrypsin), member 3 | 14 | q32.13 |
| ENSG00000172243 | CLEC7A | C-type lectin domain family 7, member A | 12 | p13.2 |
| ENSG00000132357 | CARD6 | caspase recruitment domain family, member 6 | 5 | p13.1 |
| ENSG00000112936 | C7 | complement component 7 | 5 | p13.1 |
| ENSG00000105246 | EBI3 | Epstein-Barr virus induced 3 | 19 | p13.3 |
| ENSG00000039537 | C6 | complement component 6 | 5 | p13.1 |
| ENSG00000173391 | OLR1 | oxidized low density lipoprotein (lectin-like) receptor 1 | 12 | p13.2 |
| ENSG00000104755 | ADAM2 | ADAM metallopeptidase domain 2 | 8 | p11.22 |
| ENSG00000183020 | AP2A2 | adaptor-related protein complex 2, alpha 2 subunit | 11 | p15.5 |
| ENSG00000196639 | HRH1 | histamine receptor H1 | 3 | p25.3 |
| ENSG00000167664 | TMIGD2 | transmembrane and immunoglobulin domain containing 2 | 19 | p13.3 |
| ENSG00000154710 | RABGEF1 | RAB guanine nucleotide exchange factor (GEF) 1 | 7 | q11.21 |
| ENSG00000131203 | IDO1 | indoleamine 2,3-dioxygenase 1 | 8 | p11.21 |
| ENSG00000187621 | TCL6 | T-cell leukemia/lymphoma 6 (non-protein coding) | 14 | q32.13 |
| ENSG00000176907 | C8orf4 | chromosome 8 open reading frame 4 | 8 | p11.21 |
| ENSG00000134853 | PDGFRA | platelet-derived growth factor receptor, alpha polypeptide | 4 | q12 |
| ENSG00000188215 | DCUN1D3 | DCN1, defective in cullin neddylation 1, domain containing 3 (S. cerevisiae) | 16 | p12.3 |
| ENSG00000141985 | SH3GL1 | SH3-domain GRB2-like 1 | 19 | p13.3 |
| ENSG00000104332 | SFRP1 | secreted frizzled-related protein 1 | 8 | p11.21 |
| ENSG00000183542 | KLRC4 | killer cell lectin-like receptor subfamily C, member 4 | 12 | p13.2 |
| ENSG00000126524 | SBDS | Shwachman-Bodian-Diamond syndrome | 7 | q11.21 |
| ENSG00000117983 | MUC5B | mucin 5B, oligomeric mucus/gel-forming | 11 | p15.5 |
| ENSG00000157150 | TIMP4 | TIMP metallopeptidase inhibitor 4 | 3 | p25.2 |
| ENSG00000112964 | GHR | growth hormone receptor | 5 | p13.1 |
| ENSG00000100721 | TCL1A | T-cell leukemia/lymphoma 1A | 14 | q32.13 |
| ENSG00000205810 | KLRC3 | killer cell lectin-like receptor subfamily C, member 3 | 12 | p13.2 |
| ENSG00000169018 | FEM1B | fem-1 homolog b (C. elegans) | 15 | q23 |
| ENSG00000132170 | PPARG | peroxisome proliferator-activated receptor gamma | 3 | p25.2 |
| ENSG00000137809 | ITGA11 | integrin, alpha 11 | 15 | q23 |
| ENSG00000157404 | KIT | v-kit Hardy-Zuckerman 4 feline sarcoma viral oncogene homolog | 4 | q12 |
| ENSG00000168398 | BDKRB2 | bradykinin receptor B2 | 14 | q32.2 |
| ENSG00000205809 | KLRC2 | killer cell lectin-like receptor subfamily C, member 2 | 12 | p13.2 |
| ENSG00000100739 | BDKRB1 | bradykinin receptor B1 | 14 | q32.2 |
| ENSG00000171236 | LRG1 | leucine-rich alpha-2-glycoprotein 1 | 19 | p13.3 |
| ENSG00000029534 | ANK1 | ankyrin 1, erythrocytic | 8 | p11.21 |
| ENSG00000078902 | TOLLIP | toll interacting protein | 11 | p15.5 |
| ENSG00000128052 | KDR | kinase insert domain receptor (a type III receptor tyrosine kinase) | 4 | q12 |
| ENSG00000185361 | TNFAIP8L1 | tumor necrosis factor, alpha-induced protein 8-like 1 | 19 | p13.3 |
| ENSG00000137808 | NOX5 | NADPH oxidase 5 isoform 3 | 15 | q23 |
| ENSG00000141480 | ARRB2 | arrestin, beta 2 | 17 | p13.2 |
| ENSG00000151882 | CCL28 | chemokine (C-C motif) ligand 28 | 5 | p12 |
| ENSG00000132155 | RAF1 | v-raf-1 murine leukemia viral oncogene homolog 1 | 3 | p25.2 |
| ENSG00000060138 | CSDA | cold shock domain protein A | 12 | p13.2 |
| ENSG00000140749 | IGSF6 | immunoglobulin superfamily, member 6 | 16 | p12.2 |
| ENSG00000127152 | BCL11B | B-cell CLL/lymphoma 11B (zinc finger protein) | 14 | q32.2 |
| ENSG00000161921 | CXCL16 | chemokine (C-X-C motif) ligand 16 | 17 | p13.2 |
| ENSG00000127666 | TICAM1 | toll-like receptor adaptor molecule 1 | 19 | p13.3 |
| ENSG00000137831 | UACA | uveal autoantigen with coiled-coil domains and ankyrin repeats | 15 | q23 |
| ENSG00000182487 | NCF1B | neutrophil cytosolic factor 1B pseudogene | 7 | q11.23 |
| ENSG00000117984 | CTSD | cathepsin D | 11 | p15.5 |
| ENSG00000142507 | PSMB6 | proteasome (prosome, macropain) subunit, beta type, 6 | 17 | p13.2 |
| ENSG00000083168 | KAT6A | K(lysine) acetyltransferase 6A | 8 | p11.21 |
| ENSG00000090989 | EXOC1 | exocyst complex component 1 | 4 | q12 |
| ENSG00000129219 | PLD2 | phospholipase D2 | 17 | p13.2 |
| ENSG00000105426 | PTPRS | protein tyrosine phosphatase, receptor type, S | 19 | p13.3 |
| ENSG00000104368 | PLAT | plasminogen activator, tissue | 8 | p11.21 |
| ENSG00000077800 | FKBP6 | FK506 binding protein 6, 36kDa | 7 | q11.23 |
| ENSG00000154764 | WNT7A | wingless-type MMTV integration site family, member 7A | 3 | p25.1 |
| ENSG00000070193 | FGF10 | fibroblast growth factor 10 | 5 | p12 |
| ENSG00000122025 | FLT3 | fms-related tyrosine kinase 3 | 13 | q12.2 |
| ENSG00000112996 | MRPS30 | mitochondrial ribosomal protein S30 | 5 | p12 |
| ENSG00000106635 | BCL7B | B-cell CLL/lymphoma 7B | 7 | q11.23 |
| ENSG00000141503 | MINK1 | misshapen-like kinase 1 | 17 | p13.2 |
| ENSG00000130598 | TNNI2 | troponin I type 2 (skeletal, fast) | 11 | p15.5 |
| ENSG00000104365 | IKBKB | inhibitor of kappa light polypeptide gene enhancer in B-cells, kinase beta | 8 | p11.21 |
| ENSG00000154767 | XPC | xeroderma pigmentosum, complementation group C | 3 | p25.1 |
| ENSG00000121380 | BCL2L14 | BCL2-like 14 (apoptosis facilitator) | 12 | p13.2 |
| ENSG00000009950 | MLXIPL | MLX interacting protein-like | 7 | q11.23 |
| ENSG00000170571 | EMB | embigin | 5 | q11.1 |
| ENSG00000070018 | LRP6 | low density lipoprotein receptor-related protein 6 | 12 | p13.2 |
| ENSG00000070501 | POLB | polymerase (DNA directed), beta | 8 | p11.21 |
| ENSG00000130592 | LSP1 | lymphocyte-specific protein 1 | 11 | p15.5 |
| ENSG00000067225 | PKM2 | pyruvate kinase, muscle | 15 | q23 |
| ENSG00000185245 | GP1BA | glycoprotein Ib (platelet), alpha polypeptide | 17 | p13.2 |
| ENSG00000132963 | POMP | proteasome maturation protein | 13 | q12.3 |
| ENSG00000106089 | STX1A | syntaxin 1A (brain) | 7 | q11.23 |
| ENSG00000111276 | CDKN1B | cyclin-dependent kinase inhibitor 1B (p27, Kip1) | 12 | p13.1 |
| ENSG00000164171 | ITGA2 | integrin, alpha 2 (CD49B, alpha 2 subunit of VLA-2 receptor) | 5 | q11.2 |
| ENSG00000130595 | TNNT3 | troponin T type 3 (skeletal, fast) | 11 | p15.5 |
| ENSG00000131931 | THAP1 | THAP domain containing, apoptosis associated protein 1 | 8 | p11.21 |
| ENSG00000165215 | CLDN3 | claudin 3 | 7 | q11.23 |
| ENSG00000189143 | CLDN4 | claudin 4 | 7 | q11.23 |
| ENSG00000078304 | PPP2R5C | protein phosphatase 2, regulatory subunit B', gamma | 14 | q32.31 |
| ENSG00000168522 | FNTA | farnesyltransferase, CAAX box, alpha | 8 | p11.21 |
| ENSG00000163453 | IGFBP7 | insulin-like growth factor binding protein 7 | 4 | q12 |
| ENSG00000171119 | NRTN | neurturin | 19 | p13.3 |
| ENSG00000113088 | GZMK | granzyme K (granzyme 3; tryptase II) | 5 | q11.2 |
| ENSG00000134398 | ERN2 | endoplasmic reticulum to nucleus signaling 2 | 16 | p12.2 |
| ENSG00000167244 | IGF2 | insulin-like growth factor 2 (somatomedin A) | 11 | p15.5 |
| ENSG00000145649 | GZMA | granzyme A (granzyme 1, cytotoxic T-lymphocyte-associated serine esterase 3) | 5 | q11.2 |
| ENSG00000140463 | BBS4 | Bardet-Biedl syndrome 4 | 15 | q24.1 |
| ENSG00000171124 | FUT3 | fucosyltransferase 3 (galactoside 3(4)-L-fucosyltransferase, Lewis blood group) | 19 | p13.3 |
| ENSG00000166501 | PRKCB | protein kinase C, beta | 16 | p12.2 |
| ENSG00000067141 | NEO1 | neogenin 1 | 15 | q24.1 |
| ENSG00000086730 | LAT2 | linker for activation of T cells family, member 2 | 7 | q11.23 |
| ENSG00000087903 | RFX2 | regulatory factor X, 2 (influences HLA class II expression) | 19 | p13.3 |
| ENSG00000189403 | HMGB1 | high mobility group box 1 | 13 | q12.3 |
| ENSG00000156642 | NPTN | neuroplastin | 15 | q24.1 |
| ENSG00000067113 | PPAP2A | phosphatidic acid phosphatase type 2A | 5 | q11.2 |
| ENSG00000130382 | MLLT1 | myeloid/lymphoid or mixed-lineage leukemia (trithorax homolog, Drosophila); translocated to, 1 | 19 | p13.3 |
| ENSG00000103855 | CD276 | CD276 molecule | 15 | q24.1 |
| ENSG00000131323 | TRAF3 | TNF receptor-associated factor 3 | 14 | q32.32 |
| ENSG00000111339 | ART4 | ADP-ribosyltransferase 4 (Dombrock blood group) | 12 | p12.3 |
| ENSG00000029725 | RABEP1 | rabaptin, RAB GTPase binding effector protein 1 | 17 | p13.2 |
| ENSG00000158865 | SLC5A11 | solute carrier family 5 (sodium/glucose cotransporter), member 11 | 16 | p12.1 |
| ENSG00000099869 | IGF2-AS | IGF2 antisense RNA | 11 | p15.5 |
| ENSG00000111348 | ARHGDIB | Rho GDP dissociation inhibitor (GDI) beta | 12 | p12.3 |
| ENSG00000140750 | ARHGAP17 | Rho GTPase activating protein 17 | 16 | p12.1 |
| ENSG00000164509 | IL31RA | interleukin 31 receptor A | 5 | q11.2 |
| ENSG00000129965 | INS-IGF2 | INS-IGF2 readthrough | 11 | p15.5 |
| ENSG00000185215 | TNFAIP2 | tumor necrosis factor, alpha-induced protein 2 | 14 | q32.32 |
| ENSG00000140464 | PML | promyelocytic leukemia | 15 | q24.1 |
| ENSG00000023287 | RB1CC1 | RB1-inducible coiled-coil 1 | 8 | q11.23 |
| ENSG00000108561 | C1QBP | complement component 1, q subcomponent binding protein | 17 | p13.2 |
| ENSG00000134352 | IL6ST | interleukin 6 signal transducer (gp130, oncostatin M receptor) | 5 | q11.2 |
| ENSG00000075413 | MARK3 | MAP/microtubule affinity-regulating kinase 3 | 14 | q32.32 |
| ENSG00000182568 | SATB1 | SATB homeobox 1 | 3 | p24.3 |
| ENSG00000158517 | NCF1 | neutrophil cytosolic factor 1 | 7 | q11.23 |
| ENSG00000183960 | KCNH8 | potassium voltage-gated channel, subfamily H (eag-related), member 8 | 3 | p24.3 |
| ENSG00000047249 | ATP6V1H | ATPase, H+ transporting, lysosomal 50/57kDa, V1 subunit H | 8 | q11.23 |
| ENSG00000125657 | TNFSF9 | tumor necrosis factor (ligand) superfamily, member 9 | 19 | p13.3 |
| ENSG00000125726 | CD70 | CD70 molecule | 19 | p13.3 |
| ENSG00000144566 | RAB5A | RAB5A, member RAS oncogene family | 3 | p24.3 |
| ENSG00000125735 | TNFSF14 | tumor necrosis factor (ligand) superfamily, member 14 | 19 | p13.3 |
| ENSG00000166170 | BAG5 | BCL2-associated athanogene 5 | 14 | q32.33 |
| ENSG00000008394 | MGST1 | microsomal glutathione S-transferase 1 | 12 | p12.3 |
| ENSG00000125730 | C3 | complement component 3 | 19 | p13.3 |
| ENSG00000077238 | IL4R | interleukin 4 receptor | 16 | p12.1 |
| ENSG00000167178 | ISLR2 | immunoglobulin superfamily containing leucine-rich repeat 2 | 15 | q24.1 |
| ENSG00000091592 | NLRP1 | NLR family, pyrin domain containing 1 | 17 | p13.2 |
| ENSG00000129009 | ISLR | immunoglobulin superfamily containing leucine-rich repeat | 15 | q24.1 |
| ENSG00000103522 | IL21R | interleukin 21 receptor | 16 | p12.1 |
| ENSG00000125733 | TRIP10 | thyroid hormone receptor interactor 10 | 19 | p13.3 |
| ENSG00000129221 | AIPL1 | aryl hydrocarbon receptor interacting protein-like 1 | 17 | p13.2 |
| ENSG00000095015 | MAP3K1 | mitogen-activated protein kinase kinase kinase 1, E3 ubiquitin protein ligase | 5 | q11.2 |
| ENSG00000133105 | RXFP2 | relaxin/insulin-like family peptide receptor 2 | 13 | q13.1 |
| ENSG00000141968 | VAV1 | vav 1 guanine nucleotide exchange factor | 19 | p13.3 |
| ENSG00000126215 | XRCC3 | X-ray repair complementing defective repair in Chinese hamster cells 3 | 14 | q32.33 |
| ENSG00000206579 | XKR4 | XK, Kell blood group complex subunit-related family, member 4 | 8 | q12.1 |
| ENSG00000138623 | SEMA7A | semaphorin 7A, GPI membrane anchor (John Milton Hagen blood group) | 15 | q24.1 |
| ENSG00000138629 | UBL7 | ubiquitin-like 7 (bone marrow stromal cell-derived) | 15 | q24.1 |
| ENSG00000088808 | PPP1R13B | protein phosphatase 1, regulatory subunit 13B | 14 | q32.33 |
| ENSG00000174837 | EMR1 | egf-like module containing, mucin-like, hormone receptor-like 1 | 19 | p13.3 |
| ENSG00000172572 | PDE3A | phosphodiesterase 3A, cGMP-inhibited | 12 | p12.2 |
| ENSG00000151090 | THRB | thyroid hormone receptor, beta | 3 | p24.2 |
| ENSG00000129235 | TXNDC17 | thioredoxin domain containing 17 | 17 | p13.1 |
| ENSG00000146722 | LOC541473 | FK506 binding protein 6, 36kDa pseudogene (LOC541473), non-coding RNA | 7 | q11.23 |
| ENSG00000188603 | CLN3 | ceroid-lipofuscinosis, neuronal 3 | 16 | p12.1 |
| ENSG00000110651 | CD81 | CD81 molecule | 11 | p15.5 |
| ENSG00000140465 | CYP1A1 | cytochrome P450, family 1, subfamily A, polypeptide 1 | 15 | q24.1 |
| ENSG00000077092 | RARB | retinoic acid receptor, beta | 3 | p24.2 |
| ENSG00000171105 | INSR | insulin receptor | 19 | p13.2 |
| ENSG00000187689 | AMTN | amelotin | 4 | q13.3 |
| ENSG00000178522 | AMBN | ameloblastin (enamel matrix protein) | 4 | q13.3 |
| ENSG00000175857 | GAPT | GRB2-binding adaptor protein, transmembrane | 5 | q11.2 |
| ENSG00000152932 | RAB3C | RAB3C, member RAS oncogene family | 5 | q11.2 |
| ENSG00000077097 | TOP2B | topoisomerase (DNA) II beta 180kDa | 3 | p24.2 |
| ENSG00000132530 | XAF1 | XIAP associated factor 1 | 17 | p13.1 |
| ENSG00000132465 | IGJ | immunoglobulin J polypeptide, linker protein for immunoglobulin alpha and mu polypeptides | 4 | q13.3 |
| ENSG00000127946 | HIP1 | huntingtin interacting protein 1 | 7 | q11.23 |
| ENSG00000184990 | SIVA1 | SIVA1, apoptosis-inducing factor | 14 | q32.33 |
| ENSG00000104880 | ARHGEF18 | Rho/Rac guanine nucleotide exchange factor (GEF) 18 | 19 | p13.2 |
| ENSG00000139618 | BRCA2 | breast cancer 2, early onset | 13 | q13.1 |
| ENSG00000137575 | SDCBP | syndecan binding protein (syntenin) | 8 | q12.1 |
| ENSG00000142208 | AKT1 | v-akt murine thymoma viral oncogene homolog 1 | 14 | q32.33 |
| ENSG00000006606 | CCL26 | chemokine (C-C motif) ligand 26 | 7 | q11.23 |
| ENSG00000121351 | IAPP | islet amyloid polypeptide | 12 | p12.1 |
| ENSG00000106178 | CCL24 | chemokine (C-C motif) ligand 24 | 7 | q11.23 |
| ENSG00000198846 | TOX | thymocyte selection-associated high mobility group box | 8 | q12.1 |
| ENSG00000197272 | IL27 | interleukin 27 | 16 | p11.2 |
| ENSG00000108839 | ALOX12 | arachidonate 12-lipoxygenase | 17 | p13.1 |
| ENSG00000176046 | NUPR1 | nuclear protein, transcriptional regulator, 1 | 16 | p11.2 |
| ENSG00000049167 | ERCC8 | excision repair cross-complementing rodent repair deficiency, complementation group 8 | 5 | q12.1 |
| ENSG00000171316 | CHD7 | chromodomain helicase DNA binding protein 7 | 8 | q12.2 |
| ENSG00000198794 | SCAMP5 | secretory carrier membrane protein 5 | 15 | q24.2 |
| ENSG00000161940 | BCL6B | B-cell CLL/lymphoma 6, member B | 17 | p13.1 |
| ENSG00000184916 | JAG2 | jagged 2 | 14 | q32.33 |
| ENSG00000076826 | CAMSAP3 | calmodulin regulated spectrin-associated protein family, member 3 | 19 | p13.2 |
| ENSG00000121361 | KCNJ8 | potassium inwardly-rectifying channel, subfamily J, member 8 | 12 | p12.1 |
| ENSG00000106211 | HSPB1 | heat shock 27kDa protein 1 | 7 | q11.23 |
| ENSG00000163508 | EOMES | eomesodermin | 3 | p24.1 |
| ENSG00000076944 | STXBP2 | syntaxin binding protein 2 | 19 | p13.2 |
| ENSG00000170027 | YWHAG | tyrosine 3-monooxygenase/tryptophan 5-monooxygenase activation protein, gamma polypeptide | 7 | q11.23 |
| ENSG00000132514 | CLEC10A | C-type lectin domain family 10, member A | 17 | p13.1 |
| ENSG00000161944 | ASGR2 | asialoglycoprotein receptor 2 | 17 | p13.1 |
| ENSG00000104918 | RETN | resistin | 19 | p13.2 |
| ENSG00000104921 | FCER2 | Fc fragment of IgE, low affinity II, receptor for (CD23) | 19 | p13.2 |
| ENSG00000129757 | CDKN1C | cyclin-dependent kinase inhibitor 1C (p57, Kip2) | 11 | p15.4 |
| ENSG00000111726 | CMAS | cytidine monophosphate N-acetylneuraminic acid synthetase | 12 | p12.1 |
| ENSG00000163513 | TGFBR2 | transforming growth factor, beta receptor II (70/80kDa) | 3 | p24.1 |
| ENSG00000141505 | ASGR1 | asialoglycoprotein receptor 1 | 17 | p13.1 |
| ENSG00000179364 | PACS2 | phosphofurin acidic cluster sorting protein 2 | 14 | q32.33 |
| ENSG00000169375 | SIN3A | SIN3 transcription regulator homolog A (yeast) | 15 | q24.2 |
| ENSG00000090659 | CD209 | CD209 molecule | 19 | p13.2 |
| ENSG00000163631 | ALB | albumin | 4 | q13.3 |
| ENSG00000196296 | ATP2A1 | ATPase, Ca++ transporting, cardiac muscle, fast twitch 1 | 16 | p11.2 |
| ENSG00000104938 | CLEC4M | C-type lectin domain family 4, member M | 19 | p13.2 |
| ENSG00000170293 | CMTM8 | CKLF-like MARVEL transmembrane domain containing 8 | 3 | p22.3 |
| ENSG00000153551 | CMTM7 | CKLF-like MARVEL transmembrane domain containing 7 | 3 | p22.3 |
| ENSG00000177548 | RABEP2 | rabaptin, RAB GTPase binding effector protein 2 | 16 | p11.2 |
| ENSG00000091317 | CMTM6 | CKLF-like MARVEL transmembrane domain containing 6 | 3 | p22.3 |
| ENSG00000118308 | LRMP | lymphoid-restricted membrane protein | 12 | p12.1 |
| ENSG00000181649 | PHLDA2 | pleckstrin homology-like domain, family A, member 2 | 11 | p15.4 |
| ENSG00000147571 | CRH | corticotropin releasing hormone | 8 | q13.1 |
| ENSG00000177455 | CD19 | CD19 molecule | 16 | p11.2 |
| ENSG00000176953 | NFATC2IP | nuclear factor of activated T-cells, cytoplasmic, calcineurin-dependent 2 interacting protein | 16 | p11.2 |
| ENSG00000169435 | RASSF6 | Ras association (RalGDS/AF-6) domain family member 6 | 4 | q13.3 |
| ENSG00000133703 | KRAS | v-Ki-ras2 Kirsten rat sarcoma viral oncogene homolog | 12 | p12.1 |
| ENSG00000183813 | CCR4 | chemokine (C-C motif) receptor 4 | 3 | p22.3 |
| ENSG00000169429 | IL8 | interleukin 8 | 4 | q13.3 |
| ENSG00000104205 | SGK3 | serum/glucocorticoid regulated kinase family, member 3 | 8 | q13.1 |
| ENSG00000124875 | CXCL6 | chemokine (C-X-C motif) ligand 6 | 4 | q13.3 |
| ENSG00000135218 | CD36 | CD36 molecule (thrombospondin receptor) | 7 | q21.11 |
| ENSG00000109272 | PF4V1 | platelet factor 4 variant 1 | 4 | q13.3 |
| ENSG00000181885 | CLDN7 | claudin 7 | 17 | p13.1 |
| ENSG00000173705 | SUSD5 | sushi domain containing 5 | 3 | p22.3 |
| ENSG00000163739 | CXCL1 | chemokine (C-X-C motif) ligand 1 (melanoma growth stimulating activity, alpha) | 4 | q13.3 |
| ENSG00000075223 | SEMA3C | sema domain, immunoglobulin domain (Ig), short basic domain, secreted, (semaphorin) 3C | 7 | q21.11 |
| ENSG00000163737 | PF4 | platelet factor 4 | 4 | q13.3 |
| ENSG00000123096 | SSPN | sarcospan | 12 | p12.1 |
| ENSG00000140368 | PSTPIP1 | proline-serine-threonine phosphatase interacting protein 1 | 15 | q24.3 |
| ENSG00000163736 | PPBP | pro-platelet basic protein (chemokine (C-X-C motif) ligand 7) | 4 | q13.3 |
| ENSG00000153560 | UBP1 | upstream binding protein 1 (LBP-1a) | 3 | p22.3 |
| ENSG00000181856 | SLC2A4 | solute carrier family 2 (facilitated glucose transporter), member 4 | 17 | p13.1 |
| ENSG00000019991 | HGF | hepatocyte growth factor (hepapoietin A; scatter factor) | 7 | q21.11 |
| ENSG00000123104 | ITPR2 | inositol 1,4,5-trisphosphate receptor, type 2 | 12 | p11.23 |
| ENSG00000163735 | CXCL5 | chemokine (C-X-C motif) ligand 5 | 4 | q13.3 |
| ENSG00000131142 | CCL25 | chemokine (C-C motif) ligand 25 | 19 | p13.2 |
| ENSG00000163734 | CXCL3 | chemokine (C-X-C motif) ligand 3 | 4 | q13.3 |
| ENSG00000081041 | CXCL2 | chemokine (C-X-C motif) ligand 2 | 4 | q13.3 |
| ENSG00000132507 | EIF5A | eukaryotic translation initiation factor 5A | 17 | p13.1 |
| ENSG00000066777 | ARFGEF1 | ADP-ribosylation factor guanine nucleotide-exchange factor 1 (brefeldin A-inhibited) | 8 | q13.2 |
| ENSG00000124882 | EREG | epiregulin | 4 | q13.3 |
| ENSG00000170248 | PDCD6IP | programmed cell death 6 interacting protein | 3 | p22.3 |
| ENSG00000109321 | AREG | amphiregulin | 4 | q13.3 |
| ENSG00000112851 | ERBB2IP | erbb2 interacting protein | 5 | q12.3 |
| ENSG00000186472 | PCLO | piccolo (presynaptic cytomatrix protein) | 7 | q21.11 |
| ENSG00000136425 | CIB2 | calcium and integrin binding family member 2 | 15 | q25.1 |
| ENSG00000211455 | STK38L | serine/threonine kinase 38 like | 12 | p11.23 |
| ENSG00000174808 | BTC | betacellulin | 4 | q13.3 |
| ENSG00000197471 | SPN | sialophorin | 16 | p11.2 |
| ENSG00000170381 | SEMA3E | sema domain, immunoglobulin domain (Ig), short basic domain, secreted, (semaphorin) 3E | 7 | q21.11 |
| ENSG00000167775 | CD320 | CD320 molecule | 19 | p13.2 |
| ENSG00000075213 | SEMA3A | sema domain, immunoglobulin domain (Ig), short basic domain, secreted, (semaphorin) 3A | 7 | q21.11 |
| ENSG00000110841 | PPFIBP1 | PTPRF interacting protein, binding protein 1 (liprin beta 1) | 12 | p11.23 |
| ENSG00000076242 | MLH1 | mutL homolog 1, colon cancer, nonpolyposis type 2 (E. coli) | 3 | p22.2 |
| ENSG00000153993 | SEMA3D | sema domain, immunoglobulin domain (Ig), short basic domain, secreted, (semaphorin) 3D | 7 | q21.11 |
| ENSG00000120693 | SMAD9 | SMAD family member 9 | 13 | q13.3 |
| ENSG00000148985 | PGAP2 | post-GPI attachment to proteins 2 | 11 | p15.4 |
| ENSG00000167772 | ANGPTL4 | angiopoietin-like 4 | 19 | p13.2 |
| ENSG00000137573 | SULF1 | sulfatase 1 | 8 | q13.2 |
| ENSG00000099785 | MARCH2 | membrane-associated ring finger (C3HC4) 2, E3 ubiquitin protein ligase | 19 | p13.2 |
| ENSG00000041357 | PSMA4 | proteasome (prosome, macropain) subunit, alpha type, 4 | 15 | q25.1 |
| ENSG00000169992 | NLGN2 | neuroligin 2 | 17 | p13.1 |
| ENSG00000133246 | PRAM1 | PML-RARA regulated adaptor molecule 1 | 19 | p13.2 |
| ENSG00000134061 | CD180 | CD180 molecule | 5 | q12.3 |
| ENSG00000133110 | POSTN | periostin, osteoblast specific factor | 13 | q13.3 |
| ENSG00000181284 | TMEM102 | transmembrane protein 102 | 17 | p13.1 |
| ENSG00000104313 | EYA1 | eyes absent homolog 1 (Drosophila) | 8 | q13.3 |
| ENSG00000138755 | CXCL9 | chemokine (C-X-C motif) ligand 9 | 4 | q21.1 |
| ENSG00000144668 | ITGA9 | integrin, alpha 9 | 3 | p22.2 |
| ENSG00000149930 | TAOK2 | TAO kinase 2 | 16 | p11.2 |
| ENSG00000169245 | CXCL10 | chemokine (C-X-C motif) ligand 10 | 4 | q21.1 |
| ENSG00000169248 | CXCL11 | chemokine (C-X-C motif) ligand 11 | 4 | q21.1 |
| ENSG00000103811 | CTSH | cathepsin H | 15 | q25.1 |
| ENSG00000008277 | ADAM22 | ADAM metallopeptidase domain 22 | 7 | q21.12 |
| ENSG00000058335 | RASGRF1 | Ras protein-specific guanine nucleotide-releasing factor 1 | 15 | q25.1 |
| ENSG00000147601 | TERF1 | telomeric repeat binding factor (NIMA-interacting) 1 | 8 | q21.11 |
| ENSG00000138760 | SCARB2 | scavenger receptor class B, member 2 | 4 | q21.1 |
| ENSG00000181143 | MUC16 | mucin 16, cell surface associated | 19 | p13.2 |
| ENSG00000149927 | DOC2A | double C2-like domains, alpha | 16 | p11.2 |
| ENSG00000161955 | TNFSF13 | tumor necrosis factor (ligand) superfamily, member 13 | 17 | p13.1 |
| ENSG00000127954 | STEAP4 | STEAP family member 4 | 7 | q21.12 |
| ENSG00000140379 | BCL2A1 | BCL2-related protein A1 | 15 | q25.1 |
| ENSG00000121039 | RDH10 | retinol dehydrogenase 10 (all-trans) | 8 | q21.11 |
| ENSG00000157214 | STEAP2 | STEAP family member 2, metalloreductase | 7 | q21.13 |
| ENSG00000172936 | MYD88 | myeloid differentiation primary response 88 | 3 | p22.2 |
| ENSG00000172379 | ARNT2 | aryl-hydrocarbon receptor nuclear translocator 2 | 15 | q25.1 |
| ENSG00000102882 | MAPK3 | mitogen-activated protein kinase 3 | 16 | p11.2 |
| ENSG00000172939 | OXSR1 | oxidative-stress responsive 1 | 3 | p22.2 |
| ENSG00000156234 | CXCL13 | chemokine (C-X-C motif) ligand 13 | 4 | q21.1 |
| ENSG00000102879 | CORO1A | coronin, actin binding protein, 1A | 16 | p11.2 |
| ENSG00000154589 | LY96 | lymphocyte antigen 96 | 8 | q21.11 |
| ENSG00000157224 | CLDN12 | claudin 12 | 7 | q21.13 |
| ENSG00000129226 | CD68 | CD68 molecule | 17 | p13.1 |
| ENSG00000150893 | FREM2 | FRAS1 related extracellular matrix protein 2 | 13 | q13.3 |
| ENSG00000130508 | PXDN | peroxidasin homolog (Drosophila) | 2 | p25.3 |
| ENSG00000172349 | IL16 | interleukin 16 | 15 | q25.1 |
| ENSG00000169217 | CD2BP2 | CD2 (cytoplasmic tail) binding protein 2 | 16 | p11.2 |
| ENSG00000127445 | PIN1 | peptidylprolyl cis/trans isomerase, NIMA-interacting 1 | 19 | p13.2 |
| ENSG00000180209 | MYLPF | myosin light chain, phosphorylatable, fast skeletal muscle | 16 | p11.2 |
| ENSG00000138772 | ANXA3 | annexin A3 | 4 | q21.21 |
| ENSG00000104432 | IL7 | interleukin 7 | 8 | q21.13 |
| ENSG00000080573 | COL5A3 | collagen, type V, alpha 3 | 19 | p13.2 |
| ENSG00000005844 | ITGAL | integrin, alpha L (antigen CD11A (p180), lymphocyte function-associated antigen 1; alpha polypeptide) | 16 | p11.2 |
| ENSG00000139132 | FGD4 | FYVE, RhoGEF and PH domain containing 4 | 12 | p11.21 |
| ENSG00000167798 | C3P1 | complement component 3 precursor pseudogene | 19 | p13.2 |
| ENSG00000076554 | TPD52 | tumor protein D52 | 8 | q21.13 |
| ENSG00000087470 | DNM1L | dynamin 1-like | 12 | p11.21 |
| ENSG00000141510 | TP53 | tumor protein p53 | 17 | p13.1 |
| ENSG00000150907 | FOXO1 | forkhead box O1 | 13 | q14.11 |
| ENSG00000076641 | PAG1 | phosphoprotein associated with glycosphingolipid microdomains 1 | 8 | q21.13 |
| ENSG00000144655 | CSRNP1 | cysteine-serine-rich nuclear protein 1 | 3 | p22.2 |
| ENSG00000057294 | PKP2 | plakophilin 2 | 12 | p11.21 |
| ENSG00000152785 | BMP3 | bone morphogenetic protein 3 | 4 | q21.21 |
| ENSG00000168334 | XIRP1 | xin actin-binding repeat containing 1 | 3 | p22.2 |
| ENSG00000168329 | CX3CR1 | chemokine (C-X3-C motif) receptor 1 | 3 | p22.2 |
| ENSG00000103723 | AP3B2 | adaptor-related protein complex 3, beta 2 subunit | 15 | q25.2 |
| ENSG00000179934 | CCR8 | chemokine (C-C motif) receptor 8 | 3 | p22.1 |
| ENSG00000170323 | FABP4 | fatty acid binding protein 4, adipocyte | 8 | q21.13 |
| ENSG00000090339 | ICAM1 | intercellular adhesion molecule 1 | 19 | p13.2 |
| ENSG00000168028 | RPSA | ribosomal protein SA | 3 | p22.1 |
| ENSG00000105371 | ICAM4 | intercellular adhesion molecule 4 (Landsteiner-Wiener blood group) | 19 | p13.2 |
| ENSG00000120690 | ELF1 | E74-like factor 1 (ets domain transcription factor) | 13 | q14.11 |
| ENSG00000105376 | ICAM5 | intercellular adhesion molecule 5, telencephalin | 19 | p13.2 |
| ENSG00000173208 | ABCD2 | ATP-binding cassette, sub-family D (ALD), member 2 | 12 | q12 |
| ENSG00000115738 | ID2 | inhibitor of DNA binding 2, dominant negative helix-loop-helix protein | 2 | p25.1 |
| ENSG00000076662 | ICAM3 | intercellular adhesion molecule 3 | 19 | p13.2 |
| ENSG00000145284 | SCD5 | stearoyl-CoA desaturase 5 | 4 | q21.22 |
| ENSG00000099385 | BCL7C | B-cell CLL/lymphoma 7C | 16 | p11.2 |
| ENSG00000105397 | TYK2 | tyrosine kinase 2 | 19 | p13.2 |
| ENSG00000140600 | SH3GL3 | SH3-domain GRB2-like 3 | 15 | q25.2 |
| ENSG00000150281 | CTF1 | cardiotrophin 1 | 16 | p11.2 |
| ENSG00000188906 | LRRK2 | leucine-rich repeat kinase 2 | 12 | q12 |
| ENSG00000105401 | CDC37 | cell division cycle 37 homolog (S. cerevisiae) | 19 | p13.2 |
| ENSG00000164692 | COL1A2 | collagen, type I, alpha 2 | 7 | q21.3 |
| ENSG00000168036 | CTNNB1 | catenin (cadherin-associated protein), beta 1, 88kDa | 3 | p22.1 |
| ENSG00000170037 | CNTROB | centrobin, centrosomal BRCA2 interacting protein | 17 | p13.1 |
| ENSG00000018236 | CNTN1 | contactin 1 | 12 | q12 |
| ENSG00000119185 | ITGB1BP1 | integrin beta 1 binding protein 1 | 2 | p25.1 |
| ENSG00000099365 | STX1B | syntaxin 1B | 16 | p11.2 |
| ENSG00000103496 | STX4 | syntaxin 4 | 16 | p11.2 |
| ENSG00000123124 | WWP1 | WW domain containing E3 ubiquitin protein ligase 1 | 8 | q21.3 |
| ENSG00000179593 | ALOX15B | arachidonate 15-lipoxygenase, type B | 17 | p13.1 |
| ENSG00000187094 | CCK | cholecystokinin | 3 | p22.1 |
| ENSG00000049883 | PTCD2 | pentatricopeptide repeat domain 2 | 5 | q13.2 |
| ENSG00000151694 | ADAM17 | ADAM metallopeptidase domain 17 | 2 | p25.1 |
| ENSG00000103510 | KAT8 | K(lysine) acetyltransferase 8 | 16 | p11.2 |
| ENSG00000129355 | CDKN2D | cyclin-dependent kinase inhibitor 2D (p19, inhibits CDK4) | 19 | p13.2 |
| ENSG00000114857 | NKTR | natural killer-tumor recognition sequence | 3 | p22.1 |
| ENSG00000173157 | ADAMTS20 | ADAM metallopeptidase with thrombospondin type 1 motif, 20 | 12 | q12 |
| ENSG00000157107 | FCHO2 | FCH domain only 2 | 5 | q13.2 |
| ENSG00000104312 | RIPK2 | receptor-interacting serine-threonine kinase 2 | 8 | q21.3 |
| ENSG00000129351 | ILF3 | interleukin enhancer binding factor 3, 90kDa | 19 | p13.2 |
| ENSG00000104320 | NBN | nibrin | 8 | q21.3 |
| ENSG00000103490 | PYCARD | PYD and CARD domain containing | 16 | p11.2 |
| ENSG00000198001 | IRAK4 | interleukin-1 receptor-associated kinase 4 | 12 | q12 |
| ENSG00000127922 | SHFM1 | split hand/foot malformation (ectrodactyly) type 1 | 7 | q21.3 |
| ENSG00000102780 | DGKH | diacylglycerol kinase, eta | 13 | q14.11 |
| ENSG00000177238 | TRIM72 | tripartite motif containing 72 | 16 | p11.2 |
| ENSG00000170776 | AKAP13 | A kinase (PRKA) anchor protein 13 | 15 | q25.3 |
| ENSG00000169900 | PYDC1 | PYD (pyrin domain) containing 1 | 16 | p11.2 |
| ENSG00000144648 | CCBP2 | chemokine binding protein 2 | 3 | p22.1 |
| ENSG00000169896 | ITGAM | integrin, alpha M (complement component 3 receptor 3 subunit) | 16 | p11.2 |
| ENSG00000006128 | TAC1 | tachykinin, precursor 1 | 7 | q21.3 |
| ENSG00000172059 | KLF11 | Kruppel-like factor 11 | 2 | p25.1 |
| ENSG00000079805 | DNM2 | dynamin 2 | 19 | p13.2 |
| ENSG00000184613 | NELL2 | NEL-like 2 (chicken) | 12 | q12 |
| ENSG00000140678 | ITGAX | integrin, alpha X (complement component 3 receptor 4 subunit) | 16 | p11.2 |
| ENSG00000023516 | AKAP11 | A kinase (PRKA) anchor protein 11 | 13 | q14.11 |
| ENSG00000070669 | ASNS | asparagine synthetase (glutamine-hydrolyzing) | 7 | q21.3 |
| ENSG00000178999 | AURKB | aurora kinase B | 17 | p13.1 |
| ENSG00000163788 | SNRK | SNF related kinase | 3 | p22.1 |
| ENSG00000156886 | ITGAD | integrin, alpha D | 16 | p11.2 |
| ENSG00000120659 | TNFSF11 | tumor necrosis factor (ligand) superfamily, member 11 | 13 | q14.11 |
| ENSG00000163629 | PTPN13 | protein tyrosine phosphatase, non-receptor type 13 (APO-1/CD95 (Fas)-associated phosphatase) | 4 | q21.3 |
| ENSG00000140682 | TGFB1I1 | transforming growth factor beta 1 induced transcript 1 | 16 | p11.2 |
| ENSG00000140675 | SLC5A2 | solute carrier family 5 (sodium/glucose cotransporter), member 2 | 16 | p11.2 |
| ENSG00000110148 | CCKBR | cholecystokinin B receptor | 11 | p15.4 |
| ENSG00000170955 | PRKCDBP | protein kinase C, delta binding protein | 11 | p15.4 |
| ENSG00000181026 | AEN | apoptosis enhancing nuclease | 15 | q26.1 |
| ENSG00000166311 | SMPD1 | sphingomyelin phosphodiesterase 1, acid lysosomal | 11 | p15.4 |
| ENSG00000172183 | ISG20 | interferon stimulated exonuclease gene 20kDa | 15 | q26.1 |
| ENSG00000139211 | AMIGO2 | adhesion molecule with Ig-like domain 2 | 12 | q13.11 |
| ENSG00000157766 | ACAN | aggrecan | 15 | q26.1 |
| ENSG00000166313 | APBB1 | amyloid beta (A4) precursor protein-binding, family B, member 1 (Fe65) | 11 | p15.4 |
| ENSG00000079112 | CDH17 | cadherin 17, LI cadherin (liver-intestine) | 8 | q22.1 |
| ENSG00000130164 | LDLR | low density lipoprotein receptor | 19 | p13.2 |
| ENSG00000198742 | SMURF1 | SMAD specific E3 ubiquitin protein ligase 1 | 7 | q22.1 |
| ENSG00000140511 | HAPLN3 | hyaluronan and proteoglycan link protein 3 | 15 | q26.1 |
| ENSG00000079337 | RAPGEF3 | Rap guanine nucleotide exchange factor (GEF) 3 | 12 | q13.11 |
| ENSG00000110169 | HPX | hemopexin | 11 | p15.4 |
| ENSG00000140545 | MFGE8 | milk fat globule-EGF factor 8 protein | 15 | q26.1 |
| ENSG00000130158 | DOCK6 | dedicator of cytokinesis 6 | 19 | p13.2 |
| ENSG00000061273 | HDAC7 | histone deacetylase 7 | 12 | q13.11 |
| ENSG00000152583 | SPARCL1 | SPARC-like 1 (hevin) | 4 | q22.1 |
| ENSG00000105514 | RAB3D | RAB3D, member RAS oncogene family | 19 | p13.2 |
| ENSG00000140525 | FANCI | Fanconi anemia, complementation group I | 15 | q26.1 |
| ENSG00000133026 | MYH10 | myosin, heavy chain 10, non-muscle | 17 | p13.1 |
| ENSG00000152592 | DMP1 | dentin matrix acidic phosphoprotein 1 | 4 | q22.1 |
| ENSG00000111424 | VDR | vitamin D (1,25- dihydroxyvitamin D3) receptor | 12 | q13.11 |
| ENSG00000029559 | IBSP | integrin-binding sialoprotein | 4 | q22.1 |
| ENSG00000164938 | TP53INP1 | tumor protein p53 inducible nuclear protein 1 | 8 | q22.1 |
| ENSG00000187266 | EPOR | erythropoietin receptor | 19 | p13.2 |
| ENSG00000118785 | SPP1 | secreted phosphoprotein 1 | 4 | q22.1 |
| ENSG00000133112 | TPT1 | tumor protein, translationally-controlled 1 | 13 | q14.13 |
| ENSG00000139219 | COL2A1 | collagen, type II, alpha 1 | 12 | q13.11 |
| ENSG00000156466 | GDF6 | growth differentiation factor 6 | 8 | q22.1 |
| ENSG00000166333 | ILK | integrin-linked kinase | 11 | p15.4 |
| ENSG00000152359 | POC5 | POC5 centriolar protein homolog (Chlamydomonas) | 5 | q13.3 |
| ENSG00000130175 | PRKCSH | protein kinase C substrate 80K-H | 19 | p13.2 |
| ENSG00000166821 | PEX11A | peroxisomal biogenesis factor 11 alpha | 15 | q26.1 |
| ENSG00000166341 | DCHS1 | dachsous 1 (Drosophila) | 11 | p15.4 |
| ENSG00000129636 | ITFG1 | integrin alpha FG-GAP repeat containing 1 | 16 | q12.1 |
| ENSG00000102575 | ACP5 | acid phosphatase 5, tartrate resistant | 19 | p13.2 |
| ENSG00000134318 | ROCK2 | Rho-associated, coiled-coil containing protein kinase 2 | 2 | p25.1 |
| ENSG00000065320 | NTN1 | netrin 1 | 17 | p13.1 |
| ENSG00000181104 | F2R | coagulation factor II (thrombin) receptor | 5 | q13.3 |
| ENSG00000079387 | SENP1 | SUMO1/sentrin specific peptidase 1 | 12 | q13.11 |
| ENSG00000164251 | F2RL1 | coagulation factor II (thrombin) receptor-like 1 | 5 | q13.3 |
| ENSG00000173585 | CCR9 | chemokine (C-C motif) receptor 9 | 3 | p21.31 |
| ENSG00000164252 | AGGF1 | angiogenic factor with G patch and FHA domains 1 | 5 | q13.3 |
| ENSG00000185033 | SEMA4B | sema domain, immunoglobulin domain (Ig), transmembrane domain (TM) and short cytoplasmic domain, (semaphorin) 4B | 15 | q26.1 |
| ENSG00000185043 | CIB1 | calcium and integrin binding 1 (calmyrin) | 15 | q26.1 |
| ENSG00000172215 | CXCR6 | chemokine (C-X-C motif) receptor 6 | 3 | p21.31 |
| ENSG00000173578 | XCR1 | chemokine (C motif) receptor 1 | 3 | p21.31 |
| ENSG00000170743 | SYT9 | synaptotagmin IX | 11 | p15.4 |
| ENSG00000104375 | STK3 | serine/threonine kinase 3 | 8 | q22.2 |
| ENSG00000145335 | SNCA | synuclein, alpha (non A4 component of amyloid precursor) | 4 | q22.1 |
| ENSG00000163823 | CCR1 | chemokine (C-C motif) receptor 1 | 3 | p21.31 |
| ENSG00000196470 | SIAH1 | siah E3 ubiquitin protein ligase 1 | 16 | q12.1 |
| ENSG00000183625 | CCR3 | chemokine (C-C motif) receptor 3 | 3 | p21.31 |
| ENSG00000167531 | LALBA | lactalbumin, alpha- | 12 | q13.11 |
| ENSG00000138722 | MMRN1 | multimerin 1 | 4 | q22.1 |
| ENSG00000121807 | CCR2 | chemokine (C-C motif) receptor 2 | 3 | p21.31 |
| ENSG00000160862 | AZGP1 | alpha-2-glycoprotein 1, zinc-binding | 7 | q22.1 |
| ENSG00000160791 | CCR5 | chemokine (C-C motif) receptor 5 (gene/pseudogene) | 3 | p21.31 |
| ENSG00000152208 | GRID2 | glutamate receptor, ionotropic, delta 2 | 4 | q22.1 |
| ENSG00000071575 | TRIB2 | tribbles homolog 2 (Drosophila) | 2 | p24.3 |
| ENSG00000172238 | ATOH1 | atonal homolog 1 (Drosophila) | 4 | q22.2 |
| ENSG00000197299 | BLM | Bloom syndrome, RecQ helicase-like | 15 | q26.1 |
| ENSG00000180855 | ZNF443 | zinc finger protein 443 | 19 | p13.2 |
| ENSG00000136167 | LCP1 | lymphocyte cytosolic protein 1 (L-plastin) | 13 | q14.13 |
| ENSG00000166526 | ZNF3 | zinc finger protein 3 | 7 | q22.1 |
| ENSG00000121797 | CCRL2 | chemokine (C-C motif) receptor-like 2 | 3 | p21.31 |
| ENSG00000167535 | CACNB3 | calcium channel, voltage-dependent, beta 3 subunit | 12 | q13.12 |
| ENSG00000140564 | FURIN | furin (paired basic amino acid cleaving enzyme) | 15 | q26.1 |
| ENSG00000163106 | HPGDS | hematopoietic prostaglandin D synthase | 4 | q22.3 |
| ENSG00000166402 | TUB | tubby homolog (mouse) | 11 | p15.4 |
| ENSG00000132842 | AP3B1 | adaptor-related protein complex 3, beta 1 subunit | 5 | q14.1 |
| ENSG00000163110 | PDLIM5 | PDZ and LIM domain 5 | 4 | q22.3 |
| ENSG00000172602 | RND1 | Rho family GTPase 1 | 12 | q13.12 |
| ENSG00000134285 | FKBP11 | FK506 binding protein 11, 19 kDa | 12 | q13.12 |
| ENSG00000167207 | NOD2 | nucleotide-binding oligomerization domain containing 2 | 16 | q12.1 |
| ENSG00000138696 | BMPR1B | bone morphogenetic protein receptor, type IB | 4 | q22.3 |
| ENSG00000169884 | WNT10B | wingless-type MMTV integration site family, member 10B | 12 | q13.12 |
| ENSG00000198901 | PRC1 | protein regulator of cytokinesis 1 | 15 | q26.1 |
| ENSG00000182168 | UNC5C | unc-5 homolog C (C. elegans) | 4 | q22.3 |
| ENSG00000125084 | WNT1 | wingless-type MMTV integration site family, member 1 | 12 | q13.12 |
| ENSG00000184056 | VPS33B | vacuolar protein sorting 33 homolog B (yeast) | 15 | q26.1 |
| ENSG00000181929 | PRKAG1 | protein kinase, AMP-activated, gamma 1 non-catalytic subunit | 12 | q13.12 |
| ENSG00000167548 | MLL2 | myeloid/lymphoid or mixed-lineage leukemia 2 | 12 | q13.12 |
| ENSG00000164924 | YWHAZ | tyrosine 3-monooxygenase/tryptophan 5-monooxygenase activation protein, zeta polypeptide | 8 | q22.3 |
| ENSG00000095066 | HOOK2 | hook homolog 2 (Drosophila) | 19 | p13.13 |
| ENSG00000171223 | JUNB | jun B proto-oncogene | 19 | p13.13 |
| ENSG00000152409 | JMY | junction mediating and regulatory protein, p53 cofactor | 5 | q14.1 |
| ENSG00000167815 | PRDX2 | peroxiredoxin 2 | 19 | p13.13 |
| ENSG00000139636 | LMBR1L | limb region 1 homolog (mouse)-like | 12 | q13.12 |
| ENSG00000166971 | AKTIP | AKT interacting protein | 16 | q12.2 |
| ENSG00000197894 | ADH5 | alcohol dehydrogenase 5 (class III), chi polypeptide | 4 | q23 |
| ENSG00000048392 | RRM2B | ribonucleotide reductase M2 B (TP53 inducible) | 8 | q22.3 |
| ENSG00000140718 | FTO | fat mass and obesity associated | 16 | q12.2 |
| ENSG00000105613 | MAST1 | microtubule associated serine/threonine kinase 1 | 19 | p13.13 |
| ENSG00000113296 | THBS4 | thrombospondin 4 | 5 | q14.1 |
| ENSG00000105612 | DNASE2 | deoxyribonuclease II, lysosomal | 19 | p13.13 |
| ENSG00000105610 | KLF1 | Kruppel-like factor 1 (erythroid) | 19 | p13.13 |
| ENSG00000185551 | NR2F2 | nuclear receptor subfamily 2, group F, member 2 | 15 | q26.2 |
| ENSG00000087253 | LPCAT2 | lysophosphatidylcholine acyltransferase 2 | 16 | q12.2 |
| ENSG00000155090 | KLF10 | Kruppel-like factor 10 | 8 | q22.3 |
| ENSG00000039319 | ZFYVE16 | zinc finger, FYVE domain containing 16 | 5 | q14.1 |
| ENSG00000135451 | TROAP | trophinin associated protein | 12 | q13.12 |
| ENSG00000179218 | CALR | calreticulin | 19 | p13.13 |
| ENSG00000140443 | IGF1R | insulin-like growth factor 1 receptor | 15 | q26.3 |
| ENSG00000186897 | C1QL4 | complement component 1, q subcomponent-like 4 | 12 | q13.12 |
| ENSG00000133789 | SWAP70 | SWAP switching B-cell complex 70kDa subunit | 11 | p15.4 |
| ENSG00000198848 | CES1 | carboxylesterase 1 | 16 | q12.2 |
| ENSG00000113318 | MSH3 | mutS homolog 3 (E. coli) | 5 | q14.1 |
| ENSG00000164929 | BAALC | brain and acute leukemia, cytoplasmic | 8 | q22.3 |
| ENSG00000179284 | DAND5 | DAN domain family, member 5 | 19 | p13.13 |
| ENSG00000148926 | ADM | adrenomedullin | 11 | p15.4 |
| ENSG00000113319 | RASGRF2 | Ras protein-specific guanine nucleotide-releasing factor 2 | 5 | q14.1 |
| ENSG00000087258 | GNAO1 | guanine nucleotide binding protein (G protein), alpha activating activity polypeptide O | 16 | q13 |
| ENSG00000068305 | MEF2A | myocyte enhancer factor 2A | 15 | q26.3 |
| ENSG00000104903 | LYL1 | lymphoblastic leukemia derived sequence 1 | 19 | p13.13 |
| ENSG00000143867 | OSR1 | odd-skipped related 1 (Drosophila) | 2 | p24.1 |
| ENSG00000133800 | LYVE1 | lymphatic vessel endothelial hyaluronan receptor 1 | 11 | p15.4 |
| ENSG00000160877 | NACC1 | nucleus accumbens associated 1, BEN and BTB (POZ) domain containing | 19 | p13.13 |
| ENSG00000176406 | RIMS2 | regulating synaptic membrane exocytosis 2 | 8 | q22.3 |
| ENSG00000153064 | BANK1 | B-cell scaffold protein with ankyrin repeats 1 | 4 | q24 |
| ENSG00000170425 | ADORA2B | adenosine A2b receptor | 17 | p12 |
| ENSG00000136156 | ITM2B | integral membrane protein 2B | 13 | q14.2 |
| ENSG00000130427 | EPO | erythropoietin | 7 | q22.1 |
| ENSG00000139644 | TMBIM6 | transmembrane BAX inhibitor motif containing 6 | 12 | q13.12 |
| ENSG00000109320 | NFKB1 | nuclear factor of kappa light polypeptide gene enhancer in B-cells 1 | 4 | q24 |
| ENSG00000146839 | ZAN | zonadhesin | 7 | q22.1 |
| ENSG00000141837 | CACNA1A | calcium channel, voltage-dependent, P/Q type, alpha 1A subunit | 19 | p13.13 |
| ENSG00000125124 | BBS2 | Bardet-Biedl syndrome 2 | 16 | q13 |
| ENSG00000135472 | FAIM2 | Fas apoptotic inhibitory molecule 2 | 12 | q13.12 |
| ENSG00000147650 | LRP12 | low density lipoprotein receptor-related protein 12 | 8 | q22.3 |
| ENSG00000167580 | AQP2 | aquaporin 2 (collecting duct) | 12 | q13.12 |
| ENSG00000139687 | RB1 | retinoblastoma 1 | 13 | q14.2 |
| ENSG00000109332 | UBE2D3 | ubiquitin-conjugating enzyme E2D 3 | 4 | q24 |
| ENSG00000166582 | CENPV | centromere protein V | 17 | p11.2 |
| ENSG00000184254 | ALDH1A3 | aldehyde dehydrogenase 1 family, member A3 | 15 | q26.3 |
| ENSG00000161800 | RACGAP1 | Rac GTPase activating protein 1 | 12 | q13.12 |
| ENSG00000152422 | XRCC4 | X-ray repair complementing defective repair in Chinese hamster cells 4 | 5 | q14.2 |
| ENSG00000087077 | TRIP6 | thyroid hormone receptor interactor 6 | 7 | q22.1 |
| ENSG00000154188 | ANGPT1 | angiopoietin 1 | 8 | q23.1 |
| ENSG00000038427 | VCAN | versican | 5 | q14.2 |
| ENSG00000164047 | CAMP | cathelicidin antimicrobial peptide | 3 | p21.31 |
| ENSG00000131871 | VIMP | VCP-interacting membrane protein | 15 | q26.3 |
| ENSG00000141040 | ZNF287 | zinc finger protein 287 | 17 | p11.2 |
| ENSG00000172113 | NME6 | NME/NM23 nucleoside diphosphate kinase 6 | 3 | p21.31 |
| ENSG00000143878 | RHOB | ras homolog family member B | 2 | p24.1 |
| ENSG00000197702 | PARVA | parvin, alpha | 11 | p15.3 |
| ENSG00000145681 | HAPLN1 | hyaluronan and proteoglycan link protein 1 | 5 | q14.3 |
| ENSG00000087085 | ACHE | acetylcholinesterase | 7 | q22.1 |
| ENSG00000164176 | EDIL3 | EGF-like repeats and discoidin I-like domains 3 | 5 | q14.3 |
| ENSG00000108516 | TNFRSF13B | tumor necrosis factor receptor superfamily, member 13B | 17 | q21.2 |
| ENSG00000051108 | HERPUD1 | homocysteine-inducible, endoplasmic reticulum stress-inducible, ubiquitin-like domain member 1 | 16 | q13 |
| ENSG00000143869 | GDF7 | growth differentiation factor 7 | 2 | p24.1 |
| ENSG00000132005 | RFX1 | regulatory factor X, 1 (influences HLA class II expression) | 19 | p13.12 |
| ENSG00000087237 | CETP | cholesteryl ester transfer protein, plasma | 16 | q13 |
| ENSG00000147654 | EBAG9 | estrogen receptor binding site associated, antigen, 9 | 8 | q23.2 |
| ENSG00000104998 | IL27RA | interleukin 27 receptor, alpha | 19 | p13.12 |
| ENSG00000140853 | NLRC5 | NLR family, CARD domain containing 5 | 16 | q13 |
| ENSG00000084674 | APOB | apolipoprotein B (including Ag(x) antigen) | 2 | p24.1 |
| ENSG00000145715 | RASA1 | RAS p21 protein activator (GTPase activating protein) 1 | 5 | q14.3 |
| ENSG00000102531 | FNDC3A | fibronectin type III domain containing 3A | 13 | q14.2 |
| ENSG00000152266 | PTH | parathyroid hormone | 11 | p15.3 |
| ENSG00000110911 | SLC11A2 | solute carrier family 11 (proton-coupled divalent metal ion transporters), member 2 | 12 | q13.12 |
| ENSG00000072062 | PRKACA | protein kinase, cAMP-dependent, catalytic, alpha | 19 | p13.12 |
| ENSG00000164054 | SHISA5 | shisa homolog 5 (Xenopus laevis) | 3 | p21.31 |
| ENSG00000106366 | SERPINE1 | serpin peptidase inhibitor, clade E (nexin, plasminogen activator inhibitor type 1), member 1 | 7 | q22.1 |
| ENSG00000106367 | AP1S1 | adaptor-related protein complex 1, sigma 1 subunit | 7 | q22.1 |
| ENSG00000081189 | MEF2C | myocyte enhancer factor 2C | 5 | q14.3 |
| ENSG00000114270 | COL7A1 | collagen, type VII, alpha 1 | 3 | p21.31 |
| ENSG00000110925 | CSRNP2 | cysteine-serine-rich nuclear protein 2 | 12 | q13.12 |
| ENSG00000168743 | NPNT | nephronectin | 4 | q24 |
| ENSG00000123146 | CD97 | CD97 molecule | 19 | p13.12 |
| ENSG00000129084 | PSMA1 | proteasome (prosome, macropain) subunit, alpha type, 1 | 11 | p15.2 |
| ENSG00000113356 | POLR3G | polymerase (RNA) III (DNA directed) polypeptide G (32kD) | 5 | q14.3 |
| ENSG00000102962 | CCL22 | chemokine (C-C motif) ligand 22 | 16 | q21 |
| ENSG00000006210 | CX3CL1 | chemokine (C-X3-C motif) ligand 1 | 16 | q21 |
| ENSG00000152270 | PDE3B | phosphodiesterase 3B, cGMP-inhibited | 11 | p15.2 |
| ENSG00000164199 | GPR98 | G protein-coupled receptor 98 | 5 | q14.3 |
| ENSG00000106404 | CLDN15 | claudin 15 | 7 | q22.1 |
| ENSG00000164022 | AIMP1 | aminoacyl tRNA synthetase complex-interacting multifunctional protein 1 | 4 | q24 |
| ENSG00000102970 | CCL17 | chemokine (C-C motif) ligand 17 | 16 | q21 |
| ENSG00000119782 | FKBP1B | FK506 binding protein 1B, 12.6 kDa | 2 | p23.3 |
| ENSG00000005194 | CIAPIN1 | cytokine induced apoptosis inhibitor 1 | 16 | q21 |
| ENSG00000123143 | PKN1 | protein kinase N1 | 19 | p13.12 |
| ENSG00000170545 | SMAGP | small cell adhesion glycoprotein | 12 | q13.13 |
| ENSG00000110680 | CALCA | calcitonin-related polypeptide alpha | 11 | p15.2 |
| ENSG00000008300 | CELSR3 | cadherin, EGF LAG seven-pass G-type receptor 3 (flamingo homolog, Drosophila) | 3 | p21.31 |
| ENSG00000160951 | PTGER1 | prostaglandin E receptor 1 (subtype EP1), 42kDa | 19 | p13.12 |
| ENSG00000123159 | GIPC1 | GIPC PDZ domain containing family, member 1 | 19 | p13.12 |
| ENSG00000164754 | RAD21 | RAD21 homolog (S. pombe) | 8 | q24.11 |
| ENSG00000115129 | TP53I3 | tumor protein p53 inducible protein 3 | 2 | p23.3 |
| ENSG00000160999 | SH2B2 | SH2B adaptor protein 2 | 7 | q22.1 |
| ENSG00000205336 | GPR56 | G protein-coupled receptor 56 | 16 | q21 |
| ENSG00000138795 | LEF1 | lymphoid enhancer-binding factor 1 | 4 | q25 |
| ENSG00000198399 | ITSN2 | intersectin 2 | 2 | p23.3 |
| ENSG00000166689 | PLEKHA7 | pleckstrin homology domain containing, family A member 7 | 11 | p15.1 |
| ENSG00000131899 | LLGL1 | lethal giant larvae homolog 1 (Drosophila) | 17 | p11.2 |
| ENSG00000068745 | IP6K2 | inositol hexakisphosphate kinase 2 | 3 | p21.31 |
| ENSG00000164761 | TNFRSF11B | tumor necrosis factor receptor superfamily, member 11b | 8 | q24.12 |
| ENSG00000135503 | ACVR1B | activin A receptor, type IB | 12 | q13.13 |
| ENSG00000114302 | PRKAR2A | protein kinase, cAMP-dependent, regulatory, type II, alpha | 3 | p21.31 |
| ENSG00000147676 | MAL2 | mal, T-cell differentiation protein 2 (gene/pseudogene) | 8 | q24.12 |
| ENSG00000136960 | ENPP2 | ectonucleotide pyrophosphatase/phosphodiesterase 2 | 8 | q24.12 |
| ENSG00000140859 | KIFC3 | kinesin family member C3 | 16 | q21 |
| ENSG00000139567 | ACVRL1 | activin A receptor type II-like 1 | 12 | q13.13 |
| ENSG00000138794 | CASP6 | caspase 6, apoptosis-related cysteine peptidase | 4 | q25 |
| ENSG00000205403 | CFI | complement factor I | 4 | q25 |
| ENSG00000123358 | NR4A1 | nuclear receptor subfamily 4, group A, member 1 | 12 | q13.13 |
| ENSG00000155792 | DEPTOR | DEP domain containing MTOR-interacting protein | 8 | q24.12 |
| ENSG00000128606 | LRRC17 | leucine rich repeat containing 17 | 7 | q22.1 |
| ENSG00000183423 | LRIT3 | leucine-rich repeat, immunoglobulin-like and transmembrane domains 3 | 4 | q25 |
| ENSG00000105143 | SLC1A6 | solute carrier family 1 (high affinity aspartate/glutamate transporter), member 6 | 19 | p13.12 |
| ENSG00000187955 | COL14A1 | collagen, type XIV, alpha 1 | 8 | q24.12 |
| ENSG00000138798 | EGF | epidermal growth factor | 4 | q25 |
| ENSG00000105141 | CASP14 | caspase 14, apoptosis-related cysteine peptidase | 19 | p13.12 |
| ENSG00000178035 | IMPDH2 | IMP (inosine 5'-monophosphate) dehydrogenase 2 | 3 | p21.31 |
| ENSG00000175426 | PCSK1 | proprotein convertase subtilisin/kexin type 1 | 5 | q15 |
| ENSG00000129152 | MYOD1 | myogenic differentiation 1 | 11 | p15.1 |
| ENSG00000161057 | PSMC2 | proteasome (prosome, macropain) 26S subunit, ATPase, 2 | 7 | q22.1 |
| ENSG00000115138 | POMC | proopiomelanocortin | 2 | p23.3 |
| ENSG00000176124 | DLEU1 | deleted in lymphocytic leukemia 1 (non-protein coding) | 13 | q14.2 |
| ENSG00000172037 | LAMB2 | laminin, beta 2 (laminin S) | 3 | p21.31 |
| ENSG00000136986 | DERL1 | derlin 1 | 8 | q24.13 |
| ENSG00000186047 | DLEU7 | deleted in lymphocytic leukemia, 7 | 13 | q14.3 |
| ENSG00000166788 | SAAL1 | serum amyloid A-like 1 | 11 | p15.1 |
| ENSG00000189056 | RELN | reelin | 7 | q22.1 |
| ENSG00000166787 | SAA3P | serum amyloid A3 pseudogene | 11 | p15.1 |
| ENSG00000072134 | EPN2 | epsin 2 | 17 | p11.2 |
| ENSG00000114316 | USP4 | ubiquitin specific peptidase 4 (proto-oncogene) | 3 | p21.31 |
| ENSG00000148965 | SAA4 | serum amyloid A4, constitutive | 11 | p15.1 |
| ENSG00000134339 | SAA2 | serum amyloid A2 | 11 | p15.1 |
| ENSG00000166484 | MAPK7 | mitogen-activated protein kinase 7 | 17 | p11.2 |
| ENSG00000173432 | SAA1 | serum amyloid A1 | 11 | p15.1 |
| ENSG00000164307 | ERAP1 | endoplasmic reticulum aminopeptidase 1 | 5 | q15 |
| ENSG00000067560 | RHOA | ras homolog family member A | 3 | p21.31 |
| ENSG00000005483 | MLL5 | myeloid/lymphoid or mixed-lineage leukemia 5 (trithorax homolog, Drosophila) | 7 | q22.3 |
| ENSG00000166482 | MFAP4 | microfibrillar-associated protein 4 | 17 | p11.2 |
| ENSG00000150394 | CDH8 | cadherin 8, type 2 | 16 | q21 |
| ENSG00000140937 | CDH11 | cadherin 11, type 2, OB-cadherin (osteoblast) | 16 | q21 |
| ENSG00000145022 | TCTA | T-cell leukemia translocation altered | 3 | p21.31 |
| ENSG00000164308 | ERAP2 | endoplasmic reticulum aminopeptidase 2 | 5 | q15 |
| ENSG00000186526 | CYP4F8 | cytochrome P450, family 4, subfamily F, polypeptide 8 | 19 | p13.12 |
| ENSG00000179776 | CDH5 | cadherin 5, type 2 (vascular endothelium) | 16 | q21 |
| ENSG00000173402 | DAG1 | dystroglycan 1 (dystrophin-associated glycoprotein 1) | 3 | p21.31 |
| ENSG00000167768 | KRT1 | keratin 1 | 12 | q13.13 |
| ENSG00000135250 | SRPK2 | SRSF protein kinase 2 | 7 | q22.3 |
| ENSG00000204960 | BLACE | B-cell acute lymphoblastic leukemia expressed | 7 | q36.3 |
| ENSG00000145349 | CAMK2D | calcium/calmodulin-dependent protein kinase II delta | 4 | q26 |
| ENSG00000173531 | MST1 | macrophage stimulating 1 (hepatocyte growth factor-like) | 3 | p21.31 |
| ENSG00000170873 | MTSS1 | metastasis suppressor 1 | 8 | q24.13 |
| ENSG00000089505 | CMTM1 | CKLF-like MARVEL transmembrane domain containing 1 | 16 | q21 |
| ENSG00000174136 | RGMB | RGM domain family, member B | 5 | q15 |
| ENSG00000128536 | CDHR3 | cadherin-related family member 3 | 7 | q22.3 |
| ENSG00000179057 | IGSF22 | immunoglobulin superfamily, member 22 | 11 | p15.1 |
| ENSG00000105835 | NAMPT | nicotinamide phosphoribosyltransferase | 7 | q22.3 |
| ENSG00000111057 | KRT18 | keratin 18 | 12 | q13.13 |
| ENSG00000167461 | RAB8A | RAB8A, member RAS oncogene family | 19 | p13.11 |
| ENSG00000105851 | PIK3CG | phosphatidylinositol-4,5-bisphosphate 3-kinase, catalytic subunit gamma | 7 | q22.3 |
| ENSG00000176020 | AMIGO3 | adhesion molecule with Ig-like domain 3 | 3 | p21.31 |
| ENSG00000196684 | HSH2D | hematopoietic SH2 domain containing | 19 | p13.11 |
| ENSG00000140932 | CMTM2 | CKLF-like MARVEL transmembrane domain containing 2 | 16 | q21 |
| ENSG00000141977 | CIB3 | calcium and integrin binding family member 3 | 19 | p13.11 |
| ENSG00000170255 | MRGPRX1 | MAS-related GPR, member X1 | 11 | p15.1 |
| ENSG00000005249 | PRKAR2B | protein kinase, cAMP-dependent, regulatory, type II, beta | 7 | q22.3 |
| ENSG00000140931 | CMTM3 | CKLF-like MARVEL transmembrane domain containing 3 | 16 | q22.1 |
| ENSG00000173334 | TRIB1 | tribbles homolog 1 (Drosophila) | 8 | q24.13 |
| ENSG00000183723 | CMTM4 | CKLF-like MARVEL transmembrane domain containing 4 | 16 | q22.1 |
| ENSG00000136997 | MYC | v-myc myelocytomatosis viral oncogene homolog (avian) | 8 | q24.21 |
| ENSG00000145388 | METTL14 | methyltransferase like 14 | 4 | q26 |
| ENSG00000187492 | CDHR4 | cadherin-related family member 4 | 3 | p21.31 |
| ENSG00000127528 | KLF2 | Kruppel-like factor 2 (lung) | 19 | p13.11 |
| ENSG00000136108 | CKAP2 | cytoskeleton associated protein 2 | 13 | q14.3 |
| ENSG00000127527 | EPS15L1 | epidermal growth factor receptor pathway substrate 15-like 1 | 19 | p13.11 |
| ENSG00000159593 | NAE1 | NEDD8 activating enzyme E1 subunit 1 | 16 | q22.1 |
| ENSG00000183763 | TRAIP | TRAF interacting protein | 3 | p21.31 |
| ENSG00000075790 | BCAP29 | B-cell receptor-associated protein 29 | 7 | q22.3 |
| ENSG00000166589 | CDH16 | cadherin 16, KSP-cadherin | 16 | q22.1 |
| ENSG00000105879 | CBLL1 | Cbl proto-oncogene, E3 ubiquitin protein ligase-like 1 | 7 | q22.3 |
| ENSG00000164078 | MST1R | macrophage stimulating 1 receptor (c-met-related tyrosine kinase) | 3 | p21.31 |
| ENSG00000166592 | RRAD | Ras-related associated with diabetes | 16 | q22.1 |
| ENSG00000138735 | PDE5A | phosphodiesterase 5A, cGMP-specific | 4 | q26 |
| ENSG00000167780 | SOAT2 | sterol O-acyltransferase 2 | 12 | q13.13 |
| ENSG00000091136 | LAMB1 | laminin, beta 1 | 7 | q31.1 |
| ENSG00000109854 | HTATIP2 | HIV-1 Tat interactive protein 2, 30kDa | 11 | p15.1 |
| ENSG00000003756 | RBM5 | RNA binding motif protein 5 | 3 | p21.31 |
| ENSG00000127533 | F2RL3 | coagulation factor II (thrombin) receptor-like 3 | 19 | p13.11 |
| ENSG00000136110 | LECT1 | leukocyte cell derived chemotaxin 1 | 13 | q14.3 |
| ENSG00000001617 | SEMA3F | sema domain, immunoglobulin domain (Ig), short basic domain, secreted, (semaphorin) 3F | 3 | p21.31 |
| ENSG00000091128 | LAMB4 | laminin, beta 4 | 7 | q31.1 |
| ENSG00000157884 | CIB4 | calcium and integrin binding family member 4 | 2 | p23.3 |
| ENSG00000165973 | NELL1 | NEL-like 1 (chicken) | 11 | p15.1 |
| ENSG00000007171 | NOS2 | nitric oxide synthase 2, inducible | 17 | q11.2 |
| ENSG00000102871 | TRADD | TNFRSF1A-associated via death domain | 16 | q22.1 |
| ENSG00000136099 | PCDH8 | protocadherin 8 | 13 | q14.3 |
| ENSG00000139626 | ITGB7 | integrin, beta 7 | 12 | q13.13 |
| ENSG00000050730 | TNIP3 | TNFAIP3 interacting protein 3 | 4 | q27 |
| ENSG00000091129 | NRCAM | neuronal cell adhesion molecule | 7 | q31.1 |
| ENSG00000114353 | GNAI2 | guanine nucleotide binding protein (G protein), alpha inhibiting activity polypeptide 2 | 3 | p21.31 |
| ENSG00000140939 | NOL3 | nucleolar protein 3 (apoptosis repressor with CARD domain) | 16 | q22.1 |
| ENSG00000102837 | OLFM4 | olfactomedin 4 | 13 | q14.3 |
| ENSG00000172819 | RARG | retinoic acid receptor, gamma | 12 | q13.13 |
| ENSG00000042832 | TG | thyroglobulin | 8 | q24.22 |
| ENSG00000179044 | EXOC3L1 | exocyst complex component 3-like 1 | 16 | q22.1 |
| ENSG00000164111 | ANXA5 | annexin A5 | 4 | q27 |
| ENSG00000151422 | FER | fer (fps/fes related) tyrosine kinase | 5 | q21.3 |
| ENSG00000183161 | FANCF | Fanconi anemia, complementation group F | 11 | p14.3 |
| ENSG00000012171 | SEMA3B | sema domain, immunoglobulin domain (Ig), short basic domain, secreted, (semaphorin) 3B | 3 | p21.31 |
| ENSG00000087095 | NLK | nemo-like kinase | 17 | q11.2 |
| ENSG00000148935 | GAS2 | growth arrest-specific 2 | 11 | p14.3 |
| ENSG00000135476 | ESPL1 | extra spindle pole bodies homolog 1 (S. cerevisiae) | 12 | q13.13 |
| ENSG00000102890 | ELMO3 | engulfment and cell motility 3 | 16 | q22.1 |
| ENSG00000104415 | WISP1 | WNT1 inducible signaling pathway protein 1 | 8 | q24.22 |
| ENSG00000118946 | PCDH17 | protocadherin 17 | 13 | q21.1 |
| ENSG00000109083 | IFT20 | intraflagellar transport 20 homolog (Chlamydomonas) | 17 | q11.2 |
| ENSG00000109079 | TNFAIP1 | tumor necrosis factor, alpha-induced protein 1 (endothelial) | 17 | q11.2 |
| ENSG00000104419 | NDRG1 | N-myc downstream regulated 1 | 8 | q24.22 |
| ENSG00000138686 | BBS7 | Bardet-Biedl syndrome 7 | 4 | q27 |
| ENSG00000145777 | TSLP | thymic stromal lymphopoietin | 5 | q22.1 |
| ENSG00000178591 | DEFB125 | defensin, beta 125 | 20 | p13 |
| ENSG00000173114 | LRRN3 | leucine rich repeat neuronal 3 | 7 | q31.1 |
| ENSG00000128512 | DOCK4 | dedicator of cytokinesis 4 | 7 | q31.1 |
| ENSG00000125788 | DEFB126 | defensin, beta 126 | 20 | p13 |
| ENSG00000130303 | BST2 | bone marrow stromal cell antigen 2 | 19 | p13.11 |
| ENSG00000088782 | DEFB127 | defensin, beta 127 | 20 | p13 |
| ENSG00000135409 | AMHR2 | anti-Mullerian hormone receptor, type II | 12 | q13.13 |
| ENSG00000185982 | DEFB128 | defensin, beta 128 | 20 | p13 |
| ENSG00000007216 | SLC13A2 | solute carrier family 13 (sodium-dependent dicarboxylate transporter), member 2 | 17 | q11.2 |
| ENSG00000125903 | DEFB129 | defensin, beta 129 | 20 | p13 |
| ENSG00000186458 | DEFB132 | defensin, beta 132 | 20 | p13 |
| ENSG00000138080 | EMILIN1 | elastin microfibril interfacer 1 | 2 | p23.3 |
| ENSG00000148943 | LIN7C | lin-7 homolog C (C. elegans) | 11 | p14.1 |
| ENSG00000109101 | FOXN1 | forkhead box N1 | 17 | q11.2 |
| ENSG00000197991 | PCDH20 | protocadherin 20 | 13 | q21.2 |
| ENSG00000006652 | IFRD1 | interferon-related developmental regulator 1 | 7 | q31.1 |
| ENSG00000176697 | BDNF | brain-derived neurotrophic factor | 11 | p14.1 |
| ENSG00000134986 | NREP | neuronal regeneration related protein homolog (rat) | 5 | q22.1 |
| ENSG00000164113 | ADAD1 | adenosine deaminase domain containing 1 (testis-specific) | 4 | q27 |
| ENSG00000184226 | PCDH9 | protocadherin 9 | 13 | q21.32 |
| ENSG00000109471 | IL2 | interleukin 2 | 4 | q27 |
| ENSG00000139625 | MAP3K12 | mitogen-activated protein kinase kinase kinase 12 | 12 | q13.13 |
| ENSG00000130309 | GLT25D1 | glycosyltransferase 25 domain containing 1 | 19 | p13.11 |
| ENSG00000109107 | ALDOC | aldolase C, fructose-bisphosphate | 17 | q11.2 |
| ENSG00000139546 | TARBP2 | TAR (HIV-1) RNA binding protein 2 | 12 | q13.13 |
| ENSG00000130477 | UNC13A | unc-13 homolog A (C. elegans) | 19 | p13.11 |
| ENSG00000138684 | IL21 | interleukin 21 | 4 | q27 |
| ENSG00000134982 | APC | adenomatous polyposis coli | 5 | q22.2 |
| ENSG00000130479 | MAP1S | microtubule-associated protein 1S | 19 | p13.11 |
| ENSG00000138685 | FGF2 | fibroblast growth factor 2 (basic) | 4 | q28.1 |
| ENSG00000138028 | CGREF1 | cell growth regulator with EF-hand domain 1 | 2 | p23.3 |
| ENSG00000130475 | FCHO1 | FCH domain only 1 | 19 | p13.11 |
| ENSG00000131808 | FSHB | follicle stimulating hormone, beta polypeptide | 11 | p14.1 |
| ENSG00000169398 | PTK2 | PTK2 protein tyrosine kinase 2 | 8 | q24.3 |
| ENSG00000101255 | TRIB3 | tribbles homolog 3 (Drosophila) | 20 | p13 |
| ENSG00000114737 | CISH | cytokine inducible SH2-containing protein | 3 | p21.2 |
| ENSG00000132581 | SDF2 | stromal cell-derived factor 2 | 17 | q11.2 |
| ENSG00000105639 | JAK3 | Janus kinase 3 | 19 | p13.11 |
| ENSG00000125826 | RBCK1 | RanBP-type and C3HC4-type zinc finger containing 1 | 20 | p13 |
| ENSG00000088538 | DOCK3 | dedicator of cytokinesis 3 | 3 | p21.2 |
| ENSG00000196159 | FAT4 | FAT tumor suppressor homolog 4 (Drosophila) | 4 | q28.1 |
| ENSG00000105974 | CAV1 | caveolin 1, caveolae protein, 22kDa | 7 | q31.2 |
| ENSG00000109113 | RAB34 | RAB34, member RAS oncogene family | 17 | q11.2 |
| ENSG00000145041 | VPRBP | Vpr (HIV-1) binding protein | 3 | p21.2 |
| ENSG00000142731 | PLK4 | polo-like kinase 4 | 4 | q28.1 |
| ENSG00000096996 | IL12RB1 | interleukin 12 receptor, beta 1 | 19 | p13.11 |
| ENSG00000181790 | BAI1 | brain-specific angiogenesis inhibitor 1 | 8 | q24.3 |
| ENSG00000105989 | WNT2 | wingless-type MMTV integration site family member 2 | 7 | q31.2 |
| ENSG00000198576 | ARC | activity-regulated cytoskeleton-associated protein | 8 | q24.3 |
| ENSG00000076604 | TRAF4 | TNF receptor-associated factor 4 | 17 | q11.2 |
| ENSG00000205777 | GAGE1 | G antigen 1 | X | p11.23 |
| ENSG00000164219 | PGGT1B | protein geranylgeranyltransferase type I, beta subunit | 5 | q22.3 |
| ENSG00000205220 | PSMB10 | proteasome (prosome, macropain) subunit, beta type, 10 | 16 | q22.1 |
| ENSG00000001626 | CFTR | cystic fibrosis transmembrane conductance regulator (ATP-binding cassette sub-family C, member 7) | 7 | q31.2 |
| ENSG00000105647 | PIK3R2 | phosphoinositide-3-kinase, regulatory subunit 2 (beta) | 19 | p13.11 |
| ENSG00000160886 | LY6K | lymphocyte antigen 6 complex, locus K | 8 | q24.3 |
| ENSG00000126233 | SLURP1 | secreted LY6/PLAUR domain containing 1 | 8 | q24.3 |
| ENSG00000123405 | NFE2 | nuclear factor (erythroid-derived 2), 45kDa | 12 | q13.13 |
| ENSG00000105649 | RAB3A | RAB3A, member RAS oncogene family | 19 | p13.11 |
| ENSG00000132589 | FLOT2 | flotillin 2 | 17 | q11.2 |
| ENSG00000167656 | LY6D | lymphocyte antigen 6 complex, locus D | 8 | q24.3 |
| ENSG00000077684 | PHF17 | PHD finger protein 17 | 4 | q28.2 |
| ENSG00000104499 | GML | glycosylphosphatidylinositol anchored molecule like | 8 | q24.3 |
| ENSG00000161638 | ITGA5 | integrin, alpha 5 (fibronectin receptor, alpha polypeptide) | 12 | q13.13 |
| ENSG00000184937 | WT1 | Wilms tumor 1 | 11 | p13 |
| ENSG00000138650 | PCDH10 | protocadherin 10 | 4 | q28.3 |
| ENSG00000072736 | NFATC3 | nuclear factor of activated T-cells, cytoplasmic, calcineurin-dependent 3 | 16 | q22.1 |
| ENSG00000196535 | MYO18A | myosin XVIIIA | 17 | q11.2 |
| ENSG00000130513 | GDF15 | growth differentiation factor 15 | 19 | p13.11 |
| ENSG00000189184 | PCDH18 | protocadherin 18 | 4 | q28.3 |
| ENSG00000160932 | LY6E | lymphocyte antigen 6 complex, locus E | 8 | q24.3 |
| ENSG00000071243 | ING3 | inhibitor of growth family, member 3 | 7 | q31.31 |
| ENSG00000092421 | SEMA6A | sema domain, transmembrane domain (TM), and cytoplasmic domain, (semaphorin) 6A | 5 | q23.1 |
| ENSG00000123360 | PDE1B | phosphodiesterase 1B, calmodulin-dependent | 12 | q13.2 |
| ENSG00000176956 | LY6H | lymphocyte antigen 6 complex, locus H | 8 | q24.3 |
| ENSG00000135447 | PPP1R1A | protein phosphatase 1, regulatory (inhibitor) subunit 1A | 12 | q13.2 |
| ENSG00000163794 | UCN | urocortin | 2 | p23.3 |
| ENSG00000164087 | POC1A | POC1 centriolar protein homolog A (Chlamydomonas) | 3 | p21.2 |
| ENSG00000173366 | TLR9 | TLR9 | 3 | p21.2 |
| ENSG00000196937 | FAM3C | family with sequence similarity 3, member C | 7 | q31.31 |
| ENSG00000105701 | FKBP8 | FK506 binding protein 8, 38kDa | 19 | p13.11 |
| ENSG00000145779 | TNFAIP8 | tumor necrosis factor, alpha-induced protein 8 | 5 | q23.1 |
| ENSG00000062038 | CDH3 | cadherin 3, type 1, P-cadherin (placental) | 16 | q22.1 |
| ENSG00000081803 | CADPS2 | Ca++-dependent secretion activator 2 | 7 | q31.32 |
| ENSG00000006016 | CRLF1 | cytokine receptor-like factor 1 | 19 | p13.11 |
| ENSG00000039068 | CDH1 | cadherin 1, type 1, E-cadherin (epithelial) | 16 | q22.1 |
| ENSG00000110422 | HIPK3 | homeodomain interacting protein kinase 3 | 11 | p13 |
| ENSG00000151304 | SRFBP1 | serum response factor binding protein 1 | 5 | q23.1 |
| ENSG00000136153 | LMO7 | LIM domain 7 | 13 | q22.2 |
| ENSG00000101280 | ANGPT4 | angiopoietin 4 | 20 | p13 |
| ENSG00000110427 | KIAA1549L | KIAA1549-like | 11 | p13 |
| ENSG00000115234 | SNX17 | sorting nexin 17 | 2 | p23.3 |
| ENSG00000135424 | ITGA7 | integrin, alpha 7 | 12 | q13.2 |
| ENSG00000085063 | CD59 | CD59 molecule, complement regulatory protein | 11 | p13 |
| ENSG00000105664 | COMP | cartilage oligomeric matrix protein | 19 | p13.11 |
| ENSG00000125818 | PSMF1 | proteasome (prosome, macropain) inhibitor subunit 1 (PI31) | 20 | p13 |
| ENSG00000179387 | ELMOD2 | ELMO/CED-12 domain containing 2 | 4 | q31.1 |
| ENSG00000104522 | TSTA3 | tissue specific transplantation antigen P35B | 8 | q24.3 |
| ENSG00000109424 | UCP1 | uncoupling protein 1 (mitochondrial, proton carrier) | 4 | q31.1 |
| ENSG00000205302 | SNX2 | sorting nexin 2 | 5 | q23.2 |
| ENSG00000135441 | BLOC1S1 | biogenesis of lysosomal organelles complex-1, subunit 1 | 12 | q13.2 |
| ENSG00000182271 | TMIGD1 | transmembrane and immunoglobulin domain containing 1 | 17 | q11.2 |
| ENSG00000010319 | SEMA3G | sema domain, immunoglobulin domain (Ig), short basic domain, secreted, (semaphorin) 3G | 3 | p21.1 |
| ENSG00000130283 | GDF1 | growth differentiation factor 1 | 19 | p13.11 |
| ENSG00000135404 | CD63 | CD63 molecule | 12 | q13.2 |
| ENSG00000132612 | VPS4A | vacuolar protein sorting 4 homolog A (S. cerevisiae) | 16 | q22.1 |
| ENSG00000168938 | PPIC | peptidylprolyl isomerase C (cyclophilin C) | 5 | q23.2 |
| ENSG00000164136 | IL15 | interleukin 15 | 4 | q31.21 |
| ENSG00000106304 | SPAM1 | sperm adhesion molecule 1 (PH-20 hyaluronidase, zona pellucida binding) | 7 | q31.32 |
| ENSG00000010322 | NISCH | nischarin | 3 | p21.1 |
| ENSG00000135414 | GDF11 | growth differentiation factor 11 | 12 | q13.2 |
| ENSG00000180900 | SCRIB | scribbled homolog (Drosophila) | 8 | q24.3 |
| ENSG00000101298 | SNPH | syntaphilin | 20 | p13 |
| ENSG00000010327 | STAB1 | stabilin 1 | 3 | p21.1 |
| ENSG00000176390 | CRLF3 | cytokine receptor-like factor 3 | 17 | q11.2 |
| ENSG00000121691 | CAT | catalase | 11 | p13 |
| ENSG00000102908 | NFAT5 | nuclear factor of activated T-cells 5, tonicity-responsive | 16 | q22.1 |
| ENSG00000179950 | PUF60 | poly-U binding splicing factor 60KDa | 8 | q24.3 |
| ENSG00000102794 | IRG1 | immunoresponsive 1 homolog (mouse) | 13 | q22.3 |
| ENSG00000185189 | NRBP2 | nuclear receptor binding protein 2 | 8 | q24.3 |
| ENSG00000205755 | CRLF2 | cytokine receptor-like factor 2 | X | p22.33 |
| ENSG00000181481 | RNF135 | ring finger protein 135 | 17 | q11.2 |
| ENSG00000106328 | FSCN3 | fascin homolog 3, actin-bundling protein, testicular (Strongylocentrotus purpuratus) | 7 | q32.1 |
| ENSG00000065357 | DGKA | diacylglycerol kinase, alpha 80kDa | 12 | q13.2 |
| ENSG00000198223 | CSF2RA | colony stimulating factor 2 receptor, alpha, low-affinity (granulocyte-macrophage) | X | p22.33 |
| ENSG00000181019 | NQO1 | NAD(P)H dehydrogenase, quinone 1 | 16 | q22.1 |
| ENSG00000064545 | TMEM161A | transmembrane protein 161A | 19 | p13.11 |
| ENSG00000106331 | PAX4 | paired box 4 | 7 | q32.1 |
| ENSG00000183090 | FREM3 | FRAS1 related extracellular matrix 3 | 4 | q31.21 |
| ENSG00000196712 | NF1 | neurofibromin 1 | 17 | q11.2 |
| ENSG00000197465 | GYPE | glycophorin E (MNS blood group) | 4 | q31.21 |
| ENSG00000149089 | APIP | APAF1 interacting protein | 11 | p13 |
| ENSG00000088832 | FKBP1A | FK506 binding protein 1A, 12kDa | 20 | p13 |
| ENSG00000173926 | MARCH3 | membrane-associated ring finger (C3HC4) 3, E3 ubiquitin protein ligase | 5 | q23.2 |
| ENSG00000198373 | WWP2 | WW domain containing E3 ubiquitin protein ligase 2 | 16 | q22.1 |
| ENSG00000026508 | CD44 | CD44 molecule (Indian blood group) | 11 | p13 |
| ENSG00000145794 | MEGF10 | multiple EGF-like-domains 10 | 5 | q23.2 |
| ENSG00000174697 | LEP | leptin | 7 | q32.1 |
| ENSG00000170180 | GYPA | glycophorin A (MNS blood group) | 4 | q31.21 |
| ENSG00000184162 | NR2C2AP | nuclear receptor 2C2-associated protein | 19 | p13.11 |
| ENSG00000138002 | IFT172 | intraflagellar transport 172 homolog (Chlamydomonas) | 2 | p23.3 |
| ENSG00000130287 | NCAN | neurocan | 19 | p13.11 |
| ENSG00000126861 | OMG | oligodendrocyte myelin glycoprotein | 17 | q11.2 |
| ENSG00000111540 | RAB5B | RAB5B, member RAS oncogene family | 12 | q13.2 |
| ENSG00000187664 | HAPLN4 | hyaluronan and proteoglycan link protein 4 | 19 | p13.11 |
| ENSG00000131242 | RAB11FIP4 | RAB11 family interacting protein 4 (class II) | 17 | q11.2 |
| ENSG00000055957 | ITIH1 | inter-alpha-trypsin inhibitor heavy chain 1 | 3 | p21.1 |
| ENSG00000185291 | IL3RA | interleukin 3 receptor, alpha (low affinity) | X | p22.33 |
| ENSG00000055955 | ITIH4 | inter-alpha-trypsin inhibitor heavy chain family, member 4 | 3 | p21.1 |
| ENSG00000169100 | SLC25A6 | solute carrier family 25 (mitochondrial carrier; adenine nucleotide translocator), member 6 | X | p22.33 |
| ENSG00000110436 | SLC1A2 | solute carrier family 1 (glial high affinity glutamate transporter), member 2 | 11 | p13 |
| ENSG00000065361 | ERBB3 | v-erb-b2 erythroblastic leukemia viral oncogene homolog 3 (avian) | 12 | q13.2 |
| ENSG00000126858 | RHOT1 | ras homolog family member T1 | 17 | q11.2 |
| ENSG00000170365 | SMAD1 | SMAD family member 1 | 4 | q31.21 |
| ENSG00000186010 | NDUFA13 | NADH dehydrogenase (ubiquinone) 1 alpha subcomplex, 13 | 19 | p13.11 |
| ENSG00000105717 | PBX4 | pre-B-cell leukemia homeobox 4 | 19 | p13.11 |
| ENSG00000163932 | PRKCD | protein kinase C, delta | 3 | p21.1 |
| ENSG00000175104 | TRAF6 | TNF receptor-associated factor 6, E3 ubiquitin protein ligase | 11 | p12 |
| ENSG00000166349 | RAG1 | recombination activating gene 1 | 11 | p12 |
| ENSG00000108671 | PSMD11 | proteasome (prosome, macropain) 26S subunit, non-ATPase, 11 | 17 | q11.2 |
| ENSG00000175097 | RAG2 | recombination activating gene 2 | 11 | p12 |
| ENSG00000176749 | CDK5R1 | cyclin-dependent kinase 5, regulatory subunit 1 (p35) | 17 | q11.2 |
| ENSG00000197976 | AKAP17A | A kinase (PRKA) anchor protein 17A | X | p22.33 |
| ENSG00000151617 | EDNRA | endothelin receptor type A | 4 | q31.22 |
| ENSG00000166181 | API5 | apoptosis inhibitor 5 | 11 | p12 |
| ENSG00000181852 | RNF41 | ring finger protein 41 | 12 | q13.3 |
| ENSG00000169567 | HINT1 | histidine triad nucleotide binding protein 1 | 5 | q23.3 |
| ENSG00000157368 | IL34 | interleukin 34 | 16 | q22.1 |
| ENSG00000141316 | SPACA3 | sperm acrosome associated 3 | 17 | q11.2 |
| ENSG00000136160 | EDNRB | endothelin receptor type B | 13 | q22.3 |
| ENSG00000128604 | IRF5 | interferon regulatory factor 5 | 7 | q32.1 |
| ENSG00000158985 | CDC42SE2 | CDC42 small effector 2 | 5 | q31.1 |
| ENSG00000056736 | IL17RB | interleukin 17 receptor B | 3 | p21.1 |
| ENSG00000185122 | HSF1 | heat shock transcription factor 1 | 8 | q24.3 |
| ENSG00000108691 | CCL2 | chemokine (C-C motif) ligand 2 | 17 | q12 |
| ENSG00000089012 | SIRPG | signal-regulatory protein gamma | 20 | p13 |
| ENSG00000108688 | CCL7 | chemokine (C-C motif) ligand 7 | 17 | q12 |
| ENSG00000152192 | POU4F1 | POU class 4 homeobox 1 | 13 | q31.1 |
| ENSG00000172156 | CCL11 | chemokine (C-C motif) ligand 11 | 17 | q12 |
| ENSG00000128602 | SMO | smoothened, frizzled family receptor | 7 | q32.1 |
| ENSG00000108700 | CCL8 | chemokine (C-C motif) ligand 8 | 17 | q12 |
| ENSG00000198053 | SIRPA | signal-regulatory protein alpha | 20 | p13 |
| ENSG00000181374 | CCL13 | chemokine (C-C motif) ligand 13 | 17 | q12 |
| ENSG00000114251 | WNT5A | wingless-type MMTV integration site family, member 5A | 3 | p14.3 |
| ENSG00000158019 | BRE | brain and reproductive organ-expressed (TNFRSF1A modulator) | 2 | p23.2 |
| ENSG00000108702 | CCL1 | chemokine (C-C motif) ligand 1 | 17 | q12 |
| ENSG00000002586 | CD99 | CD99 molecule | X | p22.33 |
| ENSG00000052850 | ALX4 | ALX homeobox 4 | 11 | p11.2 |
| ENSG00000172687 | ZNF738 | zinc finger protein 738 | 19 | p12 |
| ENSG00000085117 | CD82 | CD82 molecule | 11 | p11.2 |
| ENSG00000110944 | IL23A | interleukin 23, alpha subunit p19 | 12 | q13.3 |
| ENSG00000170581 | STAT2 | signal transducer and activator of transcription 2, 113kDa | 12 | q13.3 |
| ENSG00000163947 | ARHGEF3 | Rho guanine nucleotide exchange factor (GEF) 3 | 3 | p14.3 |
| ENSG00000124343 | XG | Xg blood group | X | p22.33 |
| ENSG00000160949 | TONSL | tonsoku-like, DNA repair protein | 8 | q24.3 |
| ENSG00000140835 | CHST4 | carbohydrate (N-acetylglucosamine 6-O) sulfotransferase 4 | 16 | q22.2 |
| ENSG00000091732 | ZC3HC1 | zinc finger, C3HC-type containing 1 | 7 | q32.2 |
| ENSG00000144730 | IL17RD | interleukin 17 receptor D | 3 | p14.3 |
| ENSG00000145425 | RPS3A | ribosomal protein S3A | 4 | q31.3 |
| ENSG00000092871 | RFFL | ring finger and FYVE-like domain containing E3 ubiquitin protein ligase | 17 | q12 |
| ENSG00000164399 | IL3 | interleukin 3 (colony-stimulating factor, multiple) | 5 | q31.1 |
| ENSG00000160973 | FOXH1 | forkhead box H1 | 8 | q24.3 |
| ENSG00000166747 | AP1G1 | adaptor-related protein complex 1, gamma 1 subunit | 16 | q22.2 |
| ENSG00000121653 | MAPK8IP1 | mitogen-activated protein kinase 8 interacting protein 1 | 11 | p11.2 |
| ENSG00000164400 | CSF2 | colony stimulating factor 2 (granulocyte-macrophage) | 5 | q31.1 |
| ENSG00000165905 | GYLTL1B | glycosyltransferase-like 1B | 11 | p11.2 |
| ENSG00000110955 | ATP5B | ATP synthase, H+ transporting, mitochondrial F1 complex, beta polypeptide | 12 | q13.3 |
| ENSG00000102967 | DHODH | dihydroorotate dehydrogenase (quinone) | 16 | q22.2 |
| ENSG00000197372 | ZNF675 | zinc finger protein 675 | 19 | p12 |
| ENSG00000110958 | PTGES3 | prostaglandin E synthase 3 (cytosolic) | 12 | q13.3 |
| ENSG00000149091 | DGKZ | diacylglycerol kinase, zeta | 11 | p11.2 |
| ENSG00000158623 | COPG2 | coatomer protein complex, subunit gamma 2 | 7 | q32.2 |
| ENSG00000166750 | SLFN5 | schlafen family member 5 | 17 | q12 |
| ENSG00000172716 | SLFN11 | schlafen family member 11 | 17 | q12 |
| ENSG00000172123 | SLFN12 | schlafen family member 12 | 17 | q12 |
| ENSG00000146938 | NLGN4X | neuroligin 4, X-linked | X | p22.31 |
| ENSG00000154760 | SLFN13 | schlafen family member 13 | 17 | q12 |
| ENSG00000205045 | SLFN12L | schlafen family member 12-like | 17 | q12 |
| ENSG00000166289 | PLEKHF1 | pleckstrin homology domain containing, family F (with FYVE domain) member 1 | 19 | q12 |
| ENSG00000137462 | TLR2 | toll-like receptor 2 | 4 | q31.3 |
| ENSG00000128567 | PODXL | podocalyxin-like | 7 | q32.3 |
| ENSG00000145423 | SFRP2 | secreted frizzled-related protein 2 | 4 | q31.3 |
| ENSG00000105173 | CCNE1 | cyclin E1 | 19 | q12 |
| ENSG00000197410 | DCHS2 | dachsous 2 (Drosophila) | 4 | q31.3 |
| ENSG00000103035 | PSMD7 | proteasome (prosome, macropain) 26S subunit, non-ATPase, 7 | 16 | q23.1 |
| ENSG00000131558 | EXOC4 | exocyst complex component 4 | 7 | q33 |
| ENSG00000163687 | DNASE1L3 | deoxyribonuclease I-like 3 | 3 | p14.3 |
| ENSG00000171564 | FGB | fibrinogen beta chain | 4 | q31.3 |
| ENSG00000180210 | F2 | coagulation factor II (thrombin) | 11 | p11.2 |
| ENSG00000171094 | ALK | anaplastic lymphoma receptor tyrosine kinase | 2 | p23.1 |
| ENSG00000171560 | FGA | fibrinogen alpha chain | 4 | q31.3 |
| ENSG00000166888 | STAT6 | signal transducer and activator of transcription 6, interleukin-4 induced | 12 | q13.3 |
| ENSG00000125347 | IRF1 | interferon regulatory factor 1 | 5 | q31.1 |
| ENSG00000171557 | FGG | fibrinogen gamma chain | 4 | q32.1 |
| ENSG00000123384 | LRP1 | low density lipoprotein receptor-related protein 1 | 12 | q13.3 |
| ENSG00000105185 | PDCD5 | programmed cell death 5 | 19 | q13.11 |
| ENSG00000113525 | IL5 | interleukin 5 (colony-stimulating factor, eosinophil) | 5 | q31.1 |
| ENSG00000011201 | KAL1 | Kallmann syndrome 1 sequence | X | p22.31 |
| ENSG00000198643 | FAM3D | family with sequence similarity 3, member D | 3 | p14.2 |
| ENSG00000050820 | BCAR1 | breast cancer anti-estrogen resistance 1 | 16 | q23.1 |
| ENSG00000134569 | LRP4 | low density lipoprotein receptor-related protein 4 | 11 | p11.2 |
| ENSG00000153774 | CFDP1 | craniofacial development protein 1 | 16 | q23.1 |
| ENSG00000125257 | ABCC4 | ATP-binding cassette, sub-family C (CFTR/MRP), member 4 | 13 | q32.1 |
| ENSG00000145431 | PDGFC | platelet derived growth factor C | 4 | q32.1 |
| ENSG00000175189 | INHBC | inhibin, beta C | 12 | q13.3 |
| ENSG00000130881 | LRP3 | low density lipoprotein receptor-related protein 3 | 19 | q13.11 |
| ENSG00000169194 | IL13 | interleukin 13 | 5 | q31.1 |
| ENSG00000153266 | FEZF2 | FEZ family zinc finger 2 | 3 | p14.2 |
| ENSG00000153879 | CEBPG | CCAAT/enhancer binding protein (C/EBP), gamma | 19 | q13.11 |
| ENSG00000065457 | ADAT1 | adenosine deaminase, tRNA-specific 1 | 16 | q23.1 |
| ENSG00000163618 | CADPS | Ca++-dependent secretion activator | 3 | p14.2 |
| ENSG00000113520 | IL4 | interleukin 4 | 5 | q31.1 |
| ENSG00000134873 | CLDN10 | claudin 10 | 13 | q32.1 |
| ENSG00000165912 | PACSIN3 | protein kinase C and casein kinase substrate in neurons 3 | 11 | p11.2 |
| ENSG00000175197 | DDIT3 | DNA-damage-inducible transcript 3 | 12 | q13.3 |
| ENSG00000152910 | CNTNAP4 | contactin associated protein-like 4 | 16 | q23.1 |
| ENSG00000184492 | FOXD4L1 | forkhead box D4-like 1 | 2 | q14.1 |
| ENSG00000025434 | NR1H3 | nuclear receptor subfamily 1, group H, member 3 | 11 | p11.2 |
| ENSG00000105220 | GPI | glucose-6-phosphate isomerase | 19 | q13.11 |
| ENSG00000126249 | PDCD2L | programmed cell death 2-like | 19 | q13.11 |
| ENSG00000110514 | MADD | MAP-kinase activating death domain | 11 | p11.2 |
| ENSG00000157680 | DGKI | diacylglycerol kinase, iota | 7 | q33 |
| ENSG00000171497 | PPID | peptidylprolyl isomerase D | 4 | q32.1 |
| ENSG00000162959 | MEMO1 | mediator of cell motility 1 | 2 | p22.3 |
| ENSG00000163636 | PSMD6 | proteasome (prosome, macropain) 26S subunit, non-ATPase, 6 | 3 | p14.1 |
| ENSG00000140876 | NUDT7 | nudix (nucleoside diphosphate linked moiety X)-type motif 7 | 16 | q23.1 |
| ENSG00000102580 | DNAJC3 | DnaJ (Hsp40) homolog, subfamily C, member 3 | 13 | q32.1 |
| ENSG00000186153 | WWOX | WW domain containing oxidoreductase | 16 | q23.1 |
| ENSG00000021574 | SPAST | spastin | 2 | p22.3 |
| ENSG00000134571 | MYBPC3 | myosin binding protein C, cardiac | 11 | p11.2 |
| ENSG00000164129 | NPY5R | neuropeptide Y receptor Y5 | 4 | q32.2 |
| ENSG00000066336 | SPI1 | spleen focus forming virus (SFFV) proviral integration oncogene spi1 | 11 | p11.2 |
| ENSG00000151276 | MAGI1 | membrane associated guanylate kinase, WW and PDZ domain containing 1 | 3 | p14.1 |
| ENSG00000165916 | PSMC3 | proteasome (prosome, macropain) 26S subunit, ATPase, 3 | 11 | p11.2 |
| ENSG00000091106 | NLRC4 | NLR family, CARD domain containing 4 | 2 | p22.3 |
| ENSG00000107099 | DOCK8 | dedicator of cytokinesis 8 | 9 | p24.3 |
| ENSG00000088881 | EBF4 | early B-cell factor 4 | 20 | p13 |
| ENSG00000164404 | GDF9 | growth differentiation factor 9 | 5 | q31.1 |
| ENSG00000115760 | BIRC6 | baculoviral IAP repeat containing 6 | 2 | p22.3 |
| ENSG00000144749 | LRIG1 | leucine-rich repeats and immunoglobulin-like domains 1 | 3 | p14.1 |
| ENSG00000089327 | FXYD5 | FXYD domain containing ion transport regulator 5 | 19 | q13.12 |
| ENSG00000135446 | CDK4 | cyclin-dependent kinase 4 | 12 | q14.1 |
| ENSG00000164406 | LEAP2 | liver expressed antimicrobial peptide 2 | 5 | q31.1 |
| ENSG00000197943 | PLCG2 | phospholipase C, gamma 2 (phosphatidylinositol-specific) | 16 | q23.3 |
| ENSG00000064393 | HIPK2 | homeodomain interacting protein kinase 2 | 7 | q34 |
| ENSG00000172247 | C1QTNF4 | C1q and tumor necrosis factor related protein 4 | 11 | p11.2 |
| ENSG00000111012 | CYP27B1 | cytochrome P450, family 27, subfamily B, polypeptide 1 | 12 | q14.1 |
| ENSG00000183662 | FAM19A1 | family with sequence similarity 19 (chemokine (C-C motif)-like), member A1 | 3 | p14.1 |
| ENSG00000049323 | LTBP1 | latent transforming growth factor beta binding protein 1 | 2 | p22.3 |
| ENSG00000163377 | FAM19A4 | family with sequence similarity 19 (chemokine (C-C motif)-like), member A4 | 3 | p14.1 |
| ENSG00000125363 | AMELX | amelogenin, X-linked | X | p22.2 |
| ENSG00000088882 | CPXM1 | carboxypeptidase X (M14 family), member 1 | 20 | p13 |
| ENSG00000059377 | TBXAS1 | thromboxane A synthase 1 (platelet) | 7 | q34 |
| ENSG00000037897 | METTL1 | methyltransferase like 1 | 12 | q14.1 |
| ENSG00000140945 | CDH13 | cadherin 13, H-cadherin (heart) | 16 | q23.3 |
| ENSG00000105697 | HAMP | hepcidin antimicrobial peptide | 19 | q13.12 |
| ENSG00000105695 | MAG | myelin associated glycoprotein | 19 | q13.12 |
| ENSG00000012124 | CD22 | CD22 molecule | 19 | q13.12 |
| ENSG00000187098 | MITF | microphthalmia-associated transcription factor | 3 | p13 |
| ENSG00000154447 | SH3RF1 | SH3 domain containing ring finger 1 | 4 | q33 |
| ENSG00000139263 | LRIG3 | leucine-rich repeats and immunoglobulin-like domains 3 | 12 | q14.1 |
| ENSG00000196664 | TLR7 | toll-like receptor 7 | X | p22.2 |
| ENSG00000198673 | FAM19A2 | family with sequence similarity 19 (chemokine (C-C motif)-like), member A2 | 12 | q14.1 |
| ENSG00000101916 | TLR8 | toll-like receptor 8 | X | p22.2 |
| ENSG00000171055 | FEZ2 | fasciculation and elongation protein zeta 2 (zygin II) | 2 | p22.2 |
| ENSG00000157764 | BRAF | v-raf murine sarcoma viral oncogene homolog B1 | 7 | q34 |
| ENSG00000114861 | FOXP1 | forkhead box P1 | 3 | p13 |
| ENSG00000081059 | TCF7 | transcription factor 7 (T-cell specific, HMG-box) | 5 | q31.1 |
| ENSG00000006530 | AGK | acylglycerol kinase | 7 | q34 |
| ENSG00000140955 | ADAD2 | adenosine deaminase domain containing 2 | 16 | q24.1 |
| ENSG00000166148 | AVPR1A | arginine vasopressin receptor 1A | 12 | q14.2 |
| ENSG00000107937 | GTPBP4 | GTP binding protein 4 | 10 | p15.3 |
| ENSG00000164104 | HMGB2 | high mobility group box 2 | 4 | q34.1 |
| ENSG00000198759 | EGFL6 | EGF-like-domain, multiple 6 | X | p22.2 |
| ENSG00000103187 | COTL1 | coactosin-like 1 (Dictyostelium) | 16 | q24.1 |
| ENSG00000113575 | PPP2CA | protein phosphatase 2, catalytic subunit, alpha isozyme | 5 | q31.1 |
| ENSG00000163421 | PROK2 | prokineticin 2 | 3 | p13 |
| ENSG00000147852 | VLDLR | very low density lipoprotein receptor | 9 | p24.2 |
| ENSG00000163602 | RYBP | RING1 and YY1 binding protein | 3 | p13 |
| ENSG00000164107 | HAND2 | heart and neural crest derivatives expressed 2 | 4 | q34.1 |
| ENSG00000119048 | UBE2B | ubiquitin-conjugating enzyme E2B | 5 | q31.1 |
| ENSG00000055332 | EIF2AK2 | eukaryotic translation initiation factor 2-alpha kinase 2 | 2 | p22.2 |
| ENSG00000164120 | HPGD | hydroxyprostaglandin dehydrogenase 15-(NAD) | 4 | q34.1 |
| ENSG00000011590 | ZBTB32 | zinc finger and BTB domain containing 32 | 19 | q13.12 |
| ENSG00000183735 | TBK1 | TANK-binding kinase 1 | 12 | q14.2 |
| ENSG00000164615 | CAMLG | calcium modulating ligand | 5 | q31.1 |
| ENSG00000113805 | CNTN3 | contactin 3 (plasmacytoma associated) | 3 | p12.3 |
| ENSG00000165125 | TRPV6 | transient receptor potential cation channel, subfamily V, member 6 | 7 | q34 |
| ENSG00000205155 | PSENEN | presenilin enhancer 2 homolog (C. elegans) | 19 | q13.12 |
| ENSG00000174106 | LEMD3 | LEM domain containing 3 | 12 | q14.3 |
| ENSG00000140968 | IRF8 | interferon regulatory factor 8 | 16 | q24.1 |
| ENSG00000150628 | SPATA4 | spermatogenesis associated 4 | 4 | q34.2 |
| ENSG00000185736 | ADARB2 | adenosine deaminase, RNA-specific, B2 | 10 | p15.3 |
| ENSG00000101405 | OXT | oxytocin/neurophysin I prepropeptide | 20 | p13 |
| ENSG00000185008 | ROBO2 | roundabout, axon guidance receptor, homolog 2 (Drosophila) | 3 | p12.3 |
| ENSG00000197993 | KEL | Kell blood group, metallo-endopeptidase | 7 | q34 |
| ENSG00000069011 | PITX1 | paired-like homeodomain 1 | 5 | q31.1 |
| ENSG00000101200 | AVP | arginine vasopressin | 20 | p13 |
| ENSG00000080298 | RFX3 | regulatory factor X, 3 (influences HLA class II expression) | 9 | p24.2 |
| ENSG00000150630 | VEGFC | vascular endothelial growth factor C | 4 | q34.3 |
| ENSG00000176692 | FOXC2 | forkhead box C2 (MFH-1, mesenchyme forkhead 1) | 16 | q24.1 |
| ENSG00000169855 | ROBO1 | roundabout, axon guidance receptor, homolog 1 (Drosophila) | 3 | p12.3 |
| ENSG00000161270 | NPHS1 | nephrosis 1, congenital, Finnish type (nephrin) | 19 | q13.12 |
| ENSG00000115825 | PRKD3 | protein kinase D3 | 2 | p22.2 |
| ENSG00000109674 | NEIL3 | nei endonuclease VIII-like 3 (E. coli) | 4 | q34.3 |
| ENSG00000088387 | DOCK9 | dedicator of cytokinesis 9 | 13 | q32.3 |
| ENSG00000090376 | IRAK3 | interleukin-1 receptor-associated kinase 3 | 12 | q14.3 |
| ENSG00000067057 | PFKP | phosphofructokinase, platelet | 10 | p15.2 |
| ENSG00000125686 | MED1 | mediator complex subunit 1 | 17 | q12 |
| ENSG00000175161 | CADM2 | cell adhesion molecule 2 | 3 | p12.1 |
| ENSG00000145824 | CXCL14 | chemokine (C-X-C motif) ligand 14 | 5 | q31.1 |
| ENSG00000181544 | FANCB | Fanconi anemia, complementation group B | X | p22.2 |
| ENSG00000145839 | IL9 | interleukin 9 | 5 | q31.1 |
| ENSG00000126259 | KIRREL2 | kin of IRRE like 2 (Drosophila) | 19 | q13.12 |
| ENSG00000145826 | LECT2 | leukocyte cell-derived chemotaxin 2 | 5 | q31.1 |
| ENSG00000120708 | TGFBI | transforming growth factor, beta-induced, 68kDa | 5 | q31.1 |
| ENSG00000106144 | CASP2 | caspase 2, apoptosis-related cysteine peptidase | 7 | q34 |
| ENSG00000127334 | DYRK2 | dual-specificity tyrosine-(Y)-phosphorylation regulated kinase 2 | 12 | q15 |
| ENSG00000179021 | C3orf38 | chromosome 3 open reading frame 38 | 3 | p11.1 |
| ENSG00000105290 | APLP1 | amyloid beta (A4) precursor-like protein 1 | 19 | q13.12 |
| ENSG00000113658 | SMAD5 | SMAD family member 5 | 5 | q31.1 |
| ENSG00000111537 | IFNG | interferon, gamma | 12 | q15 |
| ENSG00000044524 | EPHA3 | EPH receptor A3 | 3 | p11.1 |
| ENSG00000177300 | CLDN22 | claudin 22 | 4 | q35.1 |
| ENSG00000167604 | NFKBID | nuclear factor of kappa light polypeptide gene enhancer in B-cells inhibitor, delta | 19 | q13.12 |
| ENSG00000106688 | SLC1A1 | solute carrier family 1 (neuronal/epithelial high affinity glutamate transporter, system Xag), member 1 | 9 | p24.2 |
| ENSG00000111536 | IL26 | interleukin 26 | 12 | q15 |
| ENSG00000168556 | ING2 | inhibitor of growth family, member 2 | 4 | q35.1 |
| ENSG00000011600 | TYROBP | TYRO protein tyrosine kinase binding protein | 19 | q13.12 |
| ENSG00000127318 | IL22 | interleukin 22 | 12 | q15 |
| ENSG00000141736 | ERBB2 | v-erb-b2 erythroblastic leukemia viral oncogene homolog 2, neuro/glioblastoma derived oncogene homolog (avian) | 17 | q12 |
| ENSG00000126243 | LRFN3 | leucine rich repeat and fibronectin type III domain containing 3 | 19 | q13.12 |
| ENSG00000152377 | SPOCK1 | sparc/osteonectin, cwcv and kazal-like domains proteoglycan (testican) 1 | 5 | q31.2 |
| ENSG00000165197 | FIGF | c-fos induced growth factor (vascular endothelial growth factor D) | X | p22.2 |
| ENSG00000159840 | ZYX | zyxin | 7 | q34 |
| ENSG00000169508 | GPR183 | G protein-coupled receptor 183 | 13 | q32.3 |
| ENSG00000124391 | IL17C | interleukin 17C | 16 | q24.2 |
| ENSG00000168310 | IRF2 | interferon regulatory factor 2 | 4 | q35.1 |
| ENSG00000051523 | CYBA | cytochrome b-245, alpha polypeptide | 16 | q24.2 |
| ENSG00000164305 | CASP3 | caspase 3, apoptosis-related cysteine peptidase | 4 | q35.1 |
| ENSG00000061492 | WNT8A | wingless-type MMTV integration site family, member 8A | 5 | q31.2 |
| ENSG00000156575 | PRG3 | proteoglycan 3 | 11 | q12.1 |
| ENSG00000112981 | NME5 | NME/NM23 family member 5 | 5 | q31.2 |
| ENSG00000130234 | ACE2 | angiotensin I converting enzyme (peptidyl-dipeptidase A) 2 | X | p22.2 |
| ENSG00000186652 | PRG2 | proteoglycan 2, bone marrow (natural killer cell activator, eosinophil granule major basic protein) | 11 | q12.1 |
| ENSG00000167914 | GSDMA | gasdermin A | 17 | q21.1 |
| ENSG00000067082 | KLF6 | Kruppel-like factor 6 | 10 | p15.2 |
| ENSG00000174469 | CNTNAP2 | contactin associated protein-like 2 | 7 | q35 |
| ENSG00000108344 | PSMD3 | proteasome (prosome, macropain) 26S subunit, non-ATPase, 3 | 17 | q21.1 |
| ENSG00000149131 | SERPING1 | serpin peptidase inhibitor, clade G (C1 inhibitor), member 1 | 11 | q12.1 |
| ENSG00000108342 | CSF3 | colony stimulating factor 3 (granulocyte) | 17 | q21.1 |
| ENSG00000055130 | CUL1 | cullin 1 | 7 | q36.1 |
| ENSG00000129993 | CBFA2T3 | core-binding factor, runt domain, alpha subunit 2; translocated to, 3 | 16 | q24.3 |
| ENSG00000182287 | AP1S2 | adaptor-related protein complex 1, sigma 2 subunit | X | p22.2 |
| ENSG00000096968 | JAK2 | Janus kinase 2 | 9 | p24.1 |
| ENSG00000057019 | DCBLD2 | discoidin, CUB and LCCL domain containing 2 | 3 | q12.1 |
| ENSG00000126351 | THRA | thyroid hormone receptor, alpha | 17 | q21.1 |
| ENSG00000198561 | CTNND1 | catenin (cadherin-associated protein), delta 1 | 11 | q12.1 |
| ENSG00000144810 | COL8A1 | collagen, type VIII, alpha 1 | 3 | q12.1 |
| ENSG00000176715 | ACSF3 | acyl-CoA synthetase family member 3 | 16 | q24.3 |
| ENSG00000120738 | EGR1 | early growth response 1 | 5 | q31.2 |
| ENSG00000129910 | CDH15 | cadherin 15, type 1, M-cadherin (myotubule) | 16 | q24.3 |
| ENSG00000113013 | HSPA9 | heat shock 70kDa protein 9 (mortalin) | 5 | q31.2 |
| ENSG00000115904 | SOS1 | son of sevenless homolog 1 (Drosophila) | 2 | p22.1 |
| ENSG00000164342 | TLR3 | toll-like receptor 3 | 4 | q35.1 |
| ENSG00000151632 | AKR1C2 | aldo-keto reductase family 1, member C2 (dihydrodiol dehydrogenase 2; bile acid binding protein; 3-alpha hydroxysteroid dehydrogenase, type III) | 10 | p15.1 |
| ENSG00000044115 | CTNNA1 | catenin (cadherin-associated protein), alpha 1, 102kDa | 5 | q31.2 |
| ENSG00000206535 | LNP1 | leukemia NUP98 fusion partner 1 | 3 | q12.2 |
| ENSG00000011566 | MAP4K3 | mitogen-activated protein kinase kinase kinase kinase 3 | 2 | p22.1 |
| ENSG00000131759 | RARA | retinoic acid receptor, alpha | 17 | q21.2 |
| ENSG00000120217 | CD274 | CD274 molecule | 9 | p24.1 |
| ENSG00000131747 | TOP2A | topoisomerase (DNA) II alpha 170kDa | 17 | q21.2 |
| ENSG00000197646 | PDCD1LG2 | programmed cell death 1 ligand 2 | 9 | p24.1 |
| ENSG00000197558 | SSPO | SCO-spondin homolog (Bos taurus) | 7 | q36.1 |
| ENSG00000088827 | SIGLEC1 | sialic acid binding Ig-like lectin 1, sialoadhesin | 20 | p13 |
| ENSG00000131746 | TNS4 | tensin 4 | 17 | q21.2 |
| ENSG00000110031 | LPXN | leupaxin | 11 | q12.1 |
| ENSG00000083857 | FAT1 | FAT tumor suppressor homolog 1 (Drosophila) | 4 | q35.2 |
| ENSG00000170476 | MZB1 | marginal zone B and B1 cell-specific protein | 5 | q31.2 |
| ENSG00000126353 | CCR7 | chemokine (C-C motif) receptor 7 | 17 | q21.2 |
| ENSG00000184584 | TMEM173 | transmembrane protein 173 | 5 | q31.2 |
| ENSG00000196139 | AKR1C3 | aldo-keto reductase family 1, member C3 (3-alpha hydroxysteroid dehydrogenase, type II) | 10 | p15.1 |
| ENSG00000166840 | GLYATL1 | glycine-N-acyltransferase-like 1 | 11 | q12.1 |
| ENSG00000198542 | ITGBL1 | integrin, beta-like 1 (with EGF-like repeat domains) | 13 | q33.1 |
| ENSG00000196329 | GIMAP5 | GTPase, IMAP family member 5 | 7 | q36.1 |
| ENSG00000173451 | THAP2 | THAP domain containing, apoptosis associated protein 2 | 12 | q21.1 |
| ENSG00000197629 | MPEG1 | macrophage expressed 1 | 11 | q12.1 |
| ENSG00000187741 | FANCA | Fanconi anemia, complementation group A | 16 | q24.3 |
| ENSG00000144802 | NFKBIZ | nuclear factor of kappa light polypeptide gene enhancer in B-cells inhibitor, zeta | 3 | q12.3 |
| ENSG00000011332 | DPF1 | D4, zinc and double PHD fingers family 1 | 19 | q13.2 |
| ENSG00000171431 | KRT20 | keratin 20 | 17 | q21.2 |
| ENSG00000137033 | IL33 | interleukin 33 | 9 | p24.1 |
| ENSG00000170017 | ALCAM | activated leukocyte cell adhesion molecule | 3 | q13.11 |
| ENSG00000114423 | CBLB | Cbl proto-oncogene, E3 ubiquitin protein ligase B | 3 | q13.11 |
| ENSG00000166889 | PATL1 | protein associated with topoisomerase II homolog 1 (yeast) | 11 | q12.1 |
| ENSG00000166900 | STX3 | syntaxin 3 | 11 | q12.1 |
| ENSG00000164867 | NOS3 | nitric oxide synthase 3 (endothelial cell) | 7 | q36.1 |
| ENSG00000099341 | PSMD8 | proteasome (prosome, macropain) 26S subunit, non-ATPase, 8 | 19 | q13.2 |
| ENSG00000196776 | CD47 | CD47 molecule | 3 | q13.12 |
| ENSG00000102104 | RS1 | retinoschisin 1 | X | p22.13 |
| ENSG00000178473 | UCN3 | urocortin 3 | 10 | p15.1 |
| ENSG00000114446 | IFT57 | intraflagellar transport 57 homolog (Chlamydomonas) | 3 | q13.13 |
| ENSG00000171777 | RASGRP4 | RAS guanyl releasing protein 4 | 19 | q13.2 |
| ENSG00000139289 | PHLDA1 | pleckstrin homology-like domain, family A, member 1 | 12 | q21.2 |
| ENSG00000149534 | MS4A2 | membrane-spanning 4-domains, subfamily A, member 2 | 11 | q12.1 |
| ENSG00000173848 | NET1 | neuroepithelial cell transforming 1 | 10 | p15.1 |
| ENSG00000164885 | CDK5 | cyclin-dependent kinase 5 | 7 | q36.1 |
| ENSG00000164896 | FASTK | Fas-activated serine/threonine kinase | 7 | q36.1 |
| ENSG00000134897 | BIVM | basic, immunoglobulin-like variable motif containing | 13 | q33.1 |
| ENSG00000088888 | MAVS | mitochondrial antiviral signaling protein | 20 | p13 |
| ENSG00000156738 | MS4A1 | membrane-spanning 4-domains, subfamily A, member 1 | 11 | q12.2 |
| ENSG00000163519 | TRAT1 | T cell receptor associated transmembrane adaptor 1 | 3 | q13.13 |
| ENSG00000170458 | CD14 | CD14 molecule | 5 | q31.3 |
| ENSG00000134899 | ERCC5 | excision repair cross-complementing rodent repair deficiency, complementation group 5 | 13 | q33.1 |
| ENSG00000113141 | IK | IK cytokine, down-regulator of HLA II | 5 | q31.3 |
| ENSG00000104814 | MAP4K1 | mitogen-activated protein kinase kinase kinase kinase 1 | 19 | q13.2 |
| ENSG00000067715 | SYT1 | synaptotagmin I | 12 | q21.2 |
| ENSG00000177707 | PVRL3 | poliovirus receptor-related 3 | 3 | q13.13 |
| ENSG00000177425 | PAWR | PRKC, apoptosis, WT1, regulator | 12 | q21.2 |
| ENSG00000183134 | PTGDR2 | prostaglandin D2 receptor 2 | 11 | q12.2 |
| ENSG00000130402 | ACTN4 | actinin, alpha 4 | 19 | q13.2 |
| ENSG00000153283 | CD96 | CD96 molecule | 3 | q13.13 |
| ENSG00000125255 | SLC10A2 | solute carrier family 10 (sodium/bile acid cotransporter family), member 2 | 13 | q33.1 |
| ENSG00000204970 | PCDHA1 | protocadherin alpha 1 | 5 | q31.3 |
| ENSG00000204969 | PCDHA2 | protocadherin alpha 2 | 5 | q31.3 |
| ENSG00000178934 | LGALS7B | lectin, galactoside-binding, soluble, 7B | 19 | q13.2 |
| ENSG00000013374 | NUB1 | negative regulator of ubiquitin-like proteins 1 | 7 | q36.1 |
| ENSG00000171747 | LGALS4 | lectin, galactoside-binding, soluble, 4 | 19 | q13.2 |
| ENSG00000204967 | PCDHA4 | protocadherin alpha 4 | 5 | q31.3 |
| ENSG00000125266 | EFNB2 | ephrin-B2 | 13 | q33.3 |
| ENSG00000204965 | PCDHA5 | protocadherin alpha 5 | 5 | q31.3 |
| ENSG00000139304 | PTPRQ | protein tyrosine phosphatase, receptor type, Q | 12 | q21.31 |
| ENSG00000013725 | CD6 | CD6 molecule | 11 | q12.2 |
| ENSG00000204963 | PCDHA7 | protocadherin alpha 7 | 5 | q31.3 |
| ENSG00000111052 | LIN7A | lin-7 homolog A (C. elegans) | 12 | q21.31 |
| ENSG00000204962 | PCDHA8 | protocadherin alpha 8 | 5 | q31.3 |
| ENSG00000106617 | PRKAG2 | protein kinase, AMP-activated, gamma 2 non-catalytic subunit | 7 | q36.1 |
| ENSG00000204961 | PCDHA9 | protocadherin alpha 9 | 5 | q31.3 |
| ENSG00000081842 | PCDHA6 | protocadherin alpha 6 | 5 | q31.3 |
| ENSG00000110448 | CD5 | CD5 molecule | 11 | q12.2 |
| ENSG00000174500 | GCSAM | germinal center-associated, signaling and motility | 3 | q13.2 |
| ENSG00000174405 | LIG4 | ligase IV, DNA, ATP-dependent | 13 | q33.3 |
| ENSG00000055609 | MLL3 | myeloid/lymphoid or mixed-lineage leukemia 3 | 7 | q36.1 |
| ENSG00000173801 | JUP | junction plakoglobin | 17 | q21.2 |
| ENSG00000104825 | NFKBIB | nuclear factor of kappa light polypeptide gene enhancer in B-cells inhibitor, beta | 19 | q13.2 |
| ENSG00000091972 | CD200 | CD200 molecule | 3 | q13.2 |
| ENSG00000171867 | PRNP | prion protein | 20 | p13 |
| ENSG00000171815 | PCDHB1 | protocadherin beta 1 | 5 | q31.3 |
| ENSG00000102524 | TNFSF13B | tumor necrosis factor (ligand) superfamily, member 13b | 13 | q33.3 |
| ENSG00000141756 | FKBP10 | FK506 binding protein 10, 65 kDa | 17 | q21.2 |
| ENSG00000186265 | BTLA | B and T lymphocyte associated | 3 | q13.2 |
| ENSG00000112852 | PCDHB2 | protocadherin beta 2 | 5 | q31.3 |
| ENSG00000113205 | PCDHB3 | protocadherin beta 3 | 5 | q31.3 |
| ENSG00000144848 | ATG3 | autophagy related 3 | 3 | q13.2 |
| ENSG00000081818 | PCDHB4 | protocadherin beta 4 | 5 | q31.3 |
| ENSG00000113209 | PCDHB5 | protocadherin beta 5 | 5 | q31.3 |
| ENSG00000147010 | SH3KBP1 | SH3-domain kinase binding protein 1 | X | p22.12 |
| ENSG00000113211 | PCDHB6 | protocadherin beta 6 | 5 | q31.3 |
| ENSG00000113212 | PCDHB7 | protocadherin beta 7 | 5 | q31.3 |
| ENSG00000206531 | CD200R1L | CD200 receptor 1-like | 3 | q13.2 |
| ENSG00000163606 | CD200R1 | CD200 receptor 1 | 3 | q13.2 |
| ENSG00000120322 | PCDHB8 | protocadherin beta 8 | 5 | q31.3 |
| ENSG00000196584 | XRCC2 | X-ray repair complementing defective repair in Chinese hamster cells 2 | 7 | q36.1 |
| ENSG00000179751 | SYCN | syncollin | 19 | q13.2 |
| ENSG00000120324 | PCDHB10 | protocadherin beta 10 | 5 | q31.3 |
| ENSG00000197110 | IL28B | interleukin 28B (interferon, lambda 3) | 19 | q13.2 |
| ENSG00000183709 | IL28A | interleukin 28A (interferon, lambda 2) | 19 | q13.2 |
| ENSG00000197479 | PCDHB11 | protocadherin beta 11 | 5 | q31.3 |
| ENSG00000182393 | IL29 | interleukin 29 (interferon, lambda 1) | 19 | q13.2 |
| ENSG00000120328 | PCDHB12 | protocadherin beta 12 | 5 | q31.3 |
| ENSG00000171132 | PRKCE | protein kinase C, epsilon | 2 | p21 |
| ENSG00000187372 | PCDHB13 | protocadherin beta 13 | 5 | q31.3 |
| ENSG00000144857 | BOC | Boc homolog (mouse) | 3 | q13.2 |
| ENSG00000185950 | IRS2 | insulin receptor substrate 2 | 13 | q34 |
| ENSG00000120327 | PCDHB14 | protocadherin beta 14 | 5 | q31.3 |
| ENSG00000011347 | SYT7 | synaptotagmin VII | 11 | q12.2 |
| ENSG00000146001 | PCDHB18 | protocadherin beta 18 pseudogene | 5 | q31.3 |
| ENSG00000187498 | COL4A1 | collagen, type IV, alpha 1 | 13 | q34 |
| ENSG00000113248 | PCDHB15 | protocadherin beta 15 | 5 | q31.3 |
| ENSG00000108773 | KAT2A | K(lysine) acetyltransferase 2A | 17 | q21.2 |
| ENSG00000128016 | ZFP36 | zinc finger protein 36, C3H type, homolog (mouse) | 19 | q13.2 |
| ENSG00000090924 | PLEKHG2 | pleckstrin homology domain containing, family G (with RhoGef domain) member 2 | 19 | q13.2 |
| ENSG00000204956 | PCDHGA1 | protocadherin gamma subfamily A, 1 | 5 | q31.3 |
| ENSG00000108774 | RAB5C | RAB5C, member RAS oncogene family | 17 | q21.2 |
| ENSG00000134470 | IL15RA | interleukin 15 receptor, alpha | 10 | p15.1 |
| ENSG00000049130 | KITLG | KIT ligand | 12 | q21.32 |
| ENSG00000081853 | PCDHGA2 | protocadherin gamma subfamily A, 2 | 5 | q31.3 |
| ENSG00000164690 | SHH | sonic hedgehog | 7 | q36.3 |
| ENSG00000161610 | HCRT | hypocretin (orexin) neuropeptide precursor | 17 | q21.2 |
| ENSG00000139318 | DUSP6 | dual specificity phosphatase 6 | 12 | q21.33 |
| ENSG00000173757 | STAT5B | signal transducer and activator of transcription 5B | 17 | q21.2 |
| ENSG00000105197 | TIMM50 | translocase of inner mitochondrial membrane 50 homolog (S. cerevisiae) | 19 | q13.2 |
| ENSG00000134460 | IL2RA | interleukin 2 receptor, alpha | 10 | p15.1 |
| ENSG00000139323 | POC1B | POC1 centriolar protein homolog B (Chlamydomonas) | 12 | q21.33 |
| ENSG00000126561 | STAT5A | signal transducer and activator of transcription 5A | 17 | q21.2 |
| ENSG00000171150 | SOCS5 | suppressor of cytokine signaling 5 | 2 | p21 |
| ENSG00000151577 | DRD3 | dopamine receptor D3 | 3 | q13.31 |
| ENSG00000176396 | EID2 | EP300 interacting inhibitor of differentiation 2 | 19 | q13.2 |
| ENSG00000168610 | STAT3 | signal transducer and activator of transcription 3 (acute-phase response factor) | 17 | q21.2 |
| ENSG00000171720 | HDAC3 | histone deacetylase 3 | 5 | q31.3 |
| ENSG00000009335 | UBE3C | ubiquitin protein ligase E3C | 7 | q36.3 |
| ENSG00000197948 | FCHSD1 | FCH and double SH3 domains 1 | 5 | q31.3 |
| ENSG00000149503 | INCENP | inner centromere protein antigens 135/155kDa | 11 | q12.3 |
| ENSG00000177189 | RPS6KA3 | ribosomal protein S6 kinase, 90kDa, polypeptide 3 | X | p22.12 |
| ENSG00000147869 | CER1 | cerberus 1, cysteine knot superfamily, homolog (Xenopus laevis) | 9 | p22.3 |
| ENSG00000119888 | EPCAM | epithelial cell adhesion molecule | 2 | p21 |
| ENSG00000105993 | DNAJB6 | DnaJ (Hsp40) homolog, subfamily B, member 6 | 7 | q36.3 |
| ENSG00000181847 | TIGIT | T cell immunoreceptor with Ig and ITIM domains | 3 | q13.31 |
| ENSG00000164946 | FREM1 | FRAS1 related extracellular matrix 1 | 9 | p22.3 |
| ENSG00000011465 | DCN | decorin | 12 | q21.33 |
| ENSG00000095002 | MSH2 | mutS homolog 2, colon cancer, nonpolyposis type 1 (E. coli) | 2 | p21 |
| ENSG00000156453 | PCDH1 | protocadherin 1 | 5 | q31.3 |
| ENSG00000172020 | GAP43 | growth associated protein 43 | 3 | q13.31 |
| ENSG00000149021 | SCGB1A1 | secretoglobin, family 1A, member 1 (uteroglobin) | 11 | q12.3 |
| ENSG00000185565 | LSAMP | limbic system-associated membrane protein | 3 | q13.31 |
| ENSG00000133639 | BTG1 | B-cell translocation gene 1, anti-proliferative | 12 | q21.33 |
| ENSG00000013275 | PSMC4 | proteasome (prosome, macropain) 26S subunit, ATPase, 4 | 19 | q13.2 |
| ENSG00000144847 | IGSF11 | immunoglobulin superfamily, member 11 | 3 | q13.32 |
| ENSG00000116062 | MSH6 | mutS homolog 6 (E. coli) | 2 | p16.3 |
| ENSG00000205057 | CLLU1OS | chronic lymphocytic leukemia up-regulated 1 opposite strand | 12 | q22 |
| ENSG00000113555 | PCDH12 | protocadherin 12 | 5 | q31.3 |
| ENSG00000205056 | CLLU1 | chronic lymphocytic leukemia up-regulated 1 | 12 | q22 |
| ENSG00000102606 | ARHGEF7 | Rho guanine nucleotide exchange factor (GEF) 7 | 13 | q34 |
| ENSG00000131507 | NDFIP1 | Nedd4 family interacting protein 1 | 5 | q31.3 |
| ENSG00000130758 | MAP3K10 | mitogen-activated protein kinase kinase kinase 10 | 19 | q13.2 |
| ENSG00000177889 | UBE2N | ubiquitin-conjugating enzyme E2N | 12 | q22 |
| ENSG00000113578 | FGF1 | fibroblast growth factor 1 (acidic) | 5 | q31.3 |
| ENSG00000105221 | AKT2 | v-akt murine thymoma viral oncogene homolog 2 | 19 | q13.2 |
| ENSG00000121594 | CD80 | CD80 molecule | 3 | q13.33 |
| ENSG00000120833 | SOCS2 | suppressor of cytokine signaling 2 | 12 | q22 |
| ENSG00000065675 | PRKCQ | protein kinase C, theta | 10 | p15.1 |
| ENSG00000149489 | ROM1 | retinal outer segment membrane protein 1 | 11 | q12.3 |
| ENSG00000169372 | CRADD | CASP2 and RIPK1 domain containing adaptor with death domain | 12 | q22 |
| ENSG00000136040 | PLXNC1 | plexin C1 | 12 | q22 |
| ENSG00000184451 | CCR10 | chemokine (C-C motif) receptor 10 | 17 | q21.2 |
| ENSG00000108797 | CNTNAP1 | contactin associated protein 1 | 17 | q21.2 |
| ENSG00000179915 | NRXN1 | neurexin 1 | 2 | p16.3 |
| ENSG00000162191 | UBXN1 | UBX domain protein 1 | 11 | q12.3 |
| ENSG00000131477 | RAMP2 | receptor (G protein-coupled) activity modifying protein 2 | 17 | q21.2 |
| ENSG00000082701 | GSK3B | glycogen synthase kinase 3 beta | 3 | q13.33 |
| ENSG00000126562 | WNK4 | WNK lysine deficient protein kinase 4 | 17 | q21.2 |
| ENSG00000158497 | HMHB1 | histocompatibility (minor) HB-1 | 5 | q31.3 |
| ENSG00000126581 | BECN1 | beclin 1, autophagy related | 17 | q21.31 |
| ENSG00000163430 | FSTL1 | follistatin-like 1 | 3 | q13.33 |
| ENSG00000068878 | PSME4 | proteasome (prosome, macropain) activator subunit 4 | 2 | p16.2 |
| ENSG00000168002 | POLR2G | polymerase (RNA) II (DNA directed) polypeptide G | 11 | q12.3 |
| ENSG00000131467 | PSME3 | proteasome (prosome, macropain) activator subunit 3 (PA28 gamma; Ki) | 17 | q21.31 |
| ENSG00000090006 | LTBP4 | latent transforming growth factor beta binding protein 4 | 19 | q13.2 |
| ENSG00000131471 | AOC3 | amine oxidase, copper containing 3 (vascular adhesion protein 1) | 17 | q21.31 |
| ENSG00000145087 | STXBP5L | syntaxin binding protein 5-like | 3 | q13.33 |
| ENSG00000107295 | SH3GL2 | SH3-domain GRB2-like 2 | 9 | p22.2 |
| ENSG00000126217 | MCF2L | MCF.2 cell line derived transforming sequence-like | 13 | q34 |
| ENSG00000105245 | NUMBL | numb homolog (Drosophila)-like | 19 | q13.2 |
| ENSG00000091010 | POU4F3 | POU class 4 homeobox 3 | 5 | q32 |
| ENSG00000068079 | IFI35 | interferon-induced protein 35 | 17 | q21.31 |
| ENSG00000156475 | PPP2R2B | protein phosphatase 2, regulatory subunit B, beta | 5 | q32 |
| ENSG00000115310 | RTN4 | reticulon 4 | 2 | p16.1 |
| ENSG00000012048 | BRCA1 | breast cancer 1, early onset | 17 | q21.31 |
| ENSG00000057593 | F7 | coagulation factor VII (serum prothrombin conversion accelerator) | 13 | q34 |
| ENSG00000168539 | CHRM1 | cholinergic receptor, muscarinic 1 | 11 | q12.3 |
| ENSG00000145088 | EAF2 | ELL associated factor 2 | 3 | q13.33 |
| ENSG00000176049 | JAKMIP2 | janus kinase and microtubule interacting protein 2 | 5 | q32 |
| ENSG00000163406 | SLC15A2 | solute carrier family 15 (H+/peptide transporter), member 2 | 3 | q13.33 |
| ENSG00000101311 | FERMT1 | fermitin family member 1 | 20 | p12.3 |
| ENSG00000145103 | ILDR1 | immunoglobulin-like domain containing receptor 1 | 3 | q13.33 |
| ENSG00000125845 | BMP2 | bone morphogenetic protein 2 | 20 | p12.3 |
| ENSG00000114013 | CD86 | CD86 molecule | 3 | q13.33 |
| ENSG00000133710 | SPINK5 | serine peptidase inhibitor, Kazal type 5 | 5 | q32 |
| ENSG00000155876 | RRAGA | Ras-related GTP binding A | 9 | p22.1 |
| ENSG00000166130 | IKBIP | IKBKB interacting protein | 12 | q23.1 |
| ENSG00000120868 | APAF1 | apoptotic peptidase activating factor 1 | 12 | q23.1 |
| ENSG00000133317 | LGALS12 | lectin, galactoside-binding, soluble, 12 | 11 | q12.3 |
| ENSG00000133318 | RTN3 | reticulon 3 | 11 | q13.1 |
| ENSG00000169252 | ADRB2 | adrenoceptor beta 2, surface | 5 | q32 |
| ENSG00000072518 | MARK2 | MAP/microtubule affinity-regulating kinase 2 | 11 | q13.1 |
| ENSG00000137154 | RPS6 | ribosomal protein S6 | 9 | p22.1 |
| ENSG00000105329 | TGFB1 | transforming growth factor, beta 1 | 19 | q13.2 |
| ENSG00000115392 | FANCL | Fanconi anemia, complementation group L | 2 | p16.1 |
| ENSG00000012504 | NR1H4 | nuclear receptor subfamily 1, group H, member 4 | 12 | q23.1 |
| ENSG00000119866 | BCL11A | B-cell CLL/lymphoma 11A (zinc finger protein) | 2 | p16.1 |
| ENSG00000127743 | IL17B | interleukin 17B | 5 | q32 |
| ENSG00000108861 | DUSP3 | dual specificity phosphatase 3 | 17 | q21.31 |
| ENSG00000177076 | ACER2 | alkaline ceramidase 2 | 9 | p22.1 |
| ENSG00000139842 | CUL4A | cullin 4A | 13 | q34 |
| ENSG00000161649 | CD300LG | CD300 molecule-like family member g | 17 | q21.31 |
| ENSG00000126500 | FLRT1 | fibronectin leucine rich transmembrane protein 1 | 11 | q13.1 |
| ENSG00000171843 | MLLT3 | myeloid/lymphoid or mixed-lineage leukemia (trithorax homolog, Drosophila); translocated to, 3 | 9 | p21.3 |
| ENSG00000007129 | CEACAM21 | carcinoembryonic antigen-related cell adhesion molecule 21 | 19 | q13.2 |
| ENSG00000105352 | CEACAM4 | carcinoembryonic antigen-related cell adhesion molecule 4 | 19 | q13.2 |
| ENSG00000162924 | REL | v-rel reticuloendotheliosis viral oncogene homolog (avian) | 2 | p16.1 |
| ENSG00000007306 | CEACAM7 | carcinoembryonic antigen-related cell adhesion molecule 7 | 19 | q13.2 |
| ENSG00000149781 | FERMT3 | fermitin family member 3 | 11 | q13.1 |
| ENSG00000105388 | CEACAM5 | carcinoembryonic antigen-related cell adhesion molecule 5 | 19 | q13.2 |
| ENSG00000196091 | MYBPC1 | myosin binding protein C, slow type | 12 | q23.2 |
| ENSG00000086548 | CEACAM6 | carcinoembryonic antigen-related cell adhesion molecule 6 (non-specific cross reacting antigen) | 19 | q13.2 |
| ENSG00000170956 | CEACAM3 | carcinoembryonic antigen-related cell adhesion molecule 3 | 19 | q13.2 |
| ENSG00000182578 | CSF1R | colony stimulating factor 1 receptor | 5 | q32 |
| ENSG00000173511 | VEGFB | vascular endothelial growth factor B | 11 | q13.1 |
| ENSG00000108840 | HDAC5 | histone deacetylase 5 | 17 | q21.31 |
| ENSG00000173486 | FKBP2 | FK506 binding protein 2, 13kDa | 11 | q13.1 |
| ENSG00000113721 | PDGFRB | platelet-derived growth factor receptor, beta polypeptide | 5 | q32 |
| ENSG00000111666 | CHPT1 | choline phosphotransferase 1 | 12 | q23.2 |
| ENSG00000105369 | CD79A | CD79a molecule, immunoglobulin-associated alpha | 19 | q13.2 |
| ENSG00000002330 | BAD | BCL2-associated agonist of cell death | 11 | q13.1 |
| ENSG00000136048 | DRAM1 | DNA-damage regulated autophagy modulator 1 | 12 | q23.2 |
| ENSG00000169306 | IL1RAPL1 | interleukin 1 receptor accessory protein-like 1 | X | p21.3 |
| ENSG00000171855 | IFNB1 | interferon, beta 1, fibroblast | 9 | p21.3 |
| ENSG00000177047 | IFNW1 | interferon, omega 1 | 9 | p21.3 |
| ENSG00000101349 | PAK7 | p21 protein (Cdc42/Rac)-activated kinase 7 | 20 | p12.2 |
| ENSG00000186009 | ATP4B | ATPase, H+/K+ exchanging, beta polypeptide | 13 | q34 |
| ENSG00000137080 | IFNA21 | interferon, alpha 21 | 9 | p21.3 |
| ENSG00000185974 | GRK1 | G protein-coupled receptor kinase 1 | 13 | q34 |
| ENSG00000019582 | CD74 | CD74 molecule, major histocompatibility complex, class II invariant chain | 5 | q33.1 |
| ENSG00000004939 | SLC4A1 | solute carrier family 4, anion exchanger, member 1 (erythrocyte membrane protein band 3, Diego blood group) | 17 | q21.31 |
| ENSG00000017427 | IGF1 | insulin-like growth factor 1 (somatomedin C) | 12 | q23.2 |
| ENSG00000126432 | PRDX5 | peroxiredoxin 5 | 11 | q13.1 |
| ENSG00000160145 | KALRN | kalirin, RhoGEF kinase | 3 | q21.1 |
| ENSG00000139352 | ASCL1 | achaete-scute complex homolog 1 (Drosophila) | 12 | q23.2 |
| ENSG00000030582 | GRN | granulin | 17 | q21.31 |
| ENSG00000186803 | IFNA10 | interferon, alpha 10 | 9 | p21.3 |
| ENSG00000136011 | STAB2 | stabilin 2 | 12 | q23.3 |
| ENSG00000197329 | PELI1 | pellino E3 ubiquitin protein ligase 1 | 2 | p14 |
| ENSG00000162302 | RPS6KA4 | ribosomal protein S6 kinase, 90kDa, polypeptide 4 | 11 | q13.1 |
| ENSG00000147885 | IFNA16 | interferon, alpha 16 | 9 | p21.3 |
| ENSG00000160570 | DEDD2 | death effector domain containing 2 | 19 | q13.2 |
| ENSG00000005961 | ITGA2B | integrin, alpha 2b (platelet glycoprotein IIb of IIb/IIIa complex, antigen CD41) | 17 | q21.31 |
| ENSG00000082781 | ITGB5 | integrin, beta 5 | 3 | q21.2 |
| ENSG00000132639 | SNAP25 | synaptosomal-associated protein, 25kDa | 20 | p12.2 |
| ENSG00000147873 | IFNA5 | interferon, alpha 5 | 9 | p21.3 |
| ENSG00000163848 | ZNF148 | zinc finger protein 148 | 3 | q21.2 |
| ENSG00000198642 | KLHL9 | kelch-like 9 (Drosophila) | 9 | p21.3 |
| ENSG00000079462 | PAFAH1B3 | platelet-activating factor acetylhydrolase 1b, catalytic subunit 3 (29kDa) | 19 | q13.2 |
| ENSG00000125863 | MKKS | McKusick-Kaufman syndrome | 20 | p12.2 |
| ENSG00000120235 | IFNA6 | interferon, alpha 6 | 9 | p21.3 |
| ENSG00000166598 | HSP90B1 | heat shock protein 90kDa beta (Grp94), member 1 | 12 | q23.3 |
| ENSG00000145901 | TNIP1 | TNFAIP3 interacting protein 1 | 5 | q33.1 |
| ENSG00000114520 | SNX4 | sorting nexin 4 | 3 | q21.2 |
| ENSG00000188379 | IFNA2 | interferon, alpha 2 | 9 | p21.3 |
| ENSG00000101384 | JAG1 | jagged 1 | 20 | p12.2 |
| ENSG00000073670 | ADAM11 | ADAM metallopeptidase domain 11 | 17 | q21.31 |
| ENSG00000120242 | IFNA8 | interferon, alpha 8 | 9 | p21.3 |
| ENSG00000183049 | CAMK1D | calcium/calmodulin-dependent protein kinase ID | 10 | p13 |
| ENSG00000111727 | HCFC2 | host cell factor C2 | 12 | q23.3 |
| ENSG00000197919 | IFNA1 | interferon, alpha 1 | 9 | p21.3 |
| ENSG00000110076 | NRXN2 | neurexin 2 | 11 | q13.1 |
| ENSG00000114547 | ROPN1B | rhophilin associated tail protein 1B | 3 | q21.2 |
| ENSG00000189377 | CXCL17 | chemokine (C-X-C motif) ligand 17 | 19 | q13.2 |
| ENSG00000184995 | IFNE | interferon, epsilon | 9 | p21.3 |
| ENSG00000079385 | CEACAM1 | carcinoembryonic antigen-related cell adhesion molecule 1 (biliary glycoprotein) | 19 | q13.2 |
| ENSG00000197223 | C1D | C1D nuclear receptor corepressor | 2 | p14 |
| ENSG00000171310 | CHST11 | carbohydrate (chondroitin 4) sulfotransferase 11 | 12 | q23.3 |
| ENSG00000086570 | FAT2 | FAT tumor suppressor homolog 2 (Drosophila) | 5 | q33.1 |
| ENSG00000147889 | CDKN2A | cyclin-dependent kinase inhibitor 2A | 9 | p21.3 |
| ENSG00000113140 | SPARC | secreted protein, acidic, cysteine-rich (osteonectin) | 5 | q33.1 |
| ENSG00000131094 | C1QL1 | complement component 1, q subcomponent-like 1 | 17 | q21.31 |
| ENSG00000115956 | PLEK | pleckstrin | 2 | p14 |
| ENSG00000124469 | CEACAM8 | carcinoembryonic antigen-related cell adhesion molecule 8 | 19 | q13.2 |
| ENSG00000169618 | PROKR1 | prokineticin receptor 1 | 2 | p13.3 |
| ENSG00000147883 | CDKN2B | cyclin-dependent kinase inhibitor 2B (p15, inhibits CDK4) | 9 | p21.3 |
| ENSG00000163217 | BMP10 | bone morphogenetic protein 10 | 2 | p13.3 |
| ENSG00000169604 | ANTXR1 | anthrax toxin receptor 1 | 2 | p13.3 |
| ENSG00000013503 | POLR3B | polymerase (RNA) III (DNA directed) polypeptide B | 12 | q23.3 |
| ENSG00000111783 | RFX4 | regulatory factor X, 4 (influences HLA class II expression) | 12 | q23.3 |
| ENSG00000159314 | ARHGAP27 | Rho GTPase activating protein 27 | 17 | q21.31 |
| ENSG00000113196 | HAND1 | heart and neural crest derivatives expressed 1 | 5 | q33.2 |
| ENSG00000114631 | PODXL2 | podocalyxin-like 2 | 3 | q21.3 |
| ENSG00000196975 | ANXA4 | annexin A4 | 2 | p13.3 |
| ENSG00000096872 | IFT74 | intraflagellar transport 74 homolog (Chlamydomonas) | 9 | p21.2 |
| ENSG00000204936 | CD177 | CD177 molecule | 19 | q13.31 |
| ENSG00000204933 | CD177P1 | CD177 molecule pseudogene 1 | 19 | q13.31 |
| ENSG00000120156 | TEK | TEK tyrosine kinase, endothelial | 9 | p21.2 |
| ENSG00000168067 | MAP4K2 | mitogen-activated protein kinase kinase kinase kinase 2 | 11 | q13.1 |
| ENSG00000116001 | TIA1 | TIA1 cytotoxic granule-associated RNA binding protein | 2 | p13.3 |
| ENSG00000179348 | GATA2 | GATA binding protein 2 | 3 | q21.3 |
| ENSG00000125848 | FLRT3 | fibronectin leucine rich transmembrane protein 3 | 20 | p12.1 |
| ENSG00000145850 | TIMD4 | T-cell immunoglobulin and mucin domain containing 4 | 5 | q33.3 |
| ENSG00000075035 | WSCD2 | WSC domain containing 2 | 12 | q23.3 |
| ENSG00000113249 | HAVCR1 | hepatitis A virus cellular receptor 1 | 5 | q33.3 |
| ENSG00000073050 | XRCC1 | X-ray repair complementing defective repair in Chinese hamster cells 1 | 19 | q13.31 |
| ENSG00000135077 | HAVCR2 | hepatitis A virus cellular receptor 2 | 5 | q33.3 |
| ENSG00000174600 | CMKLR1 | chemokine-like receptor 1 | 12 | q23.3 |
| ENSG00000075785 | RAB7A | RAB7A, member RAS oncogene family | 3 | q21.3 |
| ENSG00000167378 | IRGQ | immunity-related GTPase family, Q | 19 | q13.31 |
| ENSG00000075856 | SART3 | squamous cell carcinoma antigen recognized by T cells 3 | 12 | q23.3 |
| ENSG00000113263 | ITK | IL2-inducible T-cell kinase | 5 | q33.3 |
| ENSG00000133895 | MEN1 | multiple endocrine neoplasia I | 11 | q13.1 |
| ENSG00000105767 | CADM4 | cell adhesion molecule 4 | 19 | q13.31 |
| ENSG00000055163 | CYFIP2 | cytoplasmic FMR1 interacting protein 2 | 5 | q33.3 |
| ENSG00000147896 | IFNK | interferon, kappa | 9 | p21.2 |
| ENSG00000011422 | PLAUR | plasminogen activator, urokinase receptor | 19 | q13.31 |
| ENSG00000110876 | SELPLG | selectin P ligand | 12 | q24.11 |
| ENSG00000169704 | GP9 | glycoprotein IX (platelet) | 3 | q21.3 |
| ENSG00000110880 | CORO1C | coronin, actin binding protein, 1C | 12 | q24.11 |
| ENSG00000124449 | IRGC | immunity-related GTPase family, cinema | 19 | q13.31 |
| ENSG00000112679 | DUSP22 | dual specificity phosphatase 22 | 6 | p25.3 |
| ENSG00000073969 | NSF | N-ethylmaleimide-sensitive factor | 17 | q21.31 |
| ENSG00000181789 | COPG | coatomer protein complex, subunit gamma 1 | 3 | q21.3 |
| ENSG00000152672 | CLEC4F | C-type lectin domain family 4, member F | 2 | p13.3 |
| ENSG00000116031 | CD207 | CD207 molecule, langerin | 2 | p13.3 |
| ENSG00000137265 | IRF4 | interferon regulatory factor 4 | 6 | p25.3 |
| ENSG00000108379 | WNT3 | wingless-type MMTV integration site family, member 3 | 17 | q21.32 |
| ENSG00000113282 | CLINT1 | clathrin interactor 1 | 5 | q33.3 |
| ENSG00000110047 | EHD1 | EH-domain containing 1 | 11 | q13.1 |
| ENSG00000047597 | XK | X-linked Kx blood group (McLeod syndrome) | X | p21.1 |
| ENSG00000076248 | UNG | uracil-DNA glycosylase | 12 | q24.11 |
| ENSG00000164330 | EBF1 | early B-cell factor 1 | 5 | q33.3 |
| ENSG00000165168 | CYBB | cytochrome b-245, beta polypeptide | X | p21.1 |
| ENSG00000144043 | TEX261 | testis expressed 261 | 2 | p13.3 |
| ENSG00000129071 | MBD4 | methyl-CpG binding domain protein 4 | 3 | q21.3 |
| ENSG00000107201 | DDX58 | DEAD (Asp-Glu-Ala-Asp) box polypeptide 58 | 9 | p21.1 |
| ENSG00000163913 | IFT122 | intraflagellar transport 122 homolog (Chlamydomonas) | 3 | q21.3 |
| ENSG00000112685 | EXOC2 | exocyst complex component 2 | 6 | p25.3 |
| ENSG00000113302 | IL12B | interleukin 12B (natural killer cell stimulatory factor 2, cytotoxic lymphocyte maturation factor 2, p40) | 5 | q33.3 |
| ENSG00000170214 | ADRA1B | adrenoceptor alpha 1B | 5 | q33.3 |
| ENSG00000197579 | TOPORS | topoisomerase I binding, arginine/serine-rich, E3 ubiquitin protein ligase | 9 | p21.1 |
| ENSG00000164379 | FOXQ1 | forkhead box Q1 | 6 | p25.3 |
| ENSG00000054598 | FOXC1 | forkhead box C1 | 6 | p25.3 |
| ENSG00000145861 | C1QTNF2 | C1q and tumor necrosis factor related protein 2 | 5 | q33.3 |
| ENSG00000101955 | SRPX | sushi-repeat containing protein, X-linked | X | p11.4 |
| ENSG00000112699 | GMDS | GDP-mannose 4,6-dehydratase | 6 | p25.3 |
| ENSG00000073861 | TBX21 | T-box 21 | 17 | q21.32 |
| ENSG00000144036 | EXOC6B | exocyst complex component 6B | 2 | p13.2 |
| ENSG00000172752 | COL6A5 | collagen, type VI, alpha 5 | 3 | q22.1 |
| ENSG00000206384 | COL6A6 | collagen, type VI, alpha 6 | 3 | q22.1 |
| ENSG00000152457 | DCLRE1C | DNA cross-link repair 1C | 10 | p13 |
| ENSG00000017260 | ATP2C1 | ATPase, Ca++ transporting, type 2C, member 1 | 3 | q22.1 |
| ENSG00000073008 | PVR | poliovirus receptor | 19 | q13.31 |
| ENSG00000116127 | ALMS1 | Alstrom syndrome 1 | 2 | p13.1 |
| ENSG00000113328 | CCNG1 | cyclin G1 | 5 | q34 |
| ENSG00000082641 | NFE2L1 | nuclear factor (erythroid-derived 2)-like 1 | 17 | q21.32 |
| ENSG00000186567 | CEACAM19 | carcinoembryonic antigen-related cell adhesion molecule 19 | 19 | q13.31 |
| ENSG00000170584 | NUDCD2 | NudC domain containing 2 | 5 | q34 |
| ENSG00000069399 | BCL3 | B-cell CLL/lymphoma 3 | 19 | q13.32 |
| ENSG00000114670 | NEK11 | NIMA-related kinase 11 | 3 | q22.1 |
| ENSG00000162298 | SYVN1 | synovial apoptosis inhibitor 1, synoviolin | 11 | q13.1 |
| ENSG00000187244 | BCAM | basal cell adhesion molecule (Lutheran blood group) | 19 | q13.32 |
| ENSG00000170542 | SERPINB9 | serpin peptidase inhibitor, clade B (ovalbumin), member 9 | 6 | p25.2 |
| ENSG00000141293 | SKAP1 | src kinase associated phosphoprotein 1 | 17 | q21.32 |
| ENSG00000124356 | STAMBP | STAM binding protein | 2 | p13.1 |
| ENSG00000130202 | PVRL2 | poliovirus receptor-related 2 (herpesvirus entry mediator B) | 19 | q13.32 |
| ENSG00000086062 | B4GALT1 | UDP-Gal:betaGlcNAc beta 1,4- galactosyltransferase, polypeptide 1 | 9 | p21.1 |
| ENSG00000125850 | OVOL2 | ovo-like 2 (Drosophila) | 20 | p11.23 |
| ENSG00000130203 | APOE | apolipoprotein E | 19 | q13.32 |
| ENSG00000182742 | HOXB4 | homeobox B4 | 17 | q21.32 |
| ENSG00000130208 | APOC1 | apolipoprotein C-I | 19 | q13.32 |
| ENSG00000122970 | IFT81 | intraflagellar transport 81 homolog (Chlamydomonas) | 12 | q24.11 |
| ENSG00000133884 | DPF2 | D4, zinc and double PHD fingers family 2 | 11 | q13.1 |
| ENSG00000107262 | BAG1 | BCL2-associated athanogene | 9 | p13.3 |
| ENSG00000174437 | ATP2A2 | ATPase, Ca++ transporting, cardiac muscle, slow twitch 2 | 12 | q24.11 |
| ENSG00000104853 | CLPTM1 | cleft lip and palate associated transmembrane protein 1 | 19 | q13.32 |
| ENSG00000184347 | SLIT3 | slit homolog 3 (Drosophila) | 5 | q35.1 |
| ENSG00000104856 | RELB | v-rel reticuloendotheliosis viral oncogene homolog B | 19 | q13.32 |
| ENSG00000129048 | CCRL1 | chemokine (C-C motif) receptor-like 1 | 3 | q22.1 |
| ENSG00000134516 | DOCK2 | dedicator of cytokinesis 2 | 5 | q35.1 |
| ENSG00000124486 | USP9X | ubiquitin specific peptidase 9, X-linked | X | p11.4 |
| ENSG00000168056 | LTBP3 | latent transforming growth factor beta binding protein 3 | 11 | q13.1 |
| ENSG00000189114 | BLOC1S3 | biogenesis of lysosomal organelles complex-1, subunit 3 | 19 | q13.32 |
| ENSG00000132664 | POLR3F | polymerase (RNA) III (DNA directed) polypeptide F, 39 kDa | 20 | p11.23 |
| ENSG00000043462 | LCP2 | lymphocyte cytosolic protein 2 (SH2 domain containing leukocyte protein of 76kDa) | 5 | q35.1 |
| ENSG00000007047 | MARK4 | MAP/microtubule affinity-regulating kinase 4 | 19 | q13.32 |
| ENSG00000077943 | ITGA8 | integrin, alpha 8 | 10 | p13 |
| ENSG00000136436 | CALCOCO2 | calcium binding and coiled-coil domain 2 | 17 | q21.32 |
| ENSG00000114993 | RTKN | rhotekin | 2 | p13.1 |
| ENSG00000159202 | UBE2Z | ubiquitin-conjugating enzyme E2Z | 17 | q21.32 |
| ENSG00000137275 | RIPK1 | receptor (TNFRSF)-interacting serine-threonine kinase 1 | 6 | p25.2 |
| ENSG00000148481 | FAM188A | family with sequence similarity 188, member A | 10 | p13 |
| ENSG00000104884 | ERCC2 | excision repair cross-complementing rodent repair deficiency, complementation group 2 | 19 | q13.32 |
| ENSG00000174640 | SLCO2A1 | solute carrier organic anion transporter family, member 2A1 | 3 | q22.2 |
| ENSG00000159217 | IGF2BP1 | insulin-like growth factor 2 mRNA binding protein 1 | 17 | q21.32 |
| ENSG00000104881 | PPP1R13L | protein phosphatase 1, regulatory subunit 13 like | 19 | q13.32 |
| ENSG00000164438 | TLX3 | T-cell leukemia homeobox 3 | 5 | q35.1 |
| ENSG00000173327 | MAP3K11 | mitogen-activated protein kinase kinase kinase 11 | 11 | q13.1 |
| ENSG00000181163 | NPM1 | nucleophosmin (nucleolar phosphoprotein B23, numatrin) | 5 | q35.1 |
| ENSG00000167080 | B4GALNT2 | beta-1,4-N-acetyl-galactosaminyl transferase 2 | 17 | q21.32 |
| ENSG00000163785 | RYK | receptor-like tyrosine kinase | 3 | q22.2 |
| ENSG00000117877 | CD3EAP | CD3e molecule, epsilon associated protein | 19 | q13.32 |
| ENSG00000086102 | NFX1 | nuclear transcription factor, X-box binding 1 | 9 | p13.3 |
| ENSG00000012061 | ERCC1 | excision repair cross-complementing rodent repair deficiency, complementation group 1 (includes overlapping antisense sequence) | 19 | q13.32 |
| ENSG00000137267 | TUBB2A | tubulin, beta 2A class IIa | 6 | p25.2 |
| ENSG00000173039 | RELA | v-rel reticuloendotheliosis viral oncogene homolog A (avian) | 11 | q13.1 |
| ENSG00000115297 | TLX2 | T-cell leukemia homeobox 2 | 2 | p13.1 |
| ENSG00000167085 | PHB | prohibitin | 17 | q21.33 |
| ENSG00000172977 | KAT5 | K(lysine) acetyltransferase 5 | 11 | q13.1 |
| ENSG00000147044 | CASK | calcium/calmodulin-dependent serine protein kinase (MAGUK family) | X | p11.4 |
| ENSG00000165985 | C1QL3 | complement component 1, q subcomponent-like 3 | 10 | p13 |
| ENSG00000064300 | NGFR | nerve growth factor receptor | 17 | q21.33 |
| ENSG00000180822 | PSMG4 | proteasome (prosome, macropain) assembly chaperone 4 | 6 | p25.2 |
| ENSG00000111275 | ALDH2 | aldehyde dehydrogenase 2 family (mitochondrial) | 12 | q24.12 |
| ENSG00000115317 | HTRA2 | HtrA serine peptidase 2 | 2 | p13.1 |
| ENSG00000174705 | SH3PXD2B | SH3 and PX domains 2B | 5 | q35.1 |
| ENSG00000120129 | DUSP1 | dual specificity phosphatase 1 | 5 | q35.1 |
| ENSG00000172757 | CFL1 | cofilin 1 (non-muscle) | 11 | q13.1 |
| ENSG00000010310 | GIPR | gastric inhibitory polypeptide receptor | 19 | q13.32 |
| ENSG00000115325 | DOK1 | docking protein 1, 62kDa (downstream of tyrosine kinase 1) | 2 | p13.1 |
| ENSG00000135148 | TRAFD1 | TRAF-type zinc finger domain containing 1 | 12 | q24.13 |
| ENSG00000172543 | CTSW | cathepsin W | 11 | q13.1 |
| ENSG00000165272 | AQP3 | aquaporin 3 (Gill blood group) | 9 | p13.3 |
| ENSG00000132669 | RIN2 | Ras and Rab interactor 2 | 20 | p11.23 |
| ENSG00000113734 | BNIP1 | BCL2/adenovirus E1B 19kDa interacting protein 1 | 5 | q35.1 |
| ENSG00000135622 | SEMA4F | sema domain, immunoglobulin domain (Ig), transmembrane domain (TM) and short cytoplasmic domain, (semaphorin) 4F | 2 | p13.1 |
| ENSG00000005884 | ITGA3 | integrin, alpha 3 (antigen CD49C, alpha 3 subunit of VLA-3 receptor) | 17 | q21.33 |
| ENSG00000158092 | NCK1 | NCK adaptor protein 1 | 3 | q22.3 |
| ENSG00000107611 | CUBN | cubilin (intrinsic factor-cobalamin receptor) | 10 | p13 |
| ENSG00000175602 | CCDC85B | coiled-coil domain containing 85B | 11 | q13.1 |
| ENSG00000174564 | IL20RB | interleukin 20 receptor beta | 3 | q22.3 |
| ENSG00000183072 | NKX2-5 | NK2 homeobox 5 | 5 | q35.1 |
| ENSG00000175592 | FOSL1 | FOS-like antigen 1 | 11 | q13.1 |
| ENSG00000066405 | CLDN18 | claudin 18 | 3 | q22.3 |
| ENSG00000175467 | SART1 | squamous cell carcinoma antigen recognized by T cells | 11 | q13.1 |
| ENSG00000115353 | TACR1 | tachykinin receptor 1 | 2 | p12 |
| ENSG00000104941 | RSPH6A | radial spoke head 6 homolog A (Chlamydomonas) | 19 | q13.32 |
| ENSG00000125755 | SYMPK | symplekin | 19 | q13.32 |
| ENSG00000115363 | FAM176A | eva-1 homolog A (C. elegans) | 2 | p12 |
| ENSG00000108821 | COL1A1 | collagen, type I, alpha 1 | 17 | q21.33 |
| ENSG00000170604 | IRF2BP1 | interferon regulatory factor 2 binding protein 1 | 19 | q13.32 |
| ENSG00000120149 | MSX2 | msh homeobox 2 | 5 | q35.2 |
| ENSG00000205274 | TRBV20OR9-2 | T cell receptor beta variable 20/OR9-2 (non-functional) | 9 | p13.3 |
| ENSG00000184845 | DRD1 | dopamine receptor D1 | 5 | q35.2 |
| ENSG00000143954 | REG3G | regenerating islet-derived 3 gamma | 2 | p12 |
| ENSG00000069535 | MAOB | monoamine oxidase B | X | p11.3 |
| ENSG00000183938 | TRBV21OR9-2 | T cell receptor beta variable 21/OR9-2 (pseudogene) | 9 | p13.3 |
| ENSG00000113749 | HRH2 | histamine receptor H2 | 5 | q35.2 |
| ENSG00000172016 | REG3A | regenerating islet-derived 3 alpha | 2 | p12 |
| ENSG00000145920 | CPLX2 | complexin 2 | 5 | q35.2 |
| ENSG00000066032 | CTNNA2 | catenin (cadherin-associated protein), alpha 2 | 2 | p12 |
| ENSG00000158234 | FAIM | Fas apoptotic inhibitory molecule | 3 | q22.3 |
| ENSG00000167107 | ACSF2 | acyl-CoA synthetase family member 2 | 17 | q21.33 |
| ENSG00000051382 | PIK3CB | phosphatidylinositol-4,5-bisphosphate 3-kinase, catalytic subunit beta | 3 | q22.3 |
| ENSG00000183770 | FOXL2 | forkhead box L2 | 3 | q22.3 |
| ENSG00000160014 | CALM3 | calmodulin 3 (phosphorylase kinase, delta) | 19 | q13.32 |
| ENSG00000174807 | CD248 | CD248 molecule, endosialin | 11 | q13.2 |
| ENSG00000006283 | CACNA1G | calcium channel, voltage-dependent, T type, alpha 1G subunit | 17 | q21.33 |
| ENSG00000174791 | RIN1 | Ras and Rab interactor 1 | 11 | q13.2 |
| ENSG00000160013 | PTGIR | prostaglandin I2 (prostacyclin) receptor (IP) | 19 | q13.32 |
| ENSG00000152284 | TCF7L1 | transcription factor 7-like 1 (T-cell specific, HMG-box) | 2 | p11.2 |
| ENSG00000113194 | FAF2 | Fas associated factor family member 2 | 5 | q35.2 |
| ENSG00000105287 | PRKD2 | protein kinase D2 | 19 | q13.32 |
| ENSG00000089225 | TBX5 | T-box 5 | 12 | q24.21 |
| ENSG00000074276 | CDHR2 | cadherin-related family member 2 | 5 | q35.2 |
| ENSG00000158258 | CLSTN2 | calsyntenin 2 | 3 | q23 |
| ENSG00000115459 | ELMOD3 | ELMO/CED-12 domain containing 3 | 2 | p11.2 |
| ENSG00000135111 | TBX3 | T-box 3 | 12 | q24.21 |
| ENSG00000074317 | SNCB | synuclein, beta | 5 | q35.2 |
| ENSG00000042753 | AP2S1 | adaptor-related protein complex 2, sigma 1 subunit | 19 | q13.32 |
| ENSG00000125820 | NKX2-2 | NK2 homeobox 2 | 20 | p11.22 |
| ENSG00000125813 | PAX1 | paired box 1 | 20 | p11.22 |
| ENSG00000113763 | UNC5A | unc-5 homolog A (C. elegans) | 5 | q35.2 |
| ENSG00000114125 | RNF7 | ring finger protein 7 | 3 | q23 |
| ENSG00000125798 | FOXA2 | forkhead box A2 | 20 | p11.21 |
| ENSG00000108848 | LUC7L3 | LUC7-like 3 (S. cerevisiae) | 17 | q21.33 |
| ENSG00000112799 | LY86 | lymphocyte antigen 86 | 6 | p25.1 |
| ENSG00000135116 | HRK | harakiri, BCL2 interacting protein (contains only BH3 domain) | 12 | q24.22 |
| ENSG00000114126 | TFDP2 | transcription factor Dp-2 (E2F dimerization partner 2) | 3 | q23 |
| ENSG00000173714 | WFIKKN2 | WAP, follistatin/kazal, immunoglobulin, kunitz and netrin domain containing 2 | 17 | q21.33 |
| ENSG00000105327 | BBC3 | BCL2 binding component 3 | 19 | q13.32 |
| ENSG00000088992 | TESC | tescalcin | 12 | q24.22 |
| ENSG00000174080 | CTSF | cathepsin F | 11 | q13.2 |
| ENSG00000164978 | NUDT2 | nudix (nucleoside diphosphate linked moiety X)-type motif 2 | 9 | p13.3 |
| ENSG00000197405 | C5AR1 | complement component 5a receptor 1 | 19 | q13.32 |
| ENSG00000134830 | GPR77 | G protein-coupled receptor 77 | 19 | q13.32 |
| ENSG00000178726 | THBD | thrombomodulin | 20 | p11.21 |
| ENSG00000115523 | GNLY | granulysin | 2 | p11.2 |
| ENSG00000125810 | CD93 | CD93 molecule | 20 | p11.21 |
| ENSG00000175054 | ATR | ataxia telangiectasia and Rad3 related | 3 | q23 |
| ENSG00000173898 | SPTBN2 | spectrin, beta, non-erythrocytic 2 | 11 | q13.2 |
| ENSG00000176834 | VSIG10 | V-set and immunoglobulin domain containing 10 | 12 | q24.23 |
| ENSG00000024422 | EHD2 | EH-domain containing 2 | 19 | q13.33 |
| ENSG00000125823 | CSTL1 | cystatin-like 1 | 20 | p11.21 |
| ENSG00000173020 | ADRBK1 | adrenergic, beta, receptor kinase 1 | 11 | q13.2 |
| ENSG00000096696 | DSP | desmoplakin | 6 | p24.3 |
| ENSG00000111725 | PRKAB1 | protein kinase, AMP-activated, beta 1 non-catalytic subunit | 12 | q24.23 |
| ENSG00000153563 | CD8A | CD8a molecule | 2 | p11.2 |
| ENSG00000122756 | CNTFR | ciliary neurotrophic factor receptor | 9 | p13.3 |
| ENSG00000153162 | BMP6 | bone morphogenetic protein 6 | 6 | p24.3 |
| ENSG00000108924 | HLF | hepatic leukemia factor | 17 | q22 |
| ENSG00000172116 | CD8B | CD8b molecule | 2 | p11.2 |
| ENSG00000108960 | MMD | monocyte to macrophage differentiation-associated | 17 | q22 |
| ENSG00000105483 | CARD8 | caspase recruitment domain family, member 8 | 19 | q13.33 |
| ENSG00000111737 | RAB35 | RAB35, member RAS oncogene family | 12 | q24.23 |
| ENSG00000175505 | CLCF1 | cardiotrophin-like cytokine factor 1 | 11 | q13.2 |
| ENSG00000183691 | NOG | noggin | 17 | q22 |
| ENSG00000172613 | RAD9A | RAD9 homolog A (S. pombe) | 11 | q13.2 |
| ENSG00000188313 | PLSCR1 | phospholipid scramblase 1 | 3 | q24 |
| ENSG00000153933 | DGKE | diacylglycerol kinase, epsilon 64kDa | 17 | q22 |
| ENSG00000137100 | DCTN3 | dynactin 3 (p22) | 9 | p13.3 |
| ENSG00000196923 | PDLIM7 | PDZ and LIM domain 7 (enigma) | 5 | q35.3 |
| ENSG00000124802 | EEF1E1 | eukaryotic translation elongation factor 1 epsilon 1 | 6 | p24.3 |
| ENSG00000144891 | AGTR1 | angiotensin II receptor, type 1 | 3 | q24 |
| ENSG00000089159 | PXN | paxillin | 12 | q24.23 |
| ENSG00000163751 | CPA3 | carboxypeptidase A3 (mast cell) | 3 | q24 |
| ENSG00000183258 | DDX41 | DEAD (Asp-Glu-Ala-Asp) box polypeptide 41 | 5 | q35.3 |
| ENSG00000170890 | PLA2G1B | phospholipase A2, group IB (pancreas) | 12 | q24.31 |
| ENSG00000137070 | IL11RA | interleukin 11 receptor, alpha | 9 | p13.3 |
| ENSG00000172071 | EIF2AK3 | eukaryotic translation initiation factor 2-alpha kinase 3 | 2 | p11.2 |
| ENSG00000105443 | CYTH2 | cytohesin 2 | 19 | q13.33 |
| ENSG00000136451 | VEZF1 | vascular endothelial zinc finger 1 | 17 | q22 |
| ENSG00000170855 | TRIAP1 | TP53 regulated inhibitor of apoptosis 1 | 12 | q24.31 |
| ENSG00000197794 | IGKV7-3 | immunoglobulin kappa variable 7-3 (pseudogene) | 2 | p11.2 |
| ENSG00000175325 | PROP1 | PROP paired-like homeobox 1 | 5 | q35.3 |
| ENSG00000084207 | GSTP1 | glutathione S-transferase pi 1 | 11 | q13.2 |
| ENSG00000177202 | SPACA4 | sperm acrosome associated 4 | 19 | q13.33 |
| ENSG00000121053 | EPX | eosinophil peroxidase | 17 | q22 |
| ENSG00000063176 | SPHK2 | sphingosine kinase 2 | 19 | q13.33 |
| ENSG00000018408 | WWTR1 | WW domain containing transcription regulator 1 | 3 | q25.1 |
| ENSG00000078403 | MLLT10 | myeloid/lymphoid or mixed-lineage leukemia (trithorax homolog, Drosophila); translocated to, 10 | 10 | p12.31 |
| ENSG00000172005 | MAL | mal, T-cell differentiation protein | 2 | q11.1 |
| ENSG00000111846 | GCNT2 | glucosaminyl (N-acetyl) transferase 2, I-branching enzyme (I blood group) | 6 | p24.3 |
| ENSG00000005381 | MPO | myeloperoxidase | 17 | q22 |
| ENSG00000172724 | CCL19 | chemokine (C-C motif) ligand 19 | 9 | p13.3 |
| ENSG00000157837 | SPPL3 | signal peptide peptidase like 3 | 12 | q24.31 |
| ENSG00000182264 | IZUMO1 | izumo sperm-egg fusion 1 | 19 | q13.33 |
| ENSG00000137077 | CCL21 | chemokine (C-C motif) ligand 21 | 9 | p13.3 |
| ENSG00000174951 | FUT1 | fucosyltransferase 1 (galactoside 2-alpha-L-fucosyltransferase, H blood group) | 19 | q13.33 |
| ENSG00000115041 | KCNIP3 | Kv channel interacting protein 3, calsenilin | 2 | q11.1 |
| ENSG00000110719 | TCIRG1 | T-cell, immune regulator 1, ATPase, H+ transporting, lysosomal V0 subunit A3 | 11 | q13.2 |
| ENSG00000089041 | P2RX7 | purinergic receptor P2X, ligand-gated ion channel, 7 | 12 | q24.31 |
| ENSG00000108387 | SEPT4 | septin 4 | 17 | q22 |
| ENSG00000181788 | SIAH2 | siah E3 ubiquitin protein ligase 2 | 3 | q25.1 |
| ENSG00000087074 | PPP1R15A | protein phosphatase 1, regulatory subunit 15A | 19 | q13.33 |
| ENSG00000135124 | P2RX4 | purinergic receptor P2X, ligand-gated ion channel, 4 | 12 | q24.31 |
| ENSG00000162337 | LRP5 | low density lipoprotein receptor-related protein 5 | 11 | q13.2 |
| ENSG00000176783 | RUFY1 | RUN and FYVE domain containing 1 | 5 | q35.3 |
| ENSG00000165280 | VCP | valosin containing protein | 9 | p13.3 |
| ENSG00000124827 | GCM2 | glial cells missing homolog 2 (Drosophila) | 6 | p24.2 |
| ENSG00000087088 | BAX | BCL2-associated X protein | 19 | q13.33 |
| ENSG00000069482 | GAL | galanin/GMAP prepropeptide | 11 | q13.2 |
| ENSG00000111859 | NEDD9 | neural precursor cell expressed, developmentally down-regulated 9 | 6 | p24.2 |
| ENSG00000169313 | P2RY12 | purinergic receptor P2Y, G-protein coupled, 12 | 3 | q25.1 |
| ENSG00000152580 | IGSF10 | immunoglobulin superfamily, member 10 | 3 | q25.1 |
| ENSG00000132740 | IGHMBP2 | immunoglobulin mu binding protein 2 | 11 | q13.3 |
| ENSG00000170633 | RNF34 | ring finger protein 34, E3 ubiquitin protein ligase | 12 | q24.31 |
| ENSG00000095951 | HIVEP1 | human immunodeficiency virus type I enhancer binding protein 1 | 6 | p24.1 |
| ENSG00000104827 | CGB | chorionic gonadotropin, beta polypeptide | 19 | q13.33 |
| ENSG00000141367 | CLTC | clathrin, heavy chain (Hc) | 17 | q23.1 |
| ENSG00000126759 | CFP | complement factor properdin | X | p11.23 |
| ENSG00000141378 | PTRH2 | peptidyl-tRNA hydrolase 2 | 17 | q23.1 |
| ENSG00000161011 | SQSTM1 | sequestosome 1 | 5 | q35.3 |
| ENSG00000078401 | EDN1 | endothelin 1 | 6 | p24.1 |
| ENSG00000172927 | MYEOV | myeloma overexpressed (in a subset of t(11;14) positive multiple myelomas) | 11 | q13.3 |
| ENSG00000110092 | CCND1 | cyclin D1 | 11 | q13.3 |
| ENSG00000108443 | RPS6KB1 | ribosomal protein S6 kinase, 70kDa, polypeptide 1 | 17 | q23.1 |
| ENSG00000162344 | FGF19 | fibroblast growth factor 19 | 11 | q13.3 |
| ENSG00000075388 | FGF4 | fibroblast growth factor 4 | 11 | q13.3 |
| ENSG00000186895 | FGF3 | fibroblast growth factor 3 | 11 | q13.3 |
| ENSG00000110801 | PSMD9 | proteasome (prosome, macropain) 26S subunit, non-ATPase, 9 | 12 | q24.31 |
| ENSG00000113269 | RNF130 | ring finger protein 130 | 5 | q35.3 |
| ENSG00000110987 | BCL7A | B-cell CLL/lymphoma 7A | 12 | q24.31 |
| ENSG00000168758 | SEMA4C | sema domain, immunoglobulin domain (Ig), transmembrane domain (TM) and short cytoplasmic domain, (semaphorin) 4C | 2 | q11.2 |
| ENSG00000168040 | FADD | Fas (TNFRSF6)-associated via death domain | 11 | q13.3 |
| ENSG00000104863 | LIN7B | lin-7 homolog B (C. elegans) | 19 | q13.33 |
| ENSG00000050748 | MAPK9 | mitogen-activated protein kinase 9 | 5 | q35.3 |
| ENSG00000204671 | IL31 | interleukin 31 | 12 | q24.31 |
| ENSG00000184047 | DIABLO | diablo, IAP-binding mitochondrial protein | 12 | q24.31 |
| ENSG00000161055 | SCGB3A1 | secretoglobin, family 3A, member 1 | 5 | q35.3 |
| ENSG00000139719 | VPS33A | vacuolar protein sorting 33 homolog A (S. cerevisiae) | 12 | q24.31 |
| ENSG00000198722 | UNC13B | unc-13 homolog B (C. elegans) | 9 | p13.3 |
| ENSG00000130529 | TRPM4 | transient receptor potential cation channel, subfamily M, member 4 | 19 | q13.33 |
| ENSG00000104894 | CD37 | CD37 molecule | 19 | q13.33 |
| ENSG00000115085 | ZAP70 | zeta-chain (TCR) associated protein kinase 70kDa | 2 | q11.2 |
| ENSG00000131068 | DEFB118 | defensin, beta 118 | 20 | q11.21 |
| ENSG00000137101 | CD72 | CD72 molecule | 9 | p13.3 |
| ENSG00000180483 | DEFB119 | defensin, beta 119 | 20 | q11.21 |
| ENSG00000182782 | HCAR2 | hydroxycarboxylic acid receptor 2 | 12 | q24.31 |
| ENSG00000163659 | TIPARP | TCDD-inducible poly(ADP-ribose) polymerase | 3 | q25.31 |
| ENSG00000112149 | CD83 | CD83 molecule | 6 | p23 |
| ENSG00000137078 | SIT1 | signaling threshold regulating transmembrane adaptor 1 | 9 | p13.3 |
| ENSG00000204548 | DEFB121 | defensin, beta 121 | 20 | q11.21 |
| ENSG00000090554 | FLT3LG | fms-related tyrosine kinase 3 ligand | 19 | q13.33 |
| ENSG00000204547 | DEFB122 | defensin, beta 122 (pseudogene) | 20 | q11.21 |
| ENSG00000008083 | JARID2 | jumonji, AT rich interactive domain 2 | 6 | p22.3 |
| ENSG00000180424 | DEFB123 | defensin, beta 123 | 20 | q11.21 |
| ENSG00000184276 | DEFB108B | defensin, beta 108B | 11 | q13.4 |
| ENSG00000180383 | DEFB124 | defensin, beta 124 | 20 | q11.21 |
| ENSG00000130787 | HIP1R | huntingtin interacting protein 1 related | 12 | q24.31 |
| ENSG00000163661 | PTX3 | pentraxin 3, long | 3 | q25.32 |
| ENSG00000101294 | HM13 | histocompatibility (minor) 13 | 20 | q11.21 |
| ENSG00000137496 | IL18BP | interleukin 18 binding protein | 11 | q13.4 |
| ENSG00000104870 | FCGRT | Fc fragment of IgG, receptor, transporter, alpha | 19 | q13.33 |
| ENSG00000011028 | MRC2 | mannose receptor, C type 2 | 17 | q23.2 |
| ENSG00000150967 | ABCB9 | ATP-binding cassette, sub-family B (MDR/TAP), member 9 | 12 | q24.31 |
| ENSG00000178053 | MLF1 | myeloid leukemia factor 1 | 3 | q25.32 |
| ENSG00000099256 | PRTFDC1 | phosphoribosyl transferase domain containing 1 | 10 | p12.1 |
| ENSG00000126456 | IRF3 | interferon regulatory factor 3 | 19 | q13.33 |
| ENSG00000110195 | FOLR1 | folate receptor 1 (adult) | 11 | q13.4 |
| ENSG00000159640 | ACE | angiotensin I converting enzyme (peptidyl-dipeptidase A) 1 | 17 | q23.3 |
| ENSG00000126453 | BCL2L12 | BCL2-like 12 (proline rich) | 19 | q13.33 |
| ENSG00000165458 | INPPL1 | inositol polyphosphate phosphatase-like 1 | 11 | q13.4 |
| ENSG00000168811 | IL12A | interleukin 12A (natural killer cell stimulatory factor 1, cytotoxic lymphocyte maturation factor 1, p35) | 3 | q25.33 |
| ENSG00000137076 | TLN1 | talin 1 | 9 | p13.3 |
| ENSG00000198909 | MAP3K3 | mitogen-activated protein kinase kinase kinase 3 | 17 | q23.3 |
| ENSG00000068885 | IFT80 | intraflagellar transport 80 homolog (Chlamydomonas) | 3 | q25.33 |
| ENSG00000196961 | AP2A1 | adaptor-related protein complex 2, alpha 1 subunit | 19 | q13.33 |
| ENSG00000125968 | ID1 | inhibitor of DNA binding 1, dominant negative helix-loop-helix protein | 20 | q11.21 |
| ENSG00000115526 | CHST10 | carbohydrate sulfotransferase 10 | 2 | q11.2 |
| ENSG00000115539 | PDCL3 | phosducin-like 3 | 2 | q11.2 |
| ENSG00000198231 | DDX42 | DEAD (Asp-Glu-Ala-Asp) box polypeptide 42 | 17 | q23.3 |
| ENSG00000169255 | B3GALNT1 | beta-1,3-N-acetylgalactosaminyltransferase 1 (globoside blood group) | 3 | q26.1 |
| ENSG00000171552 | BCL2L1 | BCL2-like 1 | 20 | q11.21 |
| ENSG00000137478 | FCHSD2 | FCH and double SH3 domains 2 | 11 | q13.4 |
| ENSG00000175591 | P2RY2 | purinergic receptor P2Y, G-protein coupled, 2 | 11 | q13.4 |
| ENSG00000015285 | WAS | Wiskott-Aldrich syndrome | X | p11.23 |
| ENSG00000087191 | PSMC5 | proteasome (prosome, macropain) 26S subunit, ATPase, 5 | 17 | q23.3 |
| ENSG00000110237 | ARHGEF17 | Rho guanine nucleotide exchange factor (GEF) 17 | 11 | q13.4 |
| ENSG00000104951 | IL4I1 | interleukin 4 induced 1 | 19 | q13.33 |
| ENSG00000054967 | RELT | RELT tumor necrosis factor receptor | 11 | q13.4 |
| ENSG00000108604 | SMARCD2 | SWI/SNF related, matrix associated, actin dependent regulator of chromatin, subfamily d, member 2 | 17 | q23.3 |
| ENSG00000107175 | CREB3 | cAMP responsive element binding protein 3 | 9 | p13.3 |
| ENSG00000088325 | TPX2 | TPX2, microtubule-associated, homolog (Xenopus laevis) | 20 | q11.21 |
| ENSG00000071054 | MAP4K4 | mitogen-activated protein kinase kinase kinase kinase 4 | 2 | q11.2 |
| ENSG00000169136 | ATF5 | activating transcription factor 5 | 19 | q13.33 |
| ENSG00000161640 | SIGLEC11 | sialic acid binding Ig-like lectin 11 | 19 | q13.33 |
| ENSG00000114204 | SERPINI2 | serpin peptidase inhibitor, clade I (pancpin), member 2 | 3 | q26.1 |
| ENSG00000094631 | HDAC6 | histone deacetylase 6 | X | p11.23 |
| ENSG00000073060 | SCARB1 | scavenger receptor class B, member 1 | 12 | q24.31 |
| ENSG00000161643 | SIGLEC16 | sialic acid binding Ig-like lectin 16 (gene/pseudogene) | 19 | q13.33 |
| ENSG00000101306 | MYLK2 | myosin light chain kinase 2 | 20 | q11.21 |
| ENSG00000114209 | PDCD10 | programmed cell death 10 | 3 | q26.1 |
| ENSG00000175575 | PAAF1 | proteasomal ATPase-associated factor 1 | 11 | q13.4 |
| ENSG00000163536 | SERPINI1 | serpin peptidase inhibitor, clade I (neuroserpin), member 1 | 3 | q26.1 |
| ENSG00000187726 | DNAJB13 | DnaJ (Hsp40) homolog, subfamily B, member 13 | 11 | q13.4 |
| ENSG00000137393 | RNF144B | ring finger protein 144B | 6 | p22.3 |
| ENSG00000115590 | IL1R2 | interleukin 1 receptor, type II | 2 | q11.2 |
| ENSG00000085276 | MECOM | MDS1 and EVI1 complex locus | 3 | q26.2 |
| ENSG00000115594 | IL1R1 | interleukin 1 receptor, type I | 2 | q11.2 |
| ENSG00000179772 | FOXS1 | forkhead box S1 | 20 | q11.21 |
| ENSG00000115598 | IL1RL2 | interleukin 1 receptor-like 2 | 2 | q12.1 |
| ENSG00000115602 | IL1RL1 | interleukin 1 receptor-like 1 | 2 | q12.1 |
| ENSG00000115604 | IL18R1 | interleukin 18 receptor 1 | 2 | q12.1 |
| ENSG00000115607 | IL18RAP | interleukin 18 receptor accessory protein | 2 | q12.1 |
| ENSG00000081760 | AACS | acetoacetyl-CoA synthetase | 12 | q24.31 |
| ENSG00000131408 | NR1H2 | nuclear receptor subfamily 1, group H, member 2 | 19 | q13.33 |
| ENSG00000124766 | SOX4 | SRY (sex determining region Y)-box 4 | 6 | p22.3 |
| ENSG00000007312 | CD79B | CD79b molecule, immunoglobulin-associated beta | 17 | q23.3 |
| ENSG00000198914 | POU3F3 | POU class 3 homeobox 3 | 2 | q12.1 |
| ENSG00000137133 | HINT2 | histidine triad nucleotide binding protein 2 | 9 | p13.3 |
| ENSG00000163558 | PRKCI | protein kinase C, iota | 3 | q26.2 |
| ENSG00000137103 | TMEM8B | transmembrane protein 8B | 9 | p13.3 |
| ENSG00000101336 | HCK | hemopoietic cell kinase | 20 | q11.21 |
| ENSG00000135966 | TGFBRAP1 | transforming growth factor, beta receptor associated protein 1 | 2 | q12.2 |
| ENSG00000142539 | SPIB | Spi-B transcription factor (Spi-1/PU.1 related), isoform CRA_a; Transcription factor Spi-B; cDNA FLJ57438, highly similar to Transcription factor Spi-B | 19 | q13.33 |
| ENSG00000136603 | SKIL | SKI-like oncogene | 3 | q26.2 |
| ENSG00000086967 | MYBPC2 | myosin binding protein C, fast type | 19 | q13.33 |
| ENSG00000108622 | ICAM2 | intercellular adhesion molecule 2 | 17 | q23.3 |
| ENSG00000178607 | ERN1 | endoplasmic reticulum to nucleus signaling 1 | 17 | q23.3 |
| ENSG00000013297 | CLDN11 | claudin 11 | 3 | q26.2 |
| ENSG00000071051 | NCK2 | NCK adaptor protein 2 | 2 | q12.2 |
| ENSG00000137486 | ARRB1 | arrestin, beta 1 | 11 | q13.4 |
| ENSG00000149273 | RPS3 | ribosomal protein S3 | 11 | q13.4 |
| ENSG00000111450 | STX2 | syntaxin 2 | 12 | q24.33 |
| ENSG00000154310 | TNIK | TRAF2 and NCK interacting kinase | 3 | q26.31 |
| ENSG00000146038 | DCDC2 | doublecortin domain containing 2 | 6 | p22.3 |
| ENSG00000102096 | PIM2 | pim-2 oncogene | X | p11.23 |
| ENSG00000126003 | PLAGL2 | pleiomorphic adenoma gene-like 2 | 20 | q11.21 |
| ENSG00000075651 | PLD1 | phospholipase D1, phosphatidylcholine-specific | 3 | q26.31 |
| ENSG00000108854 | SMURF2 | SMAD specific E3 ubiquitin protein ligase 2 | 17 | q24.1 |
| ENSG00000122705 | CLTA | clathrin, light chain A | 9 | p13.3 |
| ENSG00000120063 | GNA13 | guanine nucleotide binding protein (G protein), alpha 13 | 17 | q24.1 |
| ENSG00000099246 | RAB18 | RAB18, member RAS oncogene family | 10 | p12.1 |
| ENSG00000159921 | GNE | glucosamine (UDP-N-acetyl)-2-epimerase/N-acetylmannosamine kinase | 9 | p13.3 |
| ENSG00000169756 | LIMS1 | LIM and senescent cell antigen-like domains 1 | 2 | q12.3 |
| ENSG00000085741 | WNT11 | wingless-type MMTV integration site family, member 11 | 11 | q13.5 |
| ENSG00000075420 | FNDC3B | fibronectin type III domain containing 3B | 3 | q26.31 |
| ENSG00000137492 | PRKRIR | protein-kinase, interferon-inducible double stranded RNA dependent inhibitor, repressor of (P58 repressor) | 11 | q13.5 |
| ENSG00000121853 | GHSR | growth hormone secretagogue receptor | 3 | q26.31 |
| ENSG00000121858 | TNFSF10 | tumor necrosis factor (ligand) superfamily, member 10 | 3 | q26.31 |
| ENSG00000091583 | APOH | apolipoprotein H (beta-2-glycoprotein I) | 17 | q24.2 |
| ENSG00000135960 | EDAR | ectodysplasin A receptor | 2 | q13 |
| ENSG00000154229 | PRKCA | protein kinase C, alpha | 17 | q24.2 |
| ENSG00000114346 | ECT2 | epithelial cell transforming sequence 2 oncogene | 3 | q26.31 |
| ENSG00000149600 | COMMD7 | COMM domain containing 7 | 20 | q11.21 |
| ENSG00000197170 | PSMD12 | proteasome (prosome, macropain) 26S subunit, non-ATPase, 12 | 17 | q24.2 |
| ENSG00000186148 | LOC440895 | LIM and senescent cell antigen-like domains 3-like (LOC440895), transcript variant 1, non-coding RNA | 2 | q13 |
| ENSG00000144063 | MALL | mal, T-cell differentiation protein-like | 2 | q13 |
| ENSG00000144061 | NPHP1 | nephronophthisis 1 (juvenile) | 2 | q13 |
| ENSG00000184115 | LOC100288695 | LIM and senescent cell antigen-like-containing domain protein 3 (Particularly interesting new Cys-His protein 3)(PINCH-3) | 2 | q13 |
| ENSG00000149269 | PAK1 | p21 protein (Cdc42/Rac)-activated kinase 1 | 11 | q14.1 |
| ENSG00000102007 | PLP2 | proteolipid protein 2 (colonic epithelium-enriched) | X | p11.23 |
| ENSG00000129450 | SIGLEC9 | sialic acid binding Ig-like lectin 9 | 19 | q13.41 |
| ENSG00000169679 | BUB1 | budding uninhibited by benzimidazoles 1 homolog (yeast) | 2 | q13 |
| ENSG00000168995 | SIGLEC7 | sialic acid binding Ig-like lectin 7 | 19 | q13.41 |
| ENSG00000105383 | CD33 | CD33 molecule | 19 | q13.41 |
| ENSG00000186806 | VSIG10L | V-set and immunoglobulin domain containing 10 like | 19 | q13.41 |
| ENSG00000153094 | BCL2L11 | BCL2-like 11 (apoptosis facilitator) | 2 | q13 |
| ENSG00000169760 | NLGN1 | neuroligin 1 | 3 | q26.31 |
| ENSG00000105374 | NKG7 | natural killer cell group 7 sequence | 19 | q13.41 |
| ENSG00000142512 | SIGLEC10 | sialic acid binding Ig-like lectin 10 | 19 | q13.41 |
| ENSG00000108946 | PRKAR1A | protein kinase, cAMP-dependent, regulatory, type I, alpha | 17 | q24.2 |
| ENSG00000167104 | BPIFB6 | BPI fold containing family B, member 6 | 20 | q11.21 |
| ENSG00000105366 | SIGLEC8 | sialic acid binding Ig-like lectin 8 | 19 | q13.41 |
| ENSG00000033327 | GAB2 | GRB2-associated binding protein 2 | 11 | q14.1 |
| ENSG00000172667 | ZMAT3 | zinc finger, matrin-type 3 | 3 | q26.32 |
| ENSG00000121879 | PIK3CA | phosphatidylinositol-4,5-bisphosphate 3-kinase, catalytic subunit alpha | 3 | q26.32 |
| ENSG00000095739 | BAMBI | BMP and activin membrane-bound inhibitor homolog (Xenopus laevis) | 10 | p12.1 |
| ENSG00000153208 | MERTK | c-mer proto-oncogene tyrosine kinase | 2 | q13 |
| ENSG00000105492 | SIGLEC6 | sialic acid binding Ig-like lectin 6 | 19 | q13.41 |
| ENSG00000107338 | SHB | Src homology 2 domain containing adaptor protein B | 9 | p13.1 |
| ENSG00000144152 | FBLN7 | fibulin 7 | 2 | q13 |
| ENSG00000105501 | SIGLEC5 | sialic acid binding Ig-like lectin 5 | 19 | q13.41 |
| ENSG00000144161 | ZC3H8 | zinc finger CCCH-type containing 8 | 2 | q14.1 |
| ENSG00000165490 | C11orf82 | chromosome 11 open reading frame 82 | 11 | q14.1 |
| ENSG00000049768 | FOXP3 | forkhead box P3 | X | p11.23 |
| ENSG00000105509 | HAS1 | hyaluronan synthase 1 | 19 | q13.41 |
| ENSG00000171051 | FPR1 | formyl peptide receptor 1 | 19 | q13.41 |
| ENSG00000171049 | FPR2 | formyl peptide receptor 2 | 19 | q13.41 |
| ENSG00000187474 | FPR3 | formyl peptide receptor 3 | 19 | q13.41 |
| ENSG00000106714 | CNTNAP3 | contactin associated protein-like 3 | 9 | p12 |
| ENSG00000144136 | SLC20A1 | solute carrier family 20 (phosphate transporter), member 1 | 2 | q14.1 |
| ENSG00000154265 | ABCA5 | ATP-binding cassette, sub-family A (ABC1), member 5 | 17 | q24.3 |
| ENSG00000115008 | IL1A | interleukin 1, alpha | 2 | q14.1 |
| ENSG00000137501 | SYTL2 | synaptotagmin-like 2 | 11 | q14.1 |
| ENSG00000108984 | MAP2K6 | mitogen-activated protein kinase kinase 6 | 17 | q24.3 |
| ENSG00000125538 | IL1B | interleukin 1, beta | 2 | q14.1 |
| ENSG00000163098 | BIRC8 | baculoviral IAP repeat containing 8 | 19 | q13.42 |
| ENSG00000125571 | IL37 | interleukin 37 | 2 | q14.1 |
| ENSG00000068985 | PAGE1 | P antigen family, member 1 (prostate associated) | X | p11.23 |
| ENSG00000114416 | FXR1 | fragile X mental retardation, autosomal homolog 1 | 3 | q26.33 |
| ENSG00000125398 | SOX9 | SRY (sex determining region Y)-box 9 | 17 | q24.3 |
| ENSG00000136688 | IL36G | interleukin 36, gamma | 2 | q14.1 |
| ENSG00000136694 | IL36A | interleukin 36, alpha | 2 | q14.1 |
| ENSG00000181449 | SOX2 | SRY (sex determining region Y)-box 2 | 3 | q26.33 |
| ENSG00000105568 | PPP2R1A | protein phosphatase 2, regulatory subunit A, alpha | 19 | q13.41 |
| ENSG00000073921 | PICALM | phosphatidylinositol binding clathrin assembly protein | 11 | q14.2 |
| ENSG00000136696 | IL36B | interleukin 36, beta | 2 | q14.1 |
| ENSG00000171365 | CLCN5 | chloride channel, voltage-sensitive 5 | X | p11.23 |
| ENSG00000136695 | IL36RN | interleukin 36 receptor antagonist | 2 | q14.1 |
| ENSG00000136697 | IL1F10 | interleukin 1 family, member 10 (theta) | 2 | q14.1 |
| ENSG00000136689 | IL1RN | interleukin 1 receptor antagonist | 2 | q14.1 |
| ENSG00000069188 | SDK2 | sidekick cell adhesion molecule 2 | 17 | q25.1 |
| ENSG00000148516 | ZEB1 | zinc finger E-box binding homeobox 1 | 10 | p11.22 |
| ENSG00000176597 | B3GNT5 | UDP-GlcNAc:betaGal beta-1,3-N-acetylglucosaminyltransferase 5 | 3 | q27.1 |
| ENSG00000172578 | KLHL6 | kelch-like 6 (Drosophila) | 3 | q27.1 |
| ENSG00000147081 | AKAP4 | A kinase (PRKA) anchor protein 4 | X | p11.22 |
| ENSG00000174804 | FZD4 | frizzled family receptor 4 | 11 | q14.2 |
| ENSG00000109861 | CTSC | cathepsin C | 11 | q14.2 |
| ENSG00000114770 | ABCC5 | ATP-binding cassette, sub-family C (CFTR/MRP), member 5 | 3 | q27.1 |
| ENSG00000170122 | FOXD4 | forkhead box D4 | 9 | p24.3 |
| ENSG00000101412 | E2F1 | E2F transcription factor 1 | 20 | q11.22 |
| ENSG00000154529 | CNTNAP3B | contactin associated protein-like 3B | 9 | p11.2 |
| ENSG00000167851 | CD300A | CD300a molecule | 17 | q25.1 |
| ENSG00000178789 | CD300LB | CD300 molecule-like family member b | 17 | q25.1 |
| ENSG00000130385 | BMP15 | bone morphogenetic protein 15 | X | p11.22 |
| ENSG00000167850 | CD300C | CD300c molecule | 17 | q25.1 |
| ENSG00000204345 | CD300LD | CD300 molecule-like family member d | 17 | q25.1 |
| ENSG00000186407 | CD300E | CD300e molecule | 17 | q25.1 |
| ENSG00000010704 | HFE | hemochromatosis | 6 | p22.2 |
| ENSG00000186074 | CD300LF | CD300 molecule-like family member f | 17 | q25.1 |
| ENSG00000179222 | MAGED1 | melanoma antigen family D, 1 | X | p11.22 |
| ENSG00000180152 | BIRC8 | baculoviral IAP repeat-containing 8 | 2 | q14.1 |
| ENSG00000142405 | NLRP12 | NLR family, pyrin domain containing 12 | 19 | q13.42 |
| ENSG00000175166 | PSMD2 | proteasome (prosome, macropain) 26S subunit, non-ATPase, 2 | 3 | q27.1 |
| ENSG00000165323 | FAT3 | FAT tumor suppressor homolog 3 (Drosophila) | 11 | q14.3 |
| ENSG00000184523 | PTGER4P2 | prostaglandin E receptor 4 (subtype EP4) pseudogene 2 | 9 | q13 |
| ENSG00000179820 | MYADM | myeloid-associated differentiation marker | 19 | q13.42 |
| ENSG00000126583 | PRKCG | protein kinase C, gamma | 19 | q13.42 |
| ENSG00000019169 | MARCO | macrophage receptor with collagenous structure | 2 | q14.2 |
| ENSG00000144119 | C1QL2 | complement component 1, q subcomponent-like 2 | 2 | q14.2 |
| ENSG00000115107 | STEAP3 | STEAP family member 3, metalloreductase | 2 | q14.2 |
| ENSG00000170909 | OSCAR | osteoclast associated, immunoglobulin-like receptor | 19 | q13.42 |
| ENSG00000105619 | TFPT | TCF3 (E2A) fusion partner (in childhood Leukemia) | 19 | q13.42 |
| ENSG00000125450 | NUP85 | nucleoporin 85kDa | 17 | q25.1 |
| ENSG00000078747 | ITCH | itchy E3 ubiquitin protein ligase | 20 | q11.22 |
| ENSG00000110218 | PANX1 | pannexin 1 | 11 | q21 |
| ENSG00000115109 | EPB41L5 | erythrocyte membrane protein band 4.1 like 5 | 2 | q14.2 |
| ENSG00000090534 | THPO | thrombopoietin | 3 | q27.1 |
| ENSG00000105617 | LENG1 | leukocyte receptor cluster (LRC) member 1 | 19 | q13.42 |
| ENSG00000144118 | RALB | v-ral simian leukemia viral oncogene homolog B (ras related; GTP binding protein) | 2 | q14.2 |
| ENSG00000090539 | CHRD | chordin | 3 | q27.1 |
| ENSG00000163083 | INHBB | inhibin, beta B | 2 | q14.2 |
| ENSG00000074047 | GLI2 | GLI family zinc finger 2 | 2 | q14.2 |
| ENSG00000196371 | FUT4 | fucosyltransferase 4 (alpha (1,3) fucosyltransferase, myeloid-specific) | 11 | q21 |
| ENSG00000150093 | ITGB1 | integrin, beta 1 (fibronectin receptor, beta polypeptide, antigen CD29 includes MDF2, MSK12) | 10 | p11.22 |
| ENSG00000186152 | LILRP1 | leukocyte immunoglobulin-like receptor pseudogene 1 | 19 | q13.42 |
| ENSG00000134627 | PIWIL4 | piwi-like 4 (Drosophila) | 11 | q21 |
| ENSG00000204779 | FOXD4L5 | forkhead box D4-like 5 | 9 | q21.11 |
| ENSG00000204577 | LILRB3 | leukocyte immunoglobulin-like receptor, subfamily B (with TM and ITIM domains), member 3 | 19 | q13.42 |
| ENSG00000166025 | AMOTL1 | angiomotin like 1 | 11 | q21 |
| ENSG00000105609 | LILRB5 | leukocyte immunoglobulin-like receptor, subfamily B (with TM and ITIM domains), member 5 | 19 | q13.42 |
| ENSG00000184659 | FOXD4L4 | forkhead box D4-like 4 | 9 | q21.11 |
| ENSG00000131042 | LILRB2 | leukocyte immunoglobulin-like receptor, subfamily B (with TM and ITIM domains), member 2 | 19 | q13.42 |
| ENSG00000187116 | LILRA5 | leukocyte immunoglobulin-like receptor, subfamily A (with TM domain), member 5 | 19 | q13.42 |
| ENSG00000073350 | LLGL2 | lethal giant larvae homolog 2 (Drosophila) | 17 | q25.1 |
| ENSG00000099250 | NRP1 | neuropilin 1 | 10 | p11.22 |
| ENSG00000170858 | LILRA4 | leukocyte immunoglobulin-like receptor, subfamily A (with TM domain), member 4 | 19 | q13.42 |
| ENSG00000167613 | LAIR1 | leukocyte-associated immunoglobulin-like receptor 1 | 19 | q13.42 |
| ENSG00000073792 | IGF2BP2 | insulin-like growth factor 2 mRNA binding protein 2 | 3 | q27.2 |
| ENSG00000155052 | CNTNAP5 | contactin associated protein-like 5 | 2 | q14.3 |
| ENSG00000167614 | TTYH1 | tweety homolog 1 (Drosophila) | 19 | q13.42 |
| ENSG00000136732 | GYPC | glycophorin C (Gerbich blood group) | 2 | q14.3 |
| ENSG00000167615 | LENG8 | leukocyte receptor cluster (LRC) member 8 | 19 | q13.42 |
| ENSG00000136717 | BIN1 | bridging integrator 1 | 2 | q14.3 |
| ENSG00000198646 | NCOA6 | nuclear receptor coactivator 6 | 20 | q11.22 |
| ENSG00000161526 | SAP30BP | SAP30 binding protein | 17 | q25.1 |
| ENSG00000149972 | CNTN5 | contactin 5 | 11 | q22.1 |
| ENSG00000167618 | LAIR2 | leukocyte-associated immunoglobulin-like receptor 2 | 19 | q13.42 |
| ENSG00000132470 | ITGB4 | integrin, beta 4 | 17 | q25.1 |
| ENSG00000104970 | KIR3DX1 | killer cell immunoglobulin-like receptor, three domains, X1 | 19 | q13.42 |
| ENSG00000058866 | DGKG | diacylglycerol kinase, gamma 90kDa | 3 | q27.3 |
| ENSG00000104974 | LILRA1 | leukocyte immunoglobulin-like receptor, subfamily A (with TM domain), member 1 | 19 | q13.42 |
| ENSG00000104972 | LILRB1 | leukocyte immunoglobulin-like receptor, subfamily B (with TM and ITIM domains), member 1 | 19 | q13.42 |
| ENSG00000186818 | LILRB4 | leukocyte immunoglobulin-like receptor, subfamily B (with TM and ITIM domains), member 4 | 19 | q13.42 |
| ENSG00000187559 | FOXD4L3 | forkhead box D4-like 3 | 9 | q21.11 |
| ENSG00000145192 | AHSG | alpha-2-HS-glycoprotein | 3 | q27.3 |
| ENSG00000125498 | KIR2DL1 | killer cell immunoglobulin-like receptor, two domains, long cytoplasmic tail, 1 | 19 | q13.42 |
| ENSG00000154330 | PGM5 | phosphoglucomutase 5 | 9 | q21.11 |
| ENSG00000163161 | ERCC3 | excision repair cross-complementing rodent repair deficiency, complementation group 3 | 2 | q14.3 |
| ENSG00000092929 | UNC13D | unc-13 homolog D (C. elegans) | 17 | q25.1 |
| ENSG00000148498 | PARD3 | par-3 partitioning defective 3 homolog (C. elegans) | 10 | p11.21 |
| ENSG00000113905 | HRG | histidine-rich glycoprotein | 3 | q27.3 |
| ENSG00000023445 | BIRC3 | baculoviral IAP repeat containing 3 | 11 | q22.2 |
| ENSG00000113889 | KNG1 | kininogen 1 | 3 | q27.3 |
| ENSG00000110330 | BIRC2 | baculoviral IAP repeat containing 2 | 11 | q22.2 |
| ENSG00000115718 | PROC | protein C (inactivator of coagulation factors Va and VIIIa) | 2 | q14.3 |
| ENSG00000188878 | FBF1 | Fas (TNFRSF6) binding factor 1 | 17 | q25.1 |
| ENSG00000100983 | GSS | glutathione synthetase | 20 | q11.22 |
| ENSG00000161533 | ACOX1 | acyl-CoA oxidase 1, palmitoyl | 17 | q25.1 |
| ENSG00000181092 | ADIPOQ | adiponectin, C1Q and collagen domain containing | 3 | q27.3 |
| ENSG00000072163 | LIMS2 | LIM and senescent cell antigen-like domains 2 | 2 | q14.3 |
| ENSG00000165059 | PRKACG | protein kinase, cAMP-dependent, catalytic, gamma | 9 | q21.11 |
| ENSG00000165060 | FXN | frataxin | 9 | q21.11 |
| ENSG00000144230 | GPR17 | G protein-coupled receptor 17 | 2 | q14.3 |
| ENSG00000149968 | MMP3 | matrix metallopeptidase 3 (stromelysin 1, progelatinase) | 11 | q22.2 |
| ENSG00000127241 | MASP1 | mannan-binding lectin serine peptidase 1 (C4/C2 activating component of Ra-reactive factor) | 3 | q27.3 |
| ENSG00000108094 | CUL2 | cullin 2 | 10 | p11.21 |
| ENSG00000157005 | SST | somatostatin | 3 | q27.3 |
| ENSG00000113916 | BCL6 | B-cell CLL/lymphoma 6 | 3 | q27.3 |
| ENSG00000189013 | KIR2DL4 | killer cell immunoglobulin-like receptor, two domains, long cytoplasmic tail, 4 | 19 | q13.42 |
| ENSG00000182473 | EXOC7 | exocyst complex component 7 | 17 | q25.1 |
| ENSG00000145012 | LPP | LIM domain containing preferred translocation partner in lipoma | 3 | q27.3 |
| ENSG00000101000 | PROCR | protein C receptor, endothelial | 20 | q11.22 |
| ENSG00000073282 | TP63 | tumor protein p63 | 3 | q28 |
| ENSG00000170962 | PDGFD | platelet derived growth factor D | 11 | q22.3 |
| ENSG00000129654 | FOXJ1 | forkhead box J1 | 17 | q25.1 |
| ENSG00000204403 | CASP12 | caspase 12 (gene/pseudogene) | 11 | q22.3 |
| ENSG00000196954 | CASP4 | caspase 4, apoptosis-related cysteine peptidase | 11 | q22.3 |
| ENSG00000167633 | KIR3DL1 | killer cell immunoglobulin-like receptor, three domains, long cytoplasmic tail, 1 | 19 | q13.42 |
| ENSG00000107282 | APBA1 | amyloid beta (A4) precursor protein-binding, family A, member 1 | 9 | q21.12 |
| ENSG00000137757 | CASP5 | caspase 5, apoptosis-related cysteine peptidase | 11 | q22.3 |
| ENSG00000137752 | CASP1 | caspase 1, apoptosis-related cysteine peptidase | 11 | q22.3 |
| ENSG00000163347 | CLDN1 | claudin 1 | 3 | q28 |
| ENSG00000176170 | SPHK1 | sphingosine kinase 1 | 17 | q25.1 |
| ENSG00000113946 | CLDN16 | claudin 16 | 3 | q28 |
| ENSG00000186431 | FCAR | Fc fragment of IgA, receptor for | 19 | q13.42 |
| ENSG00000165072 | MAMDC2 | MAM domain containing 2 | 9 | q21.12 |
| ENSG00000196083 | IL1RAP | interleukin 1 receptor accessory protein | 3 | q28 |
| ENSG00000204397 | CARD16 | caspase recruitment domain family, member 16 | 11 | q22.3 |
| ENSG00000129673 | AANAT | aralkylamine N-acetyltransferase | 17 | q25.1 |
| ENSG00000189430 | NCR1 | natural cytotoxicity triggering receptor 1 | 19 | q13.42 |
| ENSG00000022556 | NLRP2 | NLR family, pyrin domain containing 2 | 19 | q13.42 |
| ENSG00000088053 | GP6 | glycoprotein VI (platelet) | 19 | q13.42 |
| ENSG00000136002 | ARHGEF4 | Rho guanine nucleotide exchange factor (GEF) 4 | 2 | q21.1 |
| ENSG00000070495 | JMJD6 | jumonji domain containing 6 | 17 | q25.1 |
| ENSG00000110675 | ELMOD1 | ELMO/CED-12 domain containing 1 | 11 | q22.3 |
| ENSG00000125965 | GDF5 | growth differentiation factor 5 | 20 | q11.22 |
| ENSG00000198836 | OPA1 | optic atrophy 1 (autosomal dominant) | 3 | q29 |
| ENSG00000166266 | CUL5 | cullin 5 | 11 | q22.3 |
| ENSG00000112812 | PRSS16 | protease, serine, 16 (thymus) | 6 | p22.1 |
| ENSG00000114315 | HES1 | hairy and enhancer of split 1, (Drosophila) | 3 | q29 |
| ENSG00000149311 | ATM | ataxia telangiectasia mutated | 11 | q22.3 |
| ENSG00000178732 | GP5 | glycoprotein V (platelet) | 3 | q29 |
| ENSG00000080031 | PTPRH | protein tyrosine phosphatase, receptor type, H | 19 | q13.42 |
| ENSG00000089685 | BIRC5 | baculoviral IAP repeat containing 5 | 17 | q25.3 |
| ENSG00000095752 | IL11 | interleukin 11 | 19 | q13.42 |
| ENSG00000184557 | SOCS3 | suppressor of cytokine signaling 3 | 17 | q25.3 |
| ENSG00000145113 | MUC4 | mucin 4, cell surface associated | 3 | q29 |
| ENSG00000107372 | ZFAND5 | zinc finger, AN1-type domain 5 | 9 | q21.13 |
| ENSG00000137707 | BTG4 | B-cell translocation gene 4 | 11 | q23.1 |
| ENSG00000108669 | CYTH1 | cytohesin 1 | 17 | q25.3 |
| ENSG00000170145 | SIK2 | salt-inducible kinase 2 | 11 | q23.1 |
| ENSG00000035862 | TIMP2 | TIMP metallopeptidase inhibitor 2 | 17 | q25.3 |
| ENSG00000108679 | LGALS3BP | lectin, galactoside-binding, soluble, 3 binding protein | 17 | q25.3 |
| ENSG00000072274 | TFRC | transferrin receptor (p90, CD71) | 3 | q29 |
| ENSG00000121966 | CXCR4 | chemokine (C-X-C motif) receptor 4 | 2 | q22.1 |
| ENSG00000196632 | WNK3 | WNK lysine deficient protein kinase 3 | X | p11.22 |
| ENSG00000135046 | ANXA1 | annexin A1 | 9 | q21.13 |
| ENSG00000165731 | RET | ret proto-oncogene | 10 | q11.21 |
| ENSG00000173918 | C1QTNF1 | C1q and tumor necrosis factor related protein 1 | 17 | q25.3 |
| ENSG00000109846 | CRYAB | crystallin, alpha B | 11 | q23.1 |
| ENSG00000063245 | EPN1 | epsin 1 | 19 | q13.42 |
| ENSG00000150540 | HNMT | histamine N-methyltransferase | 2 | q22.1 |
| ENSG00000168702 | LRP1B | low density lipoprotein receptor-related protein 1B | 2 | q22.2 |
| ENSG00000102302 | FGD1 | FYVE, RhoGEF and PH domain containing 1 | X | p11.22 |
| ENSG00000141582 | CBX4 | chromobox homolog 4 | 17 | q25.3 |
| ENSG00000180370 | PAK2 | p21 protein (Cdc42/Rac)-activated kinase 2 | 3 | q29 |
| ENSG00000150782 | IL18 | interleukin 18 (interferon-gamma-inducing factor) | 11 | q23.1 |
| ENSG00000141527 | CARD14 | caspase recruitment domain family, member 14 | 17 | q25.3 |
| ENSG00000075711 | DLG1 | discs, large homolog 1 (Drosophila) | 3 | q29 |
| ENSG00000115919 | KYNU | kynureninase | 2 | q22.2 |
| ENSG00000149294 | NCAM1 | neural cell adhesion molecule 1 | 11 | q23.2 |
| ENSG00000107562 | CXCL12 | chemokine (C-X-C motif) ligand 12 | 10 | q11.21 |
| ENSG00000149295 | DRD2 | dopamine receptor D2 | 11 | q23.2 |
| ENSG00000169554 | ZEB2 | zinc finger E-box binding homeobox 2 | 2 | q22.3 |
| ENSG00000198300 | PEG3 | paternally expressed 3 | 19 | q13.43 |
| ENSG00000145016 | KIAA0226 | KIAA0226 | 3 | q29 |
| ENSG00000067445 | TRO | trophinin | X | p11.21 |
| ENSG00000099139 | PCSK5 | proprotein convertase subtilisin/kexin type 5 | 9 | q21.13 |
| ENSG00000048028 | USP28 | ubiquitin specific peptidase 28 | 11 | q23.2 |
| ENSG00000105146 | AURKC | aurora kinase C | 19 | q13.43 |
| ENSG00000158571 | PFKFB1 | 6-phosphofructo-2-kinase/fructose-2,6-biphosphatase 1 | X | p11.21 |
| ENSG00000185621 | LMLN | leishmanolysin-like (metallopeptidase M8 family) | 3 | q29 |
| ENSG00000181409 | AATK | apoptosis-associated tyrosine kinase | 17 | q25.3 |
| ENSG00000109906 | ZBTB16 | zinc finger and BTB domain containing 16 | 11 | q23.2 |
| ENSG00000115963 | RND3 | Rho family GTPase 3 | 2 | q23.3 |
| ENSG00000106772 | PRUNE2 | prune homolog 2 (Drosophila) | 9 | q21.2 |
| ENSG00000123610 | TNFAIP6 | tumor necrosis factor, alpha-induced protein 6 | 2 | q23.3 |
| ENSG00000182985 | CADM1 | cell adhesion molecule 1 | 11 | q23.3 |
| ENSG00000186765 | FSCN2 | fascin homolog 2, actin-bundling protein, retinal (Strongylocentrotus purpuratus) | 17 | q25.3 |
| ENSG00000182389 | CACNB4 | calcium channel, voltage-dependent, beta 4 subunit | 2 | q23.3 |
| ENSG00000187601 | MAGEH1 | melanoma antigen family H, 1 | X | p11.21 |
| ENSG00000110243 | APOA5 | apolipoprotein A-V | 11 | q23.3 |
| ENSG00000110244 | APOA4 | apolipoprotein A-IV | 11 | q23.3 |
| ENSG00000110245 | APOC3 | apolipoprotein C-III | 11 | q23.3 |
| ENSG00000118137 | APOA1 | apolipoprotein A-I | 11 | q23.3 |
| ENSG00000141522 | ARHGDIA | Rho GDP dissociation inhibitor (GDI) alpha | 17 | q25.3 |
| ENSG00000186787 | SPIN2B | spindlin family, member 2B | X | p11.21 |
| ENSG00000115165 | CYTIP | cytohesin 1 interacting protein | 2 | q24.1 |
| ENSG00000123612 | ACVR1C | activin A receptor, type IC | 2 | q24.1 |
| ENSG00000185105 | MYADML2 | myeloid-associated differentiation marker-like 2 | 17 | q25.3 |
| ENSG00000115170 | ACVR1 | activin A receptor, type I | 2 | q24.1 |
| ENSG00000101082 | SLA2 | Src-like-adaptor 2 | 20 | q11.23 |
| ENSG00000168092 | PAFAH1B2 | platelet-activating factor acetylhydrolase 1b, catalytic subunit 2 (30kDa) | 11 | q23.3 |
| ENSG00000131089 | ARHGEF9 | Cdc42 guanine nucleotide exchange factor (GEF) 9 | X | q11.2 |
| ENSG00000144283 | PKP4 | plakophilin 4 | 2 | q24.1 |
| ENSG00000163331 | DAPL1 | death associated protein-like 1 | 2 | q24.1 |
| ENSG00000177103 | DSCAML1 | Down syndrome cell adhesion molecule like 1 | 11 | q23.3 |
| ENSG00000054219 | LY75 | lymphocyte antigen 75 | 2 | q24.2 |
| ENSG00000153246 | PLA2R1 | phospholipase A2 receptor 1, 180kDa | 2 | q24.2 |
| ENSG00000115221 | ITGB6 | integrin, beta 6 | 2 | q24.2 |
| ENSG00000110324 | IL10RA | interleukin 10 receptor, alpha | 11 | q23.3 |
| ENSG00000147065 | MSN | moesin | X | q12 |
| ENSG00000173762 | CD7 | CD7 molecule | 17 | q25.3 |
| ENSG00000141574 | SECTM1 | secreted and transmembrane 1 | 17 | q25.3 |
| ENSG00000107643 | MAPK8 | mitogen-activated protein kinase 8 | 10 | q11.22 |
| ENSG00000204655 | MOG | myelin oligodendrocyte glycoprotein | 6 | p22.1 |
| ENSG00000160593 | AMICA1 | adhesion molecule, interacts with CXADR antigen 1 | 11 | q23.3 |
| ENSG00000155659 | VSIG4 | V-set and immunoglobulin domain containing 4 | X | q12 |
| ENSG00000099326 | MZF1 | myeloid zinc finger 1 | 19 | q13.43 |
| ENSG00000160588 | MPZL3 | myelin protein zero-like 3 | 11 | q23.3 |
| ENSG00000115233 | PSMD14 | proteasome (prosome, macropain) 26S subunit, non-ATPase, 14 | 2 | q24.2 |
| ENSG00000149573 | MPZL2 | myelin protein zero-like 2 | 11 | q23.3 |
| ENSG00000198851 | CD3E | CD3e molecule, epsilon (CD3-TCR complex) | 11 | q23.3 |
| ENSG00000135018 | UBQLN1 | ubiquilin 1 | 9 | q21.32 |
| ENSG00000167286 | CD3D | CD3d molecule, delta (CD3-TCR complex) | 11 | q23.3 |
| ENSG00000160654 | CD3G | CD3g molecule, gamma (CD3-TCR complex) | 11 | q23.3 |
| ENSG00000197635 | DPP4 | dipeptidyl-peptidase 4 | 2 | q24.2 |
| ENSG00000131080 | EDA2R | ectodysplasin A2 receptor | X | q12 |
| ENSG00000204644 | ZFP57 | zinc finger protein 57 homolog (mouse) | 6 | p22.1 |
| ENSG00000118058 | MLL | myeloid/lymphoid or mixed-lineage leukemia (trithorax homolog, Drosophila) | 11 | q23.3 |
| ENSG00000115267 | IFIH1 | interferon induced with helicase C domain 1 | 2 | q24.2 |
| ENSG00000204642 | HLA-F | major histocompatibility complex, class I, F | 6 | p22.1 |
| ENSG00000079482 | OPHN1 | oligophrenin 1 | X | q12 |
| ENSG00000175711 | B3GNTL1 | UDP-GlcNAc:betaGal beta-1,3-N-acetylglucosaminyltransferase-like 1 | 17 | q25.3 |
| ENSG00000181126 | HLA-V | major histocompatibility complex, class I, V (pseudogene) | 6 | p22.1 |
| ENSG00000118096 | IFT46 | intraflagellar transport 46 homolog (Chlamydomonas) | 11 | q23.3 |
| ENSG00000136531 | SCN2A | sodium channel, voltage-gated, type II, alpha subunit | 2 | q24.3 |
| ENSG00000160683 | CXCR5 | chemokine (C-X-C motif) receptor 5 | 11 | q23.3 |
| ENSG00000090776 | EFNB1 | ephrin-B1 | X | q13.1 |
| ENSG00000178662 | CSRNP3 | cysteine-serine-rich nuclear protein 3 | 2 | q24.3 |
| ENSG00000186174 | BCL9L | B-cell CLL/lymphoma 9-like | 11 | q23.3 |
| ENSG00000197122 | SRC | v-src sarcoma (Schmidt-Ruppin A-2) viral oncogene homolog (avian) | 20 | q11.23 |
| ENSG00000158813 | EDA | ectodysplasin A | X | q13.1 |
| ENSG00000089289 | IGBP1 | immunoglobulin (CD79A) binding protein 1 | X | q13.1 |
| ENSG00000172375 | C2CD2L | C2CD2-like | 11 | q23.3 |
| ENSG00000166619 | BLCAP | bladder cancer associated protein | 20 | q11.23 |
| ENSG00000120500 | ARR3 | arrestin 3, retinal (X-arrestin) | X | q13.1 |
| ENSG00000163072 | NOSTRIN | nitric oxide synthase trafficker | 2 | q24.3 |
| ENSG00000188611 | ASAH2 | N-acylsphingosine amidohydrolase (non-lysosomal ceramidase) 2 | 10 | q11.23 |
| ENSG00000132792 | CTNNBL1 | catenin, beta like 1 | 20 | q11.23 |
| ENSG00000198964 | SGMS1 | sphingomyelin synthase 1 | 10 | q11.23 |
| ENSG00000110395 | CBL | Cbl proto-oncogene, E3 ubiquitin protein ligase | 11 | q23.3 |
| ENSG00000081479 | LRP2 | low density lipoprotein receptor-related protein 2 | 2 | q31.1 |
| ENSG00000076706 | MCAM | melanoma cell adhesion molecule | 11 | q23.3 |
| ENSG00000148584 | A1CF | APOBEC1 complementation factor | 10 | q11.23 |
| ENSG00000138399 | FASTKD1 | FAST kinase domains 1 | 2 | q31.1 |
| ENSG00000154096 | THY1 | Thy-1 cell surface antigen | 11 | q23.3 |
| ENSG00000138398 | PPIG | peptidylprolyl isomerase G (cyclophilin G) | 2 | q31.1 |
| ENSG00000110400 | PVRL1 | poliovirus receptor-related 1 (herpesvirus entry mediator C) | 11 | q23.3 |
| ENSG00000198959 | TGM2 | transglutaminase 2 (C polypeptide, protein-glutamine-gamma-glutamyltransferase) | 20 | q11.23 |
| ENSG00000196914 | ARHGEF12 | Rho guanine nucleotide exchange factor (GEF) 12 | 11 | q23.3 |
| ENSG00000101425 | BPI | bactericidal/permeability-increasing protein | 20 | q11.23 |
| ENSG00000165471 | MBL2 | mannose-binding lectin (protein C) 2, soluble | 10 | q21.1 |
| ENSG00000129988 | LBP | lipopolysaccharide binding protein | 20 | q11.23 |
| ENSG00000128683 | GAD1 | glutamate decarboxylase 1 (brain, 67kDa) | 2 | q31.1 |
| ENSG00000137642 | SORL1 | sortilin-related receptor, L(DLR class) A repeats containing | 11 | q24.1 |
| ENSG00000150275 | PCDH15 | protocadherin-related 15 | 10 | q21.1 |
| ENSG00000109943 | CRTAM | cytotoxic and regulatory T cell molecule | 11 | q24.1 |
| ENSG00000182035 | ADIG | adipogenin | 20 | q11.23 |
| ENSG00000180447 | GAS1 | growth arrest-specific 1 | 9 | q21.33 |
| ENSG00000147168 | IL2RG | interleukin 2 receptor, gamma | X | q13.1 |
| ENSG00000196730 | DAPK1 | death-associated protein kinase 1 | 9 | q21.33 |
| ENSG00000135047 | CTSL1 | cathepsin L1 | 9 | q21.33 |
| ENSG00000188029 | CTSL3 | cathepsin L family member 3 | 9 | q21.33 |
| ENSG00000144355 | DLX1 | distal-less homeobox 1 | 2 | q31.1 |
| ENSG00000204437 | CTSL1P6 | cathepsin L1 pseudogene 6 | 10 | q22.3 |
| ENSG00000091409 | ITGA6 | integrin, alpha 6 | 2 | q31.1 |
| ENSG00000198900 | TOP1 | topoisomerase (DNA) I | 20 | q12 |
| ENSG00000154144 | TBRG1 | transforming growth factor beta regulator 1 | 11 | q24.2 |
| ENSG00000091428 | RAPGEF4 | Rap guanine nucleotide exchange factor (GEF) 4 | 2 | q31.1 |
| ENSG00000091436 | ZAK | Mitogen-activated protein kinase kinase kinase MLT | 2 | q31.1 |
| ENSG00000124181 | PLCG1 | phospholipase C, gamma 1 | 20 | q12 |
| ENSG00000154146 | NRGN | neurogranin (protein kinase C substrate, RC3) | 11 | q24.2 |
| ENSG00000019102 | VSIG2 | V-set and immunoglobulin domain containing 2 | 11 | q24.2 |
| ENSG00000196338 | NLGN3 | neuroligin 3 | X | q13.1 |
| ENSG00000149564 | ESAM | endothelial cell adhesion molecule | 11 | q24.2 |
| ENSG00000172845 | SP3 | Sp3 transcription factor | 2 | q31.1 |
| ENSG00000170312 | CDK1 | cyclin-dependent kinase 1 | 10 | q21.2 |
| ENSG00000165478 | HEPACAM | hepatic and glial cell adhesion molecule | 11 | q24.2 |
| ENSG00000187764 | SEMA4D | sema domain, immunoglobulin domain (Ig), transmembrane domain (TM) and short cytoplasmic domain, (semaphorin) 4D | 9 | q22.2 |
| ENSG00000149557 | FEZ1 | fasciculation and elongation protein zeta 1 (zygin I) | 11 | q24.2 |
| ENSG00000204625 | HCG9 | HLA complex group 9 (non-protein coding) | 6 | p22.1 |
| ENSG00000130222 | GADD45G | growth arrest and DNA-damage-inducible, gamma | 9 | q22.2 |
| ENSG00000204622 | HLA-J | major histocompatibility complex, class I, J (pseudogene) | 6 | p22.1 |
| ENSG00000149547 | EI24 | etoposide induced 2.4 mRNA | 11 | q24.2 |
| ENSG00000150347 | ARID5B | AT rich interactive domain 5B (MRF1-like) | 10 | q21.2 |
| ENSG00000196090 | PTPRT | protein tyrosine phosphatase, receptor type, T | 20 | q13.11 |
| ENSG00000165025 | SYK | spleen tyrosine kinase | 9 | q22.2 |
| ENSG00000064309 | CDON | Cdon homolog (mouse) | 11 | q24.2 |
| ENSG00000183230 | CTNNA3 | catenin (cadherin-associated protein), alpha 3 | 10 | q21.3 |
| ENSG00000165030 | NFIL3 | nuclear factor, interleukin 3 regulated | 9 | q22.31 |
| ENSG00000150455 | TIRAP | toll-interleukin 1 receptor (TIR) domain containing adaptor protein | 11 | q24.2 |
| ENSG00000185513 | L3MBTL1 | l(3)mbt-like 1 (Drosophila) | 20 | q13.12 |
| ENSG00000096717 | SIRT1 | sirtuin 1 | 10 | q21.3 |
| ENSG00000116044 | NFE2L2 | nuclear factor (erythroid-derived 2)-like 2 | 2 | q31.2 |
| ENSG00000134954 | ETS1 | v-ets erythroblastosis virus E26 oncogene homolog 1 (avian) | 11 | q24.3 |
| ENSG00000204599 | TRIM39 | tripartite motif containing 39 | 6 | p22.1 |
| ENSG00000101049 | SGK2 | serum/glucocorticoid regulated kinase 2 | 20 | q13.12 |
| ENSG00000151702 | FLI1 | Friend leukemia virus integration 1 | 11 | q24.3 |
| ENSG00000120471 | TP53AIP1 | tumor protein p53 regulated apoptosis inducing protein 1 | 11 | q24.3 |
| ENSG00000147166 | ITGB1BP2 | integrin beta 1 binding protein (melusin) 2 | X | q13.1 |
| ENSG00000180228 | PRKRA | protein kinase, interferon-inducible double stranded RNA dependent activator | 2 | q31.2 |
| ENSG00000079150 | FKBP7 | FK506 binding protein 7 | 2 | q31.2 |
| ENSG00000204590 | GNL1 | guanine nucleotide binding protein-like 1 | 6 | p21.33 |
| ENSG00000101052 | IFT52 | intraflagellar transport 52 homolog (Chlamydomonas) | 20 | q13.12 |
| ENSG00000204580 | DDR1 | discoidin domain receptor tyrosine kinase 1 | 6 | p21.33 |
| ENSG00000060339 | CCAR1 | cell division cycle and apoptosis regulator 1 | 10 | q21.3 |
| ENSG00000127083 | OMD | osteomodulin | 9 | q22.31 |
| ENSG00000106819 | ASPN | asporin | 9 | q22.31 |
| ENSG00000106823 | ECM2 | extracellular matrix protein 2, female organ and adipocyte specific | 9 | q22.31 |
| ENSG00000134917 | ADAMTS8 | ADAM metallopeptidase with thrombospondin type 1 motif, 8 | 11 | q24.3 |
| ENSG00000185963 | BICD2 | bicaudal D homolog 2 (Drosophila) | 9 | q22.31 |
| ENSG00000182667 | NTM | neurotrimin | 11 | q25 |
| ENSG00000186810 | CXCR3 | chemokine (C-X-C motif) receptor 3 | X | q13.1 |
| ENSG00000183715 | OPCML | opioid binding protein/cell adhesion molecule-like | 11 | q25 |
| ENSG00000127084 | FGD3 | FYVE, RhoGEF and PH domain containing 3 | 9 | q22.31 |
| ENSG00000080854 | IGSF9B | immunoglobulin superfamily, member 9B | 11 | q25 |
| ENSG00000122862 | SRGN | serglycin | 10 | q22.1 |
| ENSG00000131669 | NINJ1 | ninjurin 1 | 9 | q22.31 |
| ENSG00000151500 | THYN1 | thymocyte nuclear protein 1 | 11 | q25 |
| ENSG00000186871 | ERCC6L | excision repair cross-complementing rodent repair deficiency, complementation group 6-like | X | q13.1 |
| ENSG00000165238 | WNK2 | WNK lysine deficient protein kinase 2 | 9 | q22.31 |
| ENSG00000125931 | CITED1 | Cbp/p300-interacting transactivator, with Glu/Asp-rich carboxy-terminal domain, 1 | X | q13.1 |
| ENSG00000132824 | SERINC3 | serine incorporator 3 | 20 | q13.12 |
| ENSG00000115232 | ITGA4 | integrin, alpha 4 (antigen CD49D, alpha 4 subunit of VLA-4 receptor) | 2 | q31.3 |
| ENSG00000137337 | MDC1 | mediator of DNA-damage checkpoint 1 | 6 | p21.33 |
| ENSG00000188452 | CERKL | ceramide kinase-like | 2 | q31.3 |
| ENSG00000196230 | TUBB | tubulin, beta class I | 6 | p21.33 |
| ENSG00000162992 | NEUROD1 | neuronal differentiation 1 | 2 | q31.3 |
| ENSG00000131668 | BARX1 | BARX homeobox 1 | 9 | q22.32 |
| ENSG00000196839 | ADA | adenosine deaminase | 20 | q13.12 |
| ENSG00000137331 | IER3 | immediate early response 3 | 6 | p21.33 |
| ENSG00000064205 | WISP2 | WNT1 inducible signaling pathway protein 2 | 20 | q13.12 |
| ENSG00000162998 | FRZB | frizzled-related protein | 2 | q32.1 |
| ENSG00000148110 | HIATL1 | hippocampus abundant transcript-like 1 | 9 | q22.32 |
| ENSG00000061676 | NCKAP1 | NCK-associated protein 1 | 2 | q32.1 |
| ENSG00000101098 | RIMS4 | regulating synaptic membrane exocytosis 4 | 20 | q13.12 |
| ENSG00000065548 | ZC3H15 | zinc finger CCCH-type containing 15 | 2 | q32.1 |
| ENSG00000158169 | FANCC | Fanconi anemia, complementation group C | 9 | q22.32 |
| ENSG00000138448 | ITGAV | integrin, alpha V | 2 | q32.1 |
| ENSG00000197467 | COL13A1 | collagen, type XIII, alpha 1 | 10 | q22.1 |
| ENSG00000204539 | CDSN | corneodesmosin | 6 | p21.33 |
| ENSG00000163012 | ZSWIM2 | zinc finger, SWIM-type containing 2 | 2 | q32.1 |
| ENSG00000144366 | GULP1 | GULP, engulfment adaptor PTB domain containing 1 | 2 | q32.1 |
| ENSG00000101109 | STK4 | serine/threonine kinase 4 | 20 | q13.12 |
| ENSG00000168542 | COL3A1 | collagen, type III, alpha 1 | 2 | q32.2 |
| ENSG00000042286 | AIFM2 | apoptosis-inducing factor, mitochondrion-associated, 2 | 10 | q22.1 |
| ENSG00000204525 | HLA-C | major histocompatibility complex, class I, C | 6 | p21.33 |
| ENSG00000124107 | SLPI | secretory leukocyte peptidase inhibitor | 20 | q13.12 |
| ENSG00000156574 | NODAL | nodal homolog (mouse) | 10 | q22.1 |
| ENSG00000138379 | MSTN | myostatin | 2 | q32.2 |
| ENSG00000180644 | PRF1 | perforin 1 (pore forming protein) | 10 | q22.1 |
| ENSG00000124232 | RBPJL | recombination signal binding protein for immunoglobulin kappa J region-like | 20 | q13.12 |
| ENSG00000124145 | SDC4 | syndecan 4 | 20 | q13.12 |
| ENSG00000166224 | SGPL1 | sphingosine-1-phosphate lyase 1 | 10 | q22.1 |
| ENSG00000107731 | UNC5B | unc-5 homolog B (C. elegans) | 10 | q22.1 |
| ENSG00000204520 | MICA | MHC class I polypeptide-related sequence A | 6 | p21.33 |
| ENSG00000136943 | CTSL2 | cathepsin L2 | 9 | q22.33 |
| ENSG00000115415 | STAT1 | signal transducer and activator of transcription 1, 91kDa | 2 | q32.2 |
| ENSG00000107736 | CDH23 | cadherin-related 23 | 10 | q22.1 |
| ENSG00000138378 | STAT4 | signal transducer and activator of transcription 4 | 2 | q32.3 |
| ENSG00000124155 | PIGT | phosphatidylinositol glycan anchor biosynthesis, class T | 20 | q13.12 |
| ENSG00000168497 | SDPR | serum deprivation response | 2 | q32.3 |
| ENSG00000204516 | MICB | MHC class I polypeptide-related sequence B | 6 | p21.33 |
| ENSG00000198563 | DDX39B | DEAD (Asp-Glu-Ala-Asp) box polypeptide 39B | 6 | p21.33 |
| ENSG00000081320 | STK17B | serine/threonine kinase 17b | 2 | q32.3 |
| ENSG00000204498 | NFKBIL1 | nuclear factor of kappa light polypeptide gene enhancer in B-cells inhibitor-like 1 | 6 | p21.33 |
| ENSG00000168209 | DDIT4 | DNA-damage-inducible transcript 4 | 10 | q22.1 |
| ENSG00000107745 | MICU1 | mitochondrial calcium uptake 1 | 10 | q22.1 |
| ENSG00000144381 | HSPD1 | heat shock 60kDa protein 1 (chaperonin) | 2 | q33.1 |
| ENSG00000204482 | LST1 | leukocyte specific transcript 1 | 6 | p21.33 |
| ENSG00000187325 | TAF9B | TAF9B RNA polymerase II, TATA box binding protein (TBP)-associated factor, 31kDa | X | q21.1 |
| ENSG00000136936 | XPA | xeroderma pigmentosum, complementation group A | 9 | q22.33 |
| ENSG00000115541 | HSPE1 | heat shock 10kDa protein 1 (chaperonin 10) | 2 | q33.1 |
| ENSG00000173198 | CYSLTR1 | cysteinyl leukotriene receptor 1 | X | q21.1 |
| ENSG00000204475 | NCR3 | natural cytotoxicity triggering receptor 3 | 6 | p21.33 |
| ENSG00000095380 | NANS | N-acetylneuraminic acid synthase | 9 | q22.33 |
| ENSG00000204472 | AIF1 | allograft inflammatory factor 1 | 6 | p21.33 |
| ENSG00000101470 | TNNC2 | troponin C type 2 (fast) | 20 | q13.12 |
| ENSG00000115934 | PPIL3 | peptidylprolyl isomerase (cyclophilin)-like 3 | 12 | p12.1 |
| ENSG00000003402 | CFLAR | CASP8 and FADD-like apoptosis regulator | 2 | q33.1 |
| ENSG00000064601 | CTSA | cathepsin A | 20 | q13.12 |
| ENSG00000204291 | COL15A1 | collagen, type XV, alpha 1 | 9 | q22.33 |
| ENSG00000072133 | RPS6KA6 | ribosomal protein S6 kinase, 90kDa, polypeptide 6 | X | q21.1 |
| ENSG00000003400 | CASP10 | caspase 10, apoptosis-related cysteine peptidase | 2 | q33.1 |
| ENSG00000106799 | TGFBR1 | transforming growth factor, beta receptor 1 | 9 | q22.33 |
| ENSG00000204438 | GPANK1 | G patch domain and ankyrin repeats 1 | 6 | p21.33 |
| ENSG00000184788 | SATL1 | spermidine/spermine N1-acetyl transferase-like 1 | X | q21.1 |
| ENSG00000064012 | CASP8 | caspase 8, apoptosis-related cysteine peptidase | 2 | q33.1 |
| ENSG00000204435 | CSNK2B | casein kinase 2, beta polypeptide | 6 | p21.33 |
| ENSG00000204428 | LY6G5C | lymphocyte antigen 6 complex, locus G5C | 6 | p21.33 |
| ENSG00000204427 | ABHD16A | abhydrolase domain containing 16A | 6 | p21.33 |
| ENSG00000148660 | CAMK2G | calcium/calmodulin-dependent protein kinase II gamma | 10 | q22.2 |
| ENSG00000100985 | MMP9 | matrix metallopeptidase 9 (gelatinase B, 92kDa gelatinase, 92kDa type IV collagenase) | 20 | q13.12 |
| ENSG00000204424 | LY6G6F | lymphocyte antigen 6 complex, locus G6F | 6 | p21.33 |
| ENSG00000204422 | LY6G6E | lymphocyte antigen 6 complex, locus G6E (pseudogene) | 6 | p21.33 |
| ENSG00000204421 | LY6G6C | lymphocyte antigen 6 complex, locus G6C | 6 | p21.33 |
| ENSG00000102290 | PCDH11X | protocadherin 11 X-linked | X | q21.31 |
| ENSG00000204217 | BMPR2 | bone morphogenetic protein receptor, type II (serine/threonine kinase) | 2 | q33.1 |
| ENSG00000122861 | PLAU | plasminogen activator, urokinase | 10 | q22.2 |
| ENSG00000101017 | CD40 | CD40 molecule, TNF receptor superfamily member 5 | 20 | q13.12 |
| ENSG00000035403 | VCL | vinculin | 10 | q22.2 |
| ENSG00000163596 | ICA1L | islet cell autoantigen 1,69kDa-like | 2 | q33.2 |
| ENSG00000147202 | DIAPH2 | diaphanous homolog 2 (Drosophila) | X | q21.33 |
| ENSG00000136872 | ALDOB | aldolase B, fructose-bisphosphate | 9 | q31.1 |
| ENSG00000149654 | CDH22 | cadherin 22, type 2 | 20 | q13.12 |
| ENSG00000156110 | ADK | adenosine kinase | 10 | q22.2 |
| ENSG00000156650 | KAT6B | K(lysine) acetyltransferase 6B | 10 | q22.2 |
| ENSG00000165194 | PCDH19 | protocadherin 19 | X | q22.1 |
| ENSG00000062598 | ELMO2 | engulfment and cell motility 2 | 20 | q13.12 |
| ENSG00000178562 | CD28 | CD28 molecule | 2 | q33.2 |
| ENSG00000102359 | SRPX2 | sushi-repeat containing protein, X-linked 2 | X | q22.1 |
| ENSG00000102362 | SYTL4 | synaptotagmin-like 4 | X | q22.1 |
| ENSG00000163599 | CTLA4 | cytotoxic T-lymphocyte-associated protein 4 | 2 | q33.2 |
| ENSG00000163600 | ICOS | inducible T-cell co-stimulator | 2 | q33.2 |
| ENSG00000007952 | NOX1 | NADPH oxidase 1 | X | q22.1 |
| ENSG00000158296 | SLC13A3 | solute carrier family 13 (sodium-dependent dicarboxylate transporter), member 3 | 20 | q13.12 |
| ENSG00000204388 | HSPA1B | heat shock 70kDa protein 1B | 6 | p21.33 |
| ENSG00000118257 | NRP2 | neuropilin 2 | 2 | q33.3 |
| ENSG00000182489 | XKRX | XK, Kell blood group complex subunit-related, X-linked | X | q22.1 |
| ENSG00000172315 | TP53RK | TP53 regulating kinase | 20 | q13.12 |
| ENSG00000023228 | NDUFS1 | NADH dehydrogenase (ubiquinone) Fe-S protein 1, 75kDa (NADH-coenzyme Q reductase) | 2 | q33.3 |
| ENSG00000165029 | ABCA1 | ATP-binding cassette, sub-family A (ABC1), member 1 | 9 | q31.1 |
| ENSG00000064655 | EYA2 | eyes absent homolog 2 (Drosophila) | 20 | q13.12 |
| ENSG00000114948 | ADAM23 | ADAM metallopeptidase domain 23 | 2 | q33.3 |
| ENSG00000118246 | FASTKD2 | FAST kinase domains 2 | 2 | q33.3 |
| ENSG00000118260 | CREB1 | cAMP responsive element binding protein 1 | 2 | q33.3 |
| ENSG00000126953 | TIMM8A | translocase of inner mitochondrial membrane 8 homolog A (yeast) | X | q22.1 |
| ENSG00000156113 | KCNMA1 | potassium large conductance calcium-activated channel, subfamily M, alpha member 1 | 10 | q22.3 |
| ENSG00000010671 | BTK | Bruton agammaglobulinemia tyrosine kinase | X | q22.1 |
| ENSG00000243649 | CFB | complement factor B | 6 | p21.33 |
| ENSG00000163251 | FZD5 | frizzled family receptor 5 | 2 | q33.3 |
| ENSG00000186051 | TAL2 | T-cell acute lymphocytic leukemia 2 | 9 | q31.2 |
| ENSG00000115020 | PIKFYVE | phosphoinositide kinase, FYVE finger containing | 2 | q34 |
| ENSG00000119318 | RAD23B | RAD23 homolog B (S. cerevisiae) | 9 | q31.2 |
| ENSG00000151208 | DLG5 | discs, large homolog 5 (Drosophila) | 10 | q22.3 |
| ENSG00000070061 | IKBKAP | inhibitor of kappa light polypeptide gene enhancer in B-cells, kinase complex-associated protein | 9 | q31.3 |
| ENSG00000168477 | TNXB | tenascin XB | 6 | p21.32 |
| ENSG00000148606 | POLR3A | polymerase (RNA) III (DNA directed) polypeptide A, 155kDa | 10 | q22.3 |
| ENSG00000168530 | MYL1 | myosin, light chain 1, alkali; skeletal, fast | 2 | q34 |
| ENSG00000021826 | CPS1 | carbamoyl-phosphate synthase 1, mitochondrial | 2 | q34 |
| ENSG00000124126 | PREX1 | phosphatidylinositol-3,4,5-trisphosphate-dependent Rac exchange factor 1 | 20 | q13.13 |
| ENSG00000178568 | ERBB4 | v-erb-a erythroblastic leukemia viral oncogene homolog 4 (avian) | 2 | q34 |
| ENSG00000108179 | PPIF | peptidylprolyl isomerase F | 10 | q22.3 |
| ENSG00000119326 | CTNNAL1 | catenin (cadherin-associated protein), alpha-like 1 | 9 | q31.3 |
| ENSG00000124198 | ARFGEF2 | ADP-ribosylation factor guanine nucleotide-exchange factor 2 (brefeldin A-inhibited) | 20 | q13.13 |
| ENSG00000138376 | BARD1 | BRCA1 associated RING domain 1 | 2 | q35 |
| ENSG00000124207 | CSE1L | CSE1 chromosome segregation 1-like (yeast) | 20 | q13.13 |
| ENSG00000133661 | SFTPD | surfactant protein D | 10 | q22.3 |
| ENSG00000115414 | FN1 | fibronectin 1 | 2 | q35 |
| ENSG00000204315 | FKBPL | FK506 binding protein like | 6 | p21.32 |
| ENSG00000122359 | ANXA11 | annexin A11 | 10 | q22.3 |
| ENSG00000165124 | SVEP1 | sushi, von Willebrand factor type A, EGF and pentraxin domain containing 1 | 9 | q31.3 |
| ENSG00000204310 | AGPAT1 | 1-acylglycerol-3-phosphate O-acyltransferase 1 (lysophosphatidic acid acyltransferase, alpha) | 6 | p21.32 |
| ENSG00000204305 | AGER | advanced glycosylation end product-specific receptor | 6 | p21.32 |
| ENSG00000133134 | BEX2 | brain expressed X-linked 2 | X | q22.2 |
| ENSG00000124212 | PTGIS | prostaglandin I2 (prostacyclin) synthase | 20 | q13.13 |
| ENSG00000204304 | PBX2 | pre-B-cell leukemia homeobox 2 | 6 | p21.32 |
| ENSG00000115425 | PECR | peroxisomal trans-2-enoyl-CoA reductase | 2 | q35 |
| ENSG00000166681 | NGFRAP1 | nerve growth factor receptor (TNFRSF16) associated protein 1 | X | q22.2 |
| ENSG00000079246 | XRCC5 | X-ray repair complementing defective repair in Chinese hamster cells 5 (double-strand-break rejoining) | 2 | q35 |
| ENSG00000185737 | NRG3 | neuregulin 3 | 10 | q23.1 |
| ENSG00000124216 | SNAI1 | snail homolog 1 (Drosophila) | 20 | q13.13 |
| ENSG00000136813 | KIAA0368 | KIAA0368 | 9 | q31.3 |
| ENSG00000148600 | CDHR1 | cadherin-related family member 1 | 10 | q23.1 |
| ENSG00000204290 | BTNL2 | butyrophilin-like 2 (MHC class II associated) | 6 | p21.32 |
| ENSG00000204033 | LRIT2 | leucine-rich repeat, immunoglobulin-like and transmembrane domains 2 | 10 | q23.1 |
| ENSG00000204287 | HLA-DRA | major histocompatibility complex, class II, DR alpha | 6 | p21.32 |
| ENSG00000148602 | LRIT1 | leucine-rich repeat, immunoglobulin-like and transmembrane domains 1 | 10 | q23.1 |
| ENSG00000180871 | CXCR2 | chemokine (C-X-C motif) receptor 2 | 2 | q35 |
| ENSG00000163464 | CXCR1 | chemokine (C-X-C motif) receptor 1 | 2 | q35 |
| ENSG00000196301 | HLA-DRB9 | major histocompatibility complex, class II, DR beta 9 (pseudogene) | 6 | p21.32 |
| ENSG00000198502 | HLA-DRB5 | major histocompatibility complex, class II, DR beta 5 | 6 | p21.32 |
| ENSG00000123560 | PLP1 | proteolipid protein 1 | X | q22.2 |
| ENSG00000172216 | CEBPB | CCAAT/enhancer binding protein (C/EBP), beta | 20 | q13.13 |
| ENSG00000196126 | HLA-DRB1 | major histocompatibility complex, class II, DR beta 1 | 6 | p21.32 |
| ENSG00000106853 | PTGR1 | prostaglandin reductase 1 | 9 | q31.3 |
| ENSG00000018280 | SLC11A1 | solute carrier family 11 (proton-coupled divalent metal ion transporters), member 1 | 2 | q35 |
| ENSG00000189108 | IL1RAPL2 | interleukin 1 receptor accessory protein-like 2 | X | q22.3 |
| ENSG00000144580 | RQCD1 | RCD1 required for cell differentiation1 homolog (S. pombe) | 2 | q35 |
| ENSG00000115556 | PLCD4 | phospholipase C, delta 4 | 2 | q35 |
| ENSG00000107779 | BMPR1A | bone morphogenetic protein receptor, type IA | 10 | q23.2 |
| ENSG00000101126 | ADNP | activity-dependent neuroprotector homeobox | 20 | q13.13 |
| ENSG00000123561 | SERPINA7 | serpin peptidase inhibitor, clade A (alpha-1 antiproteinase, antitrypsin), member 7 | X | q22.3 |
| ENSG00000196735 | HLA-DQA1 | major histocompatibility complex, class II, DQ alpha 1 | 6 | p21.32 |
| ENSG00000115592 | PRKAG3 | protein kinase, AMP-activated, gamma 3 non-catalytic subunit | 2 | q35 |
| ENSG00000133135 | RNF128 | ring finger protein 128, E3 ubiquitin protein ligase | X | q22.3 |
| ENSG00000135925 | WNT10A | wingless-type MMTV integration site family, member 10A | 2 | q35 |
| ENSG00000179344 | HLA-DQB1 | major histocompatibility complex, class II, DQ beta 1 | 6 | p21.32 |
| ENSG00000101096 | NFATC2 | nuclear factor of activated T-cells, cytoplasmic, calcineurin-dependent 2 | 20 | q13.2 |
| ENSG00000163501 | IHH | Indian hedgehog | 2 | q35 |
| ENSG00000187736 | NHEJ1 | nonhomologous end-joining factor 1 | 2 | q35 |
| ENSG00000165376 | CLDN2 | claudin 2 | X | q22.3 |
| ENSG00000171862 | PTEN | phosphatase and tensin homolog | 10 | q23.31 |
| ENSG00000204267 | TAP2 | transporter 2, ATP-binding cassette, sub-family B (MDR/TAP) | 6 | p21.32 |
| ENSG00000135924 | DNAJB2 | DnaJ (Hsp40) homolog, subfamily B, member 2 | 2 | q35 |
| ENSG00000119321 | FKBP15 | FK506 binding protein 15, 133kDa | 9 | q32 |
| ENSG00000204264 | PSMB8 | proteasome (prosome, macropain) subunit, beta type, 8 (large multifunctional peptidase 7) | 6 | p21.32 |
| ENSG00000054356 | PTPRN | protein tyrosine phosphatase, receptor type, N | 2 | q35 |
| ENSG00000168394 | TAP1 | transporter 1, ATP-binding cassette, sub-family B (MDR/TAP) | 6 | p21.32 |
| ENSG00000157514 | TSC22D3 | TSC22 domain family, member 3 | X | q22.3 |
| ENSG00000204261 | PSMB9 | proteasome (prosome, macropain) subunit, beta type, 9 (large multifunctional peptidase 2) | 6 | p21.32 |
| ENSG00000204257 | HLA-DMA | major histocompatibility complex, class II, DM alpha | 6 | p21.32 |
| ENSG00000026103 | FAS | Fas (TNF receptor superfamily, member 6) | 10 | q23.31 |
| ENSG00000204252 | HLA-DOA | major histocompatibility complex, class II, DO alpha | 6 | p21.32 |
| ENSG00000101842 | VSIG1 | V-set and immunoglobulin domain containing 1 | X | q22.3 |
| ENSG00000101843 | PSMD10 | proteasome (prosome, macropain) 26S subunit, non-ATPase, 10 | X | q22.3 |
| ENSG00000231389 | HLA-DPA1 | major histocompatibility complex, class II, DP alpha 1 | 6 | p21.32 |
| ENSG00000107798 | LIPA | lipase A, lysosomal acid, cholesterol esterase | 10 | q23.31 |
| ENSG00000119922 | IFIT2 | interferon-induced protein with tetratricopeptide repeats 2 | 10 | q23.31 |
| ENSG00000087589 | CASS4 | Cas scaffolding protein family member 4 | 20 | q13.31 |
| ENSG00000119917 | IFIT3 | interferon-induced protein with tetratricopeptide repeats 3 | 10 | q23.31 |
| ENSG00000197565 | COL4A6 | collagen, type IV, alpha 6 | X | q22.3 |
| ENSG00000106927 | AMBP | alpha-1-microglobulin/bikunin precursor | 9 | q32 |
| ENSG00000204010 | IFIT1B | interferon-induced protein with tetratricopeptide repeats 1B | 10 | q23.31 |
| ENSG00000185745 | IFIT1 | interferon-induced protein with tetratricopeptide repeats 1 | 10 | q23.31 |
| ENSG00000123999 | INHA | inhibin, alpha | 2 | q35 |
| ENSG00000152778 | IFIT5 | interferon-induced protein with tetratricopeptide repeats 5 | 10 | q23.31 |
| ENSG00000196739 | COL27A1 | collagen, type XXVII, alpha 1 | 9 | q32 |
| ENSG00000101144 | BMP7 | bone morphogenetic protein 7 | 20 | q13.31 |
| ENSG00000204248 | COL11A2 | collagen, type XI, alpha 2 | 6 | p21.32 |
| ENSG00000135903 | PAX3 | paired box 3 | 2 | q36.1 |
| ENSG00000068366 | ACSL4 | acyl-CoA synthetase long-chain family member 4 | X | q23 |
| ENSG00000171951 | SCG2 | secretogranin II | 2 | q36.1 |
| ENSG00000152056 | AP1S3 | adaptor-related protein complex 1, sigma 3 subunit | 2 | q36.1 |
| ENSG00000181634 | TNFSF15 | tumor necrosis factor (ligand) superfamily, member 15 | 9 | q32 |
| ENSG00000135919 | SERPINE2 | serpin peptidase inhibitor, clade E (nexin, plasminogen activator inhibitor type 1), member 2 | 2 | q36.1 |
| ENSG00000106952 | TNFSF8 | tumor necrosis factor (ligand) superfamily, member 8 | 9 | q33.1 |
| ENSG00000036257 | CUL3 | cullin 3 | 2 | q36.2 |
| ENSG00000041982 | TNC | tenascin C | 9 | q33.1 |
| ENSG00000135905 | DOCK10 | dedicator of cytokinesis 10 | 2 | q36.2 |
| ENSG00000124256 | ZBP1 | Z-DNA binding protein 1 | 20 | q13.31 |
| ENSG00000169047 | IRS1 | insulin receptor substrate 1 | 2 | q36.3 |
| ENSG00000119912 | IDE | insulin-degrading enzyme | 10 | q23.33 |
| ENSG00000169031 | COL4A3 | collagen, type IV, alpha 3 (Goodpasture antigen) | 2 | q36.3 |
| ENSG00000124209 | RAB22A | RAB22A, member RAS oncogene family | 20 | q13.32 |
| ENSG00000152804 | HHEX | hematopoietically expressed homeobox | 10 | q23.33 |
| ENSG00000138190 | EXOC6 | exocyst complex component 6 | 10 | q23.33 |
| ENSG00000187553 | CYP26C1 | cytochrome P450, family 26, subfamily C, polypeptide 1 | 10 | q23.33 |
| ENSG00000095596 | CYP26A1 | cytochrome P450, family 26, subfamily A, polypeptide 1 | 10 | q23.33 |
| ENSG00000115009 | CCL20 | chemokine (C-C motif) ligand 20 | 2 | q36.3 |
| ENSG00000204209 | DAXX | death-domain associated protein | 6 | p21.32 |
| ENSG00000187957 | DNER | delta/notch-like EGF repeat containing | 2 | q36.3 |
| ENSG00000136869 | TLR4 | toll-like receptor 4 | 9 | q33.1 |
| ENSG00000153827 | TRIP12 | thyroid hormone receptor interactor 12 | 2 | q36.3 |
| ENSG00000138207 | RBP4 | retinol binding protein 4, plasma | 10 | q23.33 |
| ENSG00000112514 | CUTA | cutA divalent cation tolerance homolog (E. coli) | 6 | p21.32 |
| ENSG00000126016 | AMOT | angiomotin | X | q23 |
| ENSG00000030110 | BAK1 | BCL2-antagonist/killer 1 | 6 | p21.31 |
| ENSG00000067066 | SP100 | SP100 nuclear antigen | 2 | q37.1 |
| ENSG00000123496 | IL13RA2 | interleukin 13 receptor, alpha 2 | X | q23 |
| ENSG00000096433 | ITPR3 | inositol 1,4,5-trisphosphate receptor, type 3 | 6 | p21.31 |
| ENSG00000173699 | SPATA3 | spermatogenesis associated 3 | 2 | q37.1 |
| ENSG00000138193 | PLCE1 | phospholipase C, epsilon 1 | 10 | q23.33 |
| ENSG00000173692 | PSMD1 | proteasome (prosome, macropain) 26S subunit, non-ATPase, 1 | 2 | q37.1 |
| ENSG00000135914 | HTR2B | 5-hydroxytryptamine (serotonin) receptor 2B, G protein-coupled | 2 | q37.1 |
| ENSG00000095261 | PSMD5 | proteasome (prosome, macropain) 26S subunit, non-ATPase, 5 | 9 | q33.2 |
| ENSG00000180772 | AGTR2 | angiotensin II receptor, type 2 | X | q23 |
| ENSG00000173145 | NOC3L | nucleolar complex associated 3 homolog (S. cerevisiae) | 10 | q23.33 |
| ENSG00000003096 | KLHL13 | kelch-like 13 (Drosophila) | X | q24 |
| ENSG00000119969 | HELLS | helicase, lymphoid-specific | 10 | q23.33 |
| ENSG00000056558 | TRAF1 | TNF receptor-associated factor 1 | 9 | q33.2 |
| ENSG00000106804 | C5 | complement component 5 | 9 | q33.2 |
| ENSG00000101158 | TH1L | TH1-like (Drosophila) | 20 | q13.32 |
| ENSG00000119397 | CNTRL | centriolin | 9 | q33.2 |
| ENSG00000147251 | DOCK11 | dedicator of cytokinesis 11 | X | q24 |
| ENSG00000101160 | CTSZ | cathepsin Z | 20 | q13.32 |
| ENSG00000066248 | NGEF | neuronal guanine nucleotide exchange factor | 2 | q37.1 |
| ENSG00000148180 | GSN | gelsolin | 9 | q33.2 |
| ENSG00000124205 | EDN3 | endothelin 3 | 20 | q13.32 |
| ENSG00000131724 | IL13RA1 | interleukin 13 receptor, alpha 1 | X | q24 |
| ENSG00000168918 | INPP5D | inositol polyphosphate-5-phosphatase, 145kDa | 2 | q37.1 |
| ENSG00000077044 | DGKD | diacylglycerol kinase, delta 130kDa | 2 | q37.1 |
| ENSG00000196074 | SYCP2 | synaptonemal complex protein 2 | 20 | q13.33 |
| ENSG00000119977 | TCTN3 | tectonic family member 3 | 10 | q24.1 |
| ENSG00000124507 | PACSIN1 | protein kinase C and casein kinase substrate in neurons 1 | 6 | p21.31 |
| ENSG00000138185 | ENTPD1 | ectonucleoside triphosphate diphosphohydrolase 1 | 10 | q24.1 |
| ENSG00000124215 | CDH26 | cadherin 26 | 20 | q13.33 |
| ENSG00000095585 | BLNK | B-cell linker | 10 | q24.1 |
| ENSG00000179242 | CDH4 | cadherin 4, type 1, R-cadherin (retinal) | 20 | q13.33 |
| ENSG00000125354 | SEPT6 | septin 6 | X | q24 |
| ENSG00000149657 | LSM14B | LSM14B, SCD6 homolog B (S. cerevisiae) | 20 | q13.33 |
| ENSG00000095303 | PTGS1 | prostaglandin-endoperoxide synthase 1 (prostaglandin G/H synthase and cyclooxygenase) | 9 | q33.2 |
| ENSG00000101182 | PSMA7 | proteasome (prosome, macropain) subunit, alpha type, 7 | 20 | q13.33 |
| ENSG00000112033 | PPARD | peroxisome proliferator-activated receptor delta | 6 | p21.31 |
| ENSG00000130147 | SH3BP4 | SH3-domain binding protein 4 | 2 | q37.2 |
| ENSG00000112039 | FANCE | Fanconi anemia, complementation group E | 6 | p21.31 |
| ENSG00000168505 | GBX2 | gastrulation brain homeobox 2 | 2 | q37.2 |
| ENSG00000144476 | CXCR7 | chemokine (C-X-C motif) receptor 7 | 2 | q37.3 |
| ENSG00000112041 | TULP1 | tubby like protein 1 | 6 | p21.31 |
| ENSG00000163359 | COL6A3 | collagen, type VI, alpha 3 | 2 | q37.3 |
| ENSG00000187122 | SLIT1 | slit homolog 1 (Drosophila) | 10 | q24.1 |
| ENSG00000101882 | NKAP | NFKB activating protein | X | q24 |
| ENSG00000101180 | HRH3 | histamine receptor H3 | 20 | q13.33 |
| ENSG00000096060 | FKBP5 | FK506 binding protein 5 | 6 | p21.31 |
| ENSG00000130706 | ADRM1 | adhesion regulating molecule 1 | 20 | q13.33 |
| ENSG00000005893 | LAMP2 | lysosomal-associated membrane protein 2 | X | q24 |
| ENSG00000096063 | SRPK1 | SRSF protein kinase 1 | 6 | p21.31 |
| ENSG00000130702 | LAMA5 | laminin, alpha 5 | 20 | q13.33 |
| ENSG00000132323 | ILKAP | integrin-linked kinase-associated serine/threonine phosphatase | 2 | q37.3 |
| ENSG00000165879 | FRAT1 | frequently rearranged in advanced T-cell lymphomas | 10 | q24.1 |
| ENSG00000181274 | FRAT2 | frequently rearranged in advanced T-cell lymphomas 2 | 10 | q24.1 |
| ENSG00000204104 | TRAF3IP1 | TNF receptor-associated factor 3 interacting protein 1 | 2 | q37.3 |
| ENSG00000065802 | ASB1 | ankyrin repeat and SOCS box containing 1 | 2 | q37.3 |
| ENSG00000101898 | MCTS1 | malignant T cell amplified sequence 1 | 20 | q11.21 |
| ENSG00000186891 | TNFRSF18 | tumor necrosis factor receptor superfamily, member 18 | 1 | p36.33 |
| ENSG00000068024 | HDAC4 | histone deacetylase 4 | 2 | q37.3 |
| ENSG00000186827 | TNFRSF4 | tumor necrosis factor receptor superfamily, member 4 | 1 | p36.33 |
| ENSG00000112062 | MAPK14 | mitogen-activated protein kinase 14 | 6 | p21.31 |
| ENSG00000119522 | DENND1A | DENN/MADD domain containing 1A | 9 | q33.3 |
| ENSG00000078808 | SDF4 | stromal cell derived factor 4 | 1 | p36.33 |
| ENSG00000172428 | MYEOV2 | myeloma overexpressed 2 | 2 | q37.3 |
| ENSG00000119408 | NEK6 | NIMA-related kinase 6 | 9 | q33.3 |
| ENSG00000171314 | PGAM1 | phosphoglycerate mutase 1 (brain) | 10 | q24.1 |
| ENSG00000156711 | MAPK13 | mitogen-activated protein kinase 13 | 6 | p21.31 |
| ENSG00000142330 | CAPN10 | calpain 10 | 2 | q37.3 |
| ENSG00000136930 | PSMB7 | proteasome (prosome, macropain) subunit, beta type, 7 | 9 | q33.3 |
| ENSG00000112079 | STK38 | serine/threonine kinase 38 | 6 | p21.31 |
| ENSG00000171307 | ZDHHC16 | zinc finger, DHHC-type containing 16 | 10 | q24.1 |
| ENSG00000124762 | CDKN1A | cyclin-dependent kinase inhibitor 1A (p21, Cip1) | 6 | p21.2 |
| ENSG00000136933 | RABEPK | Rab9 effector protein with kelch motifs | 9 | q33.3 |
| ENSG00000101966 | XIAP | X-linked inhibitor of apoptosis | X | q25 |
| ENSG00000044574 | HSPA5 | heat shock 70kDa protein 5 (glucose-regulated protein, 78kDa) | 9 | q33.3 |
| ENSG00000165219 | GAPVD1 | GTPase activating protein and VPS9 domains 1 | 9 | q33.3 |
| ENSG00000137409 | MTCH1 | mitochondrial carrier 1 | 6 | p21.2 |
| ENSG00000176720 | BOK | BCL2-related ovarian killer | 2 | q37.3 |
| ENSG00000101188 | NTSR1 | neurotensin receptor 1 (high affinity) | 20 | q13.33 |
| ENSG00000167081 | PBX3 | pre-B-cell leukemia homeobox 3 | 9 | q33.3 |
| ENSG00000188389 | PDCD1 | programmed cell death 1 | 2 | q37.3 |
| ENSG00000183918 | SH2D1A | SH2 domain containing 1A | X | q25 |
| ENSG00000146192 | FGD2 | FYVE, RhoGEF and PH domain containing 2 | 6 | p21.2 |
| ENSG00000137193 | PIM1 | pim-1 oncogene | 6 | p21.2 |
| ENSG00000101191 | DIDO1 | death inducer-obliterator 1 | 20 | q13.33 |
| ENSG00000101194 | SLC17A9 | solute carrier family 17, member 9 | 20 | q13.33 |
| ENSG00000120057 | SFRP5 | secreted frizzled-related protein 5 | 10 | q24.2 |
| ENSG00000125533 | BHLHE23 | basic helix-loop-helix family, member e23 | 20 | q13.33 |
| ENSG00000183826 | BTBD9 | BTB (POZ) domain containing 9 | 6 | p21.2 |
| ENSG00000008128 | CDK11A | cyclin-dependent kinase 11A | 1 | p36.33 |
| ENSG00000101197 | BIRC7 | baculoviral IAP repeat containing 7 | 20 | q13.33 |
| ENSG00000124767 | GLO1 | glyoxalase I | 6 | p21.2 |
| ENSG00000107521 | HPS1 | Hermansky-Pudlak syndrome 1 | 10 | q24.2 |
| ENSG00000102034 | ELF4 | E74-like factor 4 (ets domain transcription factor) | X | q26.1 |
| ENSG00000101203 | COL20A1 | collagen, type XX, alpha 1 | 20 | q13.33 |
| ENSG00000156709 | AIFM1 | apoptosis-inducing factor, mitochondrion-associated, 1 | X | q26.1 |
| ENSG00000148356 | LRSAM1 | leucine rich repeat and sterile alpha motif containing 1 | 9 | q33.3 |
| ENSG00000101204 | CHRNA4 | cholinergic receptor, nicotinic, alpha 4 (neuronal) | 20 | q13.33 |
| ENSG00000119919 | NKX2-3 | NK2 homeobox 3 | 10 | q24.2 |
| ENSG00000067606 | PRKCZ | protein kinase C, zeta | 1 | p36.33 |
| ENSG00000136854 | STXBP1 | syntaxin binding protein 1 | 9 | q34.11 |
| ENSG00000157933 | SKI | v-ski sarcoma viral oncogene homolog (avian) | 1 | p36.33 |
| ENSG00000147255 | IGSF1 | immunoglobulin superfamily, member 1 | X | q26.2 |
| ENSG00000196072 | BLOC1S2 | biogenesis of lysosomal organelles complex-1, subunit 2 | 10 | q24.31 |
| ENSG00000197921 | HES5 | hairy and enhancer of split 5 (Drosophila) | 1 | p36.32 |
| ENSG00000157873 | TNFRSF14 | tumor necrosis factor receptor superfamily, member 14 | 1 | p36.32 |
| ENSG00000134602 | MST4 | Serine/threonine-protein kinase MST4 | X | q26.2 |
| ENSG00000142611 | PRDM16 | PR domain containing 16 | 1 | p36.32 |
| ENSG00000161911 | TREML1 | triggering receptor expressed on myeloid cells-like 1 | 6 | p21.1 |
| ENSG00000095970 | TREM2 | triggering receptor expressed on myeloid cells 2 | 6 | p21.1 |
| ENSG00000106991 | ENG | endoglin | 9 | q34.11 |
| ENSG00000130762 | ARHGEF16 | Rho guanine nucleotide exchange factor (GEF) 16 | 1 | p36.32 |
| ENSG00000112195 | TREML2 | triggering receptor expressed on myeloid cells-like 2 | 6 | p21.1 |
| ENSG00000184106 | TREML3P | triggering receptor expressed on myeloid cells-like 3, pseudogene | 6 | p21.1 |
| ENSG00000188056 | TREML4 | triggering receptor expressed on myeloid cells-like 4 | 6 | p21.1 |
| ENSG00000124731 | TREM1 | triggering receptor expressed on myeloid cells 1 | 6 | p21.1 |
| ENSG00000096264 | NCR2 | natural cytotoxicity triggering receptor 2 | 6 | p21.1 |
| ENSG00000095539 | SEMA4G | sema domain, immunoglobulin domain (Ig), transmembrane domain (TM) and short cytoplasmic domain, (semaphorin) 4G | 10 | q24.31 |
| ENSG00000112559 | MDFI | MyoD family inhibitor | 6 | p21.1 |
| ENSG00000147257 | GPC3 | glypican 3 | X | q26.2 |
| ENSG00000078900 | TP73 | tumor protein p73 | 1 | p36.32 |
| ENSG00000165704 | HPRT1 | hypoxanthine phosphoribosyltransferase 1 | X | q26.2 |
| ENSG00000169598 | DFFB | DNA fragmentation factor, 40kDa, beta polypeptide (caspase-activated DNase) | 1 | p36.32 |
| ENSG00000112578 | BYSL | bystin-like | 6 | p21.1 |
| ENSG00000112576 | CCND3 | cyclin D3 | 6 | p21.1 |
| ENSG00000137413 | TAF8 | TAF8 RNA polymerase II, TATA box binding protein (TBP)-associated factor, 43kDa | 6 | p21.1 |
| ENSG00000196581 | AJAP1 | adherens junctions associated protein 1 | 1 | p36.32 |
| ENSG00000107807 | TLX1 | T-cell leukemia homeobox 1 | 10 | q24.31 |
| ENSG00000131697 | NPHP4 | nephronophthisis 4 | 1 | p36.31 |
| ENSG00000166169 | POLL | polymerase (DNA directed), lambda | 10 | q24.32 |
| ENSG00000171169 | NAIF1 | nuclear apoptosis inducing factor 1 | 9 | q34.11 |
| ENSG00000148334 | PTGES2 | prostaglandin E synthase 2 | 9 | q34.11 |
| ENSG00000112619 | PRPH2 | peripherin 2 (retinal degeneration, slow) | 6 | p21.1 |
| ENSG00000171611 | PTCRA | pre T-cell antigen receptor alpha | 6 | p21.1 |
| ENSG00000148346 | LCN2 | lipocalin 2 | 9 | q34.11 |
| ENSG00000101152 | DNAJC5 | DnaJ (Hsp40) homolog, subfamily C, member 5 | 20 | q13.33 |
| ENSG00000116251 | RPL22 | ribosomal protein L22 | 1 | p36.31 |
| ENSG00000102241 | HTATSF1 | HIV-1 Tat specific factor 1 | X | q26.3 |
| ENSG00000102245 | CD40LG | CD40 ligand | X | q26.3 |
| ENSG00000129675 | ARHGEF6 | Rac/Cdc42 guanine nucleotide exchange factor (GEF) 6 | X | q26.3 |
| ENSG00000203883 | SOX18 | SRY (sex determining region Y)-box 18 | 20 | q13.33 |
| ENSG00000171680 | PLEKHG5 | pleckstrin homology domain containing, family G (with RhoGef domain) member 5 | 1 | p36.31 |
| ENSG00000106976 | DNM1 | dynamin 1 | 9 | q34.11 |
| ENSG00000112655 | PTK7 | PTK7 protein tyrosine kinase 7 | 6 | p21.1 |
| ENSG00000112658 | SRF | serum response factor (c-fos serum response element-binding transcription factor) | 6 | p21.1 |
| ENSG00000101977 | MCF2 | MCF.2 cell line derived transforming sequence | X | q27.1 |
| ENSG00000077150 | NFKB2 | nuclear factor of kappa light polypeptide gene enhancer in B-cells 2 (p49/p100) | 10 | q24.32 |
| ENSG00000162413 | KLHL21 | kelch-like 21 (Drosophila) | 1 | p36.31 |
| ENSG00000146215 | CRIP3 | cysteine-rich protein 3 | 6 | p21.1 |
| ENSG00000167123 | CERCAM | cerebral endothelial cell adhesion molecule | 9 | q34.11 |
| ENSG00000041988 | THAP3 | THAP domain containing, apoptosis associated protein 3 | 1 | p36.31 |
| ENSG00000049245 | VAMP3 | vesicle-associated membrane protein 3 | 1 | p36.23 |
| ENSG00000049249 | TNFRSF9 | tumor necrosis factor receptor superfamily, member 9 | 1 | p36.23 |
| ENSG00000148843 | PDCD11 | programmed cell death 11 | 10 | q24.33 |
| ENSG00000160447 | PKN3 | protein kinase N3 | 9 | q34.11 |
| ENSG00000172426 | RSPH9 | radial spoke head 9 homolog (Chlamydomonas) | 6 | p21.1 |
| ENSG00000171603 | CLSTN1 | calsyntenin 1 | 1 | p36.22 |
| ENSG00000065613 | SLK | STE20-like kinase | 10 | q24.33 |
| ENSG00000167136 | ENDOG | endonuclease G | 9 | q34.11 |
| ENSG00000178585 | CTNNBIP1 | catenin, beta interacting protein 1 | 1 | p36.22 |
| ENSG00000112715 | VEGFA | vascular endothelial growth factor A | 6 | p21.1 |
| ENSG00000136802 | LRRC8A | leucine rich repeat containing 8 family, member A | 9 | q34.11 |
| ENSG00000102181 | CD99L2 | CD99 molecule-like 2 | X | q28 |
| ENSG00000130939 | UBE4B | ubiquitination factor E4B | 1 | p36.22 |
| ENSG00000054523 | KIF1B | kinesin family member 1B | 1 | p36.22 |
| ENSG00000175279 | APITD1 | apoptosis-inducing, TAF9-like domain 1 | 1 | p36.22 |
| ENSG00000119383 | PPP2R4 | protein phosphatase 2A activator, regulatory subunit 4 | 9 | q34.11 |
| ENSG00000160049 | DFFA | DNA fragmentation factor, 45kDa, alpha polypeptide | 1 | p36.22 |
| ENSG00000148700 | ADD3 | adducin 3 (gamma) | 10 | q25.1 |
| ENSG00000148335 | NTMT1 | N-terminal Xaa-Pro-Lys N-methyltransferase 1 | 9 | q34.11 |
| ENSG00000009724 | MASP2 | mannan-binding lectin serine peptidase 2 | 1 | p36.22 |
| ENSG00000148344 | PTGES | prostaglandin E synthase | 9 | q34.11 |
| ENSG00000198793 | MTOR | mechanistic target of rapamycin (serine/threonine kinase) | 1 | p36.22 |
| ENSG00000147400 | CETN2 | centrin, EF-hand protein, 2 | X | q28 |
| ENSG00000096384 | HSP90AB1 | heat shock protein 90kDa alpha (cytosolic), class B member 1 | 6 | p21.1 |
| ENSG00000136878 | USP20 | ubiquitin specific peptidase 20 | 9 | q34.11 |
| ENSG00000119953 | SMNDC1 | survival motor neuron domain containing 1 | 10 | q25.2 |
| ENSG00000187239 | FNBP1 | formin binding protein 1 | 9 | q34.11 |
| ENSG00000107130 | NCS1 | neuronal calcium sensor 1 | 9 | q34.11 |
| ENSG00000130707 | ASS1 | argininosuccinate synthase 1 | 9 | q34.11 |
| ENSG00000150593 | PDCD4 | programmed cell death 4 (neoplastic transformation inhibitor) | 10 | q25.2 |
| ENSG00000146232 | NFKBIE | nuclear factor of kappa light polypeptide gene enhancer in B-cells inhibitor, epsilon | 6 | p21.1 |
| ENSG00000097007 | ABL1 | c-abl oncogene 1, non-receptor tyrosine kinase | 9 | q34.12 |
| ENSG00000050555 | LAMC3 | laminin, gamma 3 | 9 | q34.12 |
| ENSG00000150594 | ADRA2A | adrenoceptor alpha 2A | 10 | q25.2 |
| ENSG00000119927 | GPAM | glycerol-3-phosphate acyltransferase, mitochondrial | 10 | q25.2 |
| ENSG00000126878 | AIF1L | allograft inflammatory factor 1-like | 9 | q34.12 |
| ENSG00000185825 | BCAP31 | B-cell receptor-associated protein 31 | X | q28 |
| ENSG00000175206 | NPPA | natriuretic peptide A | 1 | p36.22 |
| ENSG00000148737 | TCF7L2 | transcription factor 7-like 2 (T-cell specific, HMG-box) | 10 | q25.2 |
| ENSG00000130723 | PRRC2B | proline-rich coiled-coil 2B | 9 | q34.13 |
| ENSG00000146070 | PLA2G7 | phospholipase A2, group VII (platelet-activating factor acetylhydrolase, plasma) | 6 | p12.3 |
| ENSG00000148702 | HABP2 | hyaluronan binding protein 2 | 10 | q25.3 |
| ENSG00000197893 | NRAP | nebulin-related anchoring protein | 10 | q25.3 |
| ENSG00000120949 | TNFRSF8 | tumor necrosis factor receptor superfamily, member 8 | 1 | p36.22 |
| ENSG00000146072 | TNFRSF21 | tumor necrosis factor receptor superfamily, member 21 | 6 | p12.3 |
| ENSG00000165806 | CASP7 | caspase 7, apoptosis-related cysteine peptidase | 10 | q25.3 |
| ENSG00000198087 | CD2AP | CD2-associated protein | 6 | p12.3 |
| ENSG00000028137 | TNFRSF1B | tumor necrosis factor receptor superfamily, member 1B | 1 | p36.22 |
| ENSG00000198910 | L1CAM | L1 cell adhesion molecule | X | q28 |
| ENSG00000043591 | ADRB1 | adrenoceptor beta 1 | 10 | q25.3 |
| ENSG00000125492 | BARHL1 | BarH-like homeobox 1 | 9 | q34.13 |
| ENSG00000177684 | DEFB114 | defensin, beta 114 | 6 | p12.3 |
| ENSG00000203970 | DEFB110 | defensin, beta 110 locus | 6 | p12.3 |
| ENSG00000180872 | DEFB112 | defensin, beta 112 | 6 | p12.3 |
| ENSG00000169129 | AFAP1L2 | actin filament associated protein 1-like 2 | 10 | q25.3 |
| ENSG00000170927 | PKHD1 | polycystic kidney and hepatic disease 1 (autosomal recessive) | 6 | p12.2 |
| ENSG00000112115 | IL17A | interleukin 17A | 6 | p12.2 |
| ENSG00000112116 | IL17F | interleukin 17F | 6 | p12.2 |
| ENSG00000089820 | ARHGAP4 | Rho GTPase activating protein 4 | X | q28 |
| ENSG00000162493 | PDPN | podoplanin | 1 | p36.21 |
| ENSG00000172534 | HCFC1 | host cell factor C1 (VP16-accessory protein) | X | q28 |
| ENSG00000184216 | IRAK1 | interleukin-1 receptor-associated kinase 1 | X | q28 |
| ENSG00000132906 | CASP9 | caspase 9, apoptosis-related cysteine peptidase | 1 | p36.21 |
| ENSG00000175164 | ABO | ABO blood group (transferase A, alpha 1-3-N-acetylgalactosaminyltransferase; transferase B, alpha 1-3-galactosyltransferase) | 9 | q34.2 |
| ENSG00000112144 | ICK | intestinal cell (MAK-like) kinase | 6 | p12.1 |
| ENSG00000007350 | TKTL1 | transketolase-like 1 | X | q28 |
| ENSG00000196924 | FLNA | filamin A, alpha | X | q28 |
| ENSG00000001084 | GCLC | glutamate-cysteine ligase, catalytic subunit | 6 | p12.1 |
| ENSG00000165672 | PRDX3 | peroxiredoxin 3 | 10 | q26.11 |
| ENSG00000137251 | TINAG | tubulointerstitial nephritis antigen | 6 | p12.1 |
| ENSG00000162458 | FBLIM1 | filamin binding LIM protein 1 | 1 | p36.21 |
| ENSG00000198873 | GRK5 | G protein-coupled receptor kinase 5 | 10 | q26.11 |
| ENSG00000187871 | GFRAL | GDNF family receptor alpha like | 6 | p12.1 |
| ENSG00000151923 | TIAL1 | TIA1 cytotoxic granule-associated RNA binding protein-like 1 | 10 | q26.11 |
| ENSG00000112175 | BMP5 | bone morphogenetic protein 5 | 6 | p12.1 |
| ENSG00000151929 | BAG3 | BCL2-associated athanogene 3 | 10 | q26.11 |
| ENSG00000124749 | COL21A1 | collagen, type XXI, alpha 1 | 6 | p12.1 |
| ENSG00000151914 | DST | dystonin | 6 | p12.1 |
| ENSG00000160323 | ADAMTS13 | ADAM metallopeptidase with thrombospondin type 1 motif, 13 | 9 | q34.2 |
| ENSG00000142627 | EPHA2 | EPH receptor A2 | 1 | p36.13 |
| ENSG00000157191 | NECAP2 | NECAP endocytosis associated 2 | 1 | p36.13 |
| ENSG00000123454 | DBH | dopamine beta-hydroxylase (dopamine beta-monooxygenase) | 9 | q34.2 |
| ENSG00000186301 | MST1P2 | macrophage stimulating 1 (hepatocyte growth factor-like) pseudogene 2 | 1 | p36.13 |
| ENSG00000112208 | BAG2 | BCL2-associated athanogene 2 | 6 | p12.1 |
| ENSG00000160293 | VAV2 | vav 2 guanine nucleotide exchange factor | 9 | q34.2 |
| ENSG00000186715 | MST1P9 | macrophage stimulating 1-like | 1 | p36.13 |
| ENSG00000130827 | PLXNA3 | plexin A3 | X | q28 |
| ENSG00000146143 | PRIM2 | primase, DNA, polypeptide 2 (58kDa) | 6 | p11.2 |
| ENSG00000198225 | FKBP1C | FK506 binding protein 1C | 6 | q12 |
| ENSG00000186350 | RXRA | retinoid X receptor, alpha | 9 | q34.2 |
| ENSG00000160211 | G6PD | glucose-6-phosphate dehydrogenase | X | q28 |
| ENSG00000130635 | COL5A1 | collagen, type V, alpha 1 | 9 | q34.3 |
| ENSG00000160339 | FCN2 | ficolin (collagen/fibrinogen domain containing lectin) 2 (hucolin) | 9 | q34.3 |
| ENSG00000085265 | FCN1 | ficolin (collagen/fibrinogen domain containing) 1 | 9 | q34.3 |
| ENSG00000107679 | PLEKHA1 | pleckstrin homology domain containing, family A (phosphoinositide binding specific) member 1 | 10 | q26.13 |
| ENSG00000166033 | HTRA1 | HtrA serine peptidase 1 | 10 | q26.13 |
| ENSG00000082293 | COL19A1 | collagen, type XIX, alpha 1 | 6 | q13 |
| ENSG00000187908 | DMBT1 | deleted in malignant brain tumors 1 | 10 | q26.13 |
| ENSG00000112280 | COL9A1 | collagen, type IX, alpha 1 | 6 | q13 |
| ENSG00000009709 | PAX7 | paired box 7 | 1 | p36.13 |
| ENSG00000130830 | MPP1 | membrane protein, palmitoylated 1, 55kDa | X | q28 |
| ENSG00000185010 | F8 | coagulation factor VIII, procoagulant component | X | q28 |
| ENSG00000079841 | RIMS1 | regulating synaptic membrane exocytosis 1 | 6 | q13 |
| ENSG00000138161 | CUZD1 | CUB and zona pellucida-like domains 1 | 10 | q26.13 |
| ENSG00000187796 | CARD9 | caspase recruitment domain family, member 9 | 9 | q34.3 |
| ENSG00000182712 | MTCP1NB | C-x(9)-C motif containing 4 homolog (S. cerevisiae) | X | q28 |
| ENSG00000148400 | NOTCH1 | notch 1 | 9 | q34.3 |
| ENSG00000169692 | AGPAT2 | 1-acylglycerol-3-phosphate O-acyltransferase 2 (lysophosphatidic acid acyltransferase, beta) | 9 | q34.3 |
| ENSG00000156535 | CD109 | CD109 molecule | 6 | q13 |
| ENSG00000121898 | CPXM2 | carboxypeptidase X (M14 family), member 2 | 10 | q26.13 |
| ENSG00000124333 | VAMP7 | vesicle-associated membrane protein 7 | X | q28 |
| ENSG00000124334 | IL9R | interleukin 9 receptor | X | q28 |
| ENSG00000111799 | COL12A1 | collagen, type XII, alpha 1 | 6 | q14.1 |
| ENSG00000203791 | METTL10 | methyltransferase like 10 | 10 | q26.13 |
| ENSG00000158748 | HTR6 | 5-hydroxytryptamine (serotonin) receptor 6, G protein-coupled | 1 | p36.13 |
| ENSG00000175029 | CTBP2 | C-terminal binding protein 2 | 10 | q26.13 |
| ENSG00000177943 | MAMDC4 | MAM domain containing 4 | 9 | q34.3 |
| ENSG00000188257 | PLA2G2A | phospholipase A2, group IIA (platelets, synovial fluid) | 1 | p36.13 |
| ENSG00000127472 | PLA2G5 | phospholipase A2, group V | 1 | p36.13 |
| ENSG00000127191 | TRAF2 | TNF receptor-associated factor 2 | 9 | q34.3 |
| ENSG00000176919 | C8G | complement component 8, gamma polypeptide | 9 | q34.3 |
| ENSG00000090432 | MUL1 | mitochondrial E3 ubiquitin protein ligase 1 | 1 | p36.12 |
| ENSG00000107317 | PTGDS | prostaglandin D2 synthase 21kDa (brain) | 9 | q34.3 |
| ENSG00000158828 | PINK1 | PTEN induced putative kinase 1 | 1 | p36.12 |
| ENSG00000148848 | ADAM12 | ADAM metallopeptidase domain 12 | 10 | q26.2 |
| ENSG00000054179 | ENTPD2 | ectonucleoside triphosphate diphosphohydrolase 2 | 9 | q34.3 |
| ENSG00000117298 | ECE1 | endothelin converting enzyme 1 | 1 | p36.12 |
| ENSG00000150760 | DOCK1 | dedicator of cytokinesis 1 | 10 | q26.2 |
| ENSG00000132334 | PTPRE | protein tyrosine phosphatase, receptor type, E | 10 | q26.2 |
| ENSG00000196586 | MYO6 | myosin VI | 6 | q14.1 |
| ENSG00000148773 | MKI67 | antigen identified by monoclonal antibody Ki-67 | 10 | q26.2 |
| ENSG00000170430 | MGMT | O-6-methylguanine-DNA methyltransferase | 10 | q26.3 |
| ENSG00000162551 | ALPL | alkaline phosphatase, liver/bone/kidney | 1 | p36.12 |
| ENSG00000108001 | EBF3 | early B-cell factor 3 | 10 | q26.3 |
| ENSG00000108010 | GLRX3 | glutaredoxin 3 | 10 | q26.3 |
| ENSG00000188229 | TUBB4B | tubulin, beta 4B class IVb | 9 | q34.3 |
| ENSG00000176171 | BNIP3 | BCL2/adenovirus E1B 19kDa interacting protein 3 | 10 | q26.3 |
| ENSG00000188385 | JAKMIP3 | Janus kinase and microtubule interacting protein 3 | 10 | q26.3 |
| ENSG00000146243 | IRAK1BP1 | interleukin-1 receptor-associated kinase 1 binding protein 1 | 6 | q14.1 |
| ENSG00000188747 | NOXA1 | NADPH oxidase activator 1 | 9 | q34.3 |
| ENSG00000146247 | PHIP | pleckstrin homology domain interacting protein | 6 | q14.1 |
| ENSG00000142798 | HSPG2 | heparan sulfate proteoglycan 2 | 1 | p36.12 |
| ENSG00000182154 | MRPL41 | mitochondrial ribosomal protein L41 | 9 | q34.3 |
| ENSG00000070831 | CDC42 | cell division cycle 42 (GTP binding protein, 25kDa) | 1 | p36.12 |
| ENSG00000162552 | WNT4 | wingless-type MMTV integration site family, member 4 | 1 | p36.12 |
| ENSG00000005700 | IBTK | inhibitor of Bruton agammaglobulinemia tyrosine kinase | 6 | q14.1 |
| ENSG00000146242 | TPBG | trophoblast glycoprotein | 6 | q14.1 |
| ENSG00000130643 | CALY | calcyon neuron-specific vesicular protein | 10 | q26.3 |
| ENSG00000173372 | C1QA | complement component 1, q subcomponent, A chain | 1 | p36.12 |
| ENSG00000159189 | C1QC | complement component 1, q subcomponent, C chain | 1 | p36.12 |
| ENSG00000173369 | C1QB | complement component 1, q subcomponent, B chain | 1 | p36.12 |
| ENSG00000065609 | SNAP91 | synaptosomal-associated protein, 91kDa homolog (mouse) | 6 | q14.2 |
| ENSG00000135324 | MRAP2 | melanocortin 2 receptor accessory protein 2 | 6 | q14.2 |
| ENSG00000007968 | E2F2 | E2F transcription factor 2 | 1 | p36.12 |
| ENSG00000117318 | ID3 | inhibitor of DNA binding 3, dominant negative helix-loop-helix protein | 1 | p36.12 |
| ENSG00000142676 | RPL11 | ribosomal protein L11 | 1 | p36.11 |
| ENSG00000135318 | NT5E | 5'-nucleotidase, ecto (CD73) | 6 | q14.3 |
| ENSG00000188822 | CNR2 | cannabinoid receptor 2 (macrophage) | 1 | p36.11 |
| ENSG00000142677 | IL22RA1 | interleukin 22 receptor, alpha 1 | 1 | p36.11 |
| ENSG00000185436 | IL28RA | interleukin 28 receptor, alpha (interferon, lambda receptor) | 1 | p36.11 |
| ENSG00000135334 | AKIRIN2 | akirin 2 | 6 | q15 |
| ENSG00000118432 | CNR1 | cannabinoid receptor 1 (brain) | 6 | q15 |
| ENSG00000020633 | RUNX3 | runt-related transcription factor 3 | 1 | p36.11 |
| ENSG00000187010 | RHD | Rh blood group, D antigen | 1 | p36.11 |
| ENSG00000188672 | RHCE | Rh blood group, CcEe antigens | 1 | p36.11 |
| ENSG00000118412 | CASP8AP2 | caspase 8 associated protein 2 | 6 | q15 |
| ENSG00000157978 | LDLRAP1 | low density lipoprotein receptor adaptor protein 1 | 1 | p36.11 |
| ENSG00000135341 | MAP3K7 | mitogen-activated protein kinase kinase kinase 7 | 6 | q15 |
| ENSG00000135333 | EPHA7 | EPH receptor A7 | 6 | q16.1 |
| ENSG00000158006 | PAFAH2 | platelet-activating factor acetylhydrolase 2, 40kDa | 1 | p36.11 |
| ENSG00000158008 | EXTL1 | exostoses (multiple)-like 1 | 1 | p36.11 |
| ENSG00000169442 | CD52 | CD52 molecule | 1 | p36.11 |
| ENSG00000117676 | RPS6KA1 | ribosomal protein S6 kinase, 90kDa, polypeptide 1 | 1 | p36.11 |
| ENSG00000164418 | GRIK2 | glutamate receptor, ionotropic, kainate 2 | 6 | q16.3 |
| ENSG00000175793 | SFN | stratifin | 1 | p36.11 |
| ENSG00000112276 | BVES | blood vessel epicardial substance | 6 | q21 |
| ENSG00000090020 | SLC9A1 | solute carrier family 9, subfamily A (NHE1, cation proton antiporter 1), member 1 | 1 | p36.11 |
| ENSG00000142765 | SYTL1 | synaptotagmin-like 1 | 1 | p36.11 |
| ENSG00000057663 | ATG5 | autophagy related 5 | 6 | q21 |
| ENSG00000142748 | FCN3 | ficolin (collagen/fibrinogen domain containing) 3 (Hakata antigen) | 1 | p36.11 |
| ENSG00000174950 | CD164L2 | CD164 sialomucin-like 2 | 1 | p36.11 |
| ENSG00000158195 | WASF2 | WAS protein family, member 2 | 1 | p36.11 |
| ENSG00000126709 | IFI6 | interferon, alpha-inducible protein 6 | 1 | p35.3 |
| ENSG00000112335 | SNX3 | sorting nexin 3 | 6 | q21 |
| ENSG00000118689 | FOXO3 | forkhead box O3 | 6 | q21 |
| ENSG00000130775 | THEMIS2 | thymocyte selection associated family member 2 | 1 | p35.3 |
| ENSG00000135535 | CD164 | CD164 molecule, sialomucin | 6 | q21 |
| ENSG00000185250 | PPIL6 | peptidylprolyl isomerase (cyclophilin)-like 6 | 6 | q21 |
| ENSG00000135587 | SMPD2 | sphingomyelin phosphodiesterase 2, neutral membrane (neutral sphingomyelinase) | 6 | q21 |
| ENSG00000158156 | XKR8 | XK, Kell blood group complex subunit-related family, member 8 | 1 | p35.3 |
| ENSG00000169403 | PTAFR | platelet-activating factor receptor | 1 | p35.3 |
| ENSG00000155111 | CDK19 | cyclin-dependent kinase 19 | 6 | q21 |
| ENSG00000060656 | PTPRU | protein tyrosine phosphatase, receptor type, U | 1 | p35.3 |
| ENSG00000162512 | SDC3 | syndecan 3 | 1 | p35.2 |
| ENSG00000056972 | TRAF3IP2 | TRAF3 interacting protein 2 | 6 | q21 |
| ENSG00000010810 | FYN | FYN oncogene related to SRC, FGR, YES | 6 | q21 |
| ENSG00000084636 | COL16A1 | collagen, type XVI, alpha 1 | 1 | p35.2 |
| ENSG00000112769 | LAMA4 | laminin, alpha 4 | 6 | q21 |
| ENSG00000196591 | HDAC2 | histone deacetylase 2 | 6 | q21 |
| ENSG00000084652 | TXLNA | taxilin alpha | 1 | p35.2 |
| ENSG00000111816 | FRK | fyn-related kinase | 6 | q22.1 |
| ENSG00000182866 | LCK | lymphocyte-specific protein tyrosine kinase | 1 | p35.2 |
| ENSG00000111834 | RSPH4A | radial spoke head 4 homolog A (Chlamydomonas) | 6 | q22.1 |
| ENSG00000116478 | HDAC1 | histone deacetylase 1 | 1 | p35.2 |
| ENSG00000185002 | RFX6 | regulatory factor X, 6 | 6 | q22.1 |
| ENSG00000134684 | YARS | tyrosyl-tRNA synthetase | 1 | p35.1 |
| ENSG00000164465 | DCBLD1 | discoidin, CUB and LCCL domain containing 1 | 6 | q22.1 |
| ENSG00000152661 | GJA1 | gap junction protein, alpha 1, 43kDa | 6 | q22.31 |
| ENSG00000111907 | TPD52L1 | tumor protein D52-like 1 | 6 | q22.31 |
| ENSG00000126067 | PSMB2 | proteasome (prosome, macropain) subunit, beta type, 2 | 1 | p34.3 |
| ENSG00000172673 | THEMIS | thymocyte selection associated | 6 | q22.33 |
| ENSG00000152894 | PTPRK | protein tyrosine phosphatase, receptor type, K | 6 | q22.33 |
| ENSG00000171812 | COL8A2 | collagen, type VIII, alpha 2 | 1 | p34.3 |
| ENSG00000196569 | LAMA2 | laminin, alpha 2 | 6 | q22.33 |
| ENSG00000119535 | CSF3R | colony stimulating factor 3 receptor (granulocyte) | 1 | p34.3 |
| ENSG00000163874 | ZC3H12A | zinc finger CCCH-type containing 12A | 1 | p34.3 |
| ENSG00000183520 | UTP11L | UTP11-like, U3 small nucleolar ribonucleoprotein, (yeast) | 1 | p34.3 |
| ENSG00000116954 | RRAGC | Ras-related GTP binding C | 1 | p34.3 |
| ENSG00000197594 | ENPP1 | ectonucleotide pyrophosphatase/phosphodiesterase 1 | 6 | q23.2 |
| ENSG00000118523 | CTGF | connective tissue growth factor | 6 | q23.2 |
| ENSG00000112299 | VNN1 | vanin 1 | 6 | q23.2 |
| ENSG00000183682 | BMP8A | bone morphogenetic protein 8a | 1 | p34.3 |
| ENSG00000118526 | TCF21 | transcription factor 21 | 6 | q23.2 |
| ENSG00000084072 | PPIE | peptidylprolyl isomerase E (cyclophilin E) | 1 | p34.2 |
| ENSG00000116985 | BMP8B | bone morphogenetic protein 8b | 1 | p34.2 |
| ENSG00000118515 | SGK1 | serum/glucocorticoid regulated kinase 1 | 6 | q23.2 |
| ENSG00000131236 | CAP1 | CAP, adenylate cyclase-associated protein 1 (yeast) | 1 | p34.2 |
| ENSG00000131238 | PPT1 | palmitoyl-protein thioesterase 1 | 1 | p34.2 |
| ENSG00000029363 | BCLAF1 | BCL2-associated transcription factor 1 | 6 | q23.3 |
| ENSG00000197442 | MAP3K5 | mitogen-activated protein kinase kinase kinase 5 | 6 | q23.3 |
| ENSG00000117016 | RIMS3 | regulating synaptic membrane exocytosis 3 | 1 | p34.2 |
| ENSG00000016402 | IL20RA | interleukin 20 receptor, alpha | 6 | q23.3 |
| ENSG00000164485 | IL22RA2 | interleukin 22 receptor, alpha 2 | 6 | q23.3 |
| ENSG00000027697 | IFNGR1 | interferon gamma receptor 1 | 6 | q23.3 |
| ENSG00000118503 | TNFAIP3 | tumor necrosis factor, alpha-induced protein 3 | 6 | q23.3 |
| ENSG00000112378 | PERP | PERP, TP53 apoptosis effector | 6 | q23.3 |
| ENSG00000171790 | SLFNL1 | schlafen-like 1 | 1 | p34.2 |
| ENSG00000127129 | EDN2 | endothelin 2 | 1 | p34.2 |
| ENSG00000127124 | HIVEP3 | human immunodeficiency virus type I enhancer binding protein 3 | 1 | p34.2 |
| ENSG00000164442 | CITED2 | Cbp/p300-interacting transactivator, with Glu/Asp-rich carboxy-terminal domain, 2 | 6 | q24.1 |
| ENSG00000171960 | PPIH | peptidylprolyl isomerase H (cyclophilin H) | 1 | p34.2 |
| ENSG00000164007 | CLDN19 | claudin 19 | 1 | p34.2 |
| ENSG00000010818 | HIVEP2 | human immunodeficiency virus type I enhancer binding protein 2 | 6 | q24.2 |
| ENSG00000164010 | ERMAP | erythroblast membrane-associated protein (Scianna blood group) | 1 | p34.2 |
| ENSG00000189007 | ADAT2 | adenosine deaminase, tRNA-specific 2 | 6 | q24.2 |
| ENSG00000066056 | TIE1 | tyrosine kinase with immunoglobulin-like and EGF-like domains 1 | 1 | p34.2 |
| ENSG00000118495 | PLAGL1 | pleiomorphic adenoma gene-like 1 | 6 | q24.2 |
| ENSG00000117400 | MPL | myeloproliferative leukemia virus oncogene | 1 | p34.2 |
| ENSG00000142949 | PTPRF | protein tyrosine phosphatase, receptor type, F | 1 | p34.2 |
| ENSG00000164506 | STXBP5 | syntaxin binding protein 5 (tomosyn) | 6 | q24.3 |
| ENSG00000131013 | PPIL4 | peptidylprolyl isomerase (cyclophilin)-like 4 | 6 | q25.1 |
| ENSG00000164520 | RAET1E | retinoic acid early transcript 1E | 6 | q25.1 |
| ENSG00000203722 | RAET1G | retinoic acid early transcript 1G | 6 | q25.1 |
| ENSG00000131015 | ULBP2 | UL16 binding protein 2 | 6 | q25.1 |
| ENSG00000111981 | ULBP1 | UL16 binding protein 1 | 6 | q25.1 |
| ENSG00000155918 | RAET1L | retinoic acid early transcript 1L | 6 | q25.1 |
| ENSG00000131019 | ULBP3 | UL16 binding protein 3 | 6 | q25.1 |
| ENSG00000117450 | PRDX1 | peroxiredoxin 1 | 1 | p34.1 |
| ENSG00000086015 | MAST2 | microtubule associated serine/threonine kinase 2 | 1 | p34.1 |
| ENSG00000079277 | MKNK1 | MAP kinase interacting serine/threonine kinase 1 | 1 | p33 |
| ENSG00000112038 | OPRM1 | opioid receptor, mu 1 | 6 | q25.2 |
| ENSG00000146426 | TIAM2 | T-cell lymphoma invasion and metastasis 2 | 6 | q25.2 |
| ENSG00000162367 | TAL1 | T-cell acute lymphocytic leukemia 1 | 1 | p33 |
| ENSG00000186564 | FOXD2 | forkhead box D2 | 1 | p33 |
| ENSG00000171217 | CLDN20 | claudin 20 | 6 | q25.3 |
| ENSG00000117834 | SLC5A9 | solute carrier family 5 (sodium/glucose cotransporter), member 9 | 1 | p33 |
| ENSG00000185104 | FAF1 | Fas (TNFRSF6) associated factor 1 | 1 | p32.3 |
| ENSG00000123080 | CDKN2C | cyclin-dependent kinase inhibitor 2C (p18, inhibits CDK4) | 1 | p32.3 |
| ENSG00000146425 | DYNLT1 | dynein, light chain, Tctex-type 1 | 6 | q25.3 |
| ENSG00000092820 | EZR | ezrin | 6 | q25.3 |
| ENSG00000130363 | RSPH3 | radial spoke 3 homolog (Chlamydomonas) | 6 | q25.3 |
| ENSG00000164691 | TAGAP | T-cell activation RhoGTPase activating protein | 6 | q25.3 |
| ENSG00000112096 | SOD2 | superoxide dismutase 2, mitochondrial | 6 | q25.3 |
| ENSG00000169213 | RAB3B | RAB3B, member RAS oncogene family | 1 | p32.3 |
| ENSG00000130368 | MAS1 | MAS1 oncogene | 6 | q25.3 |
| ENSG00000197081 | IGF2R | insulin-like growth factor 2 receptor | 6 | q25.3 |
| ENSG00000157077 | ZFYVE9 | zinc finger, FYVE domain containing 9 | 1 | p32.3 |
| ENSG00000134744 | ZCCHC11 | zinc finger, CCHC domain containing 11 | 1 | p32.3 |
| ENSG00000157193 | LRP8 | low density lipoprotein receptor-related protein 8, apolipoprotein e receptor | 1 | p32.3 |
| ENSG00000122194 | PLG | plasminogen | 6 | q26 |
| ENSG00000085511 | MAP3K4 | mitogen-activated protein kinase kinase kinase 4 | 6 | q26 |
| ENSG00000081870 | HSPB11 | heat shock protein family B (small), member 11 | 1 | p32.3 |
| ENSG00000071242 | RPS6KA2 | ribosomal protein S6 kinase, 90kDa, polypeptide 2 | 6 | q27 |
| ENSG00000112486 | CCR6 | chemokine (C-C motif) receptor 6 | 6 | q27 |
| ENSG00000116133 | DHCR24 | 24-dehydrocholesterol reductase | 1 | p32.3 |
| ENSG00000169174 | PCSK9 | proprotein convertase subtilisin/kexin type 9 | 1 | p32.3 |
| ENSG00000130396 | MLLT4 | myeloid/lymphoid or mixed-lineage leukemia (trithorax homolog, Drosophila); translocated to, 4 | 6 | q27 |
| ENSG00000162407 | PPAP2B | phosphatidic acid phosphatase type 2B | 1 | p32.2 |
| ENSG00000162409 | PRKAA2 | protein kinase, AMP-activated, alpha 2 catalytic subunit | 1 | p32.2 |
| ENSG00000157131 | C8A | complement component 8, alpha polypeptide | 1 | p32.2 |
| ENSG00000021852 | C8B | complement component 8, beta polypeptide | 1 | p32.2 |
| ENSG00000173406 | DAB1 | disabled homolog 1 (Drosophila) | 1 | p32.1 |
| ENSG00000112562 | SMOC2 | SPARC related modular calcium binding 2 | 6 | q27 |
| ENSG00000186340 | THBS2 | thrombospondin 2 | 6 | q27 |
| ENSG00000177606 | JUN | jun proto-oncogene | 1 | p32.1 |
| ENSG00000198719 | DLL1 | delta-like 1 (Drosophila) | 6 | q27 |
| ENSG00000008018 | PSMB1 | proteasome (prosome, macropain) subunit, beta type, 1 | 6 | q27 |
| ENSG00000162604 | TM2D1 | TM2 domain containing 1 | 1 | p31.3 |
| ENSG00000071994 | PDCD2 | programmed cell death 2 | 6 | q27 |
| ENSG00000116641 | DOCK7 | dedicator of cytokinesis 7 | 1 | p31.3 |
| ENSG00000132855 | ANGPTL3 | angiopoietin-like 3 | 1 | p31.3 |
| ENSG00000187140 | FOXD3 | forkhead box D3 | 1 | p31.3 |
| ENSG00000142856 | ITGB3BP | integrin beta 3 binding protein (beta3-endonexin) | 1 | p31.3 |
| ENSG00000116652 | DLEU2L | deleted in lymphocytic leukemia 2-like | 1 | p31.3 |
| ENSG00000162434 | JAK1 | Janus kinase 1 | 1 | p31.3 |
| ENSG00000116678 | LEPR | leptin receptor | 1 | p31.3 |
| ENSG00000162594 | IL23R | interleukin 23 receptor | 1 | p31.3 |
| ENSG00000081985 | IL12RB2 | interleukin 12 receptor, beta 2 | 1 | p31.3 |
| ENSG00000142864 | SERBP1 | SERPINE1 mRNA binding protein 1 | 1 | p31.3 |
| ENSG00000116717 | GADD45A | growth arrest and DNA-damage-inducible, alpha | 1 | p31.3 |
| ENSG00000172380 | GNG12 | guanine nucleotide binding protein (G protein), gamma 12 | 1 | p31.3 |
| ENSG00000050628 | PTGER3 | prostaglandin E receptor 3 (subtype EP3) | 1 | p31.1 |
| ENSG00000172260 | NEGR1 | neuronal growth regulator 1 | 1 | p31.1 |
| ENSG00000077254 | USP33 | ubiquitin specific peptidase 33 | 1 | p31.1 |
| ENSG00000122420 | PTGFR | prostaglandin F receptor (FP) | 1 | p31.1 |
| ENSG00000137959 | IFI44L | interferon-induced protein 44-like | 1 | p31.1 |
| ENSG00000137965 | IFI44 | interferon-induced protein 44 | 1 | p31.1 |
| ENSG00000142875 | PRKACB | protein kinase, cAMP-dependent, catalytic, beta | 1 | p31.1 |
| ENSG00000117155 | SSX2IP | synovial sarcoma, X breakpoint 2 interacting protein | 1 | p22.3 |
| ENSG00000142867 | BCL10 | B-cell CLL/lymphoma 10 | 1 | p22.3 |
| ENSG00000142871 | CYR61 | cysteine-rich, angiogenic inducer, 61 | 1 | p22.3 |
| ENSG00000171502 | COL24A1 | collagen, type XXIV, alpha 1 | 1 | p22.3 |
| ENSG00000137975 | CLCA2 | chloride channel accessory 2 | 1 | p22.3 |
| ENSG00000097033 | SH3GLB1 | SH3-domain GRB2-like endophilin B1 | 1 | p22.3 |
| ENSG00000065243 | PKN2 | protein kinase N2 | 1 | p22.2 |
| ENSG00000117228 | GBP1 | guanylate binding protein 1, interferon-inducible | 1 | p22.2 |
| ENSG00000162645 | GBP2 | guanylate binding protein 2, interferon-inducible | 1 | p22.2 |
| ENSG00000069702 | TGFBR3 | transforming growth factor, beta receptor III | 1 | p22.1 |
| ENSG00000174842 | GLMN | glomulin, FKBP associated protein | 1 | p22.1 |
| ENSG00000137942 | FNBP1L | formin binding protein 1-like | 1 | p22.1 |
| ENSG00000023909 | GCLM | glutamate-cysteine ligase, modifier subunit | 1 | p22.1 |
| ENSG00000117525 | F3 | coagulation factor III (thromboplastin, tissue factor) | 1 | p21.3 |
| ENSG00000162692 | VCAM1 | vascular cell adhesion molecule 1 | 1 | p21.2 |
| ENSG00000170989 | S1PR1 | sphingosine-1-phosphate receptor 1 | 1 | p21.2 |
| ENSG00000060718 | COL11A1 | collagen, type XI, alpha 1 | 1 | p21.1 |
| ENSG00000134215 | VAV3 | vav 3 guanine nucleotide exchange factor | 1 | p13.3 |
| ENSG00000116266 | STXBP3 | syntaxin binding protein 3 | 1 | p13.3 |
| ENSG00000143126 | CELSR2 | cadherin, EGF LAG seven-pass G-type receptor 2 (flamingo homolog, Drosophila) | 1 | p13.3 |
| ENSG00000134243 | SORT1 | sortilin 1 | 1 | p13.3 |
| ENSG00000143106 | PSMA5 | proteasome (prosome, macropain) subunit, alpha type, 5 | 1 | p13.3 |
| ENSG00000181754 | AMIGO1 | adhesion molecule with Ig-like domain 1 | 1 | p13.3 |
| ENSG00000184371 | CSF1 | colony stimulating factor 1 (macrophage) | 1 | p13.3 |
| ENSG00000156150 | ALX3 | ALX homeobox 3 | 1 | p13.3 |
| ENSG00000162775 | RBM15 | RNA binding motif protein 15 | 1 | p13.3 |
| ENSG00000134248 | HBXIP | late endosomal/lysosomal adaptor, MAPK and MTOR activator 5 | 1 | p13.3 |
| ENSG00000143119 | CD53 | CD53 molecule | 1 | p13.3 |
| ENSG00000156171 | DRAM2 | DNA-damage regulated autophagy modulator 2 | 1 | p13.3 |
| ENSG00000134216 | CHIA | chitinase, acidic | 1 | p13.2 |
| ENSG00000064703 | DDX20 | DEAD (Asp-Glu-Ala-Asp) box polypeptide 20 | 1 | p13.2 |
| ENSG00000155363 | MOV10 | Mov10, Moloney leukemia virus 10, homolog (mouse) | 1 | p13.2 |
| ENSG00000184599 | FAM19A3 | family with sequence similarity 19 (chemokine (C-C motif)-like), member A3 | 1 | p13.2 |
| ENSG00000198799 | LRIG2 | leucine-rich repeats and immunoglobulin-like domains 2 | 1 | p13.2 |
| ENSG00000081026 | MAGI3 | membrane associated guanylate kinase, WW and PDZ domain containing 3 | 1 | p13.2 |
| ENSG00000134242 | PTPN22 | protein tyrosine phosphatase, non-receptor type 22 (lymphoid) | 1 | p13.2 |
| ENSG00000188761 | BCL2L15 | BCL2-like 15 | 1 | p13.2 |
| ENSG00000163349 | HIPK1 | homeodomain interacting protein kinase 1 | 1 | p13.2 |
| ENSG00000197323 | TRIM33 | tripartite motif containing 33 | 1 | p13.2 |
| ENSG00000134259 | NGF | nerve growth factor (beta polypeptide) | 1 | p13.2 |
| ENSG00000116815 | CD58 | CD58 molecule | 1 | p13.1 |
| ENSG00000143061 | IGSF3 | immunoglobulin superfamily, member 3 | 1 | p13.1 |
| ENSG00000116824 | CD2 | CD2 molecule | 1 | p13.1 |
| ENSG00000134247 | PTGFRN | prostaglandin F2 receptor negative regulator | 1 | p13.1 |
| ENSG00000134256 | CD101 | CD101 molecule | 1 | p13.1 |
| ENSG00000134258 | VTCN1 | V-set domain containing T cell activation inhibitor 1 | 1 | p12 |
| ENSG00000134250 | NOTCH2 | notch 2 | 1 | p12 |
| ENSG00000198019 | FCGR1B | Fc fragment of IgG, high affinity Ib, receptor (CD64) | 1 | p11.2 |
| ENSG00000143127 | ITGA10 | integrin, alpha 10 | 1 | q21.1 |
| ENSG00000186141 | POLR3C | polymerase (RNA) III (DNA directed) polypeptide C (62kD) | 1 | q21.1 |
| ENSG00000117281 | CD160 | CD160 molecule | 1 | q21.1 |
| ENSG00000131791 | PRKAB2 | protein kinase, AMP-activated, beta 2 non-catalytic subunit | 1 | q21.1 |
| ENSG00000116128 | BCL9 | B-cell CLL/lymphoma 9 | 1 | q21.2 |
| ENSG00000143429 | LOC645166 | lymphocyte-specific protein 1 pseudogene (LOC654342), non-coding RNA | 2 | p11.2 |
| ENSG00000150337 | FCGR1A | Fc fragment of IgG, high affinity Ia, receptor (CD64) | 1 | q21.2 |
| ENSG00000136631 | VPS45 | vacuolar protein sorting 45 homolog (S. cerevisiae) | 1 | q21.2 |
| ENSG00000117362 | APH1A | anterior pharynx defective 1 homolog A (C. elegans) | 1 | q21.2 |
| ENSG00000143382 | ADAMTSL4 | ADAMTS-like 4 | 1 | q21.2 |
| ENSG00000143384 | MCL1 | myeloid cell leukemia sequence 1 (BCL2-related) | 1 | q21.2 |
| ENSG00000163131 | CTSS | cathepsin S | 1 | q21.3 |
| ENSG00000143387 | CTSK | cathepsin K | 1 | q21.3 |
| ENSG00000143412 | ANXA9 | annexin A9 | 1 | q21.3 |
| ENSG00000163141 | BNIPL | BCL2/adenovirus E1B 19kD interacting protein like | 1 | q21.3 |
| ENSG00000197622 | CDC42SE1 | CDC42 small effector 1 | 1 | q21.3 |
| ENSG00000163154 | TNFAIP8L2 | tumor necrosis factor, alpha-induced protein 8-like 2 | 1 | q21.3 |
| ENSG00000159352 | PSMD4 | proteasome (prosome, macropain) 26S subunit, non-ATPase, 4 | 1 | q21.3 |
| ENSG00000143393 | PI4KB | phosphatidylinositol 4-kinase, catalytic, beta | 1 | q21.3 |
| ENSG00000143390 | RFX5 | regulatory factor X, 5 (influences HLA class II expression) | 1 | q21.3 |
| ENSG00000159377 | PSMB4 | proteasome (prosome, macropain) subunit, beta type, 4 | 1 | q21.3 |
| ENSG00000143536 | CRNN | cornulin | 1 | q21.3 |
| ENSG00000163220 | S100A9 | S100 calcium binding protein A9 | 1 | q21.3 |
| ENSG00000143546 | S100A8 | S100 calcium binding protein A8 | 1 | q21.3 |
| ENSG00000143556 | S100A7 | S100 calcium binding protein A7 | 1 | q21.3 |
| ENSG00000189334 | S100A14 | S100 calcium binding protein A14 | 1 | q21.3 |
| ENSG00000189171 | S100A13 | S100 calcium binding protein A13 | 1 | q21.3 |
| ENSG00000143553 | SNAPIN | SNAP-associated protein | 1 | q21.3 |
| ENSG00000143621 | ILF2 | interleukin enhancer binding factor 2, 45kDa | 1 | q21.3 |
| ENSG00000143545 | RAB13 | RAB13, member RAS oncogene family | 1 | q21.3 |
| ENSG00000143575 | HAX1 | HCLS1 associated protein X-1 | 1 | q21.3 |
| ENSG00000160712 | IL6R | interleukin 6 receptor | 1 | q21.3 |
| ENSG00000160716 | CHRNB2 | cholinergic receptor, nicotinic, beta 2 (neuronal) | 1 | q21.3 |
| ENSG00000160710 | ADAR | adenosine deaminase, RNA-specific | 1 | q21.3 |
| ENSG00000163346 | PBXIP1 | pre-B-cell leukemia homeobox interacting protein 1 | 1 | q21.3 |
| ENSG00000143537 | ADAM15 | ADAM metallopeptidase domain 15 | 1 | q21.3 |
| ENSG00000169241 | SLC50A1 | solute carrier family 50 (sugar transporter), member 1 | 1 | q22 |
| ENSG00000169231 | THBS3 | thrombospondin 3 | 1 | q22 |
| ENSG00000116521 | SCAMP3 | secretory carrier membrane protein 3 | 1 | q22 |
| ENSG00000132676 | DAP3 | death associated protein 3 | 1 | q22 |
| ENSG00000116584 | ARHGEF2 | Rho/Rac guanine nucleotide exchange factor (GEF) 2 | 1 | q22 |
| ENSG00000196189 | SEMA4A | sema domain, immunoglobulin domain (Ig), transmembrane domain (TM) and short cytoplasmic domain, (semaphorin) 4A | 1 | q22 |
| ENSG00000116604 | MEF2D | myocyte enhancer factor 2D | 1 | q22 |
| ENSG00000132702 | HAPLN2 | hyaluronan and proteoglycan link protein 2 | 1 | q23.1 |
| ENSG00000132692 | BCAN | brevican | 1 | q23.1 |
| ENSG00000143319 | ISG20L2 | interferon stimulated exonuclease gene 20kDa-like 2 | 1 | q23.1 |
| ENSG00000198400 | NTRK1 | neurotrophic tyrosine kinase, receptor, type 1 | 1 | q23.1 |
| ENSG00000187800 | PEAR1 | platelet endothelial aggregation receptor 1 | 1 | q23.1 |
| ENSG00000132694 | ARHGEF11 | Rho guanine nucleotide exchange factor (GEF) 11 | 1 | q23.1 |
| ENSG00000143297 | FCRL5 | Fc receptor-like 5 | 1 | q23.1 |
| ENSG00000163518 | FCRL4 | Fc receptor-like 4 | 1 | q23.1 |
| ENSG00000160856 | FCRL3 | Fc receptor-like 3 | 1 | q23.1 |
| ENSG00000132704 | FCRL2 | Fc receptor-like 2 | 1 | q23.1 |
| ENSG00000163534 | FCRL1 | Fc receptor-like 1 | 1 | q23.1 |
| ENSG00000073754 | CD5L | CD5 molecule-like | 1 | q23.1 |
| ENSG00000158473 | CD1D | CD1d molecule | 1 | q23.1 |
| ENSG00000158477 | CD1A | CD1a molecule | 1 | q23.1 |
| ENSG00000158481 | CD1C | CD1c molecule | 1 | q23.1 |
| ENSG00000158485 | CD1B | CD1b molecule | 1 | q23.1 |
| ENSG00000158488 | CD1E | CD1e molecule | 1 | q23.1 |
| ENSG00000163563 | MNDA | myeloid cell nuclear differentiation antigen | 1 | q23.1 |
| ENSG00000163565 | IFI16 | interferon, gamma-inducible protein 16 | 1 | q23.1 |
| ENSG00000162706 | CADM3 | cell adhesion molecule 3 | 1 | q23.2 |
| ENSG00000179639 | FCER1A | Fc fragment of IgE, high affinity I, receptor for; alpha polypeptide | 1 | q23.2 |
| ENSG00000132703 | APCS | amyloid P component, serum | 1 | q23.2 |
| ENSG00000132693 | CRP | C-reactive protein, pentraxin-related | 1 | q23.2 |
| ENSG00000181036 | FCRL6 | Fc receptor-like 6 | 1 | q23.2 |
| ENSG00000085552 | IGSF9 | immunoglobulin superfamily, member 9 | 1 | q23.2 |
| ENSG00000162729 | IGSF8 | immunoglobulin superfamily, member 8 | 1 | q23.2 |
| ENSG00000143318 | CASQ1 | calsequestrin 1 (fast-twitch, skeletal muscle) | 1 | q23.2 |
| ENSG00000162734 | PEA15 | phosphoprotein enriched in astrocytes 15 | 1 | q23.2 |
| ENSG00000162736 | NCSTN | nicastrin | 1 | q23.2 |
| ENSG00000066294 | CD84 | CD84 molecule | 1 | q23.3 |
| ENSG00000117090 | SLAMF1 | signaling lymphocytic activation molecule family member 1 | 1 | q23.3 |
| ENSG00000117091 | CD48 | CD48 molecule | 1 | q23.3 |
| ENSG00000026751 | SLAMF7 | SLAM family member 7 | 1 | q23.3 |
| ENSG00000122224 | LY9 | lymphocyte antigen 9 | 1 | q23.3 |
| ENSG00000122223 | CD244 | CD244 molecule, natural killer cell receptor 2B4 | 1 | q23.3 |
| ENSG00000158769 | F11R | F11 receptor | 1 | q23.3 |
| ENSG00000143217 | PVRL4 | poliovirus receptor-related 4 | 1 | q23.3 |
| ENSG00000158796 | DEDD | death effector domain containing | 1 | q23.3 |
| ENSG00000158869 | FCER1G | Fc fragment of IgE, high affinity I, receptor for; gamma polypeptide | 1 | q23.3 |
| ENSG00000158874 | APOA2 | apolipoprotein A-II | 1 | q23.3 |
| ENSG00000143226 | FCGR2A | Fc fragment of IgG, low affinity IIa, receptor (CD32) | 1 | q23.3 |
| ENSG00000203747 | FCGR3A | Fc fragment of IgG, low affinity IIIa, receptor (CD16a) | 1 | q23.3 |
| ENSG00000162747 | FCGR3B | Fc fragment of IgG, low affinity IIIb, receptor (CD16b) | 1 | q23.3 |
| ENSG00000072694 | FCGR2B | Fc fragment of IgG, low affinity IIb, receptor (CD32) | 1 | q23.3 |
| ENSG00000132185 | FCRLA | Fc receptor-like A | 1 | q23.3 |
| ENSG00000162746 | FCRLB | Fc receptor-like B | 1 | q23.3 |
| ENSG00000162733 | DDR2 | discoidin domain receptor tyrosine kinase 2 | 1 | q23.3 |
| ENSG00000185630 | PBX1 | pre-B-cell leukemia homeobox 1 | 1 | q23.3 |
| ENSG00000143195 | ILDR2 | immunoglobulin-like domain containing receptor 2 | 1 | q24.1 |
| ENSG00000143194 | MAEL | maelstrom homolog (Drosophila) | 1 | q24.1 |
| ENSG00000198821 | CD247 | CD247 molecule | 1 | q24.2 |
| ENSG00000143199 | ADCY10 | adenylate cyclase 10 (soluble) | 1 | q24.2 |
| ENSG00000143185 | XCL2 | chemokine (C motif) ligand 2 | 1 | q24.2 |
| ENSG00000143184 | XCL1 | chemokine (C motif) ligand 1 | 1 | q24.2 |
| ENSG00000143196 | DPT | dermatopontin | 1 | q24.2 |
| ENSG00000198734 | F5 | coagulation factor V (proaccelerin, labile factor) | 1 | q24.2 |
| ENSG00000174175 | SELP | selectin P (granule membrane protein 140kDa, antigen CD62) | 1 | q24.2 |
| ENSG00000188404 | SELL | selectin L | 1 | q24.2 |
| ENSG00000007908 | SELE | selectin E | 1 | q24.2 |
| ENSG00000075945 | KIFAP3 | kinesin-associated protein 3 | 1 | q24.2 |
| ENSG00000203740 | METTL11B | methyltransferase like 11B | 1 | q24.2 |
| ENSG00000010932 | FMO1 | flavin containing monooxygenase 1 | 1 | q24.3 |
| ENSG00000117523 | PRRC2C | proline-rich coiled-coil 2C | 1 | q24.3 |
| ENSG00000010165 | METTL13 | methyltransferase like 13 | 1 | q24.3 |
| ENSG00000197959 | DNM3 | dynamin 3 | 1 | q24.3 |
| ENSG00000117560 | FASLG | Fas ligand (TNF superfamily, member 6) | 1 | q24.3 |
| ENSG00000120337 | TNFSF18 | tumor necrosis factor (ligand) superfamily, member 18 | 1 | q25.1 |
| ENSG00000117586 | TNFSF4 | tumor necrosis factor (ligand) superfamily, member 4 | 1 | q25.1 |
| ENSG00000076321 | KLHL20 | kelch-like 20 (Drosophila) | 1 | q25.1 |
| ENSG00000120332 | TNN | tenascin N | 1 | q25.1 |
| ENSG00000116147 | TNR | tenascin R | 1 | q25.1 |
| ENSG00000152092 | ASTN1 | astrotactin 1 | 1 | q25.2 |
| ENSG00000116194 | ANGPTL1 | angiopoietin-like 1 | 1 | q25.2 |
| ENSG00000143322 | ABL2 | v-abl Abelson murine leukemia viral oncogene homolog 2 | 1 | q25.2 |
| ENSG00000057252 | SOAT1 | sterol O-acyltransferase 1 | 1 | q25.2 |
| ENSG00000121454 | LHX4 | LIM homeobox 4 | 1 | q25.2 |
| ENSG00000153029 | MR1 | major histocompatibility complex, class I-related | 1 | q25.3 |
| ENSG00000135862 | LAMC1 | laminin, gamma 1 (formerly LAMB2) | 1 | q25.3 |
| ENSG00000058085 | LAMC2 | laminin, gamma 2 | 1 | q25.3 |
| ENSG00000116701 | NCF2 | neutrophil cytosolic factor 2 | 1 | q25.3 |
| ENSG00000198756 | GLT25D2 | glycosyltransferase 25 domain containing 2 | 1 | q25.3 |
| ENSG00000073756 | PTGS2 | prostaglandin-endoperoxide synthase 2 (prostaglandin G/H synthase and cyclooxygenase) | 1 | q31.1 |
| ENSG00000116711 | PLA2G4A | phospholipase A2, group IVA (cytosolic, calcium-dependent) | 1 | q31.1 |
| ENSG00000116741 | RGS2 | regulator of G-protein signaling 2, 24kDa | 1 | q31.2 |
| ENSG00000116750 | UCHL5 | ubiquitin carboxyl-terminal hydrolase L5 | 1 | q31.2 |
| ENSG00000023572 | GLRX2 | glutaredoxin 2 | 1 | q31.2 |
| ENSG00000000971 | CFH | complement factor H | 1 | q31.3 |
| ENSG00000116785 | CFHR3 | complement factor H-related 3 | 1 | q31.3 |
| ENSG00000080910 | CFHR2 | complement factor H-related 2 | 1 | q31.3 |
| ENSG00000134365 | CFHR4 | complement factor H-related 4 | 1 | q31.3 |
| ENSG00000134389 | CFHR5 | complement factor H-related 5 | 1 | q31.3 |
| ENSG00000081237 | PTPRC | protein tyrosine phosphatase, receptor type, C | 1 | q31.3 |
| ENSG00000163395 | IGFN1 | immunoglobulin-like and fibronectin type III domain containing 1 | 1 | q32.1 |
| ENSG00000081277 | PKP1 | plakophilin 1 (ectodermal dysplasia/skin fragility syndrome) | 1 | q32.1 |
| ENSG00000174307 | PHLDA3 | pleckstrin homology-like domain, family A, member 3 | 1 | q32.1 |
| ENSG00000163485 | ADORA1 | adenosine A1 receptor | 1 | q32.1 |
| ENSG00000133055 | MYBPH | myosin binding protein H | 1 | q32.1 |
| ENSG00000159388 | BTG2 | BTG family, member 2 | 1 | q32.1 |
| ENSG00000122176 | FMOD | fibromodulin | 1 | q32.1 |
| ENSG00000122188 | LAX1 | lymphocyte transmembrane adaptor 1 | 1 | q32.1 |
| ENSG00000198625 | MDM4 | Mdm4 p53 binding protein homolog (mouse) | 1 | q32.1 |
| ENSG00000170382 | LRRN2 | leucine rich repeat neuronal 2 | 1 | q32.1 |
| ENSG00000163531 | NFASC | neurofascin | 1 | q32.1 |
| ENSG00000184144 | CNTN2 | contactin 2 (axonal) | 1 | q32.1 |
| ENSG00000163545 | NUAK2 | NUAK family, SNF1-like kinase, 2 | 1 | q32.1 |
| ENSG00000198049 | AVPR1B | arginine vasopressin receptor 1B | 1 | q32.1 |
| ENSG00000196188 | CTSE | cathepsin E | 1 | q32.1 |
| ENSG00000136634 | IL10 | interleukin 10 | 1 | q32.1 |
| ENSG00000142224 | IL19 | interleukin 19 | 1 | q32.1 |
| ENSG00000162891 | IL20 | interleukin 20 | 1 | q32.1 |
| ENSG00000162892 | IL24 | interleukin 24 | 1 | q32.1 |
| ENSG00000162894 | FAIM3 | Fas apoptotic inhibitory molecule 3 | 1 | q32.1 |
| ENSG00000162896 | PIGR | polymeric immunoglobulin receptor | 1 | q32.1 |
| ENSG00000162897 | FCAMR | Fc receptor, IgA, IgM, high affinity | 1 | q32.1 |
| ENSG00000123843 | C4BPB | complement component 4 binding protein, beta | 1 | q32.1 |
| ENSG00000123838 | C4BPA | complement component 4 binding protein, alpha | 1 | q32.2 |
| ENSG00000196352 | CD55 | CD55 molecule, decay accelerating factor for complement (Cromer blood group) | 1 | q32.2 |
| ENSG00000117322 | CR2 | complement component (3d/Epstein Barr virus) receptor 2 | 1 | q32.2 |
| ENSG00000203710 | CR1 | complement component (3b/4b) receptor 1 (Knops blood group) | 1 | q32.2 |
| ENSG00000197721 | CR1L | complement component (3b/4b) receptor 1-like | 1 | q32.2 |
| ENSG00000117335 | CD46 | CD46 molecule, complement regulatory protein | 1 | q32.2 |
| ENSG00000174059 | CD34 | CD34 molecule | 1 | q32.2 |
| ENSG00000008118 | CAMK1G | calcium/calmodulin-dependent protein kinase IG | 1 | q32.2 |
| ENSG00000196878 | LAMB3 | laminin, beta 3 | 1 | q32.2 |
| ENSG00000117595 | IRF6 | interferon regulatory factor 6 | 1 | q32.2 |
| ENSG00000082512 | TRAF5 | TNF receptor-associated factor 5 | 1 | q32.3 |
| ENSG00000162769 | FLVCR1 | feline leukemia virus subgroup C cellular receptor 1 | 1 | q32.3 |
| ENSG00000117707 | PROX1 | prospero homeobox 1 | 1 | q32.3 |
| ENSG00000092969 | TGFB2 | transforming growth factor, beta 2 | 1 | q41 |
| ENSG00000116141 | MARK1 | MAP/microtubule affinity-regulating kinase 1 | 1 | q41 |
| ENSG00000154305 | MIA3 | melanoma inhibitory activity family, member 3 | 1 | q41 |
| ENSG00000187554 | TLR5 | toll-like receptor 5 | 1 | q41 |
| ENSG00000143514 | TP53BP2 | tumor protein p53 binding protein, 2 | 1 | q41 |
| ENSG00000143768 | LEFTY2 | left-right determination factor 2 | 1 | q42.12 |
| ENSG00000143772 | ITPKB | inositol-trisphosphate 3-kinase B | 1 | q42.12 |
| ENSG00000143801 | PSEN2 | presenilin 2 (Alzheimer disease 4) | 1 | q42.13 |
| ENSG00000154342 | WNT3A | wingless-type MMTV integration site family, member 3A | 1 | q42.13 |
| ENSG00000154358 | OBSCN | obscurin, cytoskeletal calmodulin and titin-interacting RhoGEF | 1 | q42.13 |
| ENSG00000168118 | RAB4A | RAB4A, member RAS oncogene family | 1 | q42.13 |
| ENSG00000143641 | GALNT2 | UDP-N-acetyl-alpha-D-galactosamine:polypeptide N-acetylgalactosaminyltransferase 2 (GalNAc-T2) | 1 | q42.13 |
| ENSG00000135744 | AGT | angiotensinogen (serpin peptidase inhibitor, clade A, member 8) | 1 | q42.2 |
| ENSG00000116903 | EXOC8 | exocyst complex component 8 | 1 | q42.2 |
| ENSG00000059588 | TARBP1 | TAR (HIV-1) RNA binding protein 1 | 1 | q42.2 |
| ENSG00000168264 | IRF2BP2 | interferon regulatory factor 2 binding protein 2 | 1 | q42.3 |
| ENSG00000143669 | LYST | lysosomal trafficking regulator | 1 | q42.3 |
| ENSG00000116962 | NID1 | nidogen 1 | 1 | q42.3 |
| ENSG00000077522 | ACTN2 | actinin, alpha 2 | 1 | q43 |
| ENSG00000198626 | RYR2 | ryanodine receptor 2 (cardiac) | 1 | q43 |
| ENSG00000180875 | GREM2 | gremlin 2 | 1 | q43 |
| ENSG00000174371 | EXO1 | exonuclease 1 | 1 | q43 |
| ENSG00000153207 | AHCTF1 | AT hook containing transcription factor 1 | 1 | q44 |
| ENSG00000162711 | NLRP3 | NLR family, pyrin domain containing 3 | 1 | q44 |
| ENSG00000176998 | HCG4 | HLA complex group 4 (non-protein coding) | 6 | p22.1 |
| ENSG00000177839 | PCDHB9 | protocadherin beta 9 | 5 | q31.3 |
| ENSG00000183514 | TDGF1P2 | teratocarcinoma-derived growth factor 1 pseudogene 2 | 2 | q36.3 |
| ENSG00000203618 | GP1BB | glycoprotein Ib (platelet), beta polypeptide | 22 | q11.21 |
| ENSG00000211592 | IGKC | immunoglobulin kappa constant | 2 | p11.2 |
| ENSG00000211593 | IGKJ5 | immunoglobulin kappa joining 5 | 2 | p11.2 |
| ENSG00000211594 | IGKJ4 | immunoglobulin kappa joining 4 | 2 | p11.2 |
| ENSG00000211595 | IGKJ3 | immunoglobulin kappa joining 3 | 2 | p11.2 |
| ENSG00000211596 | IGKJ2 | immunoglobulin kappa joining 2 | 2 | p11.2 |
| ENSG00000211597 | IGKJ1 | immunoglobulin kappa joining 1 | 2 | p11.2 |
| ENSG00000211598 | IGKV4-1 | immunoglobulin kappa variable 4-1 | 2 | p11.2 |
| ENSG00000211599 | IGKV5-2 | immunoglobulin kappa variable 5-2 | 2 | p11.2 |
| ENSG00000211611 | IGKV6-21 | immunoglobulin kappa variable 6-21 (non-functional) | 2 | p11.2 |
| ENSG00000211623 | IGKV2D-26 | immunoglobulin kappa variable 2D-26 | 2 | p11.2 |
| ENSG00000211625 | IGKV3D-20 | immunoglobulin kappa variable 3D-20 | 2 | p11.2 |
| ENSG00000211626 | IGKV6D-41 | immunoglobulin kappa variable 6D-41 (non-functional) | 2 | p11.2 |
| ENSG00000211632 | IGKV3D-11 | immunoglobulin kappa variable 3D-11 | 2 | p11.2 |
| ENSG00000211633 | IGKV1D-42 | immunoglobulin kappa variable 1D-42 (non-functional) | 2 | p11.2 |
| ENSG00000211637 | IGLV4-69 | immunoglobulin lambda variable 4-69 | 22 | q11.22 |
| ENSG00000211638 | IGLV8-61 | immunoglobulin lambda variable 8-61 | 22 | q11.22 |
| ENSG00000211639 | IGLV4-60 | immunoglobulin lambda variable 4-60 | 22 | q11.22 |
| ENSG00000211640 | IGLV6-57 | immunoglobulin lambda variable 6-57 | 22 | q11.22 |
| ENSG00000211641 | IGLV11-55 | immunoglobulin lambda variable 11-55 (non-functional) | 22 | q11.22 |
| ENSG00000211642 | IGLV10-54 | immunoglobulin lambda variable 10-54 | 22 | q11.22 |
| ENSG00000211643 | IGLV5-52 | immunoglobulin lambda variable 5-52 | 22 | q11.22 |
| ENSG00000211644 | IGLV1-51 | immunoglobulin lambda variable 1-51 | 22 | q11.22 |
| ENSG00000211645 | IGLV1-50 | immunoglobulin lambda variable 1-50 (non-functional) | 22 | q11.22 |
| ENSG00000211647 | IGLV5-48 | immunoglobulin lambda variable 5-48 (non-functional) | 22 | q11.22 |
| ENSG00000211648 | IGLV1-47 | immunoglobulin lambda variable 1-47 | 22 | q11.22 |
| ENSG00000211649 | IGLV7-46 | immunoglobulin lambda variable 7-46 (gene/pseudogene) | 22 | q11.22 |
| ENSG00000211650 | IGLV5-45 | immunoglobulin lambda variable 5-45 | 22 | q11.22 |
| ENSG00000211651 | IGLV1-44 | immunoglobulin lambda variable 1-44 | 22 | q11.22 |
| ENSG00000211652 | IGLV7-43 | immunoglobulin lambda variable 7-43 | 22 | q11.22 |
| ENSG00000211653 | IGLV1-40 | immunoglobulin lambda variable 1-40 | 22 | q11.22 |
| ENSG00000211654 | IGLV5-37 | immunoglobulin lambda variable 5-37 | 22 | q11.22 |
| ENSG00000211655 | IGLV1-36 | immunoglobulin lambda variable 1-36 | 22 | q11.22 |
| ENSG00000211656 | IGLV2-33 | immunoglobulin lambda variable 2-33 (non-functional) | 22 | q11.22 |
| ENSG00000211657 | IGLV3-32 | immunoglobulin lambda variable 3-32 (non-functional) | 22 | q11.22 |
| ENSG00000211658 | IGLV3-27 | immunoglobulin lambda variable 3-27 | 22 | q11.22 |
| ENSG00000211659 | IGLV3-25 | immunoglobulin lambda variable 3-25 | 22 | q11.22 |
| ENSG00000211660 | IGLV2-23 | immunoglobulin lambda variable 2-23 | 22 | q11.22 |
| ENSG00000211661 | IGLV3-22 | immunoglobulin lambda variable 3-22 (gene/pseudogene) | 22 | q11.22 |
| ENSG00000211662 | IGLV3-21 | immunoglobulin lambda variable 3-21 | 22 | q11.22 |
| ENSG00000211663 | IGLV3-19 | immunoglobulin lambda variable 3-19 | 22 | q11.22 |
| ENSG00000211664 | IGLV2-18 | immunoglobulin lambda variable 2-18 | 22 | q11.22 |
| ENSG00000211665 | IGLV3-16 | immunoglobulin lambda variable 3-16 | 22 | q11.22 |
| ENSG00000211666 | IGLV2-14 | immunoglobulin lambda variable 2-14 | 22 | q11.22 |
| ENSG00000211667 | IGLV3-12 | immunoglobulin lambda variable 3-12 | 22 | q11.22 |
| ENSG00000211668 | IGLV2-11 | immunoglobulin lambda variable 2-11 | 22 | q11.22 |
| ENSG00000211669 | IGLV3-10 | immunoglobulin lambda variable 3-10 | 22 | q11.22 |
| ENSG00000211670 | IGLV3-9 | immunoglobulin lambda variable 3-9 (gene/pseudogene) | 22 | q11.22 |
| ENSG00000211672 | IGLV4-3 | immunoglobulin lambda variable 4-3 | 22 | q11.22 |
| ENSG00000211673 | IGLV3-1 | immunoglobulin lambda variable 3-1 | 22 | q11.22 |
| ENSG00000211674 | IGLJ1 | immunoglobulin lambda joining 1 | 22 | q11.22 |
| ENSG00000211675 | IGLC1 | immunoglobulin lambda constant 1 (Mcg marker) | 22 | q11.22 |
| ENSG00000211676 | IGLJ2 | immunoglobulin lambda joining 2 | 22 | q11.22 |
| ENSG00000211677 | IGLC2 | immunoglobulin lambda constant 2 (Kern-Oz- marker) | 22 | q11.22 |
| ENSG00000211678 | IGLJ3 | immunoglobulin lambda joining 3 | 22 | q11.22 |
| ENSG00000211679 | IGLC3 | immunoglobulin lambda constant 3 (Kern-Oz+ marker) | 22 | q11.22 |
| ENSG00000211680 | IGLJ4 | immunoglobulin lambda joining 4 (non-functional) | 22 | q11.22 |
| ENSG00000211681 | IGLJ5 | immunoglobulin lambda joining 5 (non-functional) | 22 | q11.22 |
| ENSG00000211682 | IGLJ6 | immunoglobulin lambda joining 6 | 22 | q11.22 |
| ENSG00000211684 | IGLJ7 | immunoglobulin lambda joining 7 | 22 | q11.22 |
| ENSG00000211685 | IGLC7 | immunoglobulin lambda constant 7 | 22 | q11.22 |
| ENSG00000211687 | TRGJ2 | T cell receptor gamma joining 2 | 7 | p14.1 |
| ENSG00000211688 | TRGJP2 | T cell receptor gamma joining P2 | 7 | p14.1 |
| ENSG00000211689 | TRGC1 | T cell receptor gamma constant 1 | 7 | p14.1 |
| ENSG00000211690 | TRGJ1 | T cell receptor gamma joining 1 | 7 | p14.1 |
| ENSG00000211691 | TRGJP | T cell receptor gamma joining P | 7 | p14.1 |
| ENSG00000211692 | TRGJP1 | T cell receptor gamma joining P1 | 7 | p14.1 |
| ENSG00000211693 | TRGV11 | T cell receptor gamma variable 11 (non-functional) | 7 | p14.1 |
| ENSG00000211694 | TRGV10 | T cell receptor gamma variable 10 (non-functional) | 7 | p14.1 |
| ENSG00000211695 | TRGV9 | T cell receptor gamma variable 9 | 7 | p14.1 |
| ENSG00000211696 | TRGV8 | T cell receptor gamma variable 8 | 7 | p14.1 |
| ENSG00000211697 | TRGV5 | T cell receptor gamma variable 5 | 7 | p14.1 |
| ENSG00000211698 | TRGV4 | T cell receptor gamma variable 4 | 7 | p14.1 |
| ENSG00000211699 | TRGV3 | T cell receptor gamma variable 3 | 7 | p14.1 |
| ENSG00000211701 | TRGV1 | T cell receptor gamma variable 1 (non-functional) | 7 | p14.1 |
| ENSG00000211706 | TRBV6-1 | T cell receptor beta variable 6-1 | 7 | q34 |
| ENSG00000211707 | TRBV7-1 | T cell receptor beta variable 7-1 (non-functional) | 7 | q34 |
| ENSG00000211710 | TRBV4-1 | T cell receptor beta variable 4-1 | 7 | q34 |
| ENSG00000211713 | TRBV6-4 | T cell receptor beta variable 6-4 | 7 | q34 |
| ENSG00000211714 | TRBV7-3 | T cell receptor beta variable 7-3 | 7 | q34 |
| ENSG00000211715 | TRBV5-3 | T cell receptor beta variable 5-3 (non-functional) | 7 | q34 |
| ENSG00000211716 | TRBV9 | T cell receptor beta variable 9 | 7 | q34 |
| ENSG00000211717 | TRBV10-1 | T cell receptor beta variable 10-1(gene/pseudogene) | 7 | q34 |
| ENSG00000211720 | TRBV11-1 | T cell receptor beta variable 11-1 | 7 | q34 |
| ENSG00000211721 | TRBV6-5 | T cell receptor beta variable 6-5 | 7 | q34 |
| ENSG00000211724 | TRBV6-6 | T cell receptor beta variable 6-6 | 7 | q34 |
| ENSG00000211725 | TRBV5-5 | T cell receptor beta variable 5-5 | 7 | q34 |
| ENSG00000211727 | TRBV7-6 | T cell receptor beta variable 7-6 | 7 | q34 |
| ENSG00000211728 | TRBV5-6 | T cell receptor beta variable 5-6 | 7 | q34 |
| ENSG00000211731 | TRBV5-7 | T cell receptor beta variable 5-7 (non-functional) | 7 | q34 |
| ENSG00000211734 | TRBV5-1 | T cell receptor beta variable 5-1 | 7 | q34 |
| ENSG00000211739 | TRBV12-2 | T cell receptor beta variable 12-2 (pseudogene) | 7 | q34 |
| ENSG00000211745 | TRBV4-2 | T cell receptor beta variable 4-2 | 7 | q34 |
| ENSG00000211746 | TRBV19 | T cell receptor beta variable 19 | 7 | q34 |
| ENSG00000211747 | TRBV20-1 | T cell receptor beta variable 20-1 | 7 | q34 |
| ENSG00000211749 | TRBV23-1 | T cell receptor beta variable 23-1 (non-functional) | 7 | q34 |
| ENSG00000211750 | TRBV24-1 | T cell receptor beta variable 24-1 | 7 | q34 |
| ENSG00000211751 | TRBV25-1 | T cell receptor beta variable 25-1 | 7 | q34 |
| ENSG00000211752 | TRBV27 | T cell receptor beta variable 27 | 7 | q34 |
| ENSG00000211753 | TRBV28 | T cell receptor beta variable 28 | 7 | q34 |
| ENSG00000211764 | TRBJ2-1 | T cell receptor beta joining 2-1 | 7 | q34 |
| ENSG00000211765 | TRBJ2-2 | T cell receptor beta joining 2-2 | 7 | q34 |
| ENSG00000211766 | TRBJ2-2P | T cell receptor beta joining 2-2P (non-functional) | 7 | q34 |
| ENSG00000211767 | TRBJ2-3 | T cell receptor beta joining 2-3 | 7 | q34 |
| ENSG00000211768 | TRBJ2-4 | T cell receptor beta joining 2-4 | 7 | q34 |
| ENSG00000211769 | TRBJ2-5 | T cell receptor beta joining 2-5 | 7 | q34 |
| ENSG00000211770 | TRBJ2-6 | T cell receptor beta joining 2-6 | 7 | q34 |
| ENSG00000211771 | TRBJ2-7 | T cell receptor beta joining 2-7 | 7 | q34 |
| ENSG00000211772 | TRBC2 | T cell receptor beta constant 2 | 7 | q34 |
| ENSG00000211776 | TRAV2 | T cell receptor alpha variable 2 | 14 | q11.2 |
| ENSG00000211777 | TRAV3 | T cell receptor alpha variable 3 (gene/pseudogene) | 14 | q11.2 |
| ENSG00000211778 | TRAV4 | T cell receptor alpha variable 4 | 14 | q11.2 |
| ENSG00000211779 | TRAV5 | T cell receptor alpha variable 5 | 14 | q11.2 |
| ENSG00000211780 | TRAV6 | T cell receptor alpha variable 6 | 14 | q11.2 |
| ENSG00000211781 | TRAV7 | T cell receptor alpha variable 7 | 14 | q11.2 |
| ENSG00000211782 | TRAV8-1 | T cell receptor alpha variable 8-1 | 14 | q11.2 |
| ENSG00000211783 | TRAV9-1 | T cell receptor alpha variable 9-1 | 14 | q11.2 |
| ENSG00000211784 | TRAV10 | T cell receptor alpha variable 10 | 14 | q11.2 |
| ENSG00000211785 | TRAV12-1 | T cell receptor alpha variable 12-1 | 14 | q11.2 |
| ENSG00000211786 | TRAV8-2 | T cell receptor alpha variable 8-2 | 14 | q11.2 |
| ENSG00000211787 | TRAV8-3 | T cell receptor alpha variable 8-3 | 14 | q11.2 |
| ENSG00000211788 | TRAV13-1 | T cell receptor alpha variable 13-1 | 14 | q11.2 |
| ENSG00000211789 | TRAV12-2 | T cell receptor alpha variable 12-2 | 14 | q11.2 |
| ENSG00000211790 | TRAV8-4 | T cell receptor alpha variable 8-4 | 14 | q11.2 |
| ENSG00000211791 | TRAV13-2 | T cell receptor alpha variable 13-2 | 14 | q11.2 |
| ENSG00000211792 | TRAV14DV4 | T cell receptor alpha variable 14/delta variable 4 | 14 | q11.2 |
| ENSG00000211793 | TRAV9-2 | T cell receptor alpha variable 9-2 | 14 | q11.2 |
| ENSG00000211794 | TRAV12-3 | T cell receptor alpha variable 12-3 | 14 | q11.2 |
| ENSG00000211795 | TRAV8-6 | T cell receptor alpha variable 8-6 | 14 | q11.2 |
| ENSG00000211796 | TRAV16 | T cell receptor alpha variable 16 | 14 | q11.2 |
| ENSG00000211797 | TRAV17 | T cell receptor alpha variable 17 | 14 | q11.2 |
| ENSG00000211798 | TRAV18 | T cell receptor alpha variable 18 | 14 | q11.2 |
| ENSG00000211799 | TRAV19 | T cell receptor alpha variable 19 | 14 | q11.2 |
| ENSG00000211800 | TRAV20 | T cell receptor alpha variable 20 | 14 | q11.2 |
| ENSG00000211801 | TRAV21 | T cell receptor alpha variable 21 | 14 | q11.2 |
| ENSG00000211802 | TRAV22 | T cell receptor alpha variable 22 | 14 | q11.2 |
| ENSG00000211803 | TRAV23DV6 | T cell receptor alpha variable 23/delta variable 6 | 14 | q11.2 |
| ENSG00000211804 | TRDV1 | T cell receptor delta variable 1 | 14 | q11.2 |
| ENSG00000211805 | TRAV24 | T cell receptor alpha variable 24 | 14 | q11.2 |
| ENSG00000211806 | TRAV25 | T cell receptor alpha variable 25 | 14 | q11.2 |
| ENSG00000211807 | TRAV26-1 | T cell receptor alpha variable 26-1 | 14 | q11.2 |
| ENSG00000211808 | TRAV8-7 | T cell receptor alpha variable 8-7 (non-functional) | 14 | q11.2 |
| ENSG00000211809 | TRAV27 | T cell receptor alpha variable 27 | 14 | q11.2 |
| ENSG00000211810 | TRAV29DV5 | T cell receptor alpha variable 29/delta variable 5 (gene/pseudogene) | 14 | q11.2 |
| ENSG00000211812 | TRAV26-2 | T cell receptor alpha variable 26-2 | 14 | q11.2 |
| ENSG00000211813 | TRAV34 | T cell receptor alpha variable 34 | 14 | q11.2 |
| ENSG00000211814 | TRAV35 | T cell receptor alpha variable 35 | 14 | q11.2 |
| ENSG00000211815 | TRAV36DV7 | T cell receptor alpha variable 36/delta variable 7 | 14 | q11.2 |
| ENSG00000211816 | TRAV38-1 | T cell receptor alpha variable 38-1 | 14 | q11.2 |
| ENSG00000211817 | TRAV38-2DV8 | T cell receptor alpha variable 38-2/delta variable 8 | 14 | q11.2 |
| ENSG00000211818 | TRAV39 | T cell receptor alpha variable 39 | 14 | q11.2 |
| ENSG00000211819 | TRAV40 | T cell receptor alpha variable 40 | 14 | q11.2 |
| ENSG00000211820 | TRAV41 | T cell receptor alpha variable 41 | 14 | q11.2 |
| ENSG00000211821 | TRDV2 | T cell receptor delta variable 2 | 14 | q11.2 |
| ENSG00000211825 | TRDJ1 | T cell receptor delta joining 1 | 14 | q11.2 |
| ENSG00000211826 | TRDJ4 | T cell receptor delta joining 4 | 14 | q11.2 |
| ENSG00000211827 | TRDJ2 | T cell receptor delta joining 2 | 14 | q11.2 |
| ENSG00000211828 | TRDJ3 | T cell receptor delta joining 3 | 14 | q11.2 |
| ENSG00000211829 | TRDC | T cell receptor delta constant | 14 | q11.2 |
| ENSG00000211831 | TRAJ61 | T cell receptor alpha joining 61 (non-functional) | 14 | q11.2 |
| ENSG00000211832 | TRAJ59 | T cell receptor alpha joining 59 (non-functional) | 14 | q11.2 |
| ENSG00000211833 | TRAJ58 | T cell receptor alpha joining 58 (non-functional) | 14 | q11.2 |
| ENSG00000211834 | TRAJ57 | T cell receptor alpha joining 57 | 14 | q11.2 |
| ENSG00000211835 | TRAJ56 | T cell receptor alpha joining 56 | 14 | q11.2 |
| ENSG00000211836 | TRAJ54 | T cell receptor alpha joining 54 | 14 | q11.2 |
| ENSG00000211837 | TRAJ53 | T cell receptor alpha joining 53 | 14 | q11.2 |
| ENSG00000211838 | TRAJ52 | T cell receptor alpha joining 52 | 14 | q11.2 |
| ENSG00000211839 | TRAJ50 | T cell receptor alpha joining 50 | 14 | q11.2 |
| ENSG00000211840 | TRAJ49 | T cell receptor alpha joining 49 | 14 | q11.2 |
| ENSG00000211841 | TRAJ48 | T cell receptor alpha joining 48 | 14 | q11.2 |
| ENSG00000211842 | TRAJ47 | T cell receptor alpha joining 47 | 14 | q11.2 |
| ENSG00000211843 | TRAJ46 | T cell receptor alpha joining 46 | 14 | q11.2 |
| ENSG00000211844 | TRAJ45 | T cell receptor alpha joining 45 | 14 | q11.2 |
| ENSG00000211845 | TRAJ44 | T cell receptor alpha joining 44 | 14 | q11.2 |
| ENSG00000211846 | TRAJ43 | T cell receptor alpha joining 43 | 14 | q11.2 |
| ENSG00000211847 | TRAJ42 | T cell receptor alpha joining 42 | 14 | q11.2 |
| ENSG00000211848 | TRAJ41 | T cell receptor alpha joining 41 | 14 | q11.2 |
| ENSG00000211849 | TRAJ40 | T cell receptor alpha joining 40 | 14 | q11.2 |
| ENSG00000211850 | TRAJ39 | T cell receptor alpha joining 39 | 14 | q11.2 |
| ENSG00000211851 | TRAJ38 | T cell receptor alpha joining 38 | 14 | q11.2 |
| ENSG00000211854 | TRAJ35 | T cell receptor alpha joining 35 (non-functional) | 14 | q11.2 |
| ENSG00000211855 | TRAJ34 | T cell receptor alpha joining 34 | 14 | q11.2 |
| ENSG00000211856 | TRAJ33 | T cell receptor alpha joining 33 | 14 | q11.2 |
| ENSG00000211857 | TRAJ32 | T cell receptor alpha joining 32 | 14 | q11.2 |
| ENSG00000211858 | TRAJ31 | T cell receptor alpha joining 31 | 14 | q11.2 |
| ENSG00000211859 | TRAJ30 | T cell receptor alpha joining 30 | 14 | q11.2 |
| ENSG00000211860 | TRAJ29 | T cell receptor alpha joining 29 | 14 | q11.2 |
| ENSG00000211861 | TRAJ28 | T cell receptor alpha joining 28 | 14 | q11.2 |
| ENSG00000211862 | TRAJ27 | T cell receptor alpha joining 27 | 14 | q11.2 |
| ENSG00000211863 | TRAJ26 | T cell receptor alpha joining 26 | 14 | q11.2 |
| ENSG00000211864 | TRAJ25 | T cell receptor alpha joining 25 (non-functional) | 14 | q11.2 |
| ENSG00000211865 | TRAJ24 | T cell receptor alpha joining 24 | 14 | q11.2 |
| ENSG00000211866 | TRAJ23 | T cell receptor alpha joining 23 | 14 | q11.2 |
| ENSG00000211867 | TRAJ22 | T cell receptor alpha joining 22 | 14 | q11.2 |
| ENSG00000211868 | TRAJ21 | T cell receptor alpha joining 21 | 14 | q11.2 |
| ENSG00000211869 | TRAJ20 | T cell receptor alpha joining 20 | 14 | q11.2 |
| ENSG00000211870 | TRAJ19 | T cell receptor alpha joining 19 (non-functional) | 14 | q11.2 |
| ENSG00000211871 | TRAJ18 | T cell receptor alpha joining 18 | 14 | q11.2 |
| ENSG00000211872 | TRAJ17 | T cell receptor alpha joining 17 | 14 | q11.2 |
| ENSG00000211873 | TRAJ16 | T cell receptor alpha joining 16 | 14 | q11.2 |
| ENSG00000211875 | TRAJ14 | T cell receptor alpha joining 14 | 14 | q11.2 |
| ENSG00000211876 | TRAJ13 | T cell receptor alpha joining 13 | 14 | q11.2 |
| ENSG00000211877 | TRAJ12 | T cell receptor alpha joining 12 | 14 | q11.2 |
| ENSG00000211878 | TRAJ11 | T cell receptor alpha joining 11 | 14 | q11.2 |
| ENSG00000211879 | TRAJ10 | T cell receptor alpha joining 10 | 14 | q11.2 |
| ENSG00000211880 | TRAJ9 | T cell receptor alpha joining 9 | 14 | q11.2 |
| ENSG00000211881 | TRAJ8 | T cell receptor alpha joining 8 | 14 | q11.2 |
| ENSG00000211882 | TRAJ7 | T cell receptor alpha joining 7 | 14 | q11.2 |
| ENSG00000211883 | TRAJ6 | T cell receptor alpha joining 6 | 14 | q11.2 |
| ENSG00000211884 | TRAJ5 | T cell receptor alpha joining 5 | 14 | q11.2 |
| ENSG00000211885 | TRAJ4 | T cell receptor alpha joining 4 | 14 | q11.2 |
| ENSG00000211886 | TRAJ3 | T cell receptor alpha joining 3 | 14 | q11.2 |
| ENSG00000211887 | TRAJ2 | T cell receptor alpha joining 2 (non-functional) | 14 | q11.2 |
| ENSG00000211888 | TRAJ1 | T cell receptor alpha joining 1 (non-functional) | 14 | q11.2 |
| ENSG00000211890 | IGHA2 | immunoglobulin heavy constant alpha 2 (A2m marker) | 14 | q32.33 |
| ENSG00000211891 | IGHE | immunoglobulin heavy constant epsilon | 14 | q32.33 |
| ENSG00000211892 | IGHG4 | immunoglobulin heavy constant gamma 4 (G4m marker) | 14 | q32.33 |
| ENSG00000211893 | IGHG2 | immunoglobulin heavy constant gamma 2 (G2m marker) | 14 | q32.33 |
| ENSG00000211895 | IGHA1 | immunoglobulin heavy constant alpha 1 | 14 | q32.33 |
| ENSG00000211896 | IGHG1 | immunoglobulin heavy constant gamma 1 (G1m marker) | 14 | q32.33 |
| ENSG00000211897 | IGHG3 | immunoglobulin heavy constant gamma 3 (G3m marker) | 14 | q32.33 |
| ENSG00000211898 | IGHD | immunoglobulin heavy constant delta | 14 | q32.33 |
| ENSG00000211899 | IGHM | immunoglobulin heavy constant mu | 14 | q32.33 |
| ENSG00000211900 | IGHJ6 | immunoglobulin heavy joining 6 | 14 | q32.33 |
| ENSG00000211904 | IGHJ2 | immunoglobulin heavy joining 2 | 14 | q32.33 |
| ENSG00000211905 | IGHJ1 | immunoglobulin heavy joining 1 | 14 | q32.33 |
| ENSG00000211907 | IGHD1-26 | immunoglobulin heavy diversity 1-26 | 14 | q32.33 |
| ENSG00000211909 | IGHD5-24 | immunoglobulin heavy diversity 5-24 (non-functional) | 14 | q32.33 |
| ENSG00000211911 | IGHD3-22 | immunoglobulin heavy diversity 3-22 | 14 | q32.33 |
| ENSG00000211912 | IGHD2-21 | immunoglobulin heavy diversity 2-21 | 14 | q32.33 |
| ENSG00000211914 | IGHD6-19 | immunoglobulin heavy diversity 6-19 | 14 | q32.33 |
| ENSG00000211915 | IGHD5-18 | immunoglobulin heavy diversity 5-18 | 14 | q32.33 |
| ENSG00000211917 | IGHD3-16 | immunoglobulin heavy diversity 3-16 | 14 | q32.33 |
| ENSG00000211918 | IGHD2-15 | immunoglobulin heavy diversity 2-15 | 14 | q32.33 |
| ENSG00000211920 | IGHD6-13 | immunoglobulin heavy diversity 6-13 | 14 | q32.33 |
| ENSG00000211921 | IGHD5-12 | immunoglobulin heavy diversity 5-12 | 14 | q32.33 |
| ENSG00000211923 | IGHD3-10 | immunoglobulin heavy diversity 3-10 | 14 | q32.33 |
| ENSG00000211924 | IGHD3-9 | immunoglobulin heavy diversity 3-9 | 14 | q32.33 |
| ENSG00000211925 | IGHD2-8 | immunoglobulin heavy diversity 2-8 | 14 | q32.33 |
| ENSG00000211928 | IGHD5-5 | immunoglobulin heavy diversity 5-5 | 14 | q32.33 |
| ENSG00000211930 | IGHD3-3 | immunoglobulin heavy diversity 3-3 | 14 | q32.33 |
| ENSG00000211931 | IGHD2-2 | immunoglobulin heavy diversity 2-2 | 14 | q32.33 |
| ENSG00000211933 | IGHV6-1 | immunoglobulin heavy variable 6-1 | 14 | q32.33 |
| ENSG00000211934 | IGHV1-2 | immunoglobulin heavy variable 1-2 | 14 | q32.33 |
| ENSG00000211935 | IGHV1-3 | immunoglobulin heavy variable 1-3 | 14 | q32.33 |
| ENSG00000211937 | IGHV2-5 | immunoglobulin heavy variable 2-5 | 14 | q32.33 |
| ENSG00000211938 | IGHV3-7 | immunoglobulin heavy variable 3-7 | 14 | q32.33 |
| ENSG00000211941 | IGHV3-11 | immunoglobulin heavy variable 3-11 (gene/pseudogene) | 14 | q32.33 |
| ENSG00000211942 | IGHV3-13 | immunoglobulin heavy variable 3-13 | 14 | q32.33 |
| ENSG00000211943 | IGHV3-15 | immunoglobulin heavy variable 3-15 | 14 | q32.33 |
| ENSG00000211944 | IGHV3-16 | immunoglobulin heavy variable 3-16 (non-functional) | 14 | q32.33 |
| ENSG00000211945 | IGHV1-18 | immunoglobulin heavy variable 1-18 | 14 | q32.33 |
| ENSG00000211946 | IGHV3-20 | immunoglobulin heavy variable 3-20 | 14 | q32.33 |
| ENSG00000211947 | IGHV3-21 | immunoglobulin heavy variable 3-21 | 14 | q32.33 |
| ENSG00000211949 | IGHV3-23 | immunoglobulin heavy variable 3-23 | 14 | q32.33 |
| ENSG00000211950 | IGHV1-24 | immunoglobulin heavy variable 1-24 | 14 | q32.33 |
| ENSG00000211951 | IGHV2-26 | immunoglobulin heavy variable 2-26 | 14 | q32.33 |
| ENSG00000211952 | IGHV4-28 | immunoglobulin heavy variable 4-28 | 14 | q32.33 |
| ENSG00000211955 | IGHV3-33 | immunoglobulin heavy variable 3-33 | 14 | q32.33 |
| ENSG00000211956 | IGHV4-34 | immunoglobulin heavy variable 4-34 | 14 | q32.33 |
| ENSG00000211957 | IGHV3-35 | immunoglobulin heavy variable 3-35 (non-functional) | 14 | q32.33 |
| ENSG00000211958 | IGHV3-38 | immunoglobulin heavy variable 3-38 (non-functional) | 14 | q32.33 |
| ENSG00000211959 | IGHV4-39 | immunoglobulin heavy variable 4-39 | 14 | q32.33 |
| ENSG00000211961 | IGHV1-45 | immunoglobulin heavy variable 1-45 | 14 | q32.33 |
| ENSG00000211962 | IGHV1-46 | immunoglobulin heavy variable 1-46 | 14 | q32.33 |
| ENSG00000211964 | IGHV3-48 | immunoglobulin heavy variable 3-48 | 14 | q32.33 |
| ENSG00000211965 | IGHV3-49 | immunoglobulin heavy variable 3-49 | 14 | q32.33 |
| ENSG00000211966 | IGHV5-51 | immunoglobulin heavy variable 5-51 | 14 | q32.33 |
| ENSG00000211967 | IGHV3-53 | immunoglobulin heavy variable 3-53 | 14 | q32.33 |
| ENSG00000211968 | IGHV1-58 | immunoglobulin heavy variable 1-58 | 14 | q32.33 |
| ENSG00000211970 | IGHV4-61 | immunoglobulin heavy variable 4-61 | 14 | q32.33 |
| ENSG00000211972 | IGHV3-66 | immunoglobulin heavy variable 3-66 | 14 | q32.33 |
| ENSG00000211973 | IGHV1-69 | immunoglobulin heavy variable 1-69 | 14 | q32.33 |
| ENSG00000211974 | IGHV2-70 | immunoglobulin heavy variable 2-70 | 14 | q32.33 |
| ENSG00000211976 | IGHV3-73 | immunoglobulin heavy variable 3-73 | 14 | q32.33 |
| ENSG00000211978 | IGHV5-78 | immunoglobulin heavy variable 5-78 (pseudogene) | 14 | q32.33 |
| ENSG00000211979 | IGHV7-81 | immunoglobulin heavy variable 7-81 (non-functional) | 14 | q32.33 |
| ENSG00000166278 | C2 | complement component 2 | 6 | p21.33 |
| ENSG00000206344 | HCG27 | HLA complex group 27 (non-protein coding) | 6 | p21.33 |
| ENSG00000204463 | BAG6 | BCL2-associated athanogene 6 | 6 | p21.33 |
| ENSG00000204592 | HLA-E | major histocompatibility complex, class I, E | 6 | p22.1 |
| ENSG00000085231 | TAF9 | TAF9 RNA polymerase II, TATA box binding protein (TBP)-associated factor, 32kDa | 5 | q13.2 |
| ENSG00000244731 | C4A | complement component 4A (Rodgers blood group) | 6 | p21.33 |
| ENSG00000206337 | HCP5 | HLA complex P5 (non-protein coding) | 6 | p21.33 |
| ENSG00000206341 | HLA-H | major histocompatibility complex, class I, H (pseudogene) | 6 | p22.1 |
| ENSG00000215788 | TNFRSF25 | tumor necrosis factor receptor superfamily, member 25 | 1 | p36.31 |
| ENSG00000215765 | KIR3DP1 | killer cell immunoglobulin-like receptor, three domains, pseudogene 1 | 19 | q13.42 |
| ENSG00000215644 | GCGR | glucagon receptor | 17 | q25.3 |
| ENSG00000215568 | GAB4 | GRB2-associated binding protein family, member 4 | 22 | q11.1 |
| ENSG00000215547 | DEFB115 | defensin, beta 115 | 20 | q11.21 |
| ENSG00000215545 | DEFB116 | defensin, beta 116 | 20 | q11.21 |
| ENSG00000215515 | IFIT1P1 | interferon-induced protein with tetratricopeptide repeats 1 pseudogene 1 | 13 | q13.1 |
| ENSG00000215483 | LINC00598 | long intergenic non-protein coding RNA 598 | 13 | q14.11 |
| ENSG00000215474 | SKOR2 | SKI family transcriptional corepressor 2 | 18 | q21.1 |
| ENSG00000215414 | PSMA6P1 | proteasome (prosome, macropain) subunit, alpha type, 6 pseudogene 1 | Y | q11.221 |
| ENSG00000215378 | DEFT1P | defensin, theta 1 pseudogene | 8 | p23.1 |
| ENSG00000215371 | DEFB108P2 | defensin, beta 108, pseudogene 2 | 8 | p23.1 |
| ENSG00000215353 | C1QBPP | complement component 1, q subcomponent binding protein, pseudogene | 21 | q21.1 |
| ENSG00000215251 | FASTKD5 | FAST kinase domains 5 | 20 | p13 |
| ENSG00000215206 | TRBV24OR9-2 | T cell receptor beta variable 24/OR9-2 (pseudogene) | 9 | p13.3 |
| ENSG00000215182 | MUC5AC | mucin 5AC, oligomeric mucus/gel-forming | 11 | p15.5 |
| ENSG00000215177 | IGLV8OR8-1 | immunoglobulin lambda variable 8/OR8-1 (pseudogene) | 8 | q11.21 |
| ENSG00000215096 | IFITM8P | interferon induced transmembrane protein 8 pseudogene | 8 | q12.3 |
| ENSG00000223865 | HLA-DPB1 | major histocompatibility complex, class II, DP beta 1 | 6 | p21.32 |
| ENSG00000215018 | COL28A1 | collagen, type XXVIII, alpha 1 | 7 | p21.3 |
| ENSG00000214897 | MOAP1 | modulator of apoptosis 1 | X | q28 |
| ENSG00000214827 | MTCP1 | mature T-cell proliferation 1 | X | q28 |
| ENSG00000214756 | METTL12 | methyltransferase like 12 | 11 | q12.3 |
| ENSG00000214706 | IFRD2 | interferon-related developmental regulator 2 | 3 | p21.31 |
| ENSG00000214643 | DEFB133 | defensin, beta 133 | 6 | p12.3 |
| ENSG00000214642 | DEFB113 | defensin, beta 113 | 6 | p12.3 |
| ENSG00000214253 | FIS1 | fission 1 (mitochondrial outer membrane) homolog (S. cerevisiae) | 7 | q22.1 |
| ENSG00000214042 | IFNA7 | interferon, alpha 7 | 9 | p21.3 |
| ENSG00000213977 | TAX1BP3 | Tax1 (human T-cell leukemia virus type I) binding protein 3 | 17 | p13.2 |
| ENSG00000213949 | ITGA1 | integrin, alpha 1 | 5 | q11.2 |
| ENSG00000213937 | CLDN9 | claudin 9 | 16 | p13.3 |
| ENSG00000213928 | IRF9 | interferon regulatory factor 9 | 14 | q12 |
| ENSG00000213927 | CCL27 | chemokine (C-C motif) ligand 27 | 9 | p13.3 |
| ENSG00000213918 | DNASE1 | deoxyribonuclease I | 16 | p13.3 |
| ENSG00000213906 | LTB4R2 | leukotriene B4 receptor 2 | 14 | q12 |
| ENSG00000213892 | CEACAM16 | carcinoembryonic antigen-related cell adhesion molecule 16 | 19 | q13.31 |
| ENSG00000213886 | UBD | ubiquitin D | 6 | p22.1 |
| ENSG00000213822 | CEACAM18 | carcinoembryonic antigen-related cell adhesion molecule 18 | 19 | q13.41 |
| ENSG00000213809 | KLRK1 | killer cell lectin-like receptor subfamily K, member 1 | 12 | p13.2 |
| ENSG00000213722 | DDAH2 | dimethylarginine dimethylaminohydrolase 2 | 6 | p21.33 |
| ENSG00000213694 | S1PR3 | sphingosine-1-phosphate receptor 3 | 9 | q22.1 |
| ENSG00000213658 | LAT | linker for activation of T cells | 16 | p11.2 |
| ENSG00000213638 | ADAT3 | adenosine deaminase, tRNA-specific 3 | 19 | p13.3 |
| ENSG00000213619 | NDUFS3 | NADH dehydrogenase (ubiquinone) Fe-S protein 3, 30kDa (NADH-coenzyme Q reductase) | 11 | p11.2 |
| ENSG00000213585 | VDAC1 | voltage-dependent anion channel 1 | 5 | q31.1 |
| ENSG00000213578 | CPLX3 | complexin 3 | 15 | q24.1 |
| ENSG00000213523 | SRA1 | steroid receptor RNA activator 1 | 5 | q31.3 |
| ENSG00000213445 | SIPA1 | signal-induced proliferation-associated 1 | 11 | q13.1 |
| ENSG00000213413 | PVRIG | poliovirus receptor related immunoglobulin domain containing | 7 | q22.1 |
| ENSG00000213341 | CHUK | conserved helix-loop-helix ubiquitous kinase | 10 | q24.31 |
| ENSG00000213316 | LTC4S | leukotriene C4 synthase | 5 | q35.3 |
| ENSG00000213275 | IFITM9P | interferon induced transmembrane protein 9 pseudogene | 11 | q13.3 |
| ENSG00000213231 | TCL1B | T-cell leukemia/lymphoma 1B | 14 | q32.13 |
| ENSG00000213190 | MLLT11 | myeloid/lymphoid or mixed-lineage leukemia (trithorax homolog, Drosophila); translocated to, 11 | 1 | q21.3 |
| ENSG00000213088 | DARC | Duffy blood group, chemokine receptor | 1 | q23.2 |
| ENSG00000213024 | NUP62 | nucleoporin 62kDa | 19 | q13.33 |
| ENSG00000213016 | KIR3DL2 | killer cell immunoglobulin-like receptor, three domains, long cytoplasmic tail, 2 | 19 | q13.42 |
| ENSG00000212717 | DEFB117 | defensin, beta 117 | 20 | q11.21 |
| ENSG00000212710 | CTAGE1 | cutaneous T-cell lymphoma-associated antigen 1 | 18 | q11.2 |
| ENSG00000217159 | LARP1P1 | La ribonucleoprotein domain family, member 1 pseudogene 1 | 6 | p22.2 |
| ENSG00000216777 | PRRC2CP1 | proline-rich coiled-coil 2C pseudogene 1 | Y | p11.2 |
| ENSG00000221801 | MIR548H2 | microRNA 548h-2 | 16 | p13.13 |
| ENSG00000222028 | PSMB11 | proteasome (prosome, macropain) subunit, beta type, 11 | 14 | q11.2 |
| ENSG00000222037 | IGLC6 | immunoglobulin lambda constant 6 (Kern+Oz- marker, gene/pseudogene) | 22 | q11.22 |
| ENSG00000221818 | EBF2 | early B-cell factor 2 | 8 | p21.2 |
| ENSG00000221829 | FANCG | Fanconi anemia, complementation group G | 9 | p13.3 |
| ENSG00000221887 | HMSD | histocompatibility (minor) serpin domain containing | 18 | q22.1 |
| ENSG00000221926 | TRIM16 | tripartite motif containing 16 | 17 | p12 |
| ENSG00000221947 | XKR9 | XK, Kell blood group complex subunit-related family, member 9 | 8 | q13.3 |
| ENSG00000221995 | TIAF1 | TGFB1-induced anti-apoptotic factor 1 | 17 | q11.2 |
| ENSG00000219438 | FAM19A5 | family with sequence similarity 19 (chemokine (C-C motif)-like), member A5 | 22 | q13.32 |
| ENSG00000216490 | IFI30 | interferon, gamma-inducible protein 30 | 19 | p13.11 |
| ENSG00000217555 | CKLF | chemokine-like factor | 16 | q21 |
| ENSG00000221957 | KIR2DS4 | killer cell immunoglobulin-like receptor, two domains, short cytoplasmic tail, 4 | 19 | q13.42 |
| ENSG00000222043 | NFE2L2 | nuclear factor (erythroid-derived 2)-like 2 | 2 | q31.2 |
| ENSG00000223629 | DEFA8P | defensin, alpha 8 pseudogene | 8 | p23.1 |
| ENSG00000233238 | DEFA9P | defensin, alpha 9 pseudogene | 8 | p23.1 |
| ENSG00000233531 | DEFA10P | defensin, alpha 10 pseudogene | 8 | p23.1 |
| ENSG00000232039 | DEFT1P2 | defensin, theta 1 pseudogene 2 | 8 | p23.1 |
| ENSG00000234178 | DEFA11P | defensin, alpha 11 pseudogene | 8 | p23.1 |
| ENSG00000230549 | USP17L1P | ubiquitin specific peptidase 17-like family member 1, pseudogene | 8 | p23.1 |
| ENSG00000229907 | DEFB108P1 | defensin, beta 108, pseudogene 1 | 8 | p23.1 |
| ENSG00000225327 | USP17L3 | ubiquitin specific peptidase 17-like family member 3 | 8 | p23.1 |
| ENSG00000235947 | EGOT | eosinophil granule ontogeny transcript (non-protein coding) | 3 | p26.1 |
| ENSG00000226942 | IL9RP3 | interleukin 9 receptor pseudogene 3 | 16 | p13.3 |
| ENSG00000233050 | DEFB130 | beta-defensin 130-like precursor | 8 | p23.1 |
| ENSG00000223773 | CD99P1 | CD99 molecule pseudogene 1 | X | p22.33 |
| ENSG00000223443 | USP17L2 | ubiquitin specific peptidase 17-like family member 2 | 8 | p23.1 |
| ENSG00000232948 | DEFB130 | defensin, beta 130 | 8 | p23.1 |
| ENSG00000237517 | DGCR5 | DiGeorge syndrome critical region gene 5 (non-protein coding) | 22 | q11.21 |
| ENSG00000232420 | IL9RP2 | interleukin 9 receptor pseudogene 2 | 10 | p15.3 |
| ENSG00000238067 | XKRYP1 | XK, Kell blood group complex subunit-related, Y-linked pseudogene 1 | Y | q11.222 |
| ENSG00000230904 | XKRYP2 | XK, Kell blood group complex subunit-related, Y-linked pseudogene 2 | Y | q11.222 |
| ENSG00000227140 | USP17L5 | ubiquitin specific peptidase 17-like family member 5 | 4 | p16.1 |
| ENSG00000204632 | HLA-G | major histocompatibility complex, class I, G | 6 | p22.1 |
| ENSG00000206503 | HLA-A | major histocompatibility complex, class I, A | 6 | p22.1 |
| ENSG00000236620 | XKRYP3 | XK, Kell blood group complex subunit-related, Y-linked pseudogene 3 | Y | q11.223 |
| ENSG00000231026 | XKRYP4 | XK, Kell blood group complex subunit-related, Y-linked pseudogene 4 | Y | q11.23 |
| ENSG00000223406 | XKRYP5 | XK, Kell blood group complex subunit-related, Y-linked pseudogene 5 | Y | q11.23 |
| ENSG00000237546 | XKRYP6 | XK, Kell blood group complex subunit-related, Y-linked pseudogene 6 | Y | q11.23 |
| ENSG00000223741 | PSMD4P1 | proteasome (prosome, macropain) 26S subunit, non-ATPase, 4 pseudogene 1 | 21 | q22.13 |
| ENSG00000230208 | IFNNP1 | interferon, nu 1, pseudogene | 9 | p21.3 |
| ENSG00000232281 | IFNWP15 | interferon, omega 1 pseudogene 15 | 9 | p21.3 |
| ENSG00000236637 | IFNA4 | interferon, alpha 4 | 9 | p21.3 |
| ENSG00000226597 | IFNWP9 | interferon, omega 1 pseudogene 9 | 9 | p21.3 |
| ENSG00000223684 | IFNWP18 | interferon, omega 1 pseudogene 18 | 9 | p21.3 |
| ENSG00000234829 | IFNA17 | interferon, alpha 17 | 9 | p21.3 |
| ENSG00000232138 | IFNWP5 | interferon, omega 1 pseudogene 5 | 9 | p21.3 |
| ENSG00000228083 | IFNA14 | interferon, alpha 14 | 9 | p21.3 |
| ENSG00000224416 | IFNA22P | interferon, alpha 22, pseudogene | 9 | p21.3 |
| ENSG00000233816 | IFNA13 | interferon, alpha 13 | 9 | p21.3 |
| ENSG00000237691 | IFNWP2 | interferon, omega 1 pseudogene 2 | 9 | p21.3 |
| ENSG00000238271 | IFNWP19 | interferon, omega 1 pseudogene 19 | 9 | p21.3 |
| ENSG00000234745 | HLA-B | major histocompatibility complex, class I, B | 6 | p21.33 |
| ENSG00000224465 | SOCS2P2 | suppressor of cytokine signaling 2 pseudogene 2 | 22 | q11.22 |
| ENSG00000223350 | IGLV9-49 | immunoglobulin lambda variable 9-49 | 22 | q11.22 |
| ENSG00000226420 | IGLV3-4 | immunoglobulin lambda variable 3-4 (pseudogene) | 22 | q11.22 |
| ENSG00000228223 | HCG11 | HLA complex group 11 (non-protein coding) | 6 | p22.2 |
| ENSG00000204469 | PRRC2A | proline-rich coiled-coil 2A | 6 | p21.33 |
| ENSG00000232810 | TNF | tumor necrosis factor | 6 | p21.33 |
| ENSG00000234122 | TRBV22OR9-2 | T cell receptor beta variable 22/OR9-2 (pseudogene) | 9 | p13.3 |
| ENSG00000229063 | TRBV23OR9-2 | T cell receptor beta variable 23/OR9-2 (non-functional) | 9 | p13.3 |
| ENSG00000231165 | TRBV26OR9-2 | T cell receptor beta variable 26/OR9-2 (pseudogene) | 9 | p13.3 |
| ENSG00000224157 | HCG14 | HLA complex group 14 (non-protein coding) | 6 | p22.1 |
| ENSG00000223394 | TRBV29OR9-2 | T cell receptor beta variable 29/OR9-2 (non-functional) | 9 | p13.3 |
| ENSG00000227214 | HCG15 | HLA complex group 15 (non-protein coding) | 6 | p22.1 |
| ENSG00000234187 | AIMP1P1 | aminoacyl tRNA synthetase complex-interacting multifunctional protein 1 pseudogene 1 | 20 | p12.1 |
| ENSG00000230243 | FKBP1AP3 | FK506 binding protein 1A, 12kDa pseudogene 3 | 13 | q14.13 |
| ENSG00000225864 | HCG4P11 | HLA complex group 4 pseudogene 11 | 6 | p22.1 |
| ENSG00000227758 | HCG9P5 | HLA complex group 9 pseudogene 5 | 6 | p22.1 |
| ENSG00000235821 | IFITM4P | interferon induced transmembrane protein 4 pseudogene | 6 | p22.1 |
| ENSG00000237042 | MICG | MHC class I polypeptide-related sequence G (pseudogene) | 6 | p22.1 |
| ENSG00000229142 | HCG4P8 | HLA complex group 4 pseudogene 8 | 6 | p22.1 |
| ENSG00000233265 | MICF | MHC class I polypeptide-related sequence F (pseudogene) | 6 | p22.1 |
| ENSG00000230521 | HCG4P7 | HLA complex group 4 pseudogene 7 | 6 | p22.1 |
| ENSG00000231130 | HLA-T | major histocompatibility complex, class I, T (pseudogene) | 6 | p22.1 |
| ENSG00000227262 | HCG4P6 | HLA complex group 4 pseudogene 6 | 6 | p22.1 |
| ENSG00000230795 | HLA-K | major histocompatibility complex, class I, K (pseudogene) | 6 | p22.1 |
| ENSG00000228078 | HLA-U | major histocompatibility complex, class I, U (pseudogene) | 6 | p22.1 |
| ENSG00000225131 | PSME2P2 | proteasome activator subunit 2 pseudogene 2 | 13 | q14.2 |
| ENSG00000227766 | HCG4P5 | HLA complex group 4 pseudogene 5 | 6 | p22.1 |
| ENSG00000235290 | HLA-W | major histocompatibility complex, class I, W (pseudogene) | 6 | p22.1 |
| ENSG00000229390 | MICD | MHC class I polypeptide-related sequence D (pseudogene) | 6 | p22.1 |
| ENSG00000238024 | DDX39BP2 | DEAD (Asp-Glu-Ala-Asp) box polypeptide 39B pseudogene 2 | 6 | p22.1 |
| ENSG00000237669 | HCG4P3 | HLA complex group 4 pseudogene 3 | 6 | p22.1 |
| ENSG00000231074 | HCG18 | HLA complex group 18 (non-protein coding) | 6 | p22.1 |
| ENSG00000231607 | DLEU2 | deleted in lymphocytic leukemia 2 (non-protein coding) | 13 | q14.2 |
| ENSG00000224372 | HLA-N | major histocompatibility complex, class I, N (pseudogene) | 6 | p22.1 |
| ENSG00000224486 | HCG19P | HLA complex group 19 pseudogene | 6 | p22.1 |
| ENSG00000226577 | MICC | MHC class I polypeptide-related sequence C (pseudogene) | 6 | p22.1 |
| ENSG00000224389 | C4B | complement component 4B (Chido blood group) | 6 | p21.33 |
| ENSG00000233429 | HOTAIRM1 | HOXA transcript antisense RNA, myeloid-specific 1 | 7 | p15.2 |
| ENSG00000228022 | HCG20 | HLA complex group 20 (non-protein coding) | 6 | p21.33 |
| ENSG00000227018 | IL6STP1 | interleukin 6 signal transducer (gp130, oncostatin M receptor) pseudogene 1 | 17 | p12 |
| ENSG00000238151 | MLLT10P1 | myeloid/lymphoid or mixed-lineage leukemia (trithorax homolog, Drosophila); translocated to, 10 pseudogene 1 | 20 | q11.21 |
| ENSG00000233529 | HCG21 | HLA complex group 21 (non-protein coding) | 6 | p21.33 |
| ENSG00000228789 | HCG22 | HLA complex group 22 (non-protein coding) | 6 | p21.33 |
| ENSG00000232041 | PSMD10P3 | proteasome 26S subunit, non-ATPase, 10 pseudogene 3 | 13 | q22.1 |
| ENSG00000229749 | COTL1P1 | coactosin-like 1 (Dictyostelium) pseudogene 1 | 17 | p11.2 |
| ENSG00000225851 | HLA-S | major histocompatibility complex, class I, S (pseudogene) | 6 | p21.33 |
| ENSG00000226979 | LTA | lymphotoxin alpha (TNF superfamily, member 1) | 6 | p21.33 |
| ENSG00000227507 | LTB | lymphotoxin beta (TNF superfamily, member 3) | 6 | p21.33 |
| ENSG00000237662 | SOCS2P1 | suppressor of cytokine signaling 2 pseudogene 1 | 20 | q11.21 |
| ENSG00000227191 | TRGC2 | T cell receptor gamma constant 2 | 7 | p14.1 |
| ENSG00000231202 | TRGVB | T cell receptor gamma variable B (pseudogene) | 7 | p14.1 |
| ENSG00000225992 | TRGVA | T cell receptor gamma variable A (pseudogene) | 7 | p14.1 |
| ENSG00000226212 | TRGV6 | T cell receptor gamma variable 6 (pseudogene) | 7 | p14.1 |
| ENSG00000228668 | TRGV5P | T cell receptor gamma variable 5P (pseudogene) | 7 | p14.1 |
| ENSG00000233306 | TRGV2 | T cell receptor gamma variable 2 | 7 | p14.1 |
| ENSG00000235323 | COTL1P2 | coactosin-like 1 (Dictyostelium) pseudogene 2 | 17 | p11.2 |
| ENSG00000226742 | HSBP1L1 | heat shock factor binding protein 1-like 1 | 18 | q23 |
| ENSG00000228962 | HCG23 | HLA complex group 23 (non-protein coding) | 6 | p21.32 |
| ENSG00000229391 | HLA-DRB6 | major histocompatibility complex, class II, DR beta 6 (pseudogene) | 6 | p21.32 |
| ENSG00000226030 | HLA-DQB3 | major histocompatibility complex, class II, DQ beta 3 | 6 | p21.32 |
| ENSG00000237541 | HLA-DQA2 | major histocompatibility complex, class II, DQ alpha 2 | 6 | p21.32 |
| ENSG00000232629 | HLA-DQB2 | major histocompatibility complex, class II, DQ beta 2 | 6 | p21.32 |
| ENSG00000235301 | HLA-Z | major histocompatibility complex, class I, Z (pseudogene) | 6 | p21.32 |
| ENSG00000231461 | HLA-DPA2 | major histocompatibility complex, class II, DP alpha 2 (pseudogene) | 6 | p21.32 |
| ENSG00000224557 | HLA-DPB2 | major histocompatibility complex, class II, DP beta 2 (pseudogene) | 6 | p21.32 |
| ENSG00000237398 | HLA-DPA3 | major histocompatibility complex, class II, DP alpha 3 (pseudogene) | 6 | p21.32 |
| ENSG00000230313 | HCG24 | HLA complex group 24 (non-protein coding) | 6 | p21.32 |
| ENSG00000232254 | CSF2RBP1 | colony stimulating factor 2 receptor, beta, low-affinity (granulocyte-macrophage) pseudogene 1 | 22 | q12.3 |
| ENSG00000225830 | ERCC6 | excision repair cross-complementing rodent repair deficiency, complementation group 6 | 10 | q11.23 |
| ENSG00000232940 | HCG25 | HLA complex group 25 (non-protein coding) | 6 | p21.32 |
| ENSG00000231925 | TAPBP | TAP binding protein (tapasin) | 6 | p21.32 |
| ENSG00000236320 | SLFN14 | schlafen family member 14 | 17 | q12 |
| ENSG00000230011 | CTSL1P4 | cathepsin L1 pseudogene 4 | 10 | q11.23 |
| ENSG00000231043 | IK | IK cytokine, down-regulator of HLA II | 2 | p16.1 |
| ENSG00000233276 | GPX1 | glutathione peroxidase 1 | 3 | p21.31 |
| ENSG00000225690 | TREML5P | triggering receptor expressed on myeloid cells-like 5, pseudogene | 6 | p21.1 |
| ENSG00000229948 | B3GALNT1 |  | 2 | p13.3 |
| ENSG00000229314 | ORM1 | orosomucoid 1 | 9 | q32 |
| ENSG00000228278 | ORM2 | orosomucoid 2 | 9 | q32 |
| ENSG00000228663 | PSMD10P1 | proteasome 26S subunit, non-ATPase, 10 pseudogene 1 | 20 | q13.13 |
| ENSG00000235568 | NFAM1 | NFAT activating protein with ITAM motif 1 | 22 | q13.2 |
| ENSG00000237111 | IGHJ3P | immunoglobulin heavy joining 3P (pseudogene) | 14 | q32.33 |
| ENSG00000237547 | IGHJ2P | immunoglobulin heavy joining 2P (pseudogene) | 14 | q32.33 |
| ENSG00000227335 | IGHJ1P | immunoglobulin heavy joining 1P (pseudogene) | 14 | q32.33 |
| ENSG00000231475 | IGHV4-31 | immunoglobulin heavy variable 4-31 | 14 | q32.33 |
| ENSG00000232216 | IGHV3-43 | immunoglobulin heavy variable 3-43 | 14 | q32.33 |
| ENSG00000229092 | IGHV3-47 | immunoglobulin heavy variable 3-47 (pseudogene) | 14 | q32.33 |
| ENSG00000224373 | IGHV4-59 | immunoglobulin heavy variable 4-59 | 14 | q32.33 |
| ENSG00000223648 | IGHV3-64 | immunoglobulin heavy variable 3-64 | 14 | q32.33 |
| ENSG00000225698 | IGHV3-72 | immunoglobulin heavy variable 3-72 | 14 | q32.33 |
| ENSG00000224650 | IGHV3-74 | immunoglobulin heavy variable 3-74 | 14 | q32.33 |
| ENSG00000225366 | TDGF1P3 | teratocarcinoma-derived growth factor 1 pseudogene 3 | X | q23 |
| ENSG00000234512 | TLR12P | toll-like receptor 12, pseudogene | 1 | p35.1 |
| ENSG00000229204 | PTGES3P3 | prostaglandin E synthase 3 (cytosolic) pseudogene 3 | 4 | q33 |
| ENSG00000232119 | MCTS1 | malignant T cell amplified sequence 1 | X | q24 |
| ENSG00000225805 | LOC100129216 | Putative uncharacterized protein ENSP00000406391 | 11 | q13.4 |
| ENSG00000237693 | IRGM | immunity-related GTPase family, M | 5 | q33.1 |
| ENSG00000230558 | CEACAMP2 | carcinoembryonic antigen-related cell adhesion molecule pseudogene 2 | 19 | q13.2 |
| ENSG00000233681 | CEACAMP1 | carcinoembryonic antigen-related cell adhesion molecule pseudogene 1 | 19 | q13.2 |
| ENSG00000231561 | CEACAMP5 | carcinoembryonic antigen-related cell adhesion molecule pseudogene 5 | 19 | q13.2 |
| ENSG00000238092 | CEACAMP6 | carcinoembryonic antigen-related cell adhesion molecule pseudogene 6 | 19 | q13.2 |
| ENSG00000227349 | CEACAMP7 | carcinoembryonic antigen-related cell adhesion molecule pseudogene 7 | 19 | q13.31 |
| ENSG00000236932 | CEACAMP8 | carcinoembryonic antigen-related cell adhesion molecule pseudogene 8 | 19 | q13.31 |
| ENSG00000230529 | CEACAMP9 | carcinoembryonic antigen-related cell adhesion molecule pseudogene 9 | 19 | q13.31 |
| ENSG00000236123 | CEACAMP11 | carcinoembryonic antigen-related cell adhesion molecule pseudogene 11 | 19 | q13.31 |
| ENSG00000230681 | CEACAMP4 | carcinoembryonic antigen-related cell adhesion molecule pseudogene 4 | 19 | q13.31 |
| ENSG00000230666 | CEACAM22P | carcinoembryonic antigen-related cell adhesion molecule 2, pseudogene | 19 | q13.31 |
| ENSG00000234906 | APOC2 | apolipoprotein C-II | 19 | q13.32 |
| ENSG00000236417 | CTSL1P1 | cathepsin L1 pseudogene 1 | 10 | q23.2 |
| ENSG00000227268 | KLLN | killin, p53-regulated DNA replication inhibitor | 10 | q23.31 |
| ENSG00000224289 | IFIT6P | interferon-induced protein with tetratricopeptide repeats 6, pseudogene | 10 | q23.31 |
| ENSG00000224607 | IGKV1D-27 | immunoglobulin kappa variable 1D-27 (pseudogene) | 2 | p11.2 |
| ENSG00000232747 | IGKV1D-35 | immunoglobulin kappa variable 1D-35 (pseudogene) | 2 | p11.2 |
| ENSG00000225523 | IGKV6D-21 | immunoglobulin kappa variable 6D-21 (non-functional) | 2 | p11.2 |
| ENSG00000224041 | IGKV3D-15 | immunoglobulin kappa variable 3D-15 (gene/pseudogene) | 2 | p11.2 |
| ENSG00000233859 | ADH5P4 | alcohol dehydrogenase 5 (class III), chi polypeptide, pseudogene 4 | 6 | q12 |
| ENSG00000223953 | C1QTNF5 | C1q and tumor necrosis factor related protein 5 | 11 | q23.3 |
| ENSG00000234224 | TMEM229A | transmembrane protein 229A | 7 | q31.32 |
| ENSG00000232676 | ADH5P2 | alcohol dehydrogenase 5 (class III), chi polypeptide, pseudogene 2 | 1 | p31.1 |
| ENSG00000226652 | PSMD10P2 | proteasome 26S subunit, non-ATPase, 10 pseudogene 2 | 3 | q27.3 |
| ENSG00000234518 | PTGES3P1 | prostaglandin E synthase 3 (cytosolic) pseudogene 1 | 1 | p22.2 |
| ENSG00000236548 | IDBG-309143 | six-twelve leukemia (STL), non-coding RNA | 6 | q22.31 |
| ENSG00000226660 | TRBV2 | T cell receptor beta variable 2 | 7 | q34 |
| ENSG00000237702 | TRBV3-1 | T cell receptor beta variable 3-1 | 7 | q34 |
| ENSG00000230099 | TRBV5-4 | T cell receptor beta variable 5-4 | 7 | q34 |
| ENSG00000227550 | TRBV7-5 | T cell receptor beta variable 7-5 (pseudogene) | 7 | q34 |
| ENSG00000229769 | TRBV10-2 | T cell receptor beta variable 10-2 | 7 | q34 |
| ENSG00000232869 | TRBV29-1 | T cell receptor beta variable 29-1 | 7 | q34 |
| ENSG00000237254 | TRBV30 | T cell receptor beta variable 30 (gene/pseudogene) | 7 | q34 |
| ENSG00000225556 | C2CD4D | C2 calcium-dependent domain containing 4D | 1 | q21.3 |
| ENSG00000229150 | CRYGEP | crystallin, gamma E, pseudogene | 2 | q33.3 |
| ENSG00000235444 | PSMB3P2 | proteasome (prosome, macropain) subunit, beta type, 3 pseudogene 2 | 2 | q35 |
| ENSG00000229754 | CXCR2P1 | chemokine (C-X-C motif) receptor 2 pseudogene 1 | 2 | q35 |
| ENSG00000223603 | CRPP1 | C-reactive protein pseudogene 1 | 1 | q23.2 |
| ENSG00000233608 | TWIST2 | twist homolog 2 (Drosophila) | 2 | q37.3 |
| ENSG00000223694 | ADH5P3 | alcohol dehydrogenase 5 (class III), chi polypeptide, pseudogene 3 | 1 | q43 |
| ENSG00000223997 | TRDD1 | T cell receptor delta diversity 1 | 14 | q11.2 |
| ENSG00000237235 | TRDD2 | T cell receptor delta diversity 2 | 14 | q11.2 |
| ENSG00000228985 | TRDD3 | T cell receptor delta diversity 3 | 14 | q11.2 |
| ENSG00000236597 | IGHD7-27 | immunoglobulin heavy diversity 7-27 | 14 | q32.33 |
| ENSG00000225825 | IGHD6-25 | immunoglobulin heavy diversity 6-25 | 14 | q32.33 |
| ENSG00000227196 | IGHD4-23 | immunoglobulin heavy diversity 4-23 (non-functional) | 14 | q32.33 |
| ENSG00000237020 | IGHD1-20 | immunoglobulin heavy diversity 1-20 | 14 | q32.33 |
| ENSG00000227800 | IGHD4-17 | immunoglobulin heavy diversity 4-17 | 14 | q32.33 |
| ENSG00000227108 | IGHD1-14 | immunoglobulin heavy diversity 1-14 (non-functional) | 14 | q32.33 |
| ENSG00000232543 | IGHD4-11 | immunoglobulin heavy diversity 4-11 (non-functional) | 14 | q32.33 |
| ENSG00000237197 | IGHD1-7 | immunoglobulin heavy diversity 1-7 | 14 | q32.33 |
| ENSG00000228131 | IGHD6-6 | immunoglobulin heavy diversity 6-6 | 14 | q32.33 |
| ENSG00000233655 | IGHD4-4 | immunoglobulin heavy diversity 4-4 | 14 | q32.33 |
| ENSG00000236170 | IGHD1-1 | immunoglobulin heavy diversity 1-1 | 14 | q32.33 |
| ENSG00000249624 | IFNAR2 | Uncharacterized protein | 21 | q22.11 |
| ENSG00000243646 | IL10RB | interleukin 10 receptor, beta | 21 | q22.11 |
| ENSG00000240654 | C1QTNF9 | C1q and tumor necrosis factor related protein 9 | 13 | q12.12 |
| ENSG00000240972 | MIF | macrophage migration inhibitory factor (glycosylation-inhibiting factor) | 22 | q11.23 |
| ENSG00000244486 | SCARF2 | scavenger receptor class F, member 2 | 22 | q11.21 |
| ENSG00000250868 | XKRY | XK, Kell blood group complex subunit-related, Y-linked | Y | q11.222 |
| ENSG00000244646 | XKRY | XK, Kell blood group complex subunit-related, Y-linked | Y | q11.222 |
| ENSG00000242114 | MTFP1 | mitochondrial fission process 1 | 22 | q12.2 |
| ENSG00000242173 | ARHGDIG | Rho GDP dissociation inhibitor (GDI) gamma | 16 | p13.3 |
| ENSG00000253626 | EIF5AL1 | eukaryotic translation initiation factor 5A-like 1 | 10 | q22.3 |
| ENSG00000249437 | NAIP | NLR family, apoptosis inhibitory protein | 5 | q13.2 |
| ENSG00000240247 | DEFA1B | defensin, alpha 1B | 8 | p23.1 |
| ENSG00000239839 | DEFA3 | defensin, alpha 3, neutrophil-specific | 8 | p23.1 |
| ENSG00000251694 | USP17L9P | ubiquitin specific peptidase 17-like family member 9, pseudogene | 4 | p16.1 |
| ENSG00000243244 | STON1 | stonin 1 | 2 | p16.3 |
| ENSG00000243509 | TNFRSF6B | tumor necrosis factor receptor superfamily, member 6b, decoy | 20 | q13.33 |
| ENSG00000240053 | LY6G5B | lymphocyte antigen 6 complex, locus G5B | 6 | p21.33 |
| ENSG00000244349 | HCG16 | HLA complex group 16 | 6 | p22.1 |
| ENSG00000253655 | IGJP1 | immunoglobulin J polypeptide pseudogene 1 | 8 | q21.2 |
| ENSG00000244355 | LY6G6D | lymphocyte antigen 6 complex, locus G6D | 6 | p21.33 |
| ENSG00000241106 | HLA-DOB | major histocompatibility complex, class II, DO beta | 6 | p21.32 |
| ENSG00000243753 | HLA-L | major histocompatibility complex, class I, L (pseudogene) | 6 | p22.1 |
| ENSG00000253958 | CLDN23 | claudin 23 | 8 | p23.1 |
| ENSG00000254203 | IGHVII-33-1 | immunoglobulin heavy variable (II)-33-1 (pseudogene) | 14 | q32.33 |
| ENSG00000253440 | IGHV3-33-2 | immunoglobulin heavy variable 3-33-2 (pseudogene) | 14 | q32.33 |
| ENSG00000253325 | IGHV7-34-1 | immunoglobulin heavy variable 7-34-1 (pseudogene) | 14 | q32.33 |
| ENSG00000253240 | IGHV3-36 | immunoglobulin heavy variable 3-36 (pseudogene) | 14 | q32.33 |
| ENSG00000253359 | IGHV3-37 | immunoglobulin heavy variable 3-37 (pseudogene) | 14 | q32.33 |
| ENSG00000250264 | TAP2 | Uncharacterized protein | 6 | p21.32 |
| ENSG00000253989 | IGHVIII-38-1 | immunoglobulin heavy variable (III)-38-1 (pseudogene) | 14 | q32.33 |
| ENSG00000253467 | IGHV7-40 | immunoglobulin heavy variable 7-40 (pseudogene) | 14 | q32.33 |
| ENSG00000253294 | IGHVII-40-1 | immunoglobulin heavy variable (II)-40-1 (pseudogene) | 14 | q32.33 |
| ENSG00000254228 | IGHV3-42 | immunoglobulin heavy variable 3-42 (pseudogene) | 14 | q32.33 |
| ENSG00000253588 | IGHVIII-44 | immunoglobulin heavy variable (III)-44 (pseudogene) | 14 | q32.33 |
| ENSG00000253465 | IGHVIV-44-1 | immunoglobulin heavy variable (IV)-44-1 (pseudogene) | 14 | q32.33 |
| ENSG00000253895 | IGHVII-44-2 | immunoglobulin heavy variable (II)-44-2 (pseudogene) | 14 | q32.33 |
| ENSG00000253808 | IGHVII-46-1 | immunoglobulin heavy variable (II)-46-1 (pseudogene) | 14 | q32.33 |
| ENSG00000253862 | IGHVIII-47-1 | immunoglobulin heavy variable (III)-47-1 (pseudogene) | 14 | q32.33 |
| ENSG00000253386 | IGHVII-49-1 | immunoglobulin heavy variable (II)-49-1 (pseudogene) | 14 | q32.33 |
| ENSG00000253241 | IGHV3-50 | immunoglobulin heavy variable 3-50 (pseudogene) | 14 | q32.33 |
| ENSG00000254167 | IGHVIII-51-1 | immunoglobulin heavy variable (III)-51-1 (pseudogene) | 14 | q32.33 |
| ENSG00000253941 | IGHVII-51-2 | immunoglobulin heavy variable (II)-51-2 (pseudogene) | 14 | q32.33 |
| ENSG00000253545 | IGHV3-52 | immunoglobulin heavy variable 3-52 (pseudogene) | 14 | q32.33 |
| ENSG00000253714 | IGHVII-53-1 | immunoglobulin heavy variable (II)-53-1 (pseudogene) | 14 | q32.33 |
| ENSG00000254395 | IGHV4-55 | immunoglobulin heavy variable 4-55 (pseudogene) | 14 | q32.33 |
| ENSG00000253131 | IGHV7-56 | immunoglobulin heavy variable 7-56 (pseudogene) | 14 | q32.33 |
| ENSG00000253759 | IGHV3-57 | immunoglobulin heavy variable 3-57 (pseudogene) | 14 | q32.33 |
| ENSG00000240065 | PSMB9 | proteasome (prosome, macropain) subunit, beta type, 9 (large multifunctional peptidase 2) | 6 | p21.32 |
| ENSG00000253742 | IGHV3-60 | immunoglobulin heavy variable 3-60 (pseudogene) | 14 | q32.33 |
| ENSG00000254329 | IGHVII-60-1 | immunoglobulin heavy variable (II)-60-1 (pseudogene) | 14 | q32.33 |
| ENSG00000253132 | IGHV3-62 | immunoglobulin heavy variable 3-62 (pseudogene) | 14 | q32.33 |
| ENSG00000253747 | IGHVII-62-1 | immunoglobulin heavy variable (II)-62-1 (pseudogene) | 14 | q32.33 |
| ENSG00000253936 | IGHV3-63 | immunoglobulin heavy variable 3-63 (pseudogene) | 14 | q32.33 |
| ENSG00000253209 | IGHV3-65 | immunoglobulin heavy variable 3-65 (pseudogene) | 14 | q32.33 |
| ENSG00000253169 | IGHVII-65-1 | immunoglobulin heavy variable (II)-65-1 (pseudogene) | 14 | q32.33 |
| ENSG00000253274 | IGHV1-67 | immunoglobulin heavy variable 1-67 (pseudogene) | 14 | q32.33 |
| ENSG00000253820 | IGHVII-67-1 | immunoglobulin heavy variable (II)-67-1 (pseudogene) | 14 | q32.33 |
| ENSG00000254036 | IGHVIII-67-2 | immunoglobulin heavy variable (III)-67-2 (pseudogene) | 14 | q32.33 |
| ENSG00000253635 | IGHVIII-67-3 | immunoglobulin heavy variable (III)-67-3 (pseudogene) | 14 | q32.33 |
| ENSG00000254052 | IGHVIII-67-4 | immunoglobulin heavy variable (III)-67-4 (pseudogene) | 14 | q32.33 |
| ENSG00000253703 | IGHV1-68 | immunoglobulin heavy variable 1-68 (pseudogene) | 14 | q32.33 |
| ENSG00000254056 | IGHV3-71 | immunoglobulin heavy variable 3-71 (pseudogene) | 14 | q32.33 |
| ENSG00000254134 | IGHVII-74-1 | immunoglobulin heavy variable (II)-74-1 (pseudogene) | 14 | q32.33 |
| ENSG00000254176 | IGHV3-75 | immunoglobulin heavy variable 3-75 (pseudogene) | 14 | q32.33 |
| ENSG00000253247 | IGHV3-76 | immunoglobulin heavy variable 3-76 (pseudogene) | 14 | q32.33 |
| ENSG00000253310 | IGHVIII-76-1 | immunoglobulin heavy variable (III)-76-1 (pseudogene) | 14 | q32.33 |
| ENSG00000253674 | IGHVII-78-1 | immunoglobulin heavy variable (II)-78-1 (pseudogene) | 14 | q32.33 |
| ENSG00000253303 | IGHVIII-82 | immunoglobulin heavy variable (III)-82 (pseudogene) | 14 | q32.33 |
| ENSG00000253435 | IGKV2-4 | immunoglobulin kappa variable 2-4 (pseudogene) | 2 | p11.2 |
| ENSG00000243466 | IGKV1-5 | immunoglobulin kappa variable 1-5 | 2 | p11.2 |
| ENSG00000239855 | IGKV1-6 | immunoglobulin kappa variable 1-6 | 2 | p11.2 |
| ENSG00000243063 | IGKV3-7 | immunoglobulin kappa variable 3-7 (non-functional) | 2 | p11.2 |
| ENSG00000240671 | IGKV1-8 | immunoglobulin kappa variable 1-8 | 2 | p11.2 |
| ENSG00000241755 | IGKV1-9 | immunoglobulin kappa variable 1-9 | 2 | p11.2 |
| ENSG00000253278 | IGKV2-10 | immunoglobulin kappa variable 2-10 (pseudogene) | 2 | p11.2 |
| ENSG00000241351 | IGKV3-11 | immunoglobulin kappa variable 3-11 | 2 | p11.2 |
| ENSG00000243290 | IGKV1-12 | immunoglobulin kappa variable 1-12 | 2 | p11.2 |
| ENSG00000253497 | IGKV1-13 | immunoglobulin kappa variable 1-13 (gene/pseudogene) | 2 | p11.2 |
| ENSG00000253265 | IGKV2-14 | immunoglobulin kappa variable 2-14 (pseudogene) | 2 | p11.2 |
| ENSG00000244437 | IGKV3-15 | immunoglobulin kappa variable 3-15 | 2 | p11.2 |
| ENSG00000240864 | IGKV1-16 | immunoglobulin kappa variable 1-16 | 2 | p11.2 |
| ENSG00000240382 | IGKV1-17 | immunoglobulin kappa variable 1-17 | 2 | p11.2 |
| ENSG00000254157 | IGKV2-18 | immunoglobulin kappa variable 2-18 (pseudogene) | 2 | p11.2 |
| ENSG00000253732 | IGKV2-19 | immunoglobulin kappa variable 2-19 (pseudogene) | 2 | p11.2 |
| ENSG00000239951 | IGKV3-20 | immunoglobulin kappa variable 3-20 | 2 | p11.2 |
| ENSG00000253578 | IGKV1-22 | immunoglobulin kappa variable 1-22 (pseudogene) | 2 | p11.2 |
| ENSG00000253625 | IGKV2-23 | immunoglobulin kappa variable 2-23 (pseudogene) | 2 | p11.2 |
| ENSG00000241294 | IGKV2-24 | immunoglobulin kappa variable 2-24 | 2 | p11.2 |
| ENSG00000253202 | IGKV3-25 | immunoglobulin kappa variable 3-25 (pseudogene) | 2 | p11.2 |
| ENSG00000254098 | IGKV2-26 | immunoglobulin kappa variable 2-26 (pseudogene) | 2 | p11.2 |
| ENSG00000244575 | IGKV1-27 | immunoglobulin kappa variable 1-27 | 2 | p11.2 |
| ENSG00000244116 | IGKV2-28 | immunoglobulin kappa variable 2-28 | 2 | p11.2 |
| ENSG00000253998 | IGKV2-29 | immunoglobulin kappa variable 2-29 (gene/pseudogene) | 2 | p11.2 |
| ENSG00000243238 | IGKV2-30 | immunoglobulin kappa variable 2-30 | 2 | p11.2 |
| ENSG00000253158 | IGKV3-31 | immunoglobulin kappa variable 3-31 (pseudogene) | 2 | p11.2 |
| ENSG00000253870 | IGKV1-32 | immunoglobulin kappa variable 1-32 (pseudogene) | 2 | p11.2 |
| ENSG00000242076 | IGKV1-33 | immunoglobulin kappa variable 1-33 | 2 | p11.2 |
| ENSG00000253860 | IGKV3-34 | immunoglobulin kappa variable 3-34 (pseudogene) | 2 | p11.2 |
| ENSG00000253461 | IGKV1-35 | immunoglobulin kappa variable 1-35 (pseudogene) | 2 | p11.2 |
| ENSG00000253487 | IGKV2-36 | immunoglobulin kappa variable 2-36 (pseudogene) | 2 | p11.2 |
| ENSG00000239862 | IGKV1-37 | immunoglobulin kappa variable 1-37 (non-functional) | 2 | p11.2 |
| ENSG00000242574 | HLA-DMB | major histocompatibility complex, class II, DM beta | 6 | p21.32 |
| ENSG00000253592 | IGKV2-38 | immunoglobulin kappa variable 2-38 (pseudogene) | 2 | p11.2 |
| ENSG00000242371 | IGKV1-39 | immunoglobulin kappa variable 1-39 (gene/pseudogene) | 2 | p11.2 |
| ENSG00000251039 | IGKV2D-40 | immunoglobulin kappa variable 2D-40 | 2 | p11.2 |
| ENSG00000251546 | IGKV1D-39 | immunoglobulin kappa variable 1D-39 | 2 | p11.2 |
| ENSG00000254009 | IGKV2D-38 | immunoglobulin kappa variable 2D-38 (pseudogene) | 2 | p11.2 |
| ENSG00000250036 | IGKV1D-37 | immunoglobulin kappa variable 1D-37 (non-functional) | 2 | p11.2 |
| ENSG00000253127 | IGKV2D-36 | immunoglobulin kappa variable 2D-36 (pseudogene) | 2 | p11.2 |
| ENSG00000253501 | IGKV3D-34 | immunoglobulin kappa variable 3D-34 (pseudogene) | 2 | p11.2 |
| ENSG00000239975 | IGKV1D-33 | immunoglobulin kappa variable 1D-33 | 2 | p11.2 |
| ENSG00000253191 | IGKV1D-32 | immunoglobulin kappa variable 1D-32 (pseudogene) | 2 | p11.2 |
| ENSG00000253999 | IGKV3D-31 | immunoglobulin kappa variable 3D-31 (pseudogene) | 2 | p11.2 |
| ENSG00000239571 | IGKV2D-30 | immunoglobulin kappa variable 2D-30 | 2 | p11.2 |
| ENSG00000243264 | IGKV2D-29 | immunoglobulin kappa variable 2D-29 | 2 | p11.2 |
| ENSG00000242534 | IGKV2D-28 | immunoglobulin kappa variable 2D-28 | 2 | p11.2 |
| ENSG00000254097 | IGKV3D-25 | immunoglobulin kappa variable 3D-25 (pseudogene) | 2 | p11.2 |
| ENSG00000241566 | IGKV2D-24 | immunoglobulin kappa variable 2D-24 (non-functional) | 2 | p11.2 |
| ENSG00000254345 | IGKV2D-23 | immunoglobulin kappa variable 2D-23 (pseudogene) | 2 | p11.2 |
| ENSG00000253365 | IGKV1D-22 | immunoglobulin kappa variable 1D-22 (pseudogene) | 2 | p11.2 |
| ENSG00000241399 | CD302 | CD302 molecule | 2 | q24.2 |
| ENSG00000253765 | IGKV2D-19 | immunoglobulin kappa variable 2D-19 (pseudogene) | 2 | p11.2 |
| ENSG00000254220 | IGKV2D-18 | immunoglobulin kappa variable 2D-18 (pseudogene) | 2 | p11.2 |
| ENSG00000242766 | IGKV1D-17 | immunoglobulin kappa variable 1D-17 | 2 | p11.2 |
| ENSG00000241244 | IGKV1D-16 | immunoglobulin kappa variable 1D-16 | 2 | p11.2 |
| ENSG00000254292 | IGKV2D-14 | immunoglobulin kappa variable 2D-14 (pseudogene) | 2 | p11.2 |
| ENSG00000249048 | TRAV31 | T cell receptor alpha variable 31 (pseudogene) | 14 | q11.2 |
| ENSG00000253906 | IGKV2D-10 | immunoglobulin kappa variable 2D-10 (pseudogene) | 2 | p11.2 |
| ENSG00000242580 | IGKV1D-43 | immunoglobulin kappa variable 1D-43 | 2 | p11.2 |
| ENSG00000239819 | IGKV1D-8 | immunoglobulin kappa variable 1D-8 | 2 | p11.2 |
| ENSG00000249446 | TRAJ60 | T cell receptor alpha joining 60 (pseudogene) | 14 | q11.2 |
| ENSG00000242736 | TRBV1 | T cell receptor beta variable 1 (pseudogene) | 7 | q34 |
| ENSG00000250688 | TRAJ55 | T cell receptor alpha joining 55 (pseudogene) | 14 | q11.2 |
| ENSG00000248366 | TRAJ51 | T cell receptor alpha joining 51 (pseudogene) | 14 | q11.2 |
| ENSG00000253291 | TRBV7-7 | T cell receptor beta variable 7-7 | 7 | q34 |
| ENSG00000253534 | TRBV6-8 | T cell receptor beta variable 6-8 | 7 | q34 |
| ENSG00000253188 | TRBV6-7 | T cell receptor beta variable 6-7 (non-functional) | 7 | q34 |
| ENSG00000253409 | TRBV7-4 | T cell receptor beta variable 7-4 (gene/pseudogene) | 7 | q34 |
| ENSG00000244661 | TRBV12-1 | T cell receptor beta variable 12-1 (pseudogene) | 7 | q34 |
| ENSG00000239944 | TRBV8-2 | T cell receptor beta variable 8-2 (pseudogene) | 7 | q34 |
| ENSG00000242771 | TRBV5-2 | T cell receptor beta variable 5-2 (pseudogene) | 7 | q34 |
| ENSG00000243889 | TRBV8-1 | T cell receptor beta variable 8-1 (pseudogene) | 7 | q34 |
| ENSG00000251578 | TRBV21-1 | T cell receptor beta variable 21-1 (pseudogene) | 7 | q34 |
| ENSG00000240578 | TRBV22-1 | T cell receptor beta variable 22-1 (pseudogene) | 7 | q34 |
| ENSG00000239992 | TRBVA | T cell receptor beta variable A (pseudogene) | 7 | q34 |
| ENSG00000249912 | TRBV26 | T cell receptor beta variable 26 (pseudogene) | 7 | q34 |
| ENSG00000241911 | TRBVB | T cell receptor beta variable B (pseudogene) | 7 | q34 |
| ENSG00000253691 | IGKV2OR22-4 | immunoglobulin kappa variable 2/OR22-4 (pseudogene) | 22 | q11.1 |
| ENSG00000253460 | IGKV2OR22-3 | immunoglobulin kappa variable 2/OR22-3 (pseudogene) | 22 | q11.1 |
| ENSG00000254264 | IGKV3OR22-2 | immunoglobulin kappa variable 3/OR22-2 (pseudogene) | 22 | q11.1 |
| ENSG00000253481 | IGKV1OR22-1 | immunoglobulin kappa variable 1/OR22-1 (pseudogene) | 22 | q11.1 |
| ENSG00000254017 | IGHEP2 | immunoglobulin heavy constant epsilon P2 (pseudogene) | 9 | p24.1 |
| ENSG00000253239 | IGLVI-70 | immunoglobulin lambda variable (I)-70 (pseudogene) | 22 | q11.22 |
| ENSG00000254355 | IGLVI-68 | immunoglobulin lambda variable (I)-68 (pseudogene) | 22 | q11.22 |
| ENSG00000253794 | IGLV10-67 | immunoglobulin lambda variable 10-67 (pseudogene) | 22 | q11.22 |
| ENSG00000253874 | IGLVIV-66-1 | immunoglobulin lambda variable (IV)-66-1 (pseudogene) | 22 | q11.22 |
| ENSG00000254075 | IGLVV-66 | immunoglobulin lambda variable (V)-66 (pseudogene) | 22 | q11.22 |
| ENSG00000254161 | IGLVIV-65 | immunoglobulin lambda variable (IV)-65 (pseudogene) | 22 | q11.22 |
| ENSG00000253242 | IGLVIV-64 | immunoglobulin lambda variable (IV)-64 (pseudogene) | 22 | q11.22 |
| ENSG00000253752 | IGLVI-63 | immunoglobulin lambda variable (I)-63 (pseudogene) | 22 | q11.22 |
| ENSG00000253823 | IGLV1-62 | immunoglobulin lambda variable 1-62 (pseudogene) | 22 | q11.22 |
| ENSG00000254308 | IGLVIV-59 | immunoglobulin lambda variable (IV)-59 (pseudogene) | 22 | q11.22 |
| ENSG00000253637 | IGLVV-58 | immunoglobulin lambda variable (V)-58 (pseudogene) | 22 | q11.22 |
| ENSG00000248993 | HLA-DMB | Major histocompatibility complex, class II, DM beta | 6 | p21.32 |
| ENSG00000253126 | IGLVI-56 | immunoglobulin lambda variable (I)-56 (pseudogene) | 22 | q11.22 |
| ENSG00000253935 | IGLVIV-53 | immunoglobulin lambda variable (IV)-53 (pseudogene) | 22 | q11.22 |
| ENSG00000254175 | IGLVI-42 | immunoglobulin lambda variable (I)-42 (pseudogene) | 22 | q11.22 |
| ENSG00000254073 | IGLVVII-41-1 | immunoglobulin lambda variable (VII)-41-1 (pseudogene) | 22 | q11.22 |
| ENSG00000253818 | IGLV1-41 | immunoglobulin lambda variable 1-41 (pseudogene) | 22 | q11.22 |
| ENSG00000253889 | IGLVI-38 | immunoglobulin lambda variable (I)-38 (pseudogene) | 22 | q11.22 |
| ENSG00000253631 | IGLV7-35 | immunoglobulin lambda variable 7-35 (pseudogene) | 22 | q11.22 |
| ENSG00000253120 | IGLV2-34 | immunoglobulin lambda variable 2-34 (pseudogene) | 22 | q11.22 |
| ENSG00000253920 | IGLV3-31 | immunoglobulin lambda variable 3-31 (pseudogene) | 22 | q11.22 |
| ENSG00000253329 | IGLV3-30 | immunoglobulin lambda variable 3-30 (pseudogene) | 22 | q11.22 |
| ENSG00000253338 | IGLV3-29 | immunoglobulin lambda variable 3-29 (pseudogene) | 22 | q11.22 |
| ENSG00000253451 | IGLV2-28 | immunoglobulin lambda variable 2-28 (pseudogene) | 22 | q11.22 |
| ENSG00000253913 | IGLV3-26 | immunoglobulin lambda variable 3-26 (pseudogene) | 22 | q11.22 |
| ENSG00000253779 | IGLVVI-25-1 | immunoglobulin lambda variable (VI)-25-1 (pseudogene) | 22 | q11.22 |
| ENSG00000253822 | IGLV3-24 | immunoglobulin lambda variable 3-24 (pseudogene) | 22 | q11.22 |
| ENSG00000253546 | IGLVVI-22-1 | immunoglobulin lambda variable (VI)-22-1 (pseudogene) | 22 | q11.22 |
| ENSG00000254240 | IGLVI-20 | immunoglobulin lambda variable (I)-20 (pseudogene) | 22 | q11.22 |
| ENSG00000253152 | IGLV3-17 | immunoglobulin lambda variable 3-17 (pseudogene) | 22 | q11.22 |
| ENSG00000253786 | IGLV3-15 | immunoglobulin lambda variable 3-15 (pseudogene) | 22 | q11.22 |
| ENSG00000253590 | IGLV3-13 | immunoglobulin lambda variable 3-13 (pseudogene) | 22 | q11.22 |
| ENSG00000254077 | IGLV3-7 | immunoglobulin lambda variable 3-7 (pseudogene) | 22 | q11.22 |
| ENSG00000253448 | IGLV3-6 | immunoglobulin lambda variable 3-6 (pseudogene) | 22 | q11.22 |
| ENSG00000253234 | IGLV2-5 | immunoglobulin lambda variable 2-5 (pseudogene) | 22 | q11.22 |
| ENSG00000253963 | IGLV3-2 | immunoglobulin lambda variable 3-2 (pseudogene) | 22 | q11.22 |
| ENSG00000254029 | IGLC4 | immunoglobulin lambda constant 4 (pseudogene) | 22 | q11.22 |
| ENSG00000254030 | IGLC5 | immunoglobulin lambda constant 5 (pseudogene) | 22 | q11.22 |
| ENSG00000243667 | WDR92 | WD repeat domain 92 | 2 | p14 |
| ENSG00000253755 | IGHGP | immunoglobulin heavy constant gamma P (non-functional) | 14 | q32.33 |
| ENSG00000253692 | IGHEP1 | immunoglobulin heavy constant epsilon P1 (pseudogene) | 14 | q32.33 |
| ENSG00000242472 | IGHJ5 | immunoglobulin heavy joining 5 | 14 | q32.33 |
| ENSG00000240041 | IGHJ4 | immunoglobulin heavy joining 4 | 14 | q32.33 |
| ENSG00000242887 | IGHJ3 | immunoglobulin heavy joining 3 | 14 | q32.33 |
| ENSG00000239649 | MYADML | myeloid-associated differentiation marker-like (pseudogene) | 2 | p22.3 |
| ENSG00000249978 | TRGV7 | T cell receptor gamma variable 7 (pseudogene) | 7 | p14.1 |
| ENSG00000254127 | IGLCOR22-1 | immunoglobulin lambda constant/OR22-1 (pseudogene) | 22 | q12.3 |
| ENSG00000254279 | IGHVII-1-1 | immunoglobulin heavy variable (II)-1-1 (pseudogene) | 14 | q32.33 |
| ENSG00000253780 | IGHVIII-2-1 | immunoglobulin heavy variable (III)-2-1 (pseudogene) | 14 | q32.33 |
| ENSG00000253387 | IGHVIII-5-1 | immunoglobulin heavy variable (III)-5-1 (pseudogene) | 14 | q32.33 |
| ENSG00000254053 | IGHVIII-5-2 | immunoglobulin heavy variable (III)-5-2 (pseudogene) | 14 | q32.33 |
| ENSG00000253763 | IGHV3-6 | immunoglobulin heavy variable 3-6 (pseudogene) | 14 | q32.33 |
| ENSG00000254215 | IGHVIII-11-1 | immunoglobulin heavy variable (III)-11-1 (pseudogene) | 14 | q32.33 |
| ENSG00000254174 | IGHV1-12 | immunoglobulin heavy variable 1-12 (pseudogene) | 14 | q32.33 |
| ENSG00000253412 | IGHVIII-13-1 | immunoglobulin heavy variable (III)-13-1 (pseudogene) | 14 | q32.33 |
| ENSG00000253709 | IGHV1-14 | immunoglobulin heavy variable 1-14 (pseudogene) | 14 | q32.33 |
| ENSG00000253458 | IGHVII-15-1 | immunoglobulin heavy variable (II)-15-1 (pseudogene) | 14 | q32.33 |
| ENSG00000254046 | IGHV1-17 | immunoglobulin heavy variable 1-17 (pseudogene) | 14 | q32.33 |
| ENSG00000253883 | IGHV3-19 | immunoglobulin heavy variable 3-19 (pseudogene) | 14 | q32.33 |
| ENSG00000253957 | IGHV3-22 | immunoglobulin heavy variable 3-22 (pseudogene) | 14 | q32.33 |
| ENSG00000253345 | IGHVII-22-1 | immunoglobulin heavy variable (II)-22-1 (pseudogene) | 14 | q32.33 |
| ENSG00000254045 | IGHVIII-22-2 | immunoglobulin heavy variable (III)-22-2 (pseudogene) | 14 | q32.33 |
| ENSG00000253441 | IGHV3-25 | immunoglobulin heavy variable 3-25 (pseudogene) | 14 | q32.33 |
| ENSG00000253367 | IGHVIII-25-1 | immunoglobulin heavy variable (III)-25-1 (pseudogene) | 14 | q32.33 |
| ENSG00000253462 | IGHVIII-26-1 | immunoglobulin heavy variable (III)-26-1 (pseudogene) | 14 | q32.33 |
| ENSG00000253482 | IGHVII-26-2 | immunoglobulin heavy variable (II)-26-2 (pseudogene) | 14 | q32.33 |
| ENSG00000254326 | IGHV7-27 | immunoglobulin heavy variable 7-27 (pseudogene) | 14 | q32.33 |
| ENSG00000253491 | IGHVII-30-1 | immunoglobulin heavy variable (II)-30-1 (pseudogene) | 14 | q32.33 |
| ENSG00000253587 | IGHV3-30-2 | immunoglobulin heavy variable 3-30-2 (pseudogene) | 14 | q32.33 |
| ENSG00000253149 | IGHVII-31-1 | immunoglobulin heavy variable (II)-31-1 (pseudogene) | 14 | q32.33 |
| ENSG00000254289 | IGHV3-32 | immunoglobulin heavy variable 3-32 (pseudogene) | 14 | q32.33 |
| ENSG00000244703 | CD46P1 | CD46 molecule, complement regulatory protein pseudogene 1 | 1 | q32.2 |
| ENSG00000244171 | PBX2P1 | pre-B-cell leukemia homeobox 2 pseudogene 1 | 3 | q24 |
| ENSG00000241506 | PSMC1P1 | proteasome (prosome, macropain) 26S subunit, ATPase, 1 pseudogene 1 | 3 | p14.1 |
| ENSG00000244255 | CFB | Complement factor B Ba fragment; Uncharacterized protein; cDNA FLJ55673, highly similar to Complement factor B | 6 | p21.33 |
| ENSG00000243414 | TICAM2 | toll-like receptor adaptor molecule 2 | 5 | q22.3 |
| ENSG00000250550 | PPBPP1 | pro-platelet basic protein pseudogene 1 | 4 | q13.3 |
| ENSG00000250339 | CXCL1P | chemokine (C-X-C motif) ligand 1 pseudogene | 4 | q13.3 |
| ENSG00000248848 | PPBPP2 | pro-platelet basic protein pseudogene 2 | 4 | q13.3 |
| ENSG00000240215 | TRBV25OR9-2 | T cell receptor beta variable 25/OR9-2 (pseudogene) | 9 | p13.3 |
| ENSG00000244122 | UGT1A7 | UDP glucuronosyltransferase 1 family, polypeptide A7 | 2 | q37.1 |
| ENSG00000240583 | AQP1 | aquaporin 1 (Colton blood group) | 7 | p14.3 |
| ENSG00000241635 | UGT1A1 | UDP glucuronosyltransferase 1 family, polypeptide A1 | 2 | q37.1 |
| ENSG00000243709 | LEFTY1 | left-right determination factor 1 | 1 | q42.12 |
| ENSG00000240403 | KIR3DL2 | killer cell immunoglobulin-like receptor, three domains, long cytoplasmic tail, 2 | 19 | q13.42 |
| ENSG00000242473 | KIR2DP1 | killer cell immunoglobulin-like receptor, two domains, pseudogene 1 | 19 | q13.42 |
| ENSG00000250424 | AQP1 | Uncharacterized protein | 7 | p14.3 |
| ENSG00000241657 | TRBV11-2 | T cell receptor beta variable 11-2 | 7 | q34 |
| ENSG00000239732 | TLR9 | toll-like receptor 9 | 3 | p21.2 |
| ENSG00000251493 | FOXD1 | forkhead box D1 | 5 | q13.2 |
| ENSG00000239697 | TNFSF12 | tumor necrosis factor (ligand) superfamily, member 12 | 17 | p13.1 |
| ENSG00000248871 | TNFSF12-TNFSF13 | TNFSF12-TNFSF13 readthrough | 17 | p13.1 |
| ENSG00000253729 | PRKDC | protein kinase, DNA-activated, catalytic polypeptide | 8 | q11.21 |
| ENSG00000254126 | CD8BP | CD8b molecule pseudogene | 2 | q12.2 |
| ENSG00000250120 | PCDHA10 | protocadherin alpha 10 | 5 | q31.3 |
| ENSG00000249158 | PCDHA11 | protocadherin alpha 11 | 5 | q31.3 |
| ENSG00000251664 | PCDHA12 | protocadherin alpha 12 | 5 | q31.3 |
| ENSG00000239389 | PCDHA13 | protocadherin alpha 13 | 5 | q31.3 |
| ENSG00000248383 | PCDHAC1 | protocadherin alpha subfamily C, 1 | 5 | q31.3 |
| ENSG00000243232 | PCDHAC2 | protocadherin alpha subfamily C, 2 | 5 | q31.3 |
| ENSG00000251537 | TRIM16 | Uncharacterized protein | 17 | p12 |
| ENSG00000242252 | BGLAP | bone gamma-carboxyglutamate (gla) protein | 1 | q22 |
| ENSG00000254245 | PCDHGA3 | protocadherin gamma subfamily A, 3 | 5 | q31.3 |
| ENSG00000254221 | PCDHGB1 | protocadherin gamma subfamily B, 1 | 5 | q31.3 |
| ENSG00000253910 | PCDHGB2 | protocadherin gamma subfamily B, 2 | 5 | q31.3 |
| ENSG00000239264 | TXNDC5 | thioredoxin domain containing 5 (endoplasmic reticulum) | 6 | p24.3 |
| ENSG00000253485 | PCDHGA5 | protocadherin gamma subfamily A, 5 | 5 | q31.3 |
| ENSG00000253731 | PCDHGA6 | protocadherin gamma subfamily A, 6 | 5 | q31.3 |
| ENSG00000253537 | PCDHGA7 | protocadherin gamma subfamily A, 7 | 5 | q31.3 |
| ENSG00000253953 | PCDHGB4 | protocadherin gamma subfamily B, 4 | 5 | q31.3 |
| ENSG00000253767 | PCDHGA8 | protocadherin gamma subfamily A, 8 | 5 | q31.3 |
| ENSG00000253305 | PCDHGB6 | protocadherin gamma subfamily B, 6 | 5 | q31.3 |
| ENSG00000253846 | PCDHGA10 | protocadherin gamma subfamily A, 10 | 5 | q31.3 |
| ENSG00000249853 | HS3ST5 | heparan sulfate (glucosamine) 3-O-sulfotransferase 5 | 6 | q22.1 |
| ENSG00000254122 | PCDHGB7 | protocadherin gamma subfamily B, 7 | 5 | q31.3 |
| ENSG00000253873 | PCDHGA11 | protocadherin gamma subfamily A, 11 | 5 | q31.3 |
| ENSG00000253159 | PCDHGA12 | protocadherin gamma subfamily A, 12 | 5 | q31.3 |
| ENSG00000240184 | PCDHGC3 | protocadherin gamma subfamily C, 3 | 5 | q31.3 |
| ENSG00000242419 | PCDHGC4 | protocadherin gamma subfamily C, 4 | 5 | q31.3 |
| ENSG00000240764 | PCDHGC5 | protocadherin gamma subfamily C, 5 | 5 | q31.3 |
| ENSG00000240505 | TNFRSF13B | tumor necrosis factor receptor superfamily, member 13B | 17 | p11.2 |
| ENSG00000250361 | GYPB | glycophorin B (MNS blood group) | 4 | q31.21 |
| ENSG00000240280 | TCAM1P | testicular cell adhesion molecule 1 homolog (mouse), pseudogene | 17 | q23.3 |
| ENSG00000241186 | TDGF1 | teratocarcinoma-derived growth factor 1 | 3 | p21.31 |
| ENSG00000239736 | CEACAMP3 | carcinoembryonic antigen-related cell adhesion molecule pseudogene 3 | 19 | q13.2 |
| ENSG00000245848 | CEBPA | CCAAT/enhancer binding protein (C/EBP), alpha | 19 | q13.11 |
| ENSG00000242265 | PEG10 | paternally expressed 10 | 7 | q21.3 |
| ENSG00000240344 | PPIL3 | peptidylprolyl isomerase (cyclophilin)-like 3 | 2 | q33.1 |
| ENSG00000248333 | CDC2L1 | cyclin-dependent kinase 11B | 1 | p36.33 |
| ENSG00000241104 | CEACAMP10 | carcinoembryonic antigen-related cell adhesion molecule pseudogene 10 | 19 | q13.31 |
| ENSG00000243284 | VSIG8 | V-set and immunoglobulin domain containing 8 | 1 | q23.2 |
| ENSG00000244038 | DDOST | dolichyl-diphosphooligosaccharide--protein glycosyltransferase | 1 | p36.12 |
| ENSG00000249915 | PDCD6 | programmed cell death 6 | 5 | p15.33 |
| ENSG00000239672 | NME1 | NME/NM23 nucleoside diphosphate kinase 1 | 17 | q21.33 |
| ENSG00000242689 | CNTF | ciliary neurotrophic factor | 11 | q12.1 |
| ENSG00000244414 | CFHR1 | complement factor H-related 1 | 1 | q31.3 |
| ENSG00000243678 | NME1-NME2 | NME1-NME2 readthrough | 17 | q21.33 |
| ENSG00000249751 | ECSCR | endothelial cell surface expressed chemotaxis and apoptosis regulator | 5 | q31.2 |
| ENSG00000242296 | DEFB109P1 | defensin, beta 109, pseudogene 1 | 8 | p23.1 |
| ENSG00000248746 | ACTN3 | actinin, alpha 3 (gene/pseudogene) | 11 | q13.2 |
| ENSG00000244682 | FCGR2C | Fc fragment of IgG, low affinity IIc, receptor for (CD32) (gene/pseudogene) | 1 | q23.3 |
| ENSG00000242515 | UGT1A10 | UDP glucuronosyltransferase 1 family, polypeptide A10 | 2 | q37.1 |
| ENSG00000248385 | TARM1 | T cell-interacting, activating receptor on myeloid cells 1 | 19 | q13.42 |
| ENSG00000244482 | LILRA6 | leukocyte immunoglobulin-like receptor, subfamily A (with TM domain), member 6 | 19 | q13.42 |
| ENSG00000239961 | LILRA4 | leukocyte immunoglobulin-like receptor, subfamily A (with TM domain), member 4 | 19 | q13.42 |
| ENSG00000239998 | LILRA2 | leukocyte immunoglobulin-like receptor, subfamily A (with TM domain), member 2 | 19 | q13.42 |
| ENSG00000243772 | KIR2DL3 | killer cell immunoglobulin-like receptor, two domains, long cytoplasmic tail, 3 | 19 | q13.42 |
| ENSG00000242019 | KIR3DL3 | killer cell immunoglobulin-like receptor, three domains, long cytoplasmic tail, 3 | 19 | q13.42 |
| ENSG00000255569 | TRAV1-1 | T cell receptor alpha variable 1-1 | 14 | q11.2 |
| ENSG00000256553 | TRAV1-2 | T cell receptor alpha variable 1-2 | 14 | q11.2 |
| ENSG00000256474 | TRAV11 | T cell receptor alpha variable 11 (pseudogene) | 14 | q11.2 |
| ENSG00000256379 | TRAV8-5 | T cell receptor alpha variable 8-5 (pseudogene) | 14 | q11.2 |
| ENSG00000258482 | TRAV15 | T cell receptor alpha variable 15 (pseudogene) | 14 | q11.2 |
| ENSG00000258835 | TRAV28 | T cell receptor alpha variable 28 (pseudogene) | 14 | q11.2 |
| ENSG00000259092 | TRAV30 | T cell receptor alpha variable 30 | 14 | q11.2 |
| ENSG00000258905 | TRAV32 | T cell receptor alpha variable 32 (pseudogene) | 14 | q11.2 |
| ENSG00000258812 | TRAV33 | T cell receptor alpha variable 33 (pseudogene) | 14 | q11.2 |
| ENSG00000259068 | TRAV37 | T cell receptor alpha variable 37 (pseudogene) | 14 | q11.2 |
| ENSG00000256590 | TRDV3 | T cell receptor delta variable 3 | 14 | q11.2 |
| ENSG00000254717 | GLYATL1P2 | glycine-N-acyltransferase-like 1 pseudogene 2 | 11 | q12.1 |
| ENSG00000255189 | GLYATL1P1 | glycine-N-acyltransferase-like 1 pseudogene 1 | 11 | q12.1 |
| ENSG00000254399 | GLYATL1P4 | glycine-N-acyltransferase-like 1 pseudogene 4 | 11 | q12.1 |
| ENSG00000255151 | GLYATL1P3 | glycine-N-acyltransferase-like 1 pseudogene 3 | 11 | q12.1 |
| ENSG00000255544 | DEFB108P3 | defensin, beta 108, pseudogene 3 | 8 | p23.1 |
| ENSG00000254866 | DEFB109P3 | defensin, beta 109, pseudogene 3 | 8 | p23.1 |
| ENSG00000257207 | LIMS1 | LIM and senescent cell antigen-like-containing domain protein 3; LIM and senescent cell antigen-like-containing domain protein 3-like; Uncharacterized protein; cDNA FLJ59124, highly similar to Particularly interesting newCys-His protein; cDNA, FLJ79109, highly similar to Particularly interesting newCys-His protein | 2 | q13 |
| ENSG00000254623 | DEFB108P4 | defensin, beta 108, pseudogene 4 | 8 | p23.1 |
| ENSG00000254521 | SIGLEC12 | sialic acid binding Ig-like lectin 12 (gene/pseudogene) | 19 | q13.41 |
| ENSG00000254415 | SIGLEC14 | sialic acid binding Ig-like lectin 14 | 19 | q13.41 |
| ENSG00000254838 | GVINP1 | GTPase, very large interferon inducible pseudogene 1 | 11 | p15.4 |
| ENSG00000257780 | GLYCAM1 | glycosylation dependent cell adhesion molecule 1 (pseudogene) | 12 | q13.2 |
| ENSG00000259529 | RNF31 | E3 ubiquitin-protein ligase RNF31 | 14 | q12 |
| ENSG00000255398 | HCAR3 | hydroxycarboxylic acid receptor 3 | 12 | q24.31 |
| ENSG00000255641 | KLRC2 | NKG2-C type II integral membrane protein | 12 | p13.2 |
| ENSG00000256206 | PSMA1 | proteasome subunit alpha type-1 isoform 3 | 11 | p15.2 |
| ENSG00000255552 | LY6G6E | lymphocyte antigen 6 complex, locus G6E (pseudogene) | 6 | p21.33 |
| ENSG00000255819 | KLRK1 | NKG2-D type II integral membrane protein | 12 | p13.2 |
| ENSG00000256043 | CTSO | cathepsin O | 4 | q32.1 |
| ENSG00000259691 | FKBP1AP2 | FK506 binding protein 1A, 12kDa pseudogene 2 | 15 | q24.1 |
| ENSG00000259337 | IGHV1OR15-2 | immunoglobulin heavy variable 1/OR15-2 (pseudogene) | 15 | q11.1 |
| ENSG00000259490 | IGHV3OR15-7 | immunoglobulin heavy variable 3/OR15-7 (pseudogene) | 15 | q11.1 |
| ENSG00000258590 | NBEAP1 | neurobeachin pseudogene 1 | 15 | q11.2 |
| ENSG00000259261 | IGHV4OR15-8 | immunoglobulin heavy variable 4/OR15-8 (non-functional) | 15 | q11.2 |
| ENSG00000259753 | ITGB3 | Integrin beta | 17 | q21.32 |
| ENSG00000258311 | BLOC1S1 | Uncharacterized protein | 12 | q13.2 |
| ENSG00000258227 | CLEC5A | C-type lectin domain family 5, member A | 7 | q34 |
| ENSG00000257127 | CLLU1 | chronic lymphocytic leukemia up-regulated 1 | 12 | q22 |
| ENSG00000255221 | CARD17 | caspase recruitment domain family, member 17 | 11 | q22.3 |
| ENSG00000255501 | CARD18 | caspase recruitment domain family, member 18 | 11 | q22.3 |
| ENSG00000254709 | IGLL5 | immunoglobulin lambda-like polypeptide 5 | 22 | q11.22 |
| ENSG00000256950 | PSMD9 | 26S proteasome non-ATPase regulatory subunit 9 isoform 2 | 12 | q24.31 |
| ENSG00000258724 | SOX7 | Transcription factor SOX-7; Uncharacterized protein; cDNA FLJ58508, highly similar to Transcription factor SOX-7 | 8 | p23.1 |
| ENSG00000259303 | IGHV2OR16-5 | immunoglobulin heavy variable 2/OR16-5 (non-functional) | 16 | p11.2 |
| ENSG00000259680 | IGHV3OR16-7 | immunoglobulin heavy variable 3/OR16-7 (pseudogene) | 16 | p11.2 |
| ENSG00000256870 | SLC5A8 | solute carrier family 5 (iodide transporter), member 8 | 12 | q23.2 |
| ENSG00000258839 | MC1R | melanocortin 1 receptor (alpha melanocyte stimulating hormone receptor) | 16 | q24.3 |
| ENSG00000256977 | LIMS3 | LIM and senescent cell antigen-like domains 3 | 2 | q13 |
| ENSG00000258653 | PTGR2 | Uncharacterized protein | 14 | q24.3 |
| ENSG00000259207 | ITGB3 | integrin, beta 3 (platelet glycoprotein IIIa, antigen CD61) | 17 | q21.32 |
| ENSG00000257103 | LSM14A | LSM14A, SCD6 homolog A (S. cerevisiae) | 19 | q13.11 |
| ENSG00000254656 | RTL1 | retrotransposon-like 1 | 14 | q32.2 |
| ENSG00000254673 | FNTA | Uncharacterized protein | 8 | p11.21 |
| ENSG00000259571 | BLID | BH3-like motif containing, cell death inducer | 11 | q24.1 |
| ENSG00000254647 | INS | insulin | 11 | p15.5 |
| ENSG00000255408 | PCDHA3 | protocadherin alpha 3 | 5 | q31.3 |
| ENSG00000255346 | NOX5 | NADPH oxidase, EF-hand calcium binding domain 5 | 15 | q23 |
| ENSG00000269099 | LSP1 | Lymphocyte-specific protein 1 | 13 | q12.13 |
| ENSG00000263846 | CIAPIN1P | cytokine induced apoptosis inhibitor 1 pseudogene | 18 | q11.2 |
| ENSG00000262081 | IL9RP4 | interleukin 9 receptor pseudogene 4 | 18 | p11.32 |
| ENSG00000267497 | NFE2L3P1 | nuclear factor (erythroid-derived 2)-like 3 pseudogene 1 | 18 | q21.32 |
| ENSG00000259842 | IGHV3OR16-13 | immunoglobulin heavy variable 3/OR16-13 (non-functional) | 16 | p11.2 |
| ENSG00000262576 | PCDHGA4 | protocadherin gamma subfamily A, 4 | 5 | q31.3 |
| ENSG00000268257 | AIRN | antisense of IGF2R non-protein coding RNA | 6 | q25.3 |
| ENSG00000261548 | HLA-P | major histocompatibility complex, class I, P (pseudogene) | 6 | p22.1 |
| ENSG00000263020 | CSNK2B | Lymphocyte antigen 6 complex, locus G5B | 6 | p21.33 |
| ENSG00000262209 | PCDHGB3 | protocadherin gamma subfamily B, 3 | 5 | q31.3 |
| ENSG00000261934 | PCDHGA9 | protocadherin gamma subfamily A, 9 | 5 | q31.3 |
| ENSG00000269881 | ITFG3 | Protein ITFG3; cDNA FLJ60496 | 16 | p13.3 |
| ENSG00000261832 | CLN3 | Battenin; CLN3 protein; Uncharacterized protein | 16 | p12.1 |
| ENSG00000259997 | IGHV3OR16-8 | immunoglobulin heavy variable 3/OR16-8 (non-functional) | 16 | p11.2 |
| ENSG00000259852 | IGHV1OR16-1 | immunoglobulin heavy variable 1/OR16-1 (pseudogene) | 16 | p11.2 |
| ENSG00000266707 | PSMD7P1 | proteasome (prosome, macropain) 26S subunit, non-ATPase, 7 pseudogene 1 | 17 | q24.1 |
| ENSG00000269179 | SIGLEC11 | Sialic acid-binding Ig-like lectin 11 | 19 | q13.33 |
| ENSG00000264813 | ACE | Angiotensin-converting enzyme; Uncharacterized protein | 17 | q23.3 |
| ENSG00000264257 | KIR3DP1 | killer cell immunoglobulin-like receptor, three domains, pseudogene 1 | 19 | q13.42 |
| ENSG00000268500 | SIGLEC5 | Sialic acid-binding Ig-like lectin 5 | 19 | q13.41 |
| ENSG00000267261 | RAB5C | Ras-related protein Rab-5C | 17 | q21.2 |
| ENSG00000267496 | FAM215A | family with sequence similarity 215, member A (non-protein coding) | 17 | q21.31 |
| ENSG00000268173 | PIK3R2 | PIK3R2 protein | 19 | p13.11 |
| ENSG00000269404 | SPIB | Spi-B transcription factor (Spi-1/PU.1 related) | 19 | q13.33 |
| ENSG00000263528 | IKBKE | inhibitor of kappa light polypeptide gene enhancer in B-cells, kinase epsilon | 1 | q32.1 |
| ENSG00000277632 | CCL3 | chemokine (C-C motif) ligand 3 | 17 | q12 |
| ENSG00000277586 | NEFL | neurofilament, light polypeptide | 8 | p21.2 |
| ENSG00000269335 | IKBKG | inhibitor of kappa light polypeptide gene enhancer in B-cells, kinase gamma | X | q28 |
