# Supplementary material for: HCV eradication with IFN-based therapy does not completely restore gene expression in PBMCs from HIV/HCV-coinfected patients
Source: J Biomed Sci. 2021 Mar 30;28:23. doi: 10.1186/s12929-021-00718-6 (PMC8010945; doi:10.1186/s12929-021-00718-6)
Supplement: Supplementary file 3 — Additional file 3: Table S3. Summary of significant differentially expressed genes (FC ≥1.5; FDR ≤0.05) in peripheral blood mononuclear cells: A) HIV/HCV-b versus HIV-mono. B) HIV/HCV-coinfected at week 24 (HIV/HCV-f) after SVR versus at baseline (HIV/HCV-b). C) HIV/HCV-f versus HIV-mono. [file 12929_2021_718_MOESM3_ESM.docx]

**Supplementary Table 3.** Summary of significant differentially expressed genes (absolute fold-change ≥1.5; FDR ≤0.05) in peripheral blood mononuclear cells: A) HIV/HCV-b versus HIV-mono. B) HIV/HCV-coinfected at week 24 (HIV/HCV-f) after SVR versus at baseline (HIV/HCV-b). C) HIV/HCV-f versus HIV-mono.

| 1. HIV/HCV-b versus HIV-mono | | | | 1. HIV/HCV-f versus HIV/HCV-b | | | | 1. HIV/HCV-f versus HIV-mono | | | |
| --- | --- | --- | --- | --- | --- | --- | --- | --- | --- | --- | --- |
| **Gene symbol** | **log2-ratio** | **p-value** | **FDR** | **Gene symbol** | **log2-ratio** | **p-value** | **FDR** | **Gene symbol** | **log2-ratio** | **p-value** | **FDR** |
| IL23A | 0.989 | ≤0.001 | 0.012 | CXCL2 | 1.533 | ≤0.001 | ≤0.001 | KLF6 | 1.498 | 0.001 | 0.013 |
| FKBP15 | 0.847 | 0.001 | 0.041 | PDCD6IP | 1.357 | ≤0.001 | ≤0.001 | HSPA5 | 1.497 | ≤0.001 | 0.003 |
| CALR | 0.751 | ≤0.001 | 0.034 | ATP5B | 1.300 | ≤0.001 | ≤0.001 | JUN | 1.394 | ≤0.001 | 0.001 |
| DDIT3 | 0.593 | ≤0.001 | 0.029 | IGSF9 | 1.172 | ≤0.001 | ≤0.001 | PRRC2C | 1.165 | ≤0.001 | 0.001 |
| STXBP1 | -0.697 | ≤0.001 | 0.034 | RAB26 | 1.139 | ≤0.001 | ≤0.001 | PPP1R15A | 1.158 | ≤0.001 | 0.001 |
| TLR5 | -1.120 | ≤0.001 | 0.029 | CSRNP1 | 1.102 | ≤0.001 | ≤0.001 | FKBP15 | 0.978 | ≤0.001 | 0.003 |
|  |  |  |  | PRKAG1 | 0.993 | ≤0.001 | ≤0.001 | NFKBIA | 0.917 | ≤0.001 | 0.003 |
|  |  |  |  | NFKB2 | 0.880 | ≤0.001 | ≤0.001 | STAT3 | 0.906 | ≤0.001 | 0.001 |
|  |  |  |  | PELI1 | 0.855 | ≤0.001 | ≤0.001 | NPM1 | 0.798 | 0.001 | 0.009 |
|  |  |  |  | NR1H2 | 0.824 | 0.014 | 0.049 | CDC42 | 0.791 | ≤0.001 | 0.005 |
|  |  |  |  | CCDC85B | 0.813 | 0.003 | 0.014 | CALR | 0.787 | ≤0.001 | 0.001 |
|  |  |  |  | BRE | 0.810 | ≤0.001 | 0.001 | IFT74 | 0.785 | 0.002 | 0.015 |
|  |  |  |  | IL7 | 0.787 | ≤0.001 | ≤0.001 | PIK3R2 | 0.785 | 0.004 | 0.026 |
|  |  |  |  | PMAIP1 | 0.784 | ≤0.001 | ≤0.001 | PRDX3 | 0.779 | ≤0.001 | 0.001 |
|  |  |  |  | SP3 | 0.780 | 0.002 | 0.010 | PI4KB | 0.766 | ≤0.001 | 0.001 |
|  |  |  |  | CDK19 | 0.748 | 0.006 | 0.024 | YWHAZ | 0.748 | 0.004 | 0.025 |
|  |  |  |  | ARFGEF1 | 0.747 | ≤0.001 | ≤0.001 | DDIT3 | 0.721 | ≤0.001 | 0.001 |
|  |  |  |  | PRRC2C | 0.742 | ≤0.001 | ≤0.001 | IL23A | 0.678 | ≤0.001 | 0.003 |
|  |  |  |  | IL6ST | 0.715 | ≤0.001 | ≤0.001 | APOA2 | 0.663 | 0.001 | 0.014 |
|  |  |  |  | NFKBID | 0.688 | ≤0.001 | ≤0.001 | SLC1A2 | 0.654 | ≤0.001 | 0.002 |
|  |  |  |  | RPSA | 0.683 | ≤0.001 | ≤0.001 | THRA | 0.647 | ≤0.001 | 0.003 |
|  |  |  |  | ADAMTS13 | 0.680 | 0.004 | 0.015 | IL16 | 0.635 | 0.002 | 0.017 |
|  |  |  |  | RELB | 0.677 | 0.008 | 0.029 | ICA1L | 0.617 | 0.001 | 0.007 |
|  |  |  |  | CDKN1A | 0.658 | ≤0.001 | ≤0.001 | IRF6 | 0.616 | 0.002 | 0.016 |
|  |  |  |  | THBS3 | 0.658 | 0.007 | 0.027 | CBL | 0.616 | 0.005 | 0.034 |
|  |  |  |  | RHOH | 0.651 | ≤0.001 | ≤0.001 | NFE2L2 | 0.586 | 0.001 | 0.009 |
|  |  |  |  | PDIA3 | 0.638 | ≤0.001 | ≤0.001 | VPS4A | -0.594 | ≤0.001 | 0.005 |
|  |  |  |  | HSPA5 | 0.630 | 0.001 | 0.004 | STXBP1 | -0.594 | 0.006 | 0.035 |
|  |  |  |  | MS4A1 | 0.618 | ≤0.001 | ≤0.001 | RPS6KA2 | -0.611 | 0.006 | 0.035 |
|  |  |  |  | KLF6 | 0.615 | ≤0.001 | ≤0.001 | IL24 | -0.617 | ≤0.001 | 0.004 |
|  |  |  |  | GDF5 | 0.608 | ≤0.001 | ≤0.001 | CENPV | -0.621 | ≤0.001 | 0.003 |
|  |  |  |  | PHLDA3 | 0.608 | 0.011 | 0.038 | ITGA6 | -0.630 | 0.006 | 0.035 |
|  |  |  |  | TREML4 | 0.607 | 0.008 | 0.029 | AMIGO3 | -0.688 | 0.002 | 0.017 |
|  |  |  |  | SPON2 | 0.604 | ≤0.001 | ≤0.001 | ITPR3 | -0.699 | 0.001 | 0.009 |
|  |  |  |  | ARR3 | -0.597 | ≤0.001 | ≤0.001 | NECAP2 | -0.712 | 0.001 | 0.013 |
|  |  |  |  | LHX4 | -0.617 | 0.007 | 0.025 | LHX4 | -0.753 | 0.002 | 0.016 |
|  |  |  |  | HLA-A | -0.631 | ≤0.001 | ≤0.001 | MRAP | -0.767 | ≤0.001 | 0.003 |
|  |  |  |  | MKL1 | -0.634 | 0.001 | 0.007 | CASP6 | -0.790 | ≤0.001 | 0.002 |
|  |  |  |  | RXRA | -0.641 | 0.006 | 0.024 | OBSCN | -0.790 | ≤0.001 | 0.003 |
|  |  |  |  | BID | -0.656 | 0.002 | 0.010 | THBS4 | -0.796 | ≤0.001 | 0.003 |
|  |  |  |  | EPX | -0.664 | 0.003 | 0.013 | CDH12 | -0.818 | ≤0.001 | 0.004 |
|  |  |  |  | IFITM1 | -0.673 | ≤0.001 | ≤0.001 | TRIP10 | -0.860 | ≤0.001 | 0.003 |
|  |  |  |  | ADH5 | -0.694 | ≤0.001 | ≤0.001 | ITGB7 | -0.890 | ≤0.001 | 0.004 |
|  |  |  |  | EPB41L5 | -0.703 | ≤0.001 | ≤0.001 | MKL1 | -0.923 | ≤0.001 | 0.003 |
|  |  |  |  | SIK2 | -0.707 | ≤0.001 | 0.002 |  |  |  |  |
|  |  |  |  | NANS | -0.720 | ≤0.001 | ≤0.001 |  |  |  |  |
|  |  |  |  | RXFP2 | -0.725 | ≤0.001 | ≤0.001 |  |  |  |  |
|  |  |  |  | CXCR2P1 | -0.739 | ≤0.001 | ≤0.001 |  |  |  |  |
|  |  |  |  | ACAN | -0.754 | ≤0.001 | ≤0.001 |  |  |  |  |
|  |  |  |  | KLF10 | -0.799 | ≤0.001 | ≤0.001 |  |  |  |  |
|  |  |  |  | TP73 | -0.806 | ≤0.001 | ≤0.001 |  |  |  |  |
|  |  |  |  | AMIGO3 | -0.825 | ≤0.001 | ≤0.001 |  |  |  |  |
|  |  |  |  | ITGB7 | -0.860 | ≤0.001 | ≤0.001 |  |  |  |  |
|  |  |  |  | NCR3 | -0.929 | ≤0.001 | ≤0.001 |  |  |  |  |
|  |  |  |  | FAT2 | -0.960 | ≤0.001 | ≤0.001 |  |  |  |  |
|  |  |  |  | SIGLEC5 | -0.991 | ≤0.001 | ≤0.001 |  |  |  |  |
|  |  |  |  | IFI44 | -1.247 | ≤0.001 | ≤0.001 |  |  |  |  |
|  |  |  |  | IFI44L | -1.642 | ≤0.001 | 0.002 |  |  |  |  |

**Statistics**: Values expressed as log2ratio. *P-values*, raw *p*-values; *FDR*, *p*-values corrected for multiple testing using the false discovery rate with Benjamini and Hochberg procedure.

**Abbreviations** HIV, human immunodeficiency virus; HCV, hepatitis C virus; HIV/HCV-b, HIV/HCV-coinfected patients at baseline; HIV/HCV-f, HIV/HCV-coinfected patients 24 weeks after SVR; HIV-mono, HIV-monoinfected patients.
