# Supplementary material for: HCV eradication with IFN-based therapy does not completely restore gene expression in PBMCs from HIV/HCV-coinfected patients
Source: J Biomed Sci. 2021 Mar 30;28:23. doi: 10.1186/s12929-021-00718-6 (PMC8010945; doi:10.1186/s12929-021-00718-6)
Supplement: Supplementary file 4 — Additional file 4: Table S4. Summary of significant KEGG pathways (FDR ≤0.05) in the HIV/HCV-f versus HIV/HCV-b comparison. [file 12929_2021_718_MOESM4_ESM.docx]

**Supplementary Table 4**. Summary of significant KEGG pathways (FDR ≤0.05) in the HIV/HCV-f versus HIV/HCV-b comparison.

| **KEGG pathways** | **Hits** | **Genes** | ***q*-values** |
| --- | --- | --- | --- |
| Epstein-Barr virus infection | 6 | BID, CDKN1A, HLA-A, NFKB2, PDIA3, RELB | 0.025 |
| p53 signaling pathway | 4 | BID, CDKN1A, PMAIP1, TP73 | 0.025 |

**Statistical:** *q*-values, *p*-values corrected for multiple testing using the false discovery rate (*FDR*) with Benjamini and Hochberg procedure. In red, up-regulated genes in HIV/HCV-f group; in green, down-regulated genes in HIV/HCV-f group.

**Abbreviations**: FDR, false discovery rate for multiple comparisons using Benjamini and Hochberg procedure; KEGG, Kyoto Encyclopedia of Genes and Genomes; HIV, human immunodeficiency virus; HCV, hepatitis C virus; HIV/HCV-b, HIV/HCV-coinfected patients at baseline; HIV/HCV-f, HIV/HCV-coinfected patients 24 weeks after SVR.
