# Supplementary material for: HCV eradication with IFN-based therapy does not completely restore gene expression in PBMCs from HIV/HCV-coinfected patients
Source: J Biomed Sci. 2021 Mar 30;28:23. doi: 10.1186/s12929-021-00718-6 (PMC8010945; doi:10.1186/s12929-021-00718-6)
Supplement: Supplementary file 5 — Additional file 5: Table S5. Summary of significant KEGG pathways (FDR ≤0.05) in the HIV/HCV-f versus HIV-mono comparison. [file 12929_2021_718_MOESM5_ESM.docx]

**Supplementary Table 5**. Summary of significant KEGG pathways (FDR ≤0.05) in the HIV/HCV-f versus HIV-mono comparison.

| **KEGG pathways** | **Hits** | **Genes** | ***q*-values** |
| --- | --- | --- | --- |
| Apoptosis | 6 | CASP6, DDIT3, ITPR3, JUN, NFKBIA, PIK3R2 | 0.002 |
| Neurotrophin signaling pathway | 5 | JUN, NFKBIA, PIK3R2, CDC42, RPS6KA2 | 0.004 |
| Focal adhesion | 6 | JUN, PIK3R2, CDC42, ITGA6, ITGB7, THBS4 | 0.004 |
| Pathways in cancer | 9 | JUN, NFKBIA, PIK3R2, CDC42, ITGA6, CBL, IL23A, NFE2L2, STAT3 | 0.004 |
| Viral carcinogenesis | 6 | JUN, NFKBIA, PIK3R2, CDC42, STAT3, YWHAZ | 0.004 |
| Hepatitis B | 5 | JUN, NFKBIA, PIK3R2, STAT3, YWHAZ | 0.013 |
| Protein processing in endoplasmic reticulum | 5 | DDIT3, NFE2L2, CALR, HSPA5, PPP1R15A | 0.013 |
| AGE-RAGE signaling pathway in diabetic complications | 4 | JUN, PIK3R2, CDC42, STAT3 | 0.014 |
| T cell receptor signaling pathway | 4 | JUN, NFKBIA, PIK3R2, CDC42 | 0.014 |
| Chagas disease (American trypanosomiasis) | 4 | JUN, NFKBIA, PIK3R2, CALR | 0.014 |
| Kaposi's sarcoma-associated herpesvirus infection | 5 | ITPR3, JUN, NFKBIA, PIK3R2, STAT3 | 0.014 |
| Th17 cell differentiation | 4 | JUN, NFKBIA, IL23A, STAT3 | 0.014 |
| Insulin resistance | 4 | NFKBIA, PIK3R2, RPS6KA2, STAT3 | 0.014 |
| Epstein-Barr virus infection | 5 | JUN, NFKBIA, PIK3R2, STAT3, CALR | 0.014 |
| Proteoglycans in cancer | 5 | ITPR3, PIK3R2, CDC42, CBL, STAT3 | 0.014 |
| Measles | 4 | JUN, NFKBIA, PIK3R2, STAT3 | 0.028 |
| Inflammatory bowel disease (IBD) | 3 | JUN, IL23A, STAT3 | 0.030 |
| Epithelial cell signaling in Helicobacter pylori infection | 3 | JUN, NFKBIA, CDC42 | 0.030 |
| Non-alcoholic fatty liver disease (NAFLD) | 4 | DDIT3, JUN, PIK3R2, CDC42 | 0.030 |
| Renal cell carcinoma | 3 | JUN, PIK3R2, CDC42 | 0.030 |
| B cell receptor signaling pathway | 3 | JUN, NFKBIA, PIK3R2 | 0.031 |
| Hepatitis C | 4 | NFKBIA, PIK3R2, STAT3, YWHAZ | 0.031 |
| Bacterial invasion of epithelial cells | 3 | PIK3R2, CDC42, CBL | 0.031 |
| Pancreatic cancer | 3 | PIK3R2, CDC42, STAT3 | 0.031 |
| Chronic myeloid leukemia | 3 | NFKBIA, PIK3R2, CBL | 0.031 |
| Jak-STAT signaling pathway | 4 | PIK3R2, IL23A, STAT3, IL24 | 0.031 |
| ECM-receptor interaction | 3 | ITGA6, ITGB7, THBS4 | 0.037 |
| ErbB signaling pathway | 3 | JUN, PIK3R2, CBL | 0.039 |
| GnRH signaling pathway | 3 | ITPR3, JUN, CDC42 | 0.047 |
| Small cell lung cancer | 3 | NFKBIA, PIK3R2, ITGA6 | 0.047 |
| Chemokine signaling pathway | 4 | NFKBIA, PIK3R2, CDC42, STAT3 | 0.047 |

**Statistical**: FDR, false discovery rate for multiple comparisons using Benjamini and Hochberg procedure. In red, up-regulated genes in HIV/HCV-f group; in green, down-regulated genes in HIV/HCV-f group.

**Abbreviations**: KEGG, Kyoto Encyclopedia of Genes and Genomes; HIV, human immunodeficiency virus; HCV, hepatitis C virus; HIV/HCV-f, HIV/HCV-coinfected patients 24 weeks after SVR; HIV-mono, HIV-monoinfected patients.
