# Supplementary material for: HCV eradication with IFN-based therapy does not completely restore gene expression in PBMCs from HIV/HCV-coinfected patients
Source: J Biomed Sci. 2021 Mar 30;28:23. doi: 10.1186/s12929-021-00718-6 (PMC8010945; doi:10.1186/s12929-021-00718-6)
Supplement: Supplementary file 6 — Additional file 6: Table S6. Summary of the most deregulated SDE genes (absolute FC ≥2 and FDR ≤0.05). [file 12929_2021_718_MOESM6_ESM.docx]

**Supplementary Table 6**. Summary of the most deregulated SDE genes (absolute FC ≥ 2 and FDR ≤ 0.05).

| 1. **A) HIV/HCV-f versus HIV/HCV-b (absolute FC ≥2)** | | |
| --- | --- | --- |
| Down | *IFI44*  *IFI44L* | - *IFI44 and IFI44L* are two of the multiple IFN-stimulated genes (ISGs) induced during chronic hepatitis C. Underexpression of *IFI44L* is associated with the development of hepatocellular carcinoma (HCC) [1]. Downregulation of *IFI44* and *IFI44L* in PBMCs are potential diagnostic biomarkers of solid tumors such as HCC [2]. - *IFI44* and *IFI44L* are also involved in controlling HIV replication, and their underexpression may favor the reactivation of HIV proviruses [3-5]. However, there is no evidence of any peg-IFN effect on total HIV-1 DNA load among HIV/HCV-coinfected patients [6]. |
| Up | *CXCL2*  *PDCD6IP*  *ATP5B*  *IGSF9*  *RAB26*  *CSRNP1* | - *CXCL2* encodes a chemokine involved in inflammatory processes related to healing, cancer, and angiogenesis [7]. Increased CXCL2 levels may promote liver regeneration [8], although high CXCL2 levels have also been linked to the development of non-alcoholic fatty liver disease (NAFLD) [9]. Therefore, the *CXCL2* overexpression in our patients could promote liver regeneration and protect against the development of HCC, but it could also foster NAFLD development. - *PDCD6IP* encodes a protein involved in many cell processes. *PDCD6IP* overexpression hinders apoptosis, making it a protective marker of HCC [10]. However, PDCD6IP mediates HIV budding and release, facilitating membrane scission at the plasma membrane [11], increasing HIV replication fitness [12]. In our patients, *PDCD6IP* overexpression may protect against HCC but also may promote HIV replication. - *ATP5B* encodes a subunit of mitochondrial ATP synthase, which leads to mitochondrial production of ATP and ROS (mtROS) [13]. *ATP5B* overexpression is related to the development of HCC [14, 15] and NAFLD [16]. In our study, the *ATP5B* overexpression could be due to a rebound effect by HCV elimination, which could favor liver disease progression. - *IGSF9* encodes an adhesion molecule that participates in cell-cell adhesion mediator activity, dendrite outgrowth, and synapse maturation, which have a relevant role in the development of the nervous system [17]. However, there is limited information on liver disease and HIV infection. - *RAB26* encodes a Rab GTPase that regulates the vesicular fusion and intracellular membrane trafficking [18]. *RBA26* overexpression negatively regulates the TLR4 expression and attenuates the inflammatory response in the endothelial barrier [19, 20]. Thus, *RAB26* overexpression could be a critical factor in reducing inflammation and chronic activation after achieving SVR. - *CSRNP1* encodes a protein that can have a tumor suppressor function, and its overexpression is linked to a lower risk of HCC [21, 22]. *CSRNP1* also induces the production of the matrix metalloproteinases 1 (MMPs)-1 [23], a key enzyme that promotes the breakdown of fibrillar collagens from the extracellular matrix, contributing to the liver fibrosis regression. |
| 1. **B) HIV/HCV-f versus HIV-mono (absolute FC ≥2)** | | |
| Up | *KLF6*  *HSPA5*  *JUN*  *PRRC2C*  *PPP1R15A* | - *KLF6* encodes a transcription factor that may act as a regenerator of liver fibrosis, steatosis, and HCC, because when *KLF6* is downregulated or absent, it causes the appearance of liver lesions [24-26]. - *HSPA5* encodes a chaperone located in the endoplasmic reticulum (ER), which is abundant under cell growth conditions and when there is an accumulation of unfolded polypeptides under ER stress. *HSPA5* overexpression promotes mechanisms that attempt to re-establish homeostasis, reducing NASH, and NAFLD [27]. - *JUN* encodes a subunit of the AP-1 transcription factor that promotes inflammation and insulin resistance. *JUN* overexpression promotes liver fibrosis and correlates with progression from steatosis to NASH [28]. - *PRRC2C* is a protein encoding gene for which there is limited information on its function and associated diseases. - *PPP1R15A* (also called GADD34) encodes a protein that helps protein phosphatase 1α (PP1α) to dephosphorylate eIF2α and restore translation in the unfolded protein response (UPR) during ER stress, recovering global protein synthesis [29]. In the liver, PPP1R15A shows a positive role in liver regeneration after stress-induced damage [30]. PPP1R15A is also linked to innate immune responses and has an anti-inflammatory role through suppressing macrophage activation [31]. Furthermore, PPP1R15A overexpression inhibits HIV-1 replication [32]. |

**Abbreviations**: HIV, human immunodeficiency virus; HCV, hepatitis C virus; HIV/HCV-b, HIV/HCV-coinfected patients at baseline; HIV/HCV-f, HIV/HCV-coinfected patients 24 weeks after SVR; HIV-mono, HIV-monoinfected patients; SDE, significantly differentially expressed; FDR, false discovery rate for multiple comparisons; FC, fold-change; C-X-C motif chemokine ligand 2 (CXCL2), programmed cell death 6 interacting protein (PDCD6IP), ATP synthase F1 subunit beta (ATP5B), immunoglobulin superfamily member 9 (IGSF9), Ras-related protein Rab-26 (RAB26), and cysteine and serine-rich nuclear protein 1 (CSRNP1), interferon-induced protein 44 (IFI44) and interferon-induced protein 44-like (IFI44L), Kruppel like factor 6 (KLF6), heat shock 70 kDa protein 5 (HSPA5), Jun Proto-Oncogene (JUN), Proline-Rich Coiled-Coil 2C (PRRC2C), and Protein Phosphatase 1 Regulatory Subunit 15A (PPP1R15A).

1. **References:**
2. 1. Huang WC, Tung SL, Chen YL, Chen PM, Chu PY: **IFI44L is a novel tumor suppressor in human hepatocellular carcinoma affecting cancer stemness, metastasis, and drug resistance via regulating met/Src signaling pathway**. *BMC Cancer* 2018, **18**(1):609.
3. 2. Chen S, Liu M, Liang B, Ge S, Peng J, Huang H, Xu Y, Tang X, Deng L: **Identification of human peripheral blood monocyte gene markers for early screening of solid tumors**. *PLoS One* 2020, **15**(3):e0230905.
4. 3. Power D, Santoso N, Dieringer M, Yu J, Huang H, Simpson S, Seth I, Miao H, Zhu J: **IFI44 suppresses HIV-1 LTR promoter activity and facilitates its latency**. *Virology* 2015, **481**:142-150.
5. 4. Papasavvas E, Azzoni L, Kossenkov AV, Dawany N, Morales KH, Fair M, Ross BN, Lynn K, Mackiewicz A, Mounzer K *et al*: **NK Response Correlates with HIV Decrease in Pegylated IFN-alpha2a-Treated Antiretroviral Therapy-Suppressed Subjects**. *J Immunol* 2019, **203**(3):705-717.
6. 5. McLaren PJ, Gawanbacht A, Pyndiah N, Krapp C, Hotter D, Kluge SF, Gotz N, Heilmann J, Mack K, Sauter D *et al*: **Identification of potential HIV restriction factors by combining evolutionary genomic signatures with functional analyses**. *Retrovirology* 2015, **12**:41.
7. 6. Strouvelle VP, Braun DL, Vongrad V, Scherrer AU, Kok YL, Kouyos RD, Stöckle M, Rauch A, Darling K, Hoffmann M *et al*: **No Effect of Pegylated Interferon-α on Total HIV-1 DNA Load in HIV-1/HCV Coinfected Patients**. *J Infect Dis* 2018, **217**(12):1883-1888.
8. 7. Rajarathnam K, Schnoor M, Richardson RM, Rajagopal S: **How do chemokines navigate neutrophils to the target site: Dissecting the structural mechanisms and signaling pathways**. *Cell Signal* 2019, **54**:69-80.
9. 8. Qin CC, Liu YN, Hu Y, Yang Y, Chen Z: **Macrophage inflammatory protein-2 as mediator of inflammation in acute liver injury**. *World J Gastroenterol* 2017, **23**(17):3043-3052.
10. 9. Zhang X, Fan L, Wu J, Xu H, Leung WY, Fu K, Wu J, Liu K, Man K, Yang X *et al*: **Macrophage p38alpha promotes nutritional steatohepatitis through M1 polarization**. *J Hepatol* 2019, **71**(1):163-174.
11. 10. Yu Q, Zhou C, Wang J, Chen L, Zheng S, Zhang J: **A functional insertion/deletion polymorphism in the promoter of PDCD6IP is associated with the susceptibility of hepatocellular carcinoma in a Chinese population**. *DNA Cell Biol* 2013, **32**(8):451-457.
12. 11. Votteler J, Sundquist WI: **Virus budding and the ESCRT pathway**. *Cell Host Microbe* 2013, **14**(3):232-241.
13. 12. van Domselaar R, Njenda DT, Rao R, Sonnerborg A, Singh K, Neogi U: **HIV-1 Subtype C with PYxE Insertion Has Enhanced Binding of Gag-p6 to Host Cell Protein ALIX and Increased Replication Fitness**. *J Virol* 2019, **93**(9).
14. 13. Chung IC, Chen LC, Tsang NM, Chuang WY, Liao TC, Yuan SN, OuYang CN, Ojcius DM, Wu CC, Chang YS: **Mitochondrial Oxidative Phosphorylation Complex Regulates NLRP3 Inflammasome Activation and Predicts Patient Survival in Nasopharyngeal Carcinoma**. *Mol Cell Proteomics* 2020, **19**(1):142-154.
15. 14. Gerresheim GK, Roeb E, Michel AM, Niepmann M: **Hepatitis C Virus Downregulates Core Subunits of Oxidative Phosphorylation, Reminiscent of the Warburg Effect in Cancer Cells**. *Cells* 2019, **8**(11).
16. 15. Santacatterina F, Sanchez-Cenizo L, Formentini L, Mobasher MA, Casas E, Rueda CB, Martinez-Reyes I, Nunez de Arenas C, Garcia-Bermudez J, Zapata JM *et al*: **Down-regulation of oxidative phosphorylation in the liver by expression of the ATPase inhibitory factor 1 induces a tumor-promoter metabolic state**. *Oncotarget* 2016, **7**(1):490-508.
17. 16. Mazumder R: **Sites of action of fusidic acid in eukaryotes. Inhibition by fusidic acid of a ribosome-independent GTPase from Artemia salina embryos**. *Eur J Biochem* 1975, **58**(2):549-554.
18. 17. Hansen M, Walmod PS: **IGSF9 family proteins**. *Neurochem Res* 2013, **38**(6):1236-1251.
19. 18. Wei Z, Zhang M, Li C, Huang W, Fan Y, Guo J, Khater M, Fukuda M, Dong Z, Hu G *et al*: **Specific TBC Domain-Containing Proteins Control the ER-Golgi-Plasma Membrane Trafficking of GPCRs**. *Cell Rep* 2019, **28**(2):554-566 e554.
20. 19. Chen H, Yuan M, Huang C, Xu Z, Li M, Zhang C, Gao Z, Zhang M, Xu J, Qian H *et al*: **Endothelial Cell Inflammation and Barriers Are Regulated by the Rab26-Mediated Balance between beta2-AR and TLR4 in Pulmonary Microvessel Endothelial Cells**. *Mediators Inflamm* 2019, **2019**:7538071.
21. 20. Li H, He B, Liu X, Li J, Liu Q, Dong W, Xu Z, Qian G, Zuo H, Hu C *et al*: **Regulation on Toll-like Receptor 4 and Cell Barrier Function by Rab26 siRNA-loaded DNA Nanovector in Pulmonary Microvascular Endothelial Cells**. *Theranostics* 2017, **7**(9):2537-2554.
22. 21. Qin A, Wu J, Zhai M, Lu Y, Huang B, Lu X, Jiang X, Qiao Z: **Axin1 inhibits proliferation, invasion, migration and EMT of hepatocellular carcinoma by targeting miR-650**. *Am J Transl Res* 2020, **12**(3):1114-1122.
23. 22. Xu B, Lv W, Li X, Zhang L, Lin J: **Prognostic genes of hepatocellular carcinoma based on gene coexpression network analysis**. *J Cell Biochem* 2019.
24. 23. Macdonald CD, Falconer AMD, Chan CM, Wilkinson DJ, Skelton A, Reynard L, Litherland GJ, Europe-Finner GN, Rowan AD: **Cytokine-induced cysteine- serine-rich nuclear protein-1 (CSRNP1) selectively contributes to MMP1 expression in human chondrocytes**. *PLoS One* 2018, **13**(11):e0207240.
25. 24. Lu XJ, Shi Y, Chen JL, Ma S: **Kruppel-like factors in hepatocellular carcinoma**. *Tumour Biol* 2015, **36**(2):533-541.
26. 25. Ghiassi-Nejad Z, Hernandez-Gea V, Woodrell C, Lang UE, Dumic K, Kwong A, Friedman SL: **Reduced hepatic stellate cell expression of Kruppel-like factor 6 tumor suppressor isoforms amplifies fibrosis during acute and chronic rodent liver injury**. *Hepatology* 2013, **57**(2):786-796.
27. 26. Bechmann LP, Vetter D, Ishida J, Hannivoort RA, Lang UE, Kocabayoglu P, Fiel MI, Munoz U, Patman GL, Ge F *et al*: **Post-transcriptional activation of PPAR alpha by KLF6 in hepatic steatosis**. *J Hepatol* 2013, **58**(5):1000-1006.
28. 27. Malhi H, Kaufman RJ: **Endoplasmic reticulum stress in liver disease**. *J Hepatol* 2011, **54**(4):795-809.
29. 28. Schulien I, Hockenjos B, Schmitt-Graeff A, Perdekamp MG, Follo M, Thimme R, Hasselblatt P: **The transcription factor c-Jun/AP-1 promotes liver fibrosis during non-alcoholic steatohepatitis by regulating Osteopontin expression**. *Cell Death Differ* 2019, **26**(9):1688-1699.
30. 29. Novoa I, Zeng H, Harding HP, Ron D: **Feedback inhibition of the unfolded protein response by GADD34-mediated dephosphorylation of eIF2alpha**. *The Journal of cell biology* 2001, **153**(5):1011-1022.
31. 30. Inaba Y, Furutani T, Kimura K, Watanabe H, Haga S, Kido Y, Matsumoto M, Yamamoto Y, Harada K, Kaneko S *et al*: **Growth arrest and DNA damage-inducible 34 regulates liver regeneration in hepatic steatosis in mice**. *Hepatology* 2015, **61**(4):1343-1356.
32. 31. Ito S, Tanaka Y, Oshino R, Okado S, Hori M, Isobe KI: **GADD34 suppresses lipopolysaccharide-induced sepsis and tissue injury through the regulation of macrophage activation**. *Cell death & disease* 2016, **7**:e2219.
33. 32. Ishaq M, Marshall H, Natarajan V: **GADD34 attenuates HIV-1 replication by viral 5'-UTR TAR RNA-mediated translational inhibition**. *Virology* 2020, **540**:119-131.
