# Supplementary material for: HCV eradication with IFN-based therapy does not completely restore gene expression in PBMCs from HIV/HCV-coinfected patients
Source: J Biomed Sci. 2021 Mar 30;28:23. doi: 10.1186/s12929-021-00718-6 (PMC8010945; doi:10.1186/s12929-021-00718-6)
Supplement: Supplementary file 7 — Additional file 7: Appendix S1. The GESIDA 3603b Cohort Study Group. [file 12929_2021_718_MOESM7_ESM.docx]

# Appendix

**The GESIDA 3603b Cohort Study Group**

***Hospital General Universitario Gregorio Marañón, Madrid:*** A Carrero, P Miralles, JC López, F Parras, B Padilla, T Aldamiz-Echevarría, F Tejerina, C Díez, L Pérez-Latorre, C Fanciulli, I Gutiérrez, M Ramírez, S Carretero, JM Bellón, J Bermejo, and J Berenguer.

***Hospital Universitario La Paz, Madrid:*** V Hontañón, JR Arribas, ML Montes, I Bernardino, JF Pascual, F Zamora, JM Peña, F Arnalich, M Díaz, J González-García.

***Hospital de la Santa Creu i Sant Pau, Barcelona:*** P Domingo, JM Guardiola.

***Hospital Universitari Vall d'Hebron, Barcelona:*** E Van den Eynde, M Pérez, E Ribera, M Crespo.

***Hospital Universitario Ramón y Cajal, Madrid:*** JL Casado, F Dronda, A Moreno, MJ Pérez-Elías, MA Sanfrutos, S Moreno, C Quereda.

***Hospital Universitario Príncipe de Asturias, Alcalá de Henares:*** A Arranz, E Casas, J de Miguel, S Schroeder, J Sanz.

***Hospital Universitario de La Princesa, Madrid:*** J Sanz, I Santos.

***Hospital Donostia, San Sebastián:*** MJ Bustinduy, JA Iribarren, F Rodríguez-Arrondo, MA Von-Wichmann.

***Hospital Clínico San Carlos, Madrid:*** J Vergas, MJ Téllez.

**Hospital Universitario San Cecilio, Granada**: D. Vinuesa, L. Muñoz, and J. Hernández-Quero.

***Hospital Clínico Universitario, Valencia:*** A Ferrer, MJ Galindo.

***Hospital General Universitario, Valencia:*** L Ortiz, E Ortega.

***Hospital Universitari La Fe, Valencia:*** M Montero, M Blanes, S Cuellar, J Lacruz, M Salavert, J López-Aldeguer.

***Hospital Universitario de Getafe, Getafe:*** G Pérez, G Gaspar.

***Fundación SEIMC-GESIDA, Madrid:*** M Yllescas, P Crespo, E Aznar, H Esteban
